# A General Iridium-Catalyzed Reductive Dienamine Synthesis Allows a Five-Step Synthesis of Catharanthine via the Elusive Dehydrosecodine

**DOI:** 10.1021/jacs.1c04980

**Published:** 2021-07-13

**Authors:** Pablo Gabriel, Yaseen A. Almehmadi, Zeng Rong Wong, Darren J. Dixon

**Affiliations:** † Department of Chemistry, Chemistry Research Laboratory, University of Oxford, 12 Mansfield Road, Oxford OX1 3TA, United Kingdom; ‡ Department of Chemistry, Rabigh College of Science and Arts, King Abdulaziz University, Jeddah 21589, Saudi Arabia

## Abstract

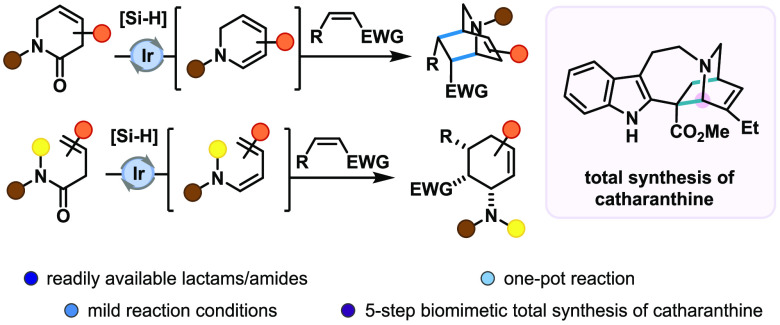

A new reductive strategy for the stereo- and regioselective synthesis of functionalized isoquinuclidines has been developed. Pivoting on the chemoselective iridium(I)-catalyzed reductive activation of β,γ-unsaturated δ-lactams, the efficiently produced reactive dienamine intermediates readily undergo [4 + 2] cycloaddition reactions with a wide range of dienophiles, resulting in the formation of bridged bicyclic amine products. This new synthetic approach was extended to aliphatic starting materials, resulting in the efficient formation of cyclohexenamine products, and readily applied as the key step in the shortest (five-step) total synthesis of vinca alkaloid catharanthine to date, proceeding via its elusive biosynthetic precursor, dehydrosecodine.

Saturated and semisaturated nitrogen-containing heterocycles are prevalent structures in bioactive natural products and pharmaceutical compounds,^1^ and accordingly, new strategic approaches for their efficient and selective synthesis are important. In parallel, Diels–Alder reactions have been—for nearly a century—one of the most powerful tools for the construction of cyclic and polycyclic products, allowing the disconnection of six-membered rings to a four-electron diene component and a two-electron dienophile.^2,3^ In the normal electron demand Diels–Alder reaction, electron-rich dienes locked in the reactive *s-cis* conformation are exceptionally reactive. As such, 1,2-dihydropyridines **1** are a class of compounds particularly poised for cycloaddition reactions, producing the 2-azabicyclo[2.2.2]octane ring system **2**, also called isoquinuclidine (Scheme 1a).^4^ This bridged nitrogen-containing bicycle is a familiar structural feature in a range of alkaloid natural products, for instance, catharanthine (**3**), cononusine (**4**), and caldaphinidine D (**5**) (Scheme 1b).^5^ Additionally, isoquinuclidines have been used as intermediates toward octahydroisoquinolines in drugs and natural products, such as pseudotabersonine (**6**) and oseltamivir (**7**) (Scheme 1c).^6^

**Scheme 1:**
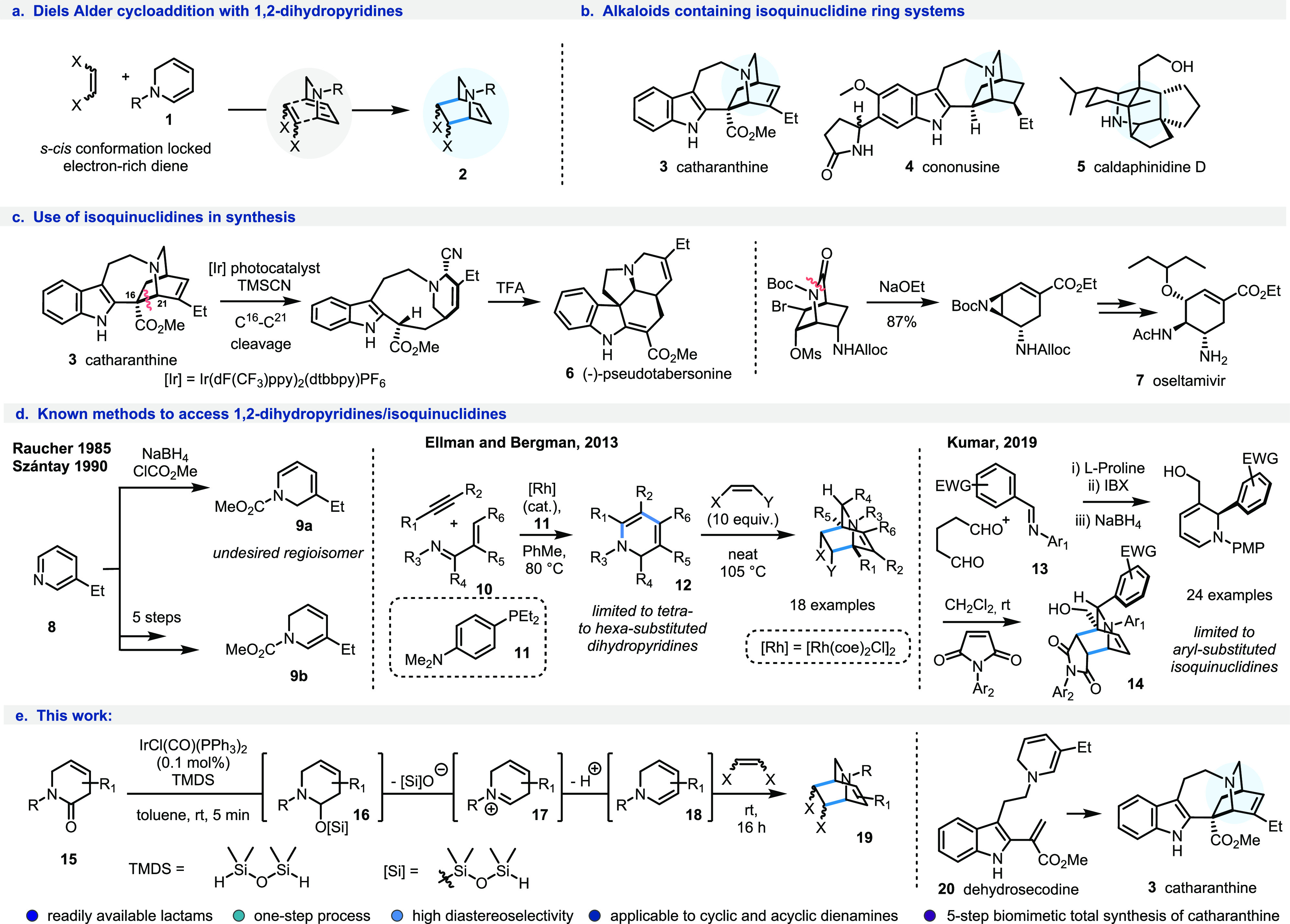
(a) Diels–Alder Cycloadditions of 1,2-Dihydropyridines; (b) Isoquinuclidine-Containing Natural Products; (c) Use of Isoquinuclidines in Synthesis; (d) Existing Methods (and Limitations) toward the Synthesis of 1,2-Dihydropyridines and Downstream Isoquinuclidines; and (e) This Work

To date, because of their inherent instability, the selective and efficient generation of electron-rich 1,2-dihydropyridines has been challenging, and in most cases the presence of a carbamoyl, or similar, electron-withdrawing group on the nitrogen atom is required to make them sufficiently stable for downstream manipulation, albeit at the expense of further deprotection steps or functional group manipulation.^7^ Other methods rely on the partial reduction of, or nucleophilic addition to, pyridinium species (Scheme 1d1),^8^ but indirect strategies are often required to circumvent the undesired or imperfect regioselectivity in the borohydride-mediated reduction^7b,7c,7f^ or nucleophilic addition. More recently, highly substituted (and inherently more stable) 1,2-dihydropyridines such as **12** have been generated via Rh-catalyzed C–H activation of α,β-unsaturated imines **10** (Scheme 1d2)^9^ as well as via multistep cascade reactions involving proline-catalyzed Mannich cyclization followed by oxidation and reduction (Scheme 1d3).^10^ Notwithstanding these elegant reports, only specific substitution patterns are currently accessible,^7−10^ and a general strategy for the controlled synthesis of electron-rich 1,2-dihydropyridines currently remains elusive.

Because of the important role of these compounds, and the challenges associated with their generation, we recognized that a mild and general reductive functionalization approach to access 1,2-dihydropyridines using readily available lactam starting materials could be of high synthetic value. Mechanistic studies from our group on the iridium-catalyzed reductive nitro-Mannich reaction revealed that tertiary lactams have a strong propensity to form enamines from the silylated hemiaminal intermediates via their corresponding iminium species.^11a−11f^ Aware of this, and the tolerance of alkene moieties to the reductive activation conditions,^11g−11v^ we reasoned that in the presence of suitably placed β,γ-unsaturation in the lactam ring of **15** (Scheme 1e), the 1,2-dihydropyridine species would likely arise from iminium ion **17** via silylated hemiaminal **16**. Reactive conjugated dienamine intermediates such as **18** are primed for downstream cycloaddition reactions with various dienophiles, and granting new access to them via a reductive manifold would provide a wealth of opportunities in both library generation, and natural product synthesis alike; herein we wish to report our findings.

We began our studies with a ^1^H NMR experiment to assess the feasibility of formation of the desired dienamine from lactam precursors (Figure 1). We subjected the model *N*-benzyl β,γ-unsaturated δ-lactam substrate **15a** to standard reduction conditions in *d*_8_-toluene (0.1 mol % of Vaska’s complex and 2 equiv of TMDS),^12^ and very pleasingly, after 20 min, we observed a clean ^1^H NMR spectrum fully assignable to dihydropyridine **18a**.^13^ Because of the expected instability of this intermediate, we chose to add in one portion the reactive dienophile *N*-phenylmaleimide **21a** directly to the reaction mixture, and indeed the desired [4 + 2] cycloadduct **19a** was formed as the major reaction product (along with TMDS-derived side-products) in 93% NMR yield and as the *endo* diastereoisomer.

**Figure 1:**
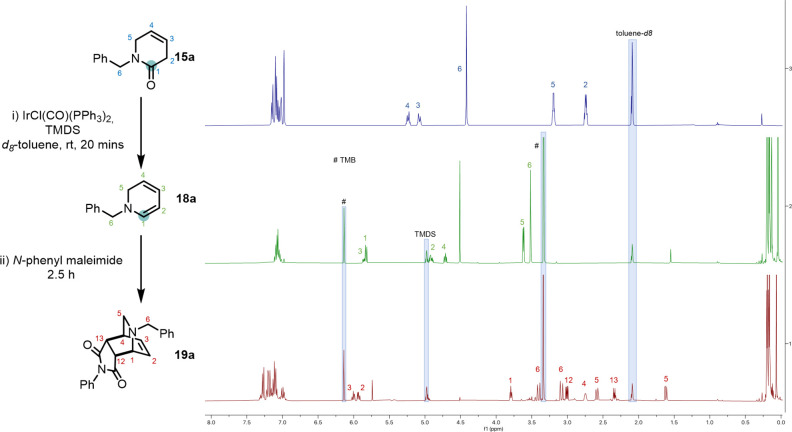
^1^H NMR spectra of the reduction of lactam **15a** to the dienamine **18a** and downstream cycloaddition with *N*-phenylmaleimide. Reaction performed in *d*_8_-toluene, in an NMR tube; 1,3,5-trimethoxybenzene (TMB) was used as internal standard.

Encouraged by these preliminary data, we began investigating the scope of this reaction by varying the substituents and substitution patterns on the lactam substrate (Scheme 2). These substrates were accessible via α-functionalization of the parent lactam (**15b**, **15d**), already known in the literature (**15c**, **15e**),^14^ or synthesized using a recently developed three-component reaction (**15f**, **15g**).^15^ We were pleased to find that, when used in conjunction with *N*-phenylmaleimide (1.05 equiv) as the dienophile, the corresponding cycloadducts of increasing complexity **19a**–**19g** could be isolated in good to excellent yields and with essentially complete diastereoselectivity.

**Scheme 2:**
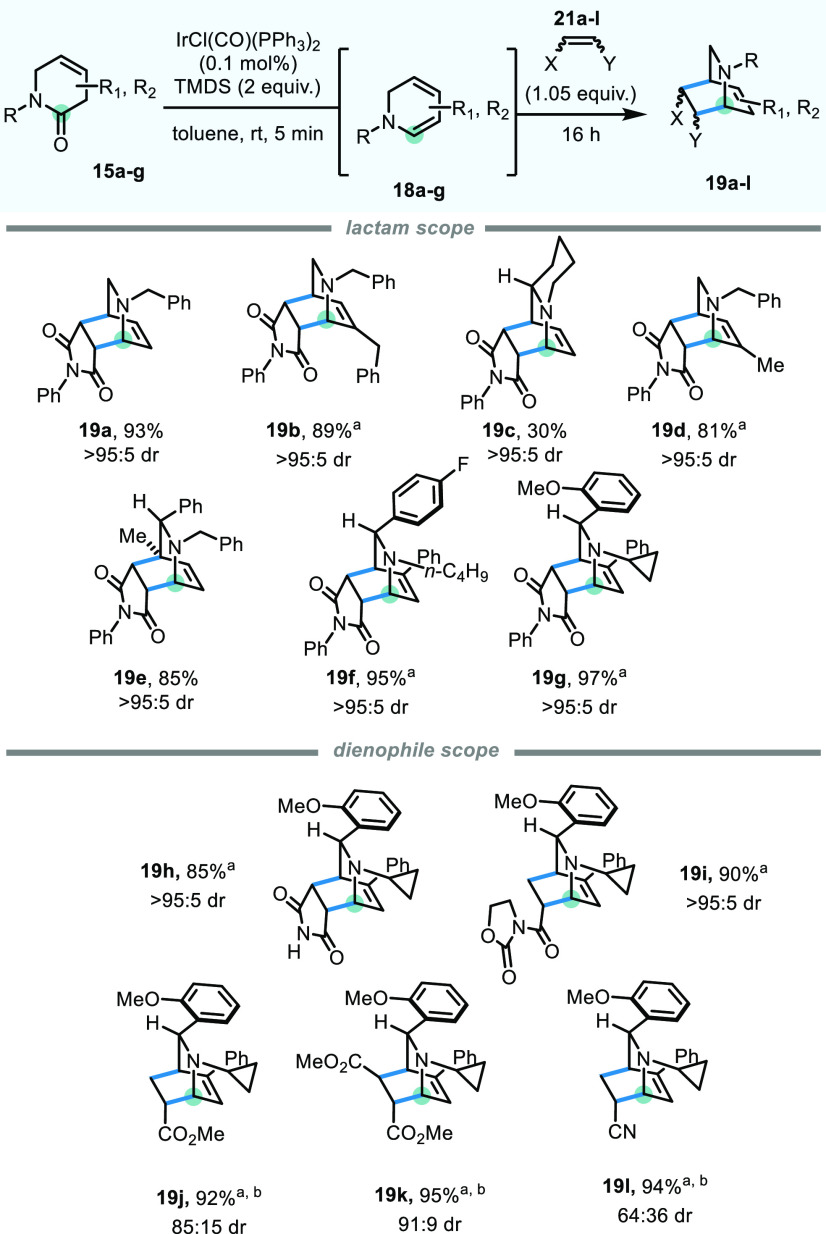
Scope of the Isoquinuclidine-Generating Methodology

Modification of the substitution on the nitrogen atom showed that reactivity was not diminished when using linear (**19f**) or alicyclic side-chains (**19g**–**19l**). Keeping **15g** as the parent lactam, we also explored the range of dienophiles that could be successfully deployed in the cycloaddition step. Pleasingly, the use of maleimide **21h** as the dienophile resulted in a smooth reaction, providing **19h** in excellent 85% yield and >95:5 dr, while oxazolidinone **21i** reacted similarly, forming **19i** in 90% yield and >95:5 dr. Methyl acrylate (**21j**), dimethyl fumarate (**21k**), and acrylonitrile (**21l**) also led to the formation of the respective cycloadducts **19j**, **19k**, and **19l**, albeit with imperfect diastereoselectivity (85:15, 91:9, and 64:36 dr, respectively).

Having successfully established a scope for the formation of isoquinuclidines from unsaturated δ-lactams, we turned our attention to acyclic systems. Simple β,γ-unsaturated amides are indeed readily available from secondary amines via coupling with 3-butenoic acid. Our hope was that our newly developed methodology could be extended to the generation of acyclic dienamine species that, in turn, could be valuable intermediates for the formation of tertiary amine-appended cyclohexene architectures, with potential control of up to four newly formed stereocenters.^16^

Although the reduction step required longer reaction times than for cyclic systems (3 h, see Scheme 3), we were pleased to find that but-3-enamides **22a**–**c** did indeed form the desired dienamines **23a**–**c** and the downstream cyclohexene structures **24a**–**f** with complete diastereocontrol upon reaction with *N*-phenylmaleimide or other dienophiles in good to excellent yields. Moving away from simple but-3-enamides, indole substrate **25a**,**b**, where the β,γ-unsaturation is an integral part of the heteroaromatic ring, also produced the desired cycloadducts **26a**,**b**. For ease of isolation, these were further oxidized by addition of DDQ at the end of the reaction and isolated as the aromatized β-carbolines **27a** and **27b** in 77% and 89% yield, respectively. Finally, both amide functional groups within succinamide **28** could be reduced to their respective enamine intermediates, forming overall a symmetric bisamino-diene species **29** that underwent cycloaddition to furnish symmetric tetrasubstituted **30** as a single isomer. Remarkably, during the course of this reaction, all six carbons contained within the final cyclohexene product saw their hybridization state change from sp^3^ to sp^2^ (or vice versa), resulting in a relatively complex architecture arising in a single-pot transformation from a simple building block.

**Scheme 3:**
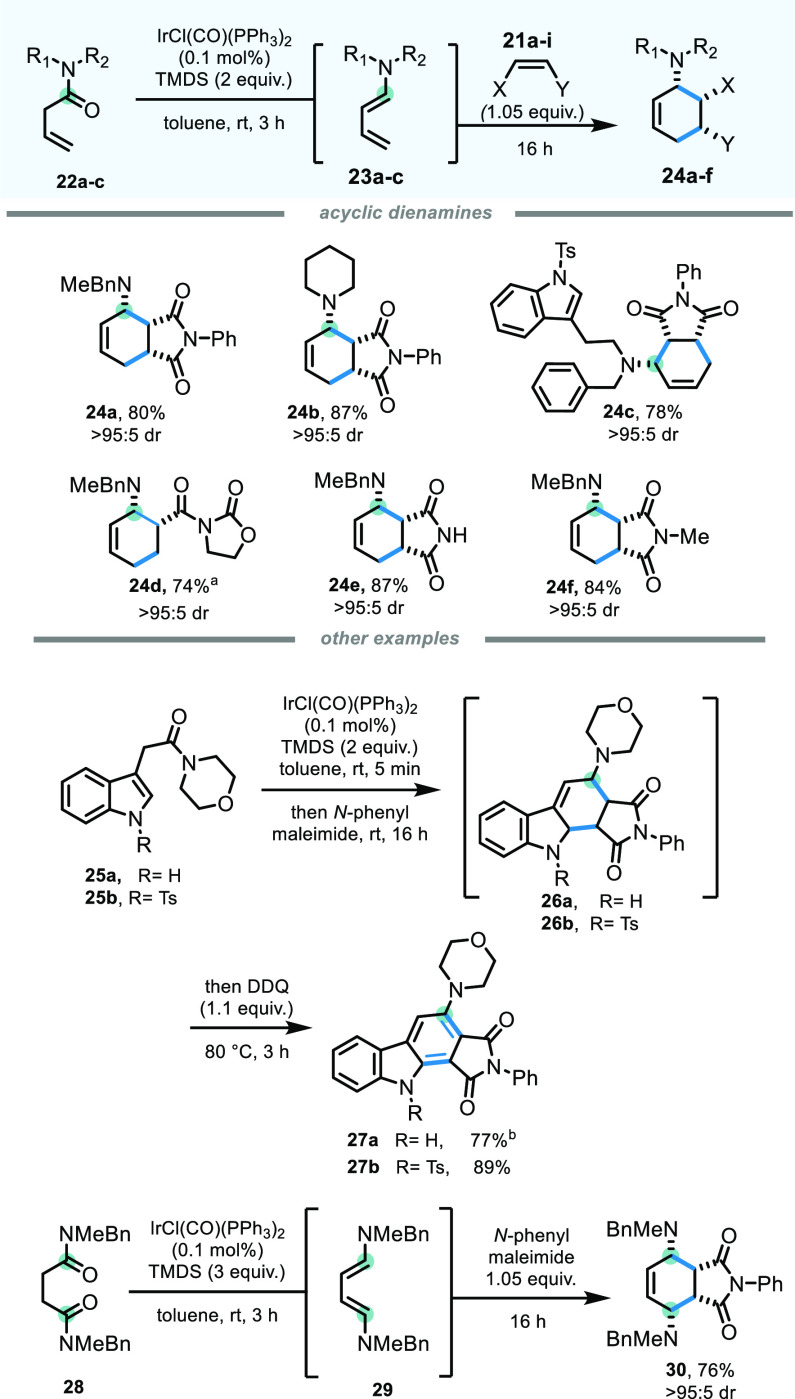
Extension to Acyclic Dienamine Generation/[4 + 2] Cycloaddition Reactions

To firmly establish this reductive dienamine generation strategy in complex natural product total synthesis, we set our sights on one of the most important yet elusive intermediates in monoterpene indole alkaloid natural products chemistry, dehydrosecodine (**20**). Since the pioneering studies of Wenkert in 1962,^17^ Scott,^18a^ and recently De Luca^18b^ and O’Connor,^18c−18e^ this functionally rich molecular entity has been putatively identified as the common precursor to a wide variety of skeletally varied Vinca, Iboga, and Aspidospema alkaloids.^18f^ Possessing a 1,2-dihydropyridine motif capable of meeting either the electronic demands of a diene (normal electron demand Diels–Alder cycloaddition toward catharanthine **3**; see Scheme 4a) or a dienophile (inverse electron demand Diels–Alder cycloaddition toward tabersonine **31**),^19^ dehydrosecodine (**20**) has remained elusive due to its high reactivity and inherently redox-sensitive functionalities, in particular 1,2-dihydropyridine and indole-2-acrylate.^18e,20^ Not unsurprisingly, nature’s way has inspired the approaches of many synthetic chemists over the years;^21^ in fact, more than half of the total and formal syntheses of catharanthine published to date have indeed relied on a Diels–Alder approach to the isoquinuclidine core.^21a−21n^ Interestingly, however, not one proceeded directly via dehydrosecodine. This is partly due to the difficulty of accessing the 5-ethyl-substituted 1,2-dihydropyridine motif (because of undesired regioselectivity in the reduction of pyridinium ions; see Scheme 1d), particularly in the presence of the sensitive/reactive indole-2-acrylate fragment.^20^

**Scheme 4:**
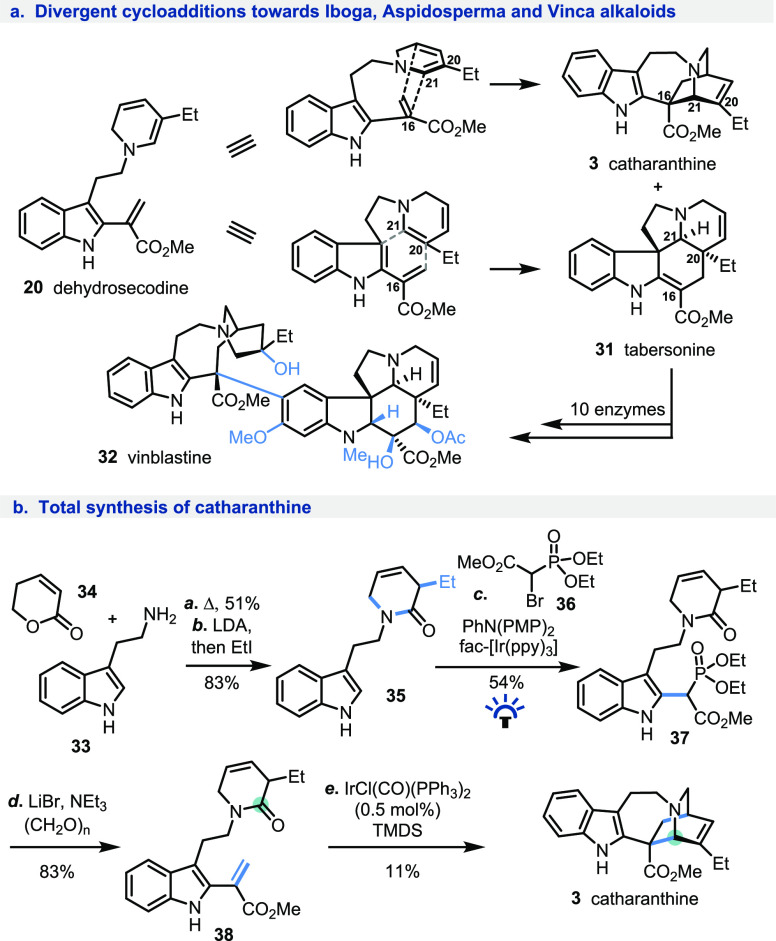
(a) Dehydrosecodine at the Center of the Monoterpene Indole Alkaloid Biosynthesis; (b) A New Total Synthesis of Catharanthine

Recognizing that our reductive strategy offers reliable regiocontrol in 1,2-dihydropyridine synthesis, as well as notable and well-documented chemoselectivity for the reduction of the lactam carbonyl over other functional groups, including alkenes, we set on a journey to access catharanthine (**3**) via its elusive biosynthetic precursor dehydrosecodine (**20**).

Our synthesis began with the formation of the α-substituted, β,γ-unsaturated δ-lactam **35** in a two-step sequence from commercially available starting materials **(**Scheme 4b). At high temperatures, tryptamine (**33**) and dihydropyrone (**34**) reacted to form the unsaturated lactam as a mixture of constitutional isomers in 51% yield.^22^ Subsequent double deprotonation of the mixture with 2 equiv of LDA and α-alkylation with ethyl iodide resulted in the formation of desired **35** in 83% yield. After extensive investigations (see the Supporting Information), and taking inspiration from Stephenson’s photoredox-catalyzed C2-functionalization of unprotected indoles,^23^ we were able to introduce a phosphonoester group at the C2 position of indole **35**, resulting in isolation of **37** in 54% yield. The phosphonoester **37** could in turn be used to install the terminal methylene group of **38** via the Rathke modification of the Horner–Wadsworth–Emmons reaction by using paraformaldehyde, in 83% yield.^24,25^

Having established a four-step route to the precursor of dehydrosecodine **20**, the stage was set for the final reductive [4 + 2] cycloaddition sequence. Pleasingly, upon submission of **38** to the newly developed reaction conditions, catharanthine (**3**) was indeed produced, albeit in trace amounts as determined by ^1^H NMR analysis of the crude reaction mixture. Extensive optimization of the reductive activation step led to an improved isolated yield (11%) of **3** when TMDS was slowly added to a solution of precursor **38** and Vaska’s complex, thus completing the fully biomimetic total synthesis of the alkaloid and establishing the intermediacy of its evasive and intriguing biosynthetic precursor, dehydrosecodine.

Efforts to isolate byproducts in the final reaction, to understand the low mass return, were unfruitful. Consequently, the reaction was performed in deuterated solvent in an NMR tube, in the hope of observing transient species.^26^ Upon slow addition of TMDS to a solution of **38** and Vaska’s complex in *d*_8_-toluene, catharanthine was immediately produced in 15% NMR yield, alongside reduced species **40** (85% NMR yield, as a mixture of isomers at the dihydropyridine), arising from the apparent hydridic reduction of the indole-2-acrylate in dehydrosecodine (**20**) (Scheme 5).^27^ Attempted purification via flash column chromatography on silica gel failed to provide **40**,^28^ while **3** could be isolated in 11% yield. Interestingly, no reaction product arising from the other intramolecular Diels–Alder (IMDA) pathway (see **31**, Scheme 4) was observed in any of these experiments.

**Scheme 5:**
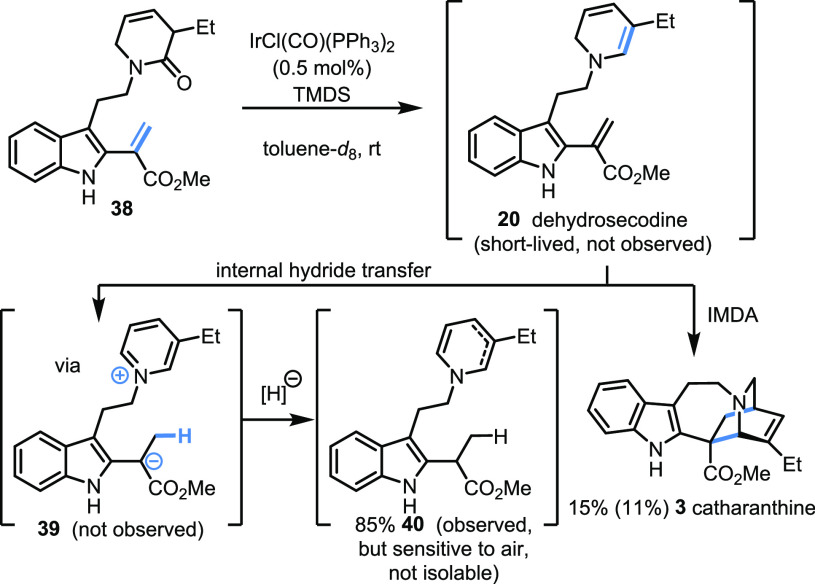
NMR Studies Uncover a Reactive and Short-Lived Species

Further efforts to improve reaction efficiency by introducing hydride scavengers did not change the ratio between catharanthine and the undesired rearranged product, suggesting an intramolecular hydride transfer, followed by protonation and hydridic reduction of the resulting pyridinium species **39** to give **40**.^29^ Although not completely unprecedented,^30^ this dihydropyridine-triggered hydride reduction of the pendant indole-2-acrylate suggests that any chemical synthesis of dehydrosecodine will likely always suffer from this undesired internal redox adjustment outside of the exquisitely controlled environment offered by nature’s optimized enzymatic pathways.

In conclusion, an iridium(I)-catalyzed reductive activation of β,γ-unsaturated δ-lactams and amides allows efficient and controlled access to cyclic and acyclic dienamines, delivering—after [4 + 2] cycloaddition—a range of bridged bicyclic and cyclohexene-substituted amine products. This robust approach proceeds with high stereocontrol, low catalyst loading, from readily available starting materials, and has enabled a short and protecting group-free total synthesis of catharanthine via its biosynthetic precursor, dehydrosecodine. Further work to uncover new reactivity of common functional groups through reductive activation approaches is ongoing in our laboratory, and the results will be disclosed in due course.

## Supporting Information Available

Experimental procedures and characterization data (PDF)
