# Supplementary material for: A General Iridium-Catalyzed Reductive Dienamine Synthesis Allows a Five-Step Synthesis of Catharanthine via the Elusive Dehydrosecodine
Source: J Am Chem Soc. 2021 Jul 13;143(29):10828–35. doi: 10.1021/jacs.1c04980 (PMC8397322; doi:10.1021/jacs.1c04980)
Supplement: Supplementary file 1 — ja1c04980_si_001.pdf [file ja1c04980_si_001.pdf]

Supporting information

For

A General Iridium-Catalyzed Reductive Dienamine Synthesis Allows a Five-Step Synthesis of Catharanthine *via* the Elusive Dehydrosecodine

Pablo Gabriel,[⊥ ǂ] Yaseen A. Almehmadi,[⊥ ǂ‡] Zeng Rong Wong,[ǂ] Darren J. Dixon[ǂ *]

[ǂ] Department of Chemistry, University of Oxford, Chemistry Research Laboratory, 12 Mansfield Road, Oxford, OX1 3TA, UK
[‡] Department of Chemistry, Rabigh College of Science and Arts, King Abdulaziz University, Jeddah 21589, Saudi Arabia

Corresponding author email: darren.dixon@chem.ox.ac.uk
[⊥] These authors have contributed equally.

Table of Contents

1. General information 4

2. Optimisation tables for the indole C2-functionalisation 5

3. Optimisation for the key step 7

4. NMR studies on the key step 8

5. General procedures 13

General Procedure A 13

General procedure B 13

General procedure C 14

General procedure D 14

General procedure E 14

6. Synthesis and characterization of starting materials 15

Previously reported substrates and coupling partners 15

5,6-Dihydro-2-pyrone (**34**) [6] 15

1-Benzyl-5,6-dihydropyridin-2(1H)-one (**S1**) 16

1-Benzyl-3,6-dihydropyridin-2(1*H*)-one (**15a**) 16

1,3-Dibenzyl-3,6-dihydropyridin-2(1*H*)-one (**15b**) 17

1-Benzyl-3-methyl-3,6-dihydropyridin-2(1H)-one (**15d**) 17

N-Benzyl-*N*-methylbut-3-enamide (**22a**) [4] 18

1-(Piperidin-1-yl)but-3-en-1-one (**22b**) [8] 18

N-(2-(1*H*-Indol-3-yl)ethyl)-*N*-benzylbut-3-enamide (**S2**) 19

N-Benzyl-*N*-(2-(1-tosyl-1*H*-indol-3-yl)ethyl)but-3-enamide (**22c**) 20

2-(1*H*-Indol-3-yl)-1-morpholinoethan-1-one (**25a**) 21

1-Morpholino-2-(1-tosyl-1*H*-indol-3-yl)ethan-1-one (**25b**) 22

N^1,N^4-Dibenzyl-N^1,N^4-dimethylsuccinamide (**28**) 23

7. NMR Study (figure 1) 24

1-Benzyl-1,2-dihydropyridine (**18a**) 24

8. Synthesis and characterization of [4+2] cycloaddition products 24

9-Benzyl-3a,4,7,7a-tetrahydro-1H-4,7-(epiminomethano)isoindole-1,3(2H)-dione (**19a**) 24

5,9-Dibenzyl-2-phenyl-3a,4,7,7a-tetrahydro-1*H*-4,7-(epiminomethano)isoindole-1,3(2*H*)-dione (**19b**) 25

2-Phenyl-3a,6,7,8,9,9a,10,10a-octahydro-4,10-ethenopyrrolo[3,4-*b*]quinolizine-1,3(2*H*,4*H*)-dione (**19c**) 26

9-Benzyl-5-methyl-2-phenyl-3a,4,7,7a-tetrahydro-1*H*-4,7-(epiminomethano)isoindole-1,3(2*H*)-dione (**19d**) 27

9-Benzyl-7-methyl-2,8-diphenyl-3a,4,7,7a-tetrahydro-1*H*-4,7-(epiminomethano)isoindole-1,3(2*H*)-dione (**19e**) 28

9-Butyl-8-(4-fluorophenyl)-2,6-diphenyl-3a,4,7,7a-tetrahydro-1*H*-4,7-(epiminomethano)isoindole-1,3(2*H*)-dione (**19f**) 29

9-Cyclopropyl-8-(2-methoxyphenyl)-2,6-diphenyl-3a,4,7,7a-tetrahydro-1*H*-4,7-(epiminomethano)isoindole-1,3(2*H*)-dione (**19g**) 31

9-Cyclopropyl-8-(2-methoxyphenyl)-6-phenyl-3a,4,7,7a-tetrahydro-1*H*-4,7-(epiminomethano)isoindole-1,3(2*H*)-dione (**19h**) 32

2-Cyclopropyl-3-(2-methoxyphenyl)-8-phenyl-2-azabicyclo[2.2.2]oct-7-ene-6-carbonyl)oxazolidin-2-one (**19i**). 33

Methyl 2-cyclopropyl-3-(2-methoxyphenyl)-8-phenyl-2-azabicyclo[2.2.2]oct-7-ene-6-carboxylate (**19j**) 34

Dimethyl 2-cyclopropyl-3-(2-methoxyphenyl)-8-phenyl-2-azabicyclo[2.2.2]oct-7-ene-5,6-dicarboxylate (**19k**) .. 35

2-Cyclopropyl-3-(2-methoxyphenyl)-8-phenyl-2-azabicyclo[2.2.2]oct-7-ene-6-carbonitrile (**19l**) 36

4-(Benzyl(methyl)amino)-2-phenyl-3a,4,7,7a-tetrahydro-1*H*-isoindole-1,3(2*H*)-dione (**24a**) 37

2-Phenyl-4-(piperidin-1-yl)-3a,4,7,7a-tetrahydro-1*H*-isoindole-1,3(2*H*)-dione (**24b**) 38

4-(Benzyl(2-(1-tosyl-1*H*-indol-3-yl)ethyl)amino)-2-phenyl-3a,4,7,7a-tetrahydro-1*H*-isoindole-1,3(2*H*)-dione (**24c**) 39

3-(2-(Benzyl(methyl)amino)cyclohex-3-ene-1-carbonyl)oxazolidin-2-one (**24d**) 40

4-(benzyl(methyl)amino)-3a,4,7,7a-tetrahydro-1H-isoindole-1,3(2H)-dione (**24e**) 41

4-(benzyl(methyl)amino)-2-methyl-3a,4,7,7a-tetrahydro-1H-isoindole-1,3(2H)-dione (**24f**) 42

4-Morpholino-2-phenylpyrrolo[3,4-*a*]carbazole-1,3(2*H*,10*H*)-dione (**27a**) 43

4-Morpholino-2-phenyl-10-tosylpyrrolo[3,4-*a*]carbazole-1,3(2*H*,10*H*)-dione (**27b**) 44

4,7-Bis(benzyl(methyl)amino)-2-phenyl-3a,4,7,7a-tetrahydro-1*H*-isoindole-1,3(2*H*)-dione (**30**) 45

9. Total Synthesis of catharanthine **46**

1-(2-(1H-indol-3-yl)ethyl)-3,6-Dihydropyridin-2(1H)-one (**S3**) and 1-(2-(1H-indol-3-yl)ethyl)-5,6-dihydropyridin-2(1H)-one (**S3'**) 46

1-(2-(1H-indol-3-yl)ethyl)-3-Ethyl-3,6-dihydropyridin-2(1H)-one (**35**) 47

Methyl 2-bromo-2-(diethoxyphosphoryl)acetate (**36**)[9] 48

Methyl 2-(diethoxyphosphoryl)-2-(3-(2-(3-ethyl-2-oxo-3,6-dihydropyridin-1(2H)-yl)ethyl)-1H-indol-2-yl)acetate (**37**) 49

Methyl 2-(diethoxyphosphoryl)-2-(3-(2-(3-ethyl-2-oxopyridin-1(2H)-yl)ethyl)-1H-indol-2- 51

yl)acetate (**37'**) 51

Methyl 2-(3-(2-(3-ethyl-2-oxo-3,6-dihydropyridin-1(2H)-yl)ethyl)-1H-indol-2-yl)acrylate (**38**) 53

Catharanthine (**3**) [10] 54

10. NMR Data **56**

11. References: **94**

1. General information

All reactions were preformed using purchased from Sigma-Aldrich, Acros Organics, Alfa Aesar, STREM or Fluorochem without further purification unless otherwise stated. All water was purified through a Merck Millipore reverse osmosis purification system prior to use. Anhydrous toluene was obtained from Acros Organics. Tetrahydrofuran, dichloromethane, and diethyl ether were dried by filtration through activated alumina (powder ~150 mesh, pore size 58 Å, basic, Sigma-Aldrich) columns and stored under an atmosphere of N_2 prior to use. Anhydrous toluene and dimethyl sulfoxide were used as supplied from Acros Organics (99.7+%, Extra Dry over Molecular Sieve, AcroSeal®) and were sparged with N_2 prior to use. Dichloromethane was used as supplied. Deuterated solvents were used as supplied. Reactions were performed in under a balloon of N_2, if not stated. Temperatures quoted are external. Solvents were removed under reduced pressure using Büchi Rotavapor apparatus.

NMR Spectra were measured on 400 MHz (^{1}H NMR at 400 MHz, ^{13}C NMR at 101 MHz, and ^{19}F NMR at 376 MHz) or Bruker 500 MHz (^{1}H NMR at 500 MHz, ^{13}C NMR at 126 MHz). Chemical shift for ^{1}H NMR and ^{13}C were referenced based on the used deuterated solvent at: ^{1}H, 7.26 ppm, ^{13}C 77.16 ($CDCl_3$), ^{1}H, 2.08 ppm, ^{13}C 137.5 (*d*$_8$-toluene), ^{1}H, 7.16 ppm, ^{13}C 128.1 (C_6D_6). NMR data are presented in the following format: chemical shift (δ) (multiplicity [app = apparent, br = broad, d = doublet, t = triplet, q = quartet, dd = doublet of doublets, dt = doublet of triplets, dq = doublet of quartets, ddt = Doublet of Doublet of Triplets, ddd = doublet of doublet of doublets, m = multiplet], coupling constant [in Hz], number of equivalent nuclei by integration, assignment). The numbering of the compounds for assignment was made based on a synthetic point of view and do not follow the IUPAC nomenclature.

High-resolution mass spectra (ESI) were recorded on Bruker μTOF mass spectrometer. Infrared spectra were recorded on a Bruker Tensor 27 FT-IR spectrometer as a thin film. Only selected maximum absorbances are reported (in *v*max (cm^{-1})). Melting points were obtained on a Leica Galen III Hot-stage melting point apparatus and microscope and on a Kofler hot block and are reported uncorrected. Analytical thin-layer chromatography (TLC) was performed on Merck silica gel 60 F254 plates and visualised with UV light (254), and/or a vanillin stain or a $KMnO_4$ solution. Silica gel column chromatography was performed using 60 Å silica gel 40-63 μm purchased from VWR

2. Optimisation tables for the indole C2-functionalisation

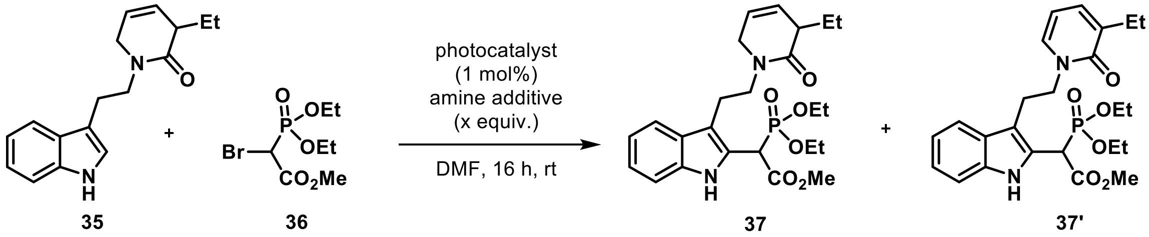

Entry	Photocatalyst	Equiv. phosphono-ester	Amine additive (equiv.)	Concentration	Conversion [i]	Selectivity [i] (ratio 37 : 37')	Comment
1	$Ru(bpy)_3Cl_2$	2.0	2.0	0.1 M	-*	1.4 : 1	
2	$Ru(bpy)_3Cl_2$	5.0	2.0	0.1 M	-*	-	only **37'** observed
3	$Ru(bpy)_3Cl_2$	1.5	2.0	0.1 M	-*	2.3 : 1	
4	$Ru(bpy)_3Cl_2$	2.0	2.0	0.1 M	-*	1.2 : 1	Argon atmosphere
5	$Ru(bpy)_3Cl_2$	2.0	2.0	0.1 M	-*	1.4 : 1	thoroughly degassed DMF
6	$Ru(bpy)_3Cl_2$	1.0	2.0	0.1 M	-*	1.4 : 1	
7	$Ru(bpy)_3Cl_2$	1.0	2.0	0.1 M	75%	4.8 : 1	-
8	*fac*-$Ir(ppy)_3$	1.0	2.0	0.1 M	76%	6.5 : 1	-
9	$[Ir(ppy)_2(dtbbpy)]PF_6$	1.0	2.0	0.1 M	79%	5.1 : 1	-
10	Eosin Y	1.0	2.0	0.1 M	0%	-	no reaction
11	$[Ir((dFCF_3)ppy)_2(dtbbpy)]PF_6$	1.0	2.0	0.1 M	79%	4.6 : 1	-
12	*fac*-$Ir(ppy)_3$	0.9	2.0	0.1 M	73%	8.1 : 1	-
8	*fac*-$Ir(ppy)_3$	1.0	2.0	0.1 M	76%	6.5 : 1	-
13	*fac*-$Ir(ppy)_3$	1.1	2.0	0.1 M	81%	4.8 : 1	-
14	*fac*-$Ir(ppy)_3$	1.5	2.0	0.1 M	92%	2.3 : 1	-
15	*fac*-$Ir(ppy)_3$	1.0	2.0	0.0125 M	40%	9.0 : 1	-
16	*fac*-$Ir(ppy)_3$	1.0	2.0	0.02 M	71%	10.8 : 1	-
17	*fac*-$Ir(ppy)_3$	1.0	2.0	0.05 M	74%	5.7 : 1	-
8	*fac*-$Ir(ppy)_3$	1.0	2.0	0.1 M	76%	6.5 : 1	-
19	*fac*-$Ir(ppy)_3$	1.0	2.0	0.2 M	81%	5.2 : 1	-
12	*fac*-$Ir(ppy)_3$	0.9	2.0	0.1 M	73%	8.1 : 1	-
20	*fac*-$Ir(ppy)_3$	0.9	1.0	0.2 M	74%	6.8 : 1	-
21	*fac*-$Ir(ppy)_3$	0.9	1.0 (DIPEA)	0.2 M	3%	-	-
22	*fac*-$Ir(ppy)_3$	1.0	0.2	0.2 M	86%	13.3 : 1	-
23	*fac*-$Ir(ppy)_3$	0.9	-	0.2 M	93%	3.4 : 1	-

Reagents and conditions: (a) Photocatalyst, 1 mol%, **35**, phosphonoester **36**, amine additive ($PhN(PMP)_2$ unless stated otherwise), photoreactor, DMF, N_2 atmosphere, rt, 14 h.

[i] Based on crude NMR ratios between **35, 37,** and **37'**. * Not measured.

Entries 1-6 were performed with a "loop" LED set-up, see data for **37'** for more information.

Entries 7-23 were performed with a photoreactor, see data for **37** for more details. Entries 8 and 12 repeated in the table for clarity.

The main issue in this reaction is the imperfect selectivity for the desired product **37**. **37'** is produced as an undesired side-product, and is not a simple oxygen-mediated oxidation of **37** (see entries 4 and 5), but rather by HAT at the lactam α-position of **36** and subsequent oxidation.

37 and **37'** are completely inseparable by FCC, pure samples of **37'** were obtained by reverse-phase preparative HPLC separation. Obtaining perfect or near-perfect selectivity is thus necessary.

Although entry 22 could be repeated on 0.1 mmol scale, it was not scalable (see next table, entry 1).

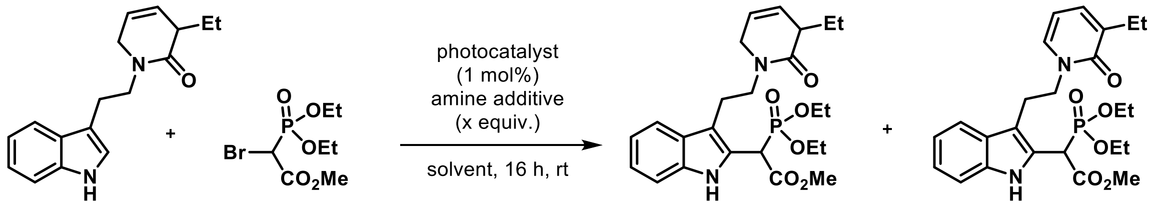

35 36 37 37'

Entry	Solvent	Photocatalyst	Equivalents phosphonate 36	Equivalents amine	Conversion [i]	Selectivity [i] (ratio 37 : 37')	Isolated yield
1 [ii]	DMF	*fac*-Ir(ppy)$_3$	0.9	0.2	75%	5.0 : 1	51%
2	PhMe	*fac*-Ir(ppy)$_3$	0.9	0.2	20%	6.5 : 1	-
3	EtOH	*fac*-Ir(ppy)$_3$	0.9	0.2	84%	>20 : 1	39%
4	MeCN	*fac*-Ir(ppy)$_3$	0.9	0.2	78%	31 : 1	-
5	DMSO	*fac*-Ir(ppy)$_3$	0.9	0.2	>90%	2.0 : 1	-
6	DCE	*fac*-Ir(ppy)$_3$	0.9	0.2	>90%	>20 : 1	51%
7	1:1 DMF/DCE	*fac*-Ir(ppy)$_3$	0.9	0.2	71%	7.1 : 1	-
8	DCE	*fac*-Ir(ppy)$_3$	0.9	0.2	>90%	>20 : 1	51%
9	DCE	Ru(bpy)$_3$Cl$_2$	0.9	0.2	47%	3.0 : 1	-
10	DCE	Ru(bpy)$_3$(PF$_6$)$_2$	0.9	0.2	- [iii]	>20 : 1	-
11	DCE	*fac*-Ir(ppy)$_3$	1.05	0.2	>90%	>20 : 1	53%
12	DCE	*fac*-Ir(ppy)$_3$	1.2	0.2	>90%	>20 : 1	53%
13	DCE	*fac*-Ir(ppy)$_3$	1.05	-	0%	-	-
14 [iv]	DCE	*fac*-Ir(ppy)$_3$	1.05	0.2	>90%	>20 : 1	53%

Reagents and conditions: (a) Photocatalyst, 1 mol%, phosphonoester **36**, PhN(PMP)$_2$, photoreactor, solvent, rt, 14 h, 0.1 mmol scale.
[i] Based on crude NMR ratios between **35**, **37**, and **37'**. [ii] On 1 mmol scale.
[iii] Although presence of the indole SM could not be confirmed, about 40% of the phosphonoester coupling partner was present in the crude.[iv] On 0.3 mmol scale.

Further screening revealed DCE to be an optimal solvent. Although the mass balance was low considering conversion, the conditions in entry 11 were reproducible (see entry 14), and were suitable to provide appreciable quantities of pure **37**.

3. Optimisation for the key step

Additives such as $PhCO_2H$, AcOH, NEt_3, SiO_2, DABCO, TMSCl, PhCHO, molecular sieves, H_2O, MeOH, and solvents and solvent mixtures comprised of toluene, chloroform, and/or dichloromethane were used, in an attempt to change the protonation form, enhance electrophilicity of the acrylate, temporarily mask the reactive alkene, enhance nucleophilicity of the diene, or capture any hydridic species post amide activation. No significant improvement was observed.

Only slow addition of TMDS as a toluene solution to a toluene solution of **38** provided increased amount of catharanthine.

Once **40** was identified as the major product, hydride scavengers such as 4-bromobenzaldehyde, or norbornene, were introduced as additives, but did not change the outcome of the reaction. Methyl acrylate was also used, but only significantly decreased the rate of the reduction of the lactam carbonyl, without altering the ratios of **40** and catharanthine produced.

4. NMR studies on the key step

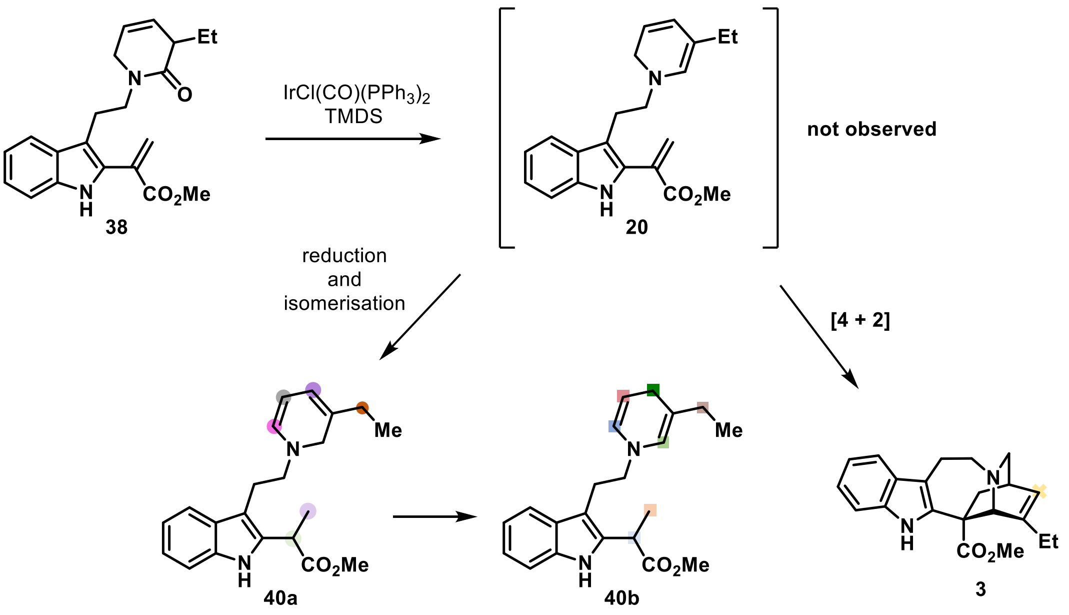

Time			
30 min	45%*	40%*	15%*
14 h	4%*	80%*	16%*
isolated	-	-	11%

Reagents and conditions: IrCl(CO)(PPh$_3$)$_2$, 1 mol%, TMDS added over 30 minutes as a solution in toluene-d_8, toluene-d_8, rt, 16 h.
* NMR yield obtained by ratio of **40a**, **40b**, and **3**.

The reaction was performed in an NMR tube. Spectra **A** and **B** in the following figure are of the reaction mixture (reaction in d_8-toluene). Filtration through a pad of silica, and NMR measurement of the filtrate in d_1-chloroform is shown as spectrum **C**. Purification by preparative TLC leads to isolation of catharanthine, whose spectrum is displayed for comparison ($CDCl_3$, spectrum **D**). **E**, **F**, and **G** are enlargements of the corresponding regions of the NMR spectra **A** and **B** (in d_8-toluene). Assignments are represented by colour shapes on the structures and respective NMR spectra. They were the results of detailed 2D NMR analysis (HSQC, HMBC, COSY) on this system, as well as on a model system without the pending C_2-acrylate.

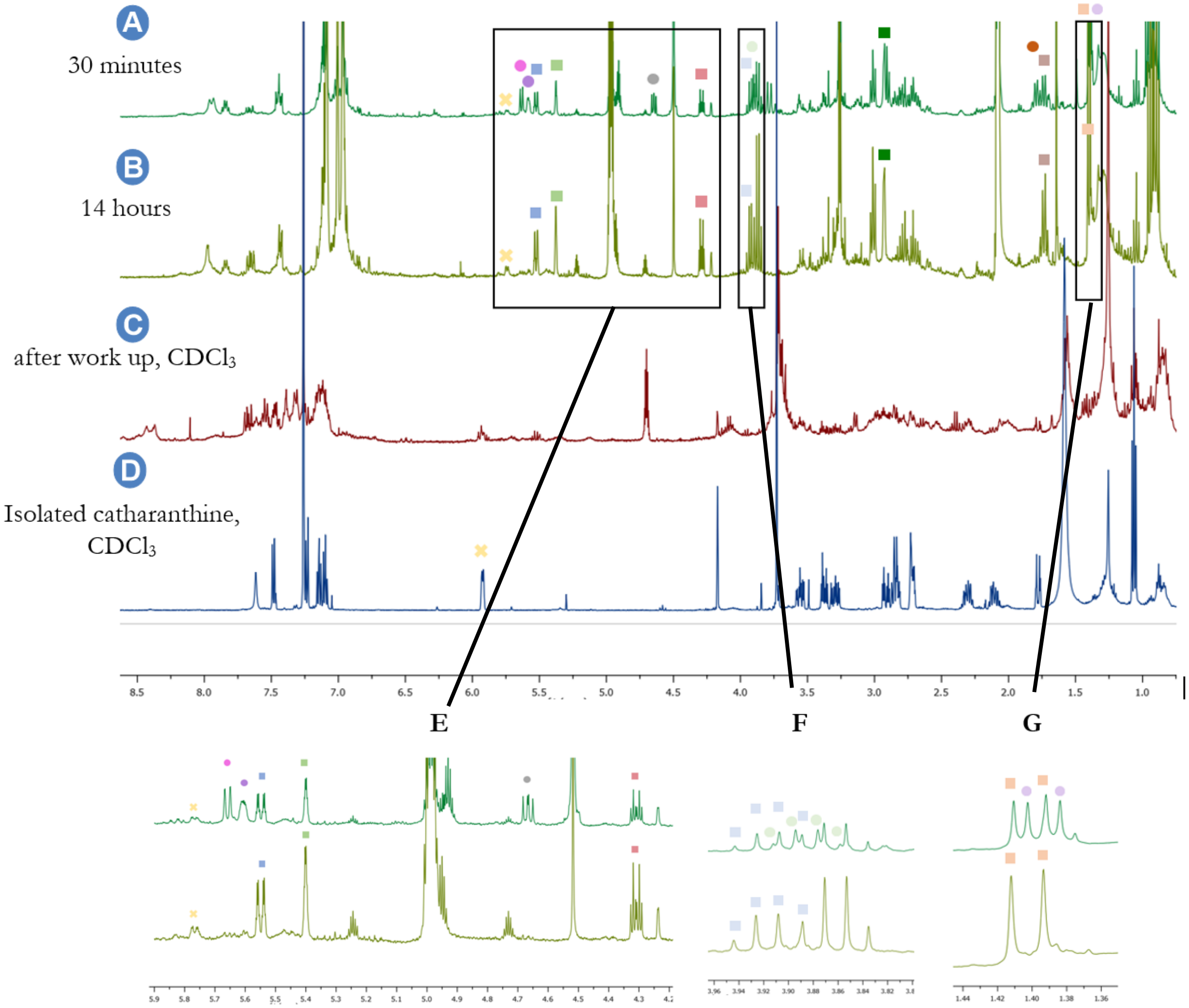

Early NMR measurements show that **40a** is the first observable intermediate, but is rapidly converted to **40b** by an isomerisation mechanism.

Interestingly, **40a** is not simply the reduced formed of dehydrosecodine **20**, due to the dihydropyridine moiety being a 1,2-dihydro-3-ethylpyridine compared to the 1,2-dihydro-5-ethylpyridine within dehydrosecodine **20**. This, combined with our fruitless efforts to find reaction conditions to increase the selectivity of the reaction for catharanthine **3** over non-productive species **40** (**a** and **b**) in the crude reaction mixture, led us to the conclusion that no hydridic species is responsible for the direct reduction of the acrylate moiety within dehydrosecodine **20**. It is rather an intramolecular hydride transfer from the 1,2-dihydro-5-ethylpyridine to the acrylate motif, giving rise to pyridinium species

39. This is also backed up by previous work by Scott *et al*, Marazano *et al*, and Wilson *et al*, see footnote 29 in the main text.

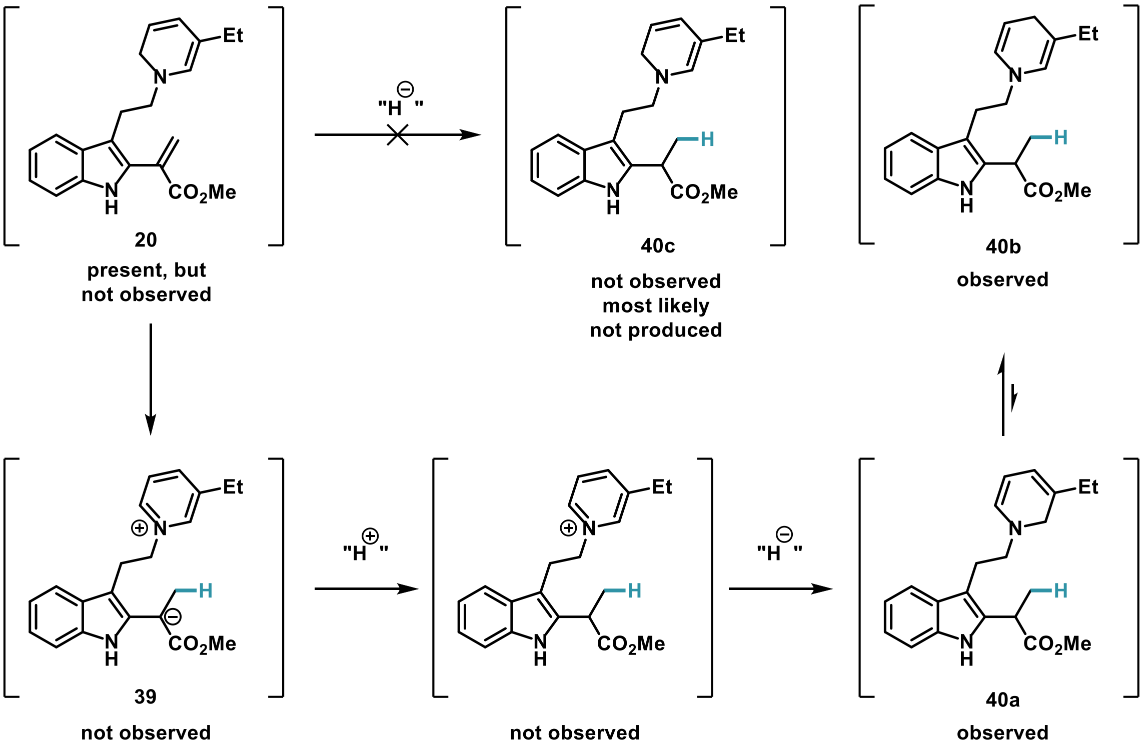

Zwitterion **39** is then protonated, and an hydridic species in the reaction media is then responsible for the reduction of the pyridinium moiety, giving rise to **40a** containing a 1,2-dihydro-3-ethyl moiety, following regioselectivity that is precedented (see main text, scheme 1, d1, and reference 7a-c). Further isomerisation then occurs over 14 h.

methyl 2-(3-(2-(3-ethylpyridin-1(2H)-yl)ethyl)-1H-indol-2-yl)propanoate (40a)

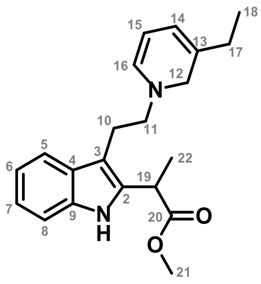

An NMR tube was charged with **38** (5.6 mg, 0.016 mmol), deuterated toluene (0.5 mL), and Vaska's complex (0.1 mg, 0.00016 mmol, 1 mol%). A solution of TMDS (8.5 μL, 0.048 mmol) in deuterated toluene (0.25 mL) was added dropwise over 20 min with the help of a syringe pump. Mild bubbling was observed. The reaction was monitored by NMR.

^{1}H NMR (C_7D_8, 400 MHz) δ_H [selected peaks]: 5.63 (app d, 1H, *J* = 7.9, 1.5 Hz, $C^{16}H$), 5.60–5.56 (m, 1H, $C^{14}H$), 4.65 (dd, 1H, *J* = 7.1, 5.6 Hz, $C^{15}H$), 3.89 (q, 1H, *J* = 7.2 Hz, $C^{19}H$), 1.80 (q, 1H, *J* = 7.7 Hz, $C^{19}H$), 1.39 (d, 3H, J = 7.3 Hz, $C^{22}H_3$).

methyl 2-(3-(2-(3-ethylpyridin-1(4H)-yl)ethyl)-1H-indol-2-yl)propanoate (40b)

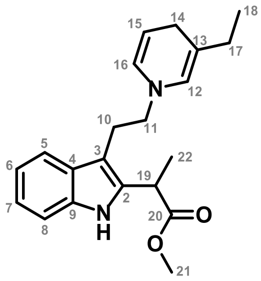

An NMR tube was charged with **38** (5.6 mg, 0.016 mmol), deuterated toluene (0.5 mL), and Vaska's complex (0.1 mg, 0.00016 mmol, 1 mol%). A solution of TMDS (8.5 μL, 0.048 mmol) in deuterated

toluene (0.25 mL) was added dropwise over 20 min with the help of a syringe pump. Mild bubbling was observed. The reaction was monitored by NMR.

^{1}H NMR (C_7D_8, 400 MHz) δ_H [selected peaks]: 5.53 (dq, 1H, J = 7.9, 1.5 Hz, $C^{16}H$), 5.38 (app sextet, 1H, $C^{12}H$), 4.29 (dt, 1H, J = 7.9, 3.2 Hz, $C^{15}H$), 3.93 (q, 1H, J = 7.3 Hz, $C^{19}H$), 3.01 (dd, 2H, J = 7.2, 6.4 Hz, $C^{11}H_2$), 2.94-2.91 (m, 2H, $C^{14}H_2$), 2.79 (dt, 1H, J = 14.5, 7.2 Hz, C^{10}Ha), 2.69 (dt, 1H, J = 14.3, 6.4 Hz, C^{10}Hb), 1.74 (app q, 2H, J = 7.7 Hz, $C^{17}H$), 1.40 (d, 3H, J = 7.3 Hz, $C^{22}H_3$), 0.94 (t, 3H, J = 7.1 Hz, $C^{18}H_3$).

Note: as this is the final intermediate before decomposition occurs, clean spectra can be obtained. Although it was not obtained in this particular NMR experiment, the following spectrum allows clear and unambiguous identification of the structure.

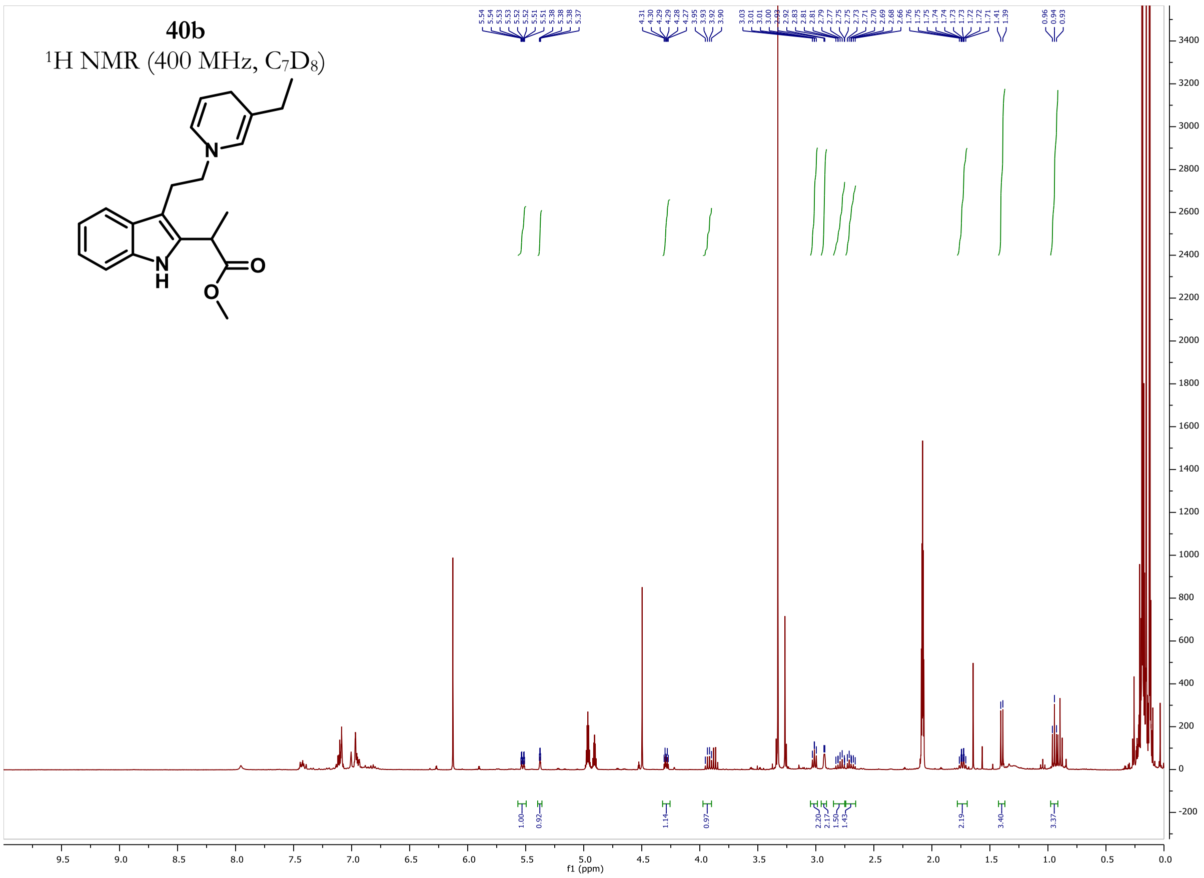

5. General procedures

General Procedure A for unsaturated δ-valerolactams synthesis.

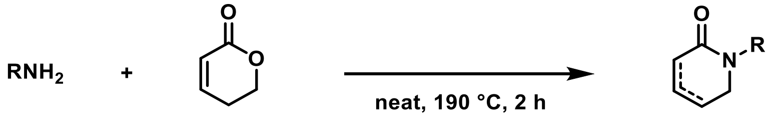

To a microwave vial was added the non-volatile amine (1.00 eq.) and dihydropyranone **34** (1.00 eq.). The vial was sealed and put under an Argon atmosphere with three vacuum-Argon cycles. It was then placed in a pre-heated oil bath, and behind a blast shield. The reaction was stirred at 190 °C for 3 h before being allowed to room temperature. The resulting gum was then dissolved in $CHCl_3$ and loaded on a silica gel column for FCC purification. The product is usually obtained as a mixture of isomers at the double-bond position.

Note: a pressure due to the generation of water can build up, the microwave vial should be checked to resist high pressure, and sized appropriately. Alternatively, the reaction vessel can also be connected to a nitrogen line equipped with a bubbler. It is important to keep oxygen out at those elevated temperatures. It may be necessary to wrap the oil bath with tin foil to allow the temperature to rise to 190 °C.

General procedure B for the synthesis of α-substituted β-γ unsaturated lactam

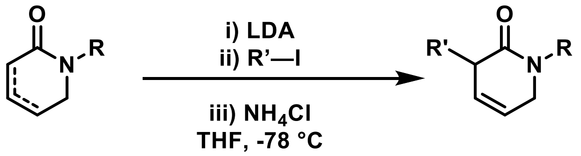

n-BuLi (2.5 M in hexanes, 1.5 eq.) was added dropwise to a solution of *i*-Pr_2NH (1.50 eq.) in THF (10 mL/mmol lactam) at −78 °C. After 30 min, a solution of lactam (1.00 eq.) in THF (1 mL/mmol lactam) was added dropwise. This mixture was allowed to stir at the same temperature for an hour before the electrophile (R'—I/Br) (1.1 eq.) was added. The reaction mixture was allowed to stir for an hour at the same temperature before being quenched with saturated NH_4Cl and allowed to warm to room temperature. The layers were separated, and the aqueous layer was extracted with CH_2Cl_2 (3 × 1.0 mL/mmol lactam). The combined organic layers were dried over Na_2SO_4 concentrated *in vacuo*. Purification *via* FCC afforded the corresponding pure β, γ–unsaturated δ-valerolactam.

General procedure C for the synthesis of amides for the corresponding carboxylic acid using HATU coupling

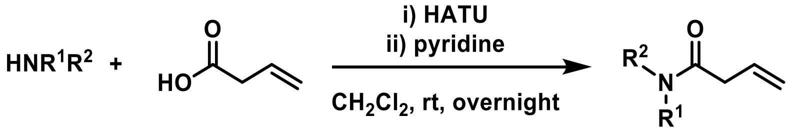

To a stirred solution of 3-butenoic acid (1 eq.) and secondary amine (1.1 eq.) in CH_2Cl_2 (0.2 M) was added HATU (1.1 eq.) at room temperature. After 5 min, pyridine (2 eq.) was added dropwise, and the mixture was allowed to stir overnight. This was followed by addition of saturated $NaHCO_3$. The resulting layers were separated, and the aqueous layer was extracted with CH_2Cl_2 (3 × 1.0 mL/mmol lactam). The combined organic phases were dried over Na_2SO_4, concentrated *in vacuo*, and purified by chromatography on silica gel to afford the corresponding amide.

General procedure D for the synthesis of amides for the corresponding carboxylic acid EDCI coupling

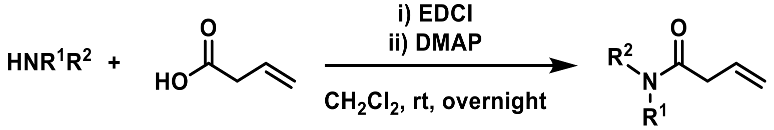

A solution of 3-butenoic acid (1 eq.) in CH_2Cl_2 (0.2 M) was added to secondary amine (1.1 eq.) and EDCI (1.1 eq.) and DMAP (0.1 eq.) at room temperature. The mixture was allowed to stir at the same temperature for 16 h, and this was followed by addition of water. The resulted layers were separated, and the aqueous layer was extracted with CH_2Cl_2 (3 × 10 mL). The combined organic phases were dried over Na_2SO_4, concentrated *in vacuo*, and purified by chromatography on silica gel to afford the corresponding amide.

General procedure E for [4+2] cycloaddition of dienamines with dienophiles

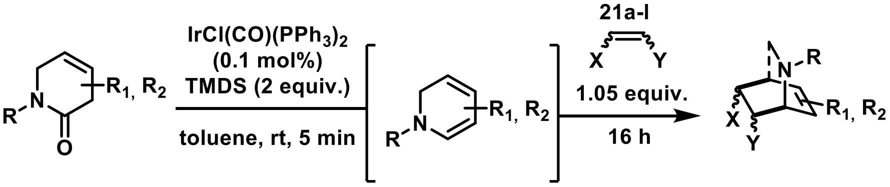

To a stirred solution of the relevant amide/lactam (0.1 mmol) and Vaska's complex (0.1 mol%) under nitrogen atmosphere in dry toluene (0.1 M) was added TMDS (0.2 mmol, 2 eq.) at room temperature. This resulted in a bubbling solution, which was left to stir for 5 min, 25 min, or 3 h, before adding the

dienophile (0.105 mmol, 1.05 eq.). This mixture was then stirred overnight, concentrated *in vacuo*, and purified by chromatography on silica gel or recrystallization in ether to afford the corresponding [4+2] cycloadduct.

6. Synthesis and characterization of starting materials

Previously reported substrates and coupling partners

15c,[1] **15e**,[2] **15f**,[3] **15g**,[3] oxazolidinone **21i**,[4] and **22a**[5] are all known literature compounds and were synthesised via these published routes.

5,6-Dihydro-2-pyrone (34) [6]

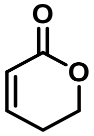

34 could be purchased from Sigma Aldrich. Alternatively, it can also be made from the following procedure, adapted from the literature.[6]

A mixture of paraformaldehyde (3 g, 100 mmol), 3-butenoic acid (8.6 g, 100 mmol, 1 eq.) and sulfuric acid (0.4 mL, 7.4 mmol) in acetic acid (25 mL, 4 M) was refluxed for 6.5 h. After cooling down to rt, anhydrous sodium acetate was added to the mixture, and the solvent was then removed. The pH of the crude reaction mixture was adjusted to 7 with sodium carbonate, extracted with CH_2Cl_2 (100 mL x 3). The organic layers were washed with water (15 mL), dried over $MgSO_4$, concentrated and purified by distillation to afford the corresponding α-β unsaturated lactone (5.3 g, 51 mmol, 51%).

^{1}H NMR ($CDCl_3$, 500 MHz) δ_H: 6.98–6.89 (m, 1H), 6.03 (dt, *J* = 9.8, 0.6 Hz, 1H), 4.45–4.38 (m, 2H), 2.50–2.40 (m, 2H).
NMR spectra and physical properties matched those reported in literature.[6]

1-Benzyl-5,6-dihydropyridin-2(1H)-one (S1)

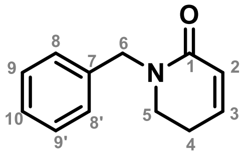

S1 +

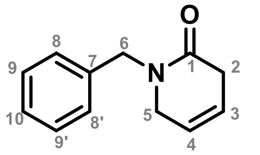

15a

Prepared according to **General Procedure A** from benzylamine and dihydropyranone (**34**). Purification *via* FCC (1 : 1 pentane/EtOAc) gave isomeric **S1** and **15a** as a 1 : 1 mixture and as a yellow oil (4.49 g, 24.0 mmol, 40%).

Selected data for **S1** obtained from the mixture of **S1** and **15a**.

^{1}H NMR ($CDCl_3$, 500 MHz) δ_H [selected peaks]: 6.55 (dt, 1H, *J* = 10.0 Hz, 4.2 Hz, C^{3}H), 6.00 (dt, 1H, *J* = 9.8, 1.8 Hz, C^{4}H), 4.62 (s, 2H, C^{6}H$_2$), 3.32 (t, 2H, *J* = 7.3 Hz, C^{5}H$_2$), 2.32 (tdd, 2H, *J* = 7.2, 4.2, 1.7 Hz, C^{2}H$_2$).

^{13}C NMR ($CDCl_3$, 126 MHz) δ_C: 164.7 (C^1), 139.5, (C^3), 137.6 (C^7), 128.7 (C^9, C$^{9'}$), 128.1 (C^8, C$^{8'}$), 127.5 (C^{10}), 125.5 (C^2), 49.8 (C^6), 44.7 (C^5), 24.3 (C^4).

1-Benzyl-3,6-dihydropyridin-2(1*H*)-one (15a)

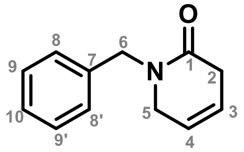

Prepared according to **General procedure B** from **S1** without adding an electrophile**.** Purification *via* FCC (1 : 1 pentane/EtOAc) gave **15a** as a yellow oil (853 mg, 4.56 mmol, 28%).

^{1}H NMR ($CDCl_3$, 500 MHz) δ_H: 7.36–7.24 (m, 5H, C^{8}H, C$^{8'}$H, C^{9}H, C$^{9'}$H, C^{10}H), 5.76 (dtt, 1H, *J* = 10.6, 3.4, 2.0 Hz, C^{4}H), 5.67 (dtt, 1H, *J* = 10.1, 3.1, 1.9 Hz, C^{3}H), 4.67 (s, 2H, C^{6}H$_2$), 3.85–3.78 (m, 2H, C^{5}H$_2$), 3.07–3.02 (m, 2H, C^{2}H$_2$).

^{13}C NMR ($CDCl_3$, 126 MHz) δ_C: 167.3 (C^1), 136.8 (C^7), 128.8 (C^9, C$^{9'}$), 128.2 (C^8, C$^{8'}$), 127.6 (C^{10}), 122.7 (C^4), 120.9 (C^3), 49.8 (C^6), 48.4 (C^5), 32.3 (C^2).

NMR spectra and physical properties matched those reported in literature.[7]

1,3-Dibenzyl-3,6-dihydropyridin-2(1*H*)-one (15b)

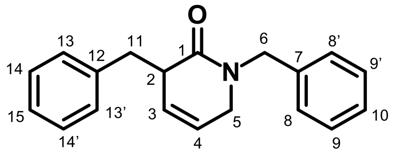

Prepared according to **General Procedure B** from **S1** and benzyl bromide. Purification *via* FCC (8 : 2 pentane/EtOAc) gave **15b** (194 mg, 699 µmol, 53%) as a colourless oil.

IR 3028, 2993, 2855, 1640 (C=O), 1493, 1453.

^{1}H NMR ($CDCl_3$, 500 MHz) δ_H: 7.31–7.26 (m, 3H, C^9H, $C^{9'}H$, $C^{10}H$), 7.26–7.20 (m, 3H, $C^{14}H$, $C^{14'}H$, $C^{15}H$), 7.20–7.16 (m, 2H, $C^{13}H$, $C^{13'}H$), 7.15–7.12 (m, 2H, C^8H, $C^{8'}H$), 5.71–5.56 (m, 2H, C^3H, C^4H), 4.66 (d, *J* = 14.7 Hz, 1H, C^6H), 4.53 (d, *J* = 14.6 Hz, 1H, $C^{6'}H$), 3.65–3.55 (m, 1H, C^5H), 3.40 (ddt, *J* = 17.2, 3.7, 2.0 Hz, 1H, $C^{5'}H$), 3.34 (dt, *J* = 7.5, 3.6 Hz, 1H, C^2H), 3.17 (dd, *J* = 13.2, 4.1 Hz, 1H, $C^{11}H$), 3.08 (dd, *J* = 13.2, 8.0 Hz, 1H, $C^{11'}H$).

^{13}C NMR ($CDCl_3$, 126 MHz) δ_C: 169.6 (C^1), 138.4 (C^{12}), 136.8 (C^7), 129.9 (C^{13}, $C^{13'}$), 128.7 (C^9, $C^{9'}$), 128.2 (C^8, $C^{8'}$), 128.2 (C^{14}, $C^{14'}$), 127.5 (C^{10}), 126.4 (C^3, C^{15}), 121.3 (C^4), 49.9 (C^6), 48.1 (C^5), 43.3 (C^2), 39.4 (C^{11}).

HRMS (ESI^+) exact mass calculated for $[M+H]^+$ ($C_{19}H_{20}ON$) requires **m/z** 278.1539, found **m/z** 278.1541.

1-Benzyl-3-methyl-3,6-dihydropyridin-2(1H)-one (15d)

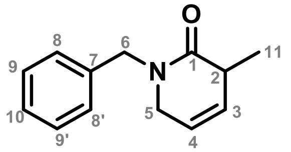

Prepared according to **General Procedure B** from **S1** and MeI. Purification *via* FCC (8 : 2 pentane/EtOAc) gave **15d** (217 mg, 78%) as a yellow oil.

IR 2929, 1642 (C=O), 1492, 1454, 1411, 1325, 1259.

^{1}H NMR ($CDCl_3$, 500 MHz) δ_H: 7.35–7.30 (m, 2H, C^9H, $C^{9'}H$), 7.29–7.25 (m, 3H, C^8H, $C^{8'}H$, $C^{10}H$), 5.73 (ddt, 1H, *J* = 10.1, 3.7, 2.0 Hz, C^3H), 5.66 (dtd, 1H, *J* = 10.0, 3.1, 1.7 Hz, C^4H), 4.67 (d, 1H, *J* = 18.7 Hz, C^6Ha), 4.65 (d, 1H, *J* = 18.7 Hz, C^6Hb), 3.82–3.78 (m, 2H, C^5H_2), 3.08–3.00 (m, 1H, C^2H), 1.36 (d, 3H, *J* = 7.4 Hz, $C^{11}H_3$).

^{13}C NMR ($CDCl_3$, 126 MHz) δ_C: 171.1 (C^{1}), 137.1 (C^{7}), 129.2 (C^{3}), 128.8 (C^{9}, $C^{9'}$), 128.2 (C^{8}, $C^{8'}$), 127.6 (C^{10}), 119.7 (C^{4}), 49.9 (C^{6}), 48.3 (C^{5}), 36.5 (C^{2}), 19.5 (C^{11}).

HRMS (ES^+) exact mass calculated for $[M+H]^+$ ($C_{13}H_{16}ON^+$) requires **m/z** 202.1226, found **m/z** 202.1229.

N-Benzyl-*N*-methylbut-3-enamide (22a) [4]

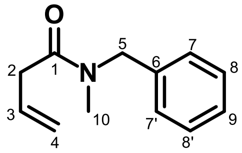

Prepared according to **General procedure D** from 3-butenoic acid and *N*-methylbenzylamie. Purification *via* FCC (8 : 2 pentane/EtOAc) gave amide **22a** as a colourless oil (1.6 g, 73%).

^{1}H NMR ($CDCl_3$, 400 MHz) δ_H: 7.45–7.23 (m, 4H, $C^{7}H$, $C^{7'}H$, $C^{8}H$, $C^{8'}H$), 7.17 (dd, *J* = 7.4, 1.4, 0.7 Hz, 1H, $C^{9}H$), 6.08–5.95 (m, 1H, $C^{3}H$), 5.27–5.04 (m, 2H, $C^{4}H$, $C^{4}H$), 4.57 (d, *J* = 22.4 Hz, 2H, $C^{5}H$, $C^{5'}H$), 3.20 (tt, *J* = 6.1, 1.5 Hz, 2H, $C^{2}H$, $C^{2'}H$), 2.93 (d, *J* = 9.9 Hz, 3H, $C^{10}H$).

NMR spectra and physical properties matched those reported in literature.[4]

1-(Piperidin-1-yl)but-3-en-1-one (22b) [8]

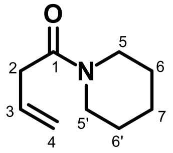

Prepared according to **General procedure D** from 3-butenoic acid and piperidine. Purification *via* FCC (8 : 2 pentane/EtOAc) gave amide **22b** as a colourless oil (1.40 g, 9.16 mmol, 92%).

^{1}H NMR ($CDCl_3$, 400 MHz) δ_H: 6.09–5.83 (m, 1H), 5.17–5.07 (m, 2H), 3.58–3.48 (m, 2H), 3.43–3.33 (m, 2H), 3.14 (dt, *J* = 6.5, 1.6 Hz, 2H), 1.62 (tq, *J* = 5.0, 2.2 Hz, 2H), 1.54 (dd, *J* = 7.0, 4.4 Hz, 4H).

^{13}C NMR ($CDCl_3$, 101 MHz) δ_C: 169.23, 131.97, 117.61, 47.01, 42.84, 39.02, 26.57, 25.65, 24.63.

NMR spectra and physical properties matched those reported in literature.[8]

N-(2-(1H-Indol-3-yl)ethyl)-N-benzylbut-3-enamide (S2)

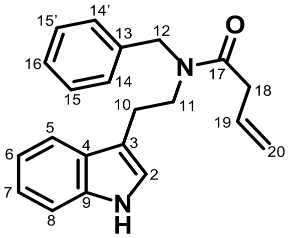

Prepared according to **General procedure C** from *N*-benzyltryptamine and 3-butenoic acid. Purification *via* FCC (9 : 1 CH_2Cl_2/MeOH) gave amide **S2** as a brown solid (2.50 g, 7.85 mmol, 82%). **S2** exists as a 52 (**A**) : 48 (**B**) mixture of rotamers in $CDCl_3$ at rt.

mp 82-83 °C

IR 3281 (broad NH), 3058, 2924, 1626 (C=O), 1452, 1340.

^{1}H NMR ($CDCl_3$, 400 MHz) δ_H: 8.22 (br s, 1H, NH (**A**)), 8.12 (br s, 1H, NH (**B**)), 7.59 (d, *J* = 7.9 Hz, 1H, C^2H (**B**)), 7.50 (d, *J* = 7.9 Hz, 1H, C^2H (**A**)), 7.39–7.25 (m, 10H, C^6H (**A**), C^6H (**B**), C^{14}H (**A**), C^{14}H (**B**), $C^{14'}$H (**A**), $C^{14'}$H (**B**), C^{15}H (**A**), C^{15}H (**B**), $C^{15'}$H (**A**) $C^{15'}$H (**B**)), 7.23–7.07 (m, 6H, C^7H (**A**), C^7H (**B**), C^8H (**A**), C^8H (**B**), C^{16}H (**A**), C^{16}H (**B**)), 6.99 (s, 1H, C^2H (**B**)), 6.92 (s, 1H, C^2H (**A**)), 6.09–5.89 (m, 2H, C^{19}H (**A**), C^{19}H (**B**)), 5.18 (dq, *J* = 10.0, 1.5 Hz, 1H, C^{20}H (**B**)), 5.15–5.07 (m, 2H, C^{20}H (**A**)), 4.98 (dq, *J* = 17.2, 1.6 Hz, 1H, $C^{20'}$H (**B**)), 4.67 (s, 2H, C^{12}H (**A**)), 4.44 (s, 2H, C^{12}H (**B**)), 3.71–3.65 (m, 2H, C^{11}H (**B**)), 3.53 (dd, *J* = 8.3, 6.5 Hz, 2H, C^{11}H (**A**)), 3.17 (dt, *J* = 6.6, 1.6 Hz, 2H, C^{18}H (**A**)), 3.08–3.01 (m, 4H, C^{10}H (**A**), C^{10}H (**B**)), 3.00 (t, *J* = 7.4 Hz, 2H, C^{18}H (**B**)).

^{13}C NMR ($CDCl_3$, 101 MHz) δ_C: 171.5 (C^{17} (**B**)), 171.5 (C^{17} (**A**)), 137.8 (C^{13} (**A**)), 137.0 (C^{13} (**B**)), 136.4 (C^9 (**A**)), 136.3 (C^9 (**B**)), 132.0 (C^{19} (**A**)), 131.9 (C^{19} (**B**)), 129.0 (C^{15} (**B**), $C^{15'}$ (**B**)), 128.7 (C^{15} (**A**), $C^{15'}$ (**A**)), 128.3 (C^{14} (**A**), $C^{14'}$ (**A**)), 127.7 (C^{16} (**A**)), 127.6 (C^4 (**A**)), 127.5 (C^{16} (**B**)), 127.1 (C^4 (**B**)), 126.5 (C^{14} (**B**), $C^{14'}$ (**B**)), 122.4 (C^2 (**A**)), 122.3 (C^2 (**B**), 122.1 (C^7 (**A**), C^{17} (**B**)), 119.7 (C^6 (**B**)), 119.5 (C^6 (**A**)), 119.0 (C^5 (**B**)), 118.3 (C^5 (**A**)), 118.0 (C^{20} (**A**)), 117.8 (C^{20} (**B**)), 113.3 (C^3 (**B**)), 112.2 (C^3 (**A**)), 111.6 (C^8 (**A**)), 111.2 (C^8 (**B**)), 52.0(C^{12} (**B**)), 5.4 (C^{12} (**A**)), 47.7 (C^{11} (**A**)), 47.6 (C^{11} (**B**)), 38.9 (C^{18} (**A**)), 38.4 (C^{18} (**B**)), 24.6 (C^{10} (**B**)), 23.6 (C^{10} (**A**)).

HRMS (ES^+) exact mass calculated for $[M+Na]^+$ ($C_{21}H_{23}ON_2$) requires **m/z** 319.8105, found **m/z** 319.1805.

N-Benzyl-N-(2-(1-tosyl-1H-indol-3-yl)ethyl)but-3-enamide (22c)

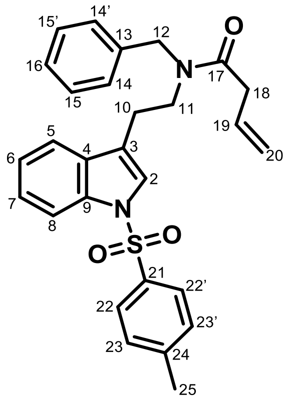

To a solution of indole **S2** (666 mg, 2.09 mmol), *p*-toluenesulfonyl chloride (479 mg, 2.51 mmol), and benzyltriethylammonium chloride (48 mg, 0.21 mmol) in CH_2Cl_2 (4 mL) was added powdered sodium hydroxide (150 mg, 3.76 mmol) at rt. The mixture was stirred for 24 h. This was followed by addition of water (4 mL). The resulted layers were separated, and the aqueous layer was extracted with CH_2Cl_2 (4 x 25 mL). The combined organic phases were washed twice with water, once with brine, dried over Na_2SO_4, concentrated *in vacuo*, and purification *via* FCC (40 : 1 CH_2Cl_2/Et_2O) gave tosylated indole **22c** (811 mg, 1.72 mmol, 82%) as a brown solid. **22c** exists as a 64 (**A**) : 36 (**B**) mixture of rotamers in $CDCl_3$ at rt.

mp 97-98 °C

IR 3064, 2923, 1645 (C=O), 1447, 1421, 1172.

^{1}H NMR ($CDCl_3$, 500 MHz) δ_H: 7.98 (t, J = 7.8 Hz, 2H, C^8H (**A**)), 7.76–7.70 (m, 3H, C^{23}H (**A**), C^{23}H (**B**)), 7.55–7.50 (m, 1H, C^5H (**A**)), 7.37–7.16 (m, 15H, C^5H (**B**), C^6H (**A**), C^6H (**B**), C^{14}H (**A**), $C^{14'}$H (**A**), C^{15}H (**A**), C^{15}H (**B**), $C^{15'}$H (**A**), $C^{15'}$H (**B**), C^{16}H (**A**), C^{16}H (**B**), C^{22}H (**A**), C^{22}H (**B**), $C^{22'}$H (**A**), $C^{22'}$H (**B**)), 7.08 (dd, J = 6.9, 1.8 Hz, 2H, C^{14}H (**B**), $C^{14'}$H (**B**)), 6.02 (ddt, J = 16.9, 10.1, 6.6 Hz, 1H, C^{19}H (**A**)), 5.91 (ddt, J = 16.9, 10.2, 6.6 Hz, 0.5H, C^{19}H (**B**)), 5.23–5.17 (m, 1H, C^{20}H (**B**)), 5.16–5.06 (m, 2H, C^{20}H (**A**)), 4.98 (dq, J = 17.2, 1.6 Hz, 0.6H, C^{20}H (**B**)), 4.58 (s, 1H, C^{12}H (**B**)), 4.36 (s, 2H, C^{12}H (**A**)), 3.63–3.57 (m, 2H, C^{11}H (**A**)), 3.53–3.47 (m, 1H, C^{11}H (**B**)), 3.16 (dt, J = 6.6, 1.5 Hz, 2H, C^{18}H (**A**)), 3.00 (dt, J = 6.6, 1.6 Hz, 1H, C^{11}H (**B**)), 2.96–2.89 (m, 2H, C^{10}H (**A**)), 2.91–2.85 (m, 1H, C^{10}H (**B**)), 2.32 (s, 4H, C^{25}H (**A**), C^{25}H (**B**)).

^{13}C NMR ($CDCl_3$, 126 MHz) δ_C: 171.6 (C^{17} (**A**)), 171.1 (C^{17} (**B**)), 145.1 (C^{21} (**B**)), 144.9 (C^{21} (**A**)), 137.6 (C^{13} (**B**)), 136.7 (C^{13} (**A**)), 135.5 (C^{24} (**A**)), 135.3 (C^{19} (**A**)), 135.3 (C^9 (**B**), C^{24} (**A**)), 131.7 (C^{19} (**B**)), 131.7 (C^{19} (**A**)), 130.9 (C^4 (**A**)), 130.4 (C^4 (**B**)), 130.1 (C^{23} (**B**), $C^{23'}$ (**B**)), 130.0 (C^{23} (**A**), $C^{23'}$ (**A**)), 129.1 (C^{15} (**A**),

C$^{15'}$ (**A**)), 128.8 (C^{15} (**B**), C$^{15'}$ (**B**)), 128.3 (C^{22} (**B**), C$^{22'}$ (**B**)), 127.9 (C^{2} (**A**)), 127.7 (C^{2} (**B**)), 126.9 (C^{22} (**A**), C^{22} (**A**), C^{14} (**B**), C^{14} (**B**)), 126.5 (C^{14} (**A**), C$^{14'}$ (**A**)), 125.2 (C^{7} (**B**)), 124.9 (C^{7} (**A**)), 123.7 (C^{6} (**B**)), 123.4 (C^{16} (**B**)), 123.4 (C^{6} (**A**)), 123.3 (C^{16} (**A**)), 120.2 (C^{3} (**A**)), 119.8 (C^{5} (**A**)), 119.0 (C^{3} (**B**)), 118.9 (C^{5} (**B**)), 118.2 (C^{20} (**A**)), 118.0 (C^{20} (**B**)), 114.1 (C^{8} (**B**)), 113.8 (C^{8} (**A**)), 52.3 (C^{12} (**A**)), 48.6 (C^{12} (**B**)), 47.0 (C^{10} (**A**)), 46.8 (C^{10} (**B**)), 38.9 (C^{18} (**A**)), 38.5 (C^{18} (**B**)), 24.6 (C^{10} (**B**)), 23.4 (C^{10} (**A**)), 21.7 (C^{25} (**A**), C^{25} (**B**)).
HRMS (ES^{+}) exact mass calculated for $[M+Na]^+$ ($C_{28}H_{29}O_3N_2{}^{32}S$) requires **m/z** 473.1893, found **m/z** 483.1891.

2-(1*H*-Indol-3-yl)-1-morpholinoethan-1-one (25a)

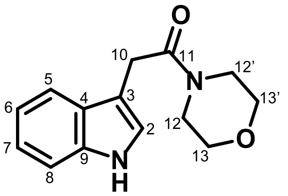

Prepared according to **General procedure D** from 3-indoleacetic acid and morpholine. Purification *via* FCC (20 : 1 CH_2Cl_2/MeOH) gave **25a** as a brown solid (1.40 g, 5.73 mmol, 66%).

mp 119-120 °C
IR 3282 (broad NH), 3057, 2964, 2857, 1627 (C=O), 1458, 1441.
^{1}H NMR ($CDCl_3$, 400 MHz) δ_H: 8.23 (br s, 1H, NH), 7.62 (dd, *J* = 7.9, 1.1 Hz, 1H, C^{5}H), 7.36 (dt, *J* = 8.2, 1.0 Hz, 1H, C^{6}H), 7.21 (ddd, *J* = 8.1, 7.0, 1.2 Hz, 1H, C^{7}H), 7.13 (td, *J* = 7.5, 1.0 Hz, 1H, C^{8}H), 7.09–7.07 (m, 1H, C^{2}H), 3.84 (d, *J* = 1.1 Hz, 2H, C^{10}H), 3.72–3.43 (m, 8H, C^{12}H, C$^{12'}$H, C^{13}H, C$^{13'}$H).
^{13}C NMR ($CDCl_3$, 101 MHz) $\delta_{C:}$ 170.3 (C^{11}), 136.3 (C^{9}), 127.1 (C^{4}), 122.5 (C^{7}), 122.4 (C^{2}), 119.9 (C^{8}), 118.8 (C^{5}), 111.4 (C^{6}), 109.3 (C^{3}), 67.0 (C^{13}), 66.7 (C$^{13'}$), 46.7 (C^{12}), 42.3 (C$^{12'}$), 31.3 (C^{10}).
HRMS (ES^{+}) exact mass calculated for $[M+Na]^+$ ($C_{14}H_{17}O_2N_2$) requires **m/z** 245.1284, found **m/z** 245.1286.

1-Morpholino-2-(1-tosyl-1*H*-indol-3-yl)ethan-1-one (25b)

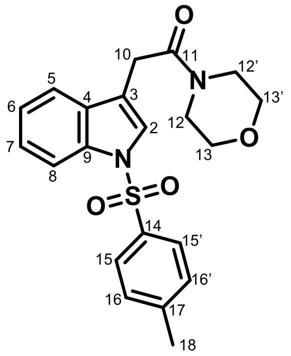

To a solution of indole **25a** (222 mg, 0.91 mmol), *p*-toluenesulfonyl chloride (208 mg, 1.09 mmol), and benzyltriethylammonium chloride (21 mg, 0.09 mmol) in CH_2Cl_2 (2 mL) was added powdered sodium hydroxide (66 mg, 1.64 mmol) at rt. The mixture was stirred for 24 h. This was followed by addition of water (2 mL). The resulted layers were separated, and the aqueous layer was extracted with CH_2Cl_2 (4 x 15 mL). The combined organic phases were washed twice with water, once with brine, dried over Na_2SO_4, concentrated *in vacuo*, and purified by recrystallization with 1 : 1 pentane/CH_2Cl_2 to afford the corresponding tosylated indole **25b** (217 mg, 544 μmol, 60%) as a slightly brown solid.

mp 128-129 °C

IR 3054, 2921, 2856, 1644 (C=O), 1364, 1173.

^{1}H NMR ($CDCl_3$, 400 MHz) δ_H: 7.99 (d, *J* = 8.3 Hz, 1H, C^8H), 7.78–7.71 (m, 2H, $C^{15}H$, $C^{15'}H$), 7.54 (br d, *J* = 7.9 Hz, 1H, C^5H), 7.46 (app s, 1H, C^2H), 7.34 (ddd, *J* = 8.4, 7.1, 1.2 Hz, 1H, C^7H), 7.25 (t, *J* = 7.5 Hz, 1H, C^6H), 7.21 (d, *J* = 8.1 Hz, 2H, $C^{16}H$, $C^{16'}H$), 3.74 (d, *J* = 1.2 Hz, 2H, $C^{10}H$), 3.64 (br s, 4H, $C^{12}H$, $C^{13}H$), 3.47–3.42 (m, 2H, $C^{13'}H$), 3.42–3.37 (m, 2H, $C^{12'}H$), 2.34 (s, 3H, $C^{18}H$).

^{13}C NMR ($CDCl_3$, 101 MHz) δ_C: 168.6 (C^{11}), 145.2 (C^{17}), 135.3 (C^9), 135.3 (C^{14}), 130.3 (C^4), 130.0 (C^{16}, $C^{16'}$), 126.9 (C^{15}, $C^{15'}$), 125.2 (C^7), 124.0 (C^2), 123.5 (C^6), 119.7 (C^5), 116.1 (C^3), 113.9 (C^8), 66.9 (C^{13}), 66.6 ($C^{13'}$), 46.7 (C^{12}), 42.3 ($C^{12'}$), 31.1 (C^{10}), 21.7 (C^{18}).

HRMS (ES^+) exact mass calculated for $[M+Na]^+$ ($C_{21}H_{23}O_5N_2{}^{32}S$) requires **m/z** 399.1373, found **m/z** 399.1369.

N^1,N^4-Dibenzyl-N^1,N^4-dimethylsuccinamide (28)

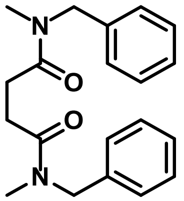

Prepared according to a modification of **General Procedure A**, using 2.0 equivalents of N-benzylmethylamine, and 1.0 equivalent of dimethylsuccinate. Purification *via* FCC (1 : 1 pentane/EtOAc) gave **28** as a yellow oil (520 mg, 1.60 mmol, 21%). **28** exists as a complex mixture of rotamers in $CDCl_3$ at rt.

mp 59-60 °C

IR 2922, 1639 (2 * C=O), 1494, 1402.

^{1}H NMR ($CDCl_3$, 500 MHz) δ_H: 7.43–7.14 (m, 10H), 4.71–4.54 (m, 4H), 3.05–2.88 (m, 6H), 2.87–2.67 (m, 4H).

^{13}C NMR ($CDCl_3$, 126 MHz) δ_C: 172.6, 172.6, 172.4, 172.4, 137.5, 136.8, 136.8, 129.0, 128.7, 128.0, 127.7, 127.4, 126.6, 53.4, 51.1, 51.1, 34.9, 34.9, 34.2, 34.2, 28.7, 28.7, 28.4, 28.4.

HRMS (ES^+) exact mass calculated for $[M+H]^+$ ($C_{20}H_{25}O_2N_2^+$) requires **m/z** 325.1911, found **m/z** 325.1912.

7. NMR Study (figure 1)

1-Benzyl-1,2-dihydropyridine (18a)

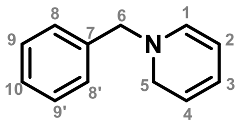

An NMR tube was charged with lactam **15a** (6.9 mg, 0.037 mmol), trimethoxybenzene as an internal standard, deuterated toluene (0.5 mL), TMDS (13 μL, 0.0111 mmol) and Vaska's complex (0.1 mg, 0.0004 mmol). The resultant reaction mixture was monitored by NMR spectroscopic analysis.
^{1}H NMR (C_7D_8, 400 MHz) δ_H [selected peaks]: 5.87–5.83 (m, 1H, C^3H), 5.82 (dt, 1H, J = 7.3, 1.0 Hz, C^1H), 4.93–4.87 (m, 1H, C^2H), 4.70 (ddd, 1H, J = 7.0, 5.4, 1.4 Hz, C^4H), 3.61 (dd, 1H, J = 3.9, 1.6 Hz, C^5H_2), 3.51 (d, 2H, J = 3.9, 1.6 Hz, C^6H_2).
^{13}C NMR (C_7D_8, 101 MHz) δ_C [selected peaks]: 138.8 (C^1), 111.7, (C^2), 95.6 (C^4), 59.3 (C^6), 48.0 (C^5).

8. Synthesis and characterization of [4+2] cycloaddition products

9-Benzyl-3a,4,7,7a-tetrahydro-1H-4,7-(epiminomethano)isoindole-1,3(2H)-dione (19a)

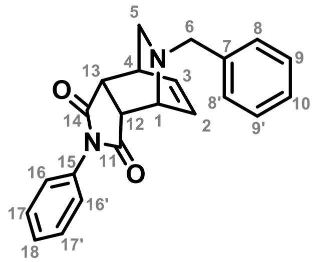

Prepared according to **General procedure E** from **15a** and *N*-phenyl maleimide. Purification *via* FCC (1 : 1 pentane/EtOAc) gave **19a** as amorphous yellow solid (11.9 mg, 34.6 μmol, 93%).
IR 1709 (2 * C=O), 1498, 1382, 1183.
^{1}H NMR ($CDCl_3$, 500 MHz) δ_H: 7.40–7.34 (m, 2H, $C^{16}H$, $C^{16'}H$), 7.33–7.24 (m, 5H, C^8H, $C^{8'}H$, C^9H, $C^{9'}H$, $C^{18}H$), 7.24–7.18 (m, 1H, $C^{10}H$), 7.12–7.07 (m, 2H, $C^{17}H$, $C^{17'}H$), 6.42 (ddd, 1H, J = 8.0, 6.3, 1.5 Hz, C^3H), 6.29 (ddd, 1H, J = 8.2, 5.2, 1.4 Hz, C^2H), 3.97 (ddd, 1H, J = 5.4, 4.0, 1.5 Hz, C^1H), 3.62 (d, 1H, J = 13.1 Hz, C^6Ha), 3.46-3.38 (C^6Hb, $C^{12}H$), 3.19–3.14 (m, 1H, C^4H), 3.04 (dd, 1H, J = 10.1, 2.1 Hz, C^5Ha), 3.00 (dd, 1H, J = 8.2, 3.2 Hz, $C^{13}H$), 2.03 (dd, 1H, J = 10.1, 2.6 Hz, C^5Hb).

^{13}**C NMR** ($CDCl_3$, 126 MHz) δ_C: 177.8 (C^{14}), 176.5 (C^{11}), 138.6 (C^{7}), 132.03 (C^{3}), 132.97 (C^{15}), 130.9 (C^{2}), 129.3 (C^{16}, $C^{16'}$), 128.9 (C^{8}, $C^{8'}$), 128.8 (C^{18}), 128.6 (C^{9}, $C^{9'}$), 127.4 (C^{10}), 126.6 (C^{17}, $C^{17'}$), 61.5 (C^{6}), 53.1 (C^{5}), 52.9 (C^{1}), 46.1 (C^{12}), 41.0 (C^{13}), 33.6 (C^{4}).

HRMS (ES^+) exact mass calculated for $[M+H]^+$ ($C_{22}H_{21}O_2N_2^+$) requires **m/z** 345.1598, found **m/z** 345.1593.

5,9-Dibenzyl-2-phenyl-3a,4,7,7a-tetrahydro-1*H*-4,7-(epiminomethano)isoindole-1,3(2*H*)-dione (19b)

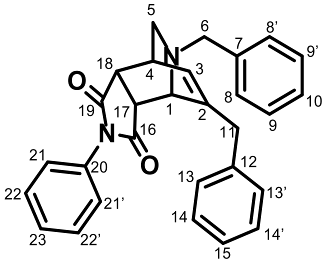

Prepared according to **General procedure E** from **15b** and *N*-phenyl maleimide. Purification *via* FCC (2 : 1 pentane/EtOAc) gave **19b** as a slightly yellow oil (38.7 mg, 89 μmol, 89%).

IR 3028, 2852, 1706 (C=O), 1497, 1379.

^{1}**H NMR** ($CDCl_3$, 400 MHz) δ_H: 7.43–7.34 (m, 2H, $C^{21}H$, $C^{21'}H$), 7.36–7.27 (m, 1H, $C^{23}H$), 7.27–7.13 (m, 6H, $C^{8}H$, $C^{8'}H$, $C^{9}H$, $C^{9'}H$, $C^{14}H$, $C^{14'}H$), 7.11–6.99 (m, 6H, $C^{10}H$, $C^{13}H$, $C^{13'}H$, $C^{15}H$, $C^{22}H$, $C^{22'}H$), 5.77 (dd, J = 6.0, 1.9 Hz, 1H, $C^{3}H$), 3.81 (dd, J = 4.2, 1.9 Hz, 1H, $C^{1}H$), 3.47 (dd, J = 8.1, 4.2 Hz, 1H, $C^{17}H$), 3.36 (dd, J = 15.7, 1.5 Hz, 1H, $C^{11}H$), 3.30–3.19 (m, 2H, $C^{6}H$, $C^{11'}H$), 3.13–3.04 (m, 2H, $C^{4}H$, $C^{6'}H$), 3.01–2.91 (m, 2H, $C^{5}H$, $C^{18}H$), 1.93 (dd, J = 9.9, 2.6 Hz, 1H, $C^{5'}H$).

^{13}**C NMR** ($CDCl_3$, 101 MHz) δ_C: 177.9 (C^{19}), 176.3 (C^{16}), 143.8 (C^{2}), 138.9 (C^{7}), 137.6 (C^{12}), 132.0 (C^{20}), 129.7 (C^{13}, $C^{13'}$), 129.3 (C^{8}, $C^{8'}$), 128.8 (C^{23}), 128.6 (C^{21}, $C^{21'}$), 128.6 (C^{14}, $C^{14'}$), 128.4 (C^{9}, $C^{9'}$), 127.2 (C^{10}), 126.7 (C^{15}), 126.6 (C^{22}, $C^{22'}$), 123.7 (C^{3}), 61.2 (C^{6}), 56.8 (C^{1}), 53.8 (C^{5}), 46.4 (C^{17}), 42.0 (C^{11}), 41.8 (C^{18}), 34.2 (C^{4}).

HRMS (ES^+) exact mass calculated for $[M+Na]^+$ ($C_{29}H_{27}O_2N_2$) requires **m/z** 425.2067, found **m/z** 425.2073.

2-Phenyl-3a,6,7,8,9,9a,10,10a-octahydro-4,10-ethenopyrrolo[3,4-*b*]quinolizine-1,3(2*H*,4*H*)-dione (19c)

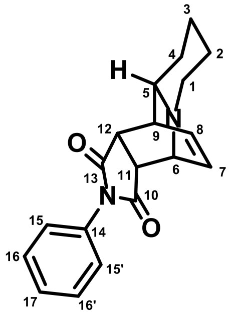

Prepared according to **General procedure E** from **15c** and *N*-phenyl maleimide. Purification *via* FCC (20 : 1 CH_2Cl_2/MeOH) gave **19c** as a colourless oil (9.3 mg, 30.2 µmol, 30%).

IR 2935, 1707 (2 * C=O), 1598, 1498, 1383.

^{1}H NMR ($CDCl_3$, 500 MHz) δ_H: 7.47–7.40 (m, 2H, $C^{16'}H$, $C^{16'}H$), 7.39–7.33 (m, 1H, $C^{17}H$), 7.18–7.13 (m, 2H, $C^{15}H$, $C^{15'}H$), 6.56 (ddd, *J* = 8.4, 5.4, 1.2 Hz, 1H, C^7H), 6.32–6.26 (m, 1H, C^8H), 3.93–3.86 (m, 1H, C^6H), 3.51–3.44 (m, 1H, $C^{11}H$), 3.19–3.11 (m, 2H, C^9H, $C^{12}H$), 2.98–2.90 (m, 1H, C^5H), 2.80 (td, *J* = 10.3, 4.2 Hz, 1H, C^1H), 2.74–2.67 (m, 1H, $C^{1'}H$), 1.80–1.68 (m, 2H, $C^{2'}H$, $C^{3'}H$), 1.68–1.57 (m, 1H, C^2H), 1.56–1.45 (m, 1H, C^3H), 1.44–1.38 (m, 2H, C^4H).

^{13}C NMR ($CDCl_3$, 126 MHz) δ_C: 177.4 (C^{10}), 176.7 (C^{13}), 134.5 (C^7), 132.0 (C^{14}), 130.6 (C^8), 129.3 (C^{16}, $C^{16'}$), 128.8 (C^{17}), 126.6 (C^{15}, $C^{15'}$), 59.3 (C^5), 55.1 (C^6), 48.1 (C^1), 44.6 (C^{11}), 43.4 (C^{12}), 38.7 (C^9), 26.4 (C^4), 23.3 (C^2), 20.7 (C^3).

HRMS (ES^+) exact mass calculated for $[M+Na]^+$ ($C_{19}H_{21}O_2N_2$) requires **m/z** 309.1597, found **m/z** 309.1596.

9-Benzyl-5-methyl-2-phenyl-3a,4,7,7a-tetrahydro-1*H*-4,7-(epiminomethano)isoindole-1,3(2*H*)-dione (19d)

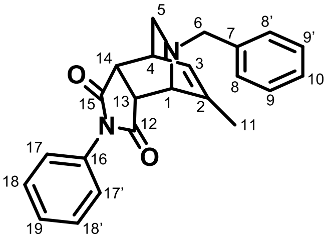

Prepared according to **General procedure E** from **15d** and *N*-phenyl maleimide. Purification *via* FCC (9 : 1 pentane/EtOAc) gave **19d** as a colourless oil (29.0 mg, 80.9 μmol, 81%)

IR 2958, 1710 (2 * C=O), 1599, 1380.

^{1}H NMR ($CDCl_3$, 500 MHz) δ_H: 7.34–7.30 (m, 2H, C17H, C17'H), 7.30–7.25 (m, 2H, C8H, C8'H), 7.21 (t, J = 7.6 Hz, 2H, C9H, C9'H), 7.15–7.08 (m, 3H, C10H, C18H, C18'H), 7.04–6.94 (m, 1H, C19H), 5.67–5.58 (m, 1H, C3H), 3.71 (dd, *J* = 4.1, 1.8 Hz, 1H, C^1H), 3.42 (d, *J* = 13.1 Hz, 1H, C^6H), 3.12 (d, *J* = 13.1 Hz, 1H, $C^{6'}$H), 3.07 (dd, *J* = 8.2, 4.1 Hz, 1H, C^{13}H), 2.81–2.75 (m, 1H, C^4H), 2.58 (dd, *J* = 10.0, 2.0 Hz, 1H, C^5H), 2.38 (dd, *J* = 8.2, 3.2 Hz, 1H, C^{14}H), 1.65 (dd, *J* = 10.0, 2.7 Hz, 1H, $C^{5'}$H), 1.61 (d, *J* = 1.7 Hz, 3H, C^{11}H).

^{13}C NMR ($CDCl_3$, 126 MHz) δ_C: 176.9 (C^{12}), 175.5 (C^{15}), 140.1 (C^2), 139.4 (C^7), 133.1 (C^{16}), 129.0 (C^8, $C^{8'}$), 128.9 (C^9, $C^{9'}$), 128.7 (C^{18}, $C^{18'}$), 128.6 (C^{10}), 127.5 (C^{19}) 127.5, 126.7 (C^{17}), 123.0 (C^3), 61.8 (C^6), 58.5 (C^1), 53.3 (C^5), 45.9 (C^{13}), 41.8 (C^{14}), 34.1 (C^4), 21.6 (C^{11}).

HRMS (ES^+) exact mass calculated for $[M+H]^+$ ($C_{23}H_{23}O_2N_2$) requires **m/z** 359.1754, found **m/z** 359.1755.

9-Benzyl-7-methyl-2,8-diphenyl-3a,4,7,7a-tetrahydro-1*H*-4,7-(epiminomethano)isoindole-1,3(2*H*)-dione (19e)

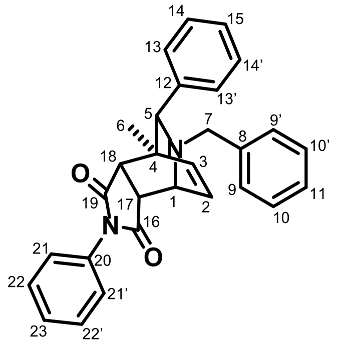

Prepared according to **General procedure E** from **15e** and *N*-phenyl maleimide. Purification *via* FCC (8 : 2 pentane/EtOAc) gave **19e** as a slightly yellow oil (37.1 mg, 85,4 µmol, 85%).

IR 3029, 2797, 1705 (C=O), 1496, 1379.

^{1}H NMR ($CDCl_3$, 400 MHz) δ_H: 7.48–7.40 (m, 2H, $C^{22}H$, $C^{22'}H$), 7.40–7.31 (m, 3H, C^9H, $C^{9'}H$, $C^{15}H$), 7.31–7.26 (m, 1H, $C^{11}H$), 7.29–7.23 (m, 6H, $C^{10}H$, $C^{10'}H$, $C^{13}H$, $C^{13'}H$, $C^{14}H$, $C^{14'}H$), 7.26–7.18 (m, 1H, $C^{23}H$), 7.18–7.12 (m, 2H, $C^{21}H$, $C^{21'}H$), 6.59 (dd, *J* = 8.1, 6.3 Hz, 1H, C^2H), 5.87 (d, *J* = 8.1 Hz, 1H, C^3H), 3.98 (ddd, *J* = 6.4, 3.5, 1.1 Hz, 1H, C^1H), 3.83 (d, *J* = 13.8 Hz, 1H, C^7H), 3.72–3.64 (m, 2H, $C^{7'}H$, $C^{17}H$), 3.19 (s, 1H, C^5H), 2.90 (d, *J* = 7.9 Hz, 1H, $C^{18}H$), 1.25 (s, 3H, C^6H).

^{13}C NMR ($CDCl_3$, 101 MHz) $\delta_{C:}$ 177.0 (C^{16}), 176.2 (C^{19}), 141.0 (C^{12}), 138.5 (C^8), 134.9 (C^3), 133.8 (C^2), 132.1 (C^{20}), 129.3 (C^{15}), 129.2 (C^{13}, $C^{13'}$), 128.8 (C^9, $C^{9'}$), 128.7 (C^{23}), 128.5 (C^{10}, $C^{10'}$), 127.8 (C^{22}, $C^{22'}$), 127.7 (C^{11}), 127.4 (C^{14}, $C^{14'}$), 126.6 (C^{21}, $C^{21'}$), 72.2 (C^6), 55.7 (C^7), 51.2 (C^1), 49.0 (C^{18}), 42.8 (C^4), 39.8 (C^{17}), 20.2 (C^{14}).

HRMS (ES^+) exact mass calculated for $[M+Na]^+$ ($C_{29}H_{27}O_2N_2$) requires **m/z** 435.2067, found **m/z** 435.2071.

9-Butyl-8-(4-fluorophenyl)-2,6-diphenyl-3a,4,7,7a-tetrahydro-1*H*-4,7-(epiminomethano)isoindole-1,3(2*H*)-dione (19f)

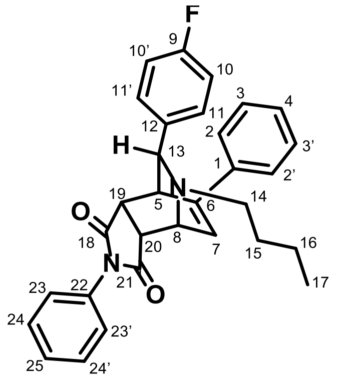

Prepared according to **General procedure E** from **15f** and *N*-phenyl maleimide. Purification *via* FCC (2 : 1 pentane/EtOAc) gave **19f** as a slightly yellow oil (45.5 mg, 94.7 μmol, 95%).

IR 2958, 1707 (2 * C=O), 1505, 1381, 1182.

^{1}H NMR ($CDCl_3$, 500 MHz) δ_H: 7.38–7.34 (m, 2H, $C^{24}H$, $C^{24'}H$), 7.33–7.28 (m, 3H, $C^{11}H$, $C^{11'}H$, $C^{25}H$), 7.16–7.10 (m, 3H, $C^{3}H$, $C^{3'}H$, $C^{4}H$), 7.02–6.98 (m, 2H, $C^{23}H$, $C^{23'}H$), 6.93–6.86 (m, 4H, $C^{2}H$, $C^{2'}H$, $C^{10}H$, $C^{10'}H$), 6.75 (dd, J = 6.6, 2.1 Hz, 1H, $C^{7}H$), 4.33 (dd, J = 6.6, 3.8 Hz, 1H, $C^{8}H$), 3.75–3.68 (m, 2H, $C^{5}H$, $C^{20}H$), 3.57 (s, 1H, $C^{13}H$), 3.42 (dd, J = 8.1, 3.5 Hz, 1H, $C^{19}H$), 2.78 (dt, J = 12.1, 7.1 Hz, 1H, $C^{14}H$), 2.63 (dt, J = 12.1, 7.2 Hz, 1H, $C^{14'}H$), 1.38–1.32 (m, 2H, $C^{15}H$), 1.32–1.21 (m, 2H, $C^{16}H$), 0.81 (t, J = 7.2 Hz, 3H, $C^{17}H$).

^{13}C NMR ($CDCl_3$, 126 MHz) δ_C: 177.4 (C^{21}), 176.8(C^{18}), 162.1 (d, J = 244.7 Hz, C^{9}), 140.8(C^{6}), 139.6 (d, J = 2.9 Hz, C^{12}), 137.4 (C^{1}), 131.8 (C^{22}), 129.2 (C^{24}, $C^{24'}$), 128.7 (m, C^{11}, $C^{11'}$, C^{25}), 128.6 (C^{3}, $C^{3'}$), 128.1 (C^{4}), 126.7 (C^{7}), 126.4 (C^{23}, $C^{23'}$), 125.2 (C^{2}, $C^{2'}$), 115.0 (d, J = 21.3 Hz, C^{10}, $C^{10'}$), 65.8 (C^{13}), 54.7 (C^{14}), 54.5 (C^{8}), 44.3 (C^{5}), 43.3 (C^{19}), 39.1 (C^{20}), 31.4 (C^{15}), 20.5 (C^{16}), 14.0 (C^{17}).

^{19}F NMR (376 MHz, $CDCl_3$) δ -116.01 – -116.19 (m).

HRMS (ES^+) exact mass calculated for $[M+Na]^+$ ($C_{31}H_{30}O_2N_2F^+$) requires **m/z** 481.2286, found **m/z** 481.2283.

The relative configuration of **19f** was assigned via the 2D NOESY correlation illustrated below:

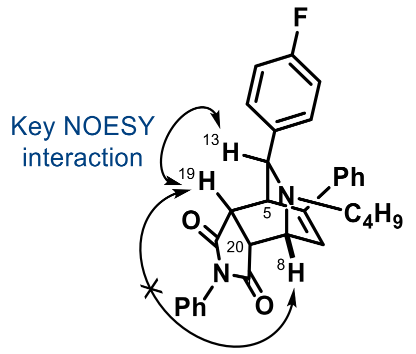
Key NOESY interaction

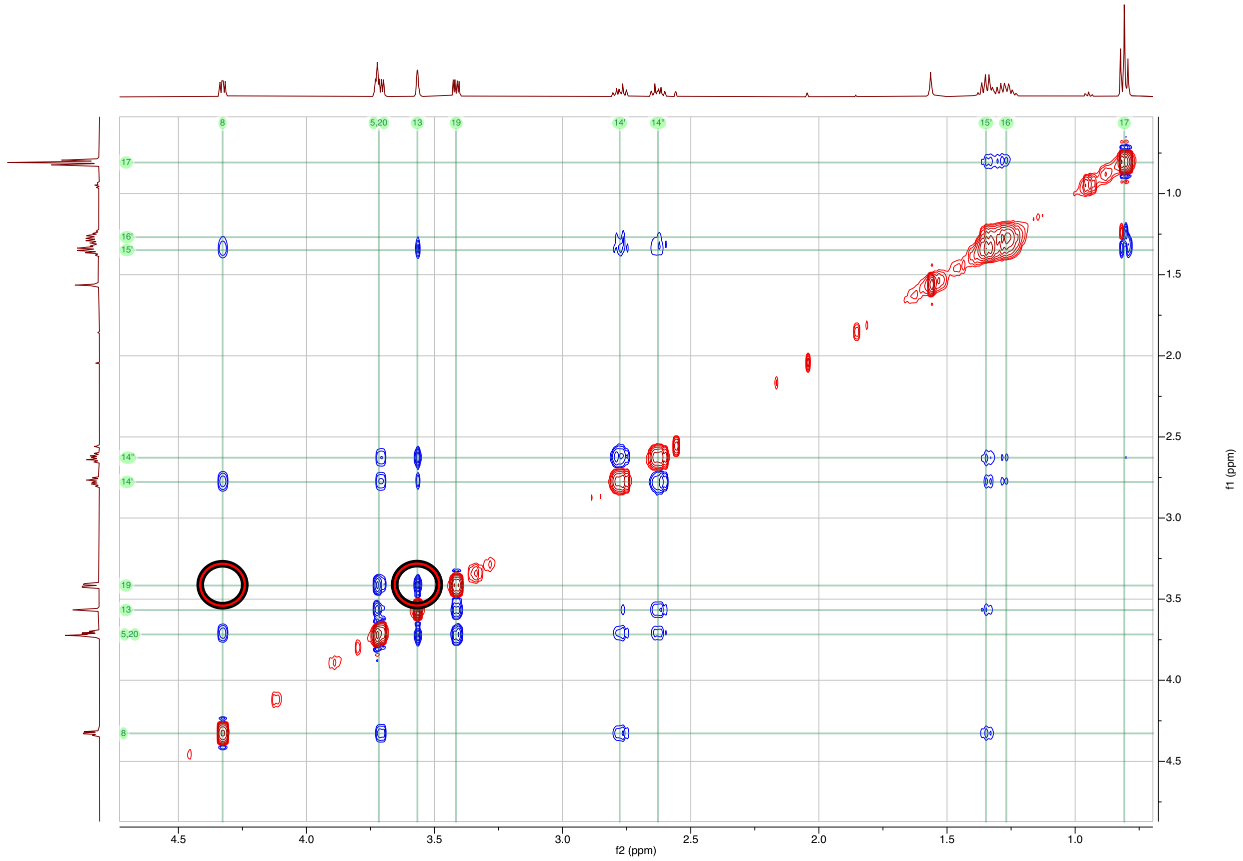
f2 (ppm)
f1 (ppm)

9-Cyclopropyl-8-(2-methoxyphenyl)-2,6-diphenyl-3a,4,7,7a-tetrahydro-1*H*-4,7-(epiminomethano)isoindole-1,3(2*H*)-dione (19g)

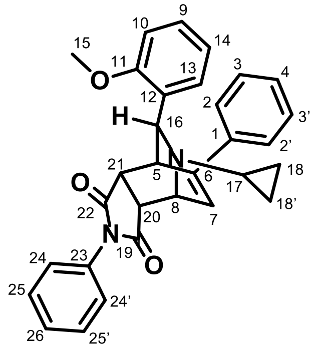

Prepared according to **General procedure E** from **15g** and *N*-phenyl maleimide. Purification *via* FCC (2 : 1 pentane/EtOAc) gave **19g** as a slightly yellow oil (46.0 mg, 96.5 μmol, 97%).

IR 2925, 1708 (2 * C=O), 1599, 1464, 1378.

^{1}H NMR ($CDCl_3$, 500 MHz) δ_H: δ 7.40–7.34 (m, 2H, $C^{25}H$, $C^{25'}H$), 7.34–7.29 (m, 1H, $C^{26}H$), 7.19 (dd, *J* = 7.7, 1.8 Hz, 1H, $C^{13}H$), 7.13–7.05 (m, 4H, $C^{3}H$, $C^{3'}H$, $C^{4}H$, $C^{9}H$), 7.04–7.00 (m, 2H, $C^{24}H$, $C^{24'}H$), 6.85–6.79 (m, 3H, $C^{2}H$, $C^{2'}H$, $C^{10}H$), 6.70 (td, *J* = 7.5, 1.1 Hz, 1H, $C^{14}H$), 6.65 (dd, *J* = 6.5, 2.2 Hz, 1H, $C^{7}H$), 4.43 (dd, *J* = 6.6, 3.9 Hz, 1H, $C^{8}H$), 4.33 (d, *J* = 2.0 Hz, 1H, $C^{16}H$), 3.94–3.92 (m, 4H, $C^{5}H$, $C^{15}H$), 3.92–3.89 (m, 1H, $C^{20}H$), 3.53 (dd, *J* = 8.0, 3.5 Hz, 1H, $C^{21}H$), 2.12 (tt, *J* = 6.7, 3.1 Hz, 1H, $C1^{7}H$), 0.54 (td, *J* = 8.2, 5.7 Hz, 2H, $C^{18'}H$), 0.29 (dtt, *J* = 15.4, 7.2, 2.8 Hz, 2H, $C^{18}H$).

^{13}C NMR ($CDCl_3$, 126 MHz) δ_C: 177.7 (C^{19}), 177.2 (C^{22}), 156.5 (C^{12}), 141.7 (C^{6}), 137.9 (C^{1}), 132.0 (C^{23}), 131.5 (C^{11}), 129.2 (C^{25}, $C^{25'}$), 129.0 (C^{13}), 128.6 (C^{9}), 128.4 (C^{3}, $C^{3'}$), 127.8 (C^{26}), 127.7 (C^{4}), 126.5 (C^{24}, $C^{24'}$), 126.0 (C^{7}), 125.2 (C^{2}, $C^{2'}$), 120.3 (C^{14}), 109.4 (C^{10}), 59.5 (C^{16}), 55.5 (C^{5}), 55.3 (C^{8}), 43.0 (C^{21}), 41.5 (C^{15}), 40.1 (C^{20}), 34.9 (C^{17}), 6.9 ($C^{18'}$), 6.1 (C^{18}).

HRMS (ES^+) exact mass calculated for $[M+H]^+$ ($C_{31}H_{29}O_3N_2$) requires **m/z** 477.2173, found **m/z** 477.2168.

9-Cyclopropyl-8-(2-methoxyphenyl)-6-phenyl-3a,4,7,7a-tetrahydro-1*H*-4,7-(epiminomethano)isoindole-1,3(2*H*)-dione (19h)

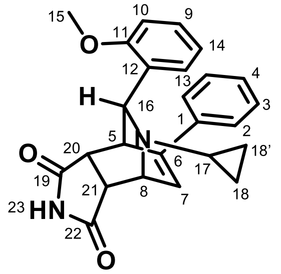

Prepared according to **General procedure E** from **15g** and malemide **22h**. Purification *via* FCC (9 : 1 pentane/EtOAc) gave **19h** as a yellow oil (34.0 mg, 84.9 μmol, 85%).

IR 3005, 2950, 1731 (C=O), 1599, 1486, 1435.

^{1}H NMR ($CDCl_3$, 500 MHz) δ_H: 7.17 (dd, *J* = 7.6, 1.8 Hz, 1H, $C^{13}H$), 7.08 (qd, *J* = 5.3, 4.3, 2.0 Hz, 4H, C^3H, $C^{3'}H$, C^4H, C^9H), 6.80 (dd, *J* = 8.1, 1.1 Hz, 1H, $C^{10}H$), 6.73–6.66 (m, 3H, C^2H, $C^{2'}H$, $C^{14}H$), 6.55 (dd, *J* = 6.6, 2.0 Hz, 1H, C^7H), 4.20 (q, *J* = 3.1 Hz, 2H, C^8H,), 4.12 (dd, *J* = 5.5, 2.7 Hz, 1H, $C^{16}H$), 3.88 (d, *J* = 6.6 Hz, 6H, $C^{23}H$, $C^{24}H$), 3.71 (s, 3H, $C^{15}H$), 3.61 (q, *J* = 2.0 Hz, 1H, $C^{20}H$), 3.21 (dd, *J* = 5.5, 2.8 Hz, 1H, C^5H), 2.33 (dq, *J* = 6.6, 3.4 Hz, 1H, $C^{17}H$), 0.57–0.35 (m, 2H, $C^{18}H$), 0.29–0.16 (m, 2H, $C^{18'}H$).

^{13}C NMR ($CDCl_3$, 126 MHz) δ_C: 174.3 (C^{22}), 174.0 (C^{19}), 156.5 (C^{11}), 143.3 (C^6), 138.5 (C^1), 132.6 (C^{12}), 129.0 (C^{13}), 128.2 (C^3, $C^{3'}$), 127.5 (C^9), 127.4 (C^4), 127.3 (C^7), 125.4 (C^2, $C^{2'}$), 120.4 (C^{14}), 109.5 (C^{10}), 56.2 (C^8), 55.7 (C^{23}), 55.5 (C^{21}), 52.3 (C^{24}), 52.2 (C^{15}), 45.3 (C^5), 42.8 (C^{20}), 40.3 (C^{16}), 33.9 (C^{17}), 6.9 (C^{18}), 5.8 ($C^{18'}$).

HRMS (ES^+) exact mass calculated for $[M+H]^+$ ($C_{27}H_{30}O_5N$) requires **m/z** 448.2118, found **m/z** 448.2121.

2-Cyclopropyl-3-(2-methoxyphenyl)-8-phenyl-2-azabicyclo[2.2.2]oct-7-ene-6-carbonyl)oxazolidin-2-one (19i)

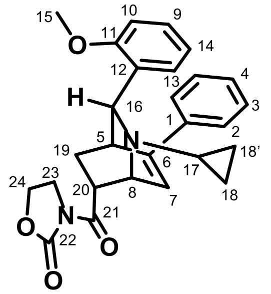

Prepared according to **General procedure E** from **15g** and oxazolidinone **21i**. Purification *via* FCC (8 : 2 pentane/EtOAc) gave **19i** as a colourless oil (40.0 mg, 90.0 μmol, 90%).

IR 2922, 1778 (C=O), 1693, 1599, 1485, 1384.

^{1}H NMR ($CDCl_3$, 500 MHz) δ_H: 7.21 (dd, *J* = 7.6, 1.8 Hz, 1H, $C^{13}H$), 7.12–7.00 (m, 4H, C^3H, $C^{3'}H$, C^4H, C^9H), 6.83 (dd, *J* = 8.2, 1.1 Hz, 1H, $C^{10}H$), 6.79–6.73 (m, 2H, C^2H, $C^{2'}H$), 6.69 (td, *J* = 7.4, 1.1 Hz, 1H, $C^{14}H$), 6.49 (dd, *J* = 6.6, 2.1 Hz, 1H, C^7H), 4.66 (ddd, *J* = 9.6, 5.3, 2.5 Hz, 1H, $C^{20}H$), 4.48–4.32 (m, 2H, $C^{23}H$, $C^{23'}H$), 4.24 (d, *J* = 1.9 Hz, 1H, $C^{16}H$), 4.11 (dd, *J* = 6.6, 2.5 Hz, 1H, C^8H), 4.04–3.95 (m, 2H, $C^{24}H$, $C^{24'}H$), 3.91 (s, 3H, $C^{15}H$), 3.22 (t, *J* = 2.3 Hz, 1H, C^5H), 2.38–2.19 (m, 2H, $C^{19}H$, $C^{17}H$), 1.96 (ddd, *J* = 12.6, 5.3, 3.0 Hz, 1H, $C^{19'}H$), 0.50 (ddt, *J* = 10.9, 6.5, 3.2 Hz, 1H, $C^{18}H$), 0.46 – 0.39 (m, 1H, $C^{18'}H$), 0.28–0.15 (m, 2H, $C^{18''}H$, $C^{18'''}H$).

^{13}C NMR ($CDCl_3$, 126 MHz) δ_C: 174.8 (C^{21}), 156.6 (C^{11}), 153.4 (C^{22}), 143.4 (C^5), 139.2 (C^1), 133.6 (C^{12}), 129.0 (C^{13}), 128.1 (C^3, $C^{3'}$), 127.1 (C^9), 126.9 (C^4), 126.4 (C^7), 125.5 (C^2, $C^{2'}$), 120.3 (C^{14}), 109.4 (C^{10}), 62.1 (C^{23}), 59.9 (C^{16}), 56.1 (C^8), 55.5 (C^{15}), 43.2 (C^{24}), 40.0 (C^5), 37.8 (C^{20}), 34.8 (C^{17}), 28.9 (C^{19}), 6.7 (C^{18}), 6.0 ($C^{18'}$).

HRMS (ES^+) exact mass calculated for $[M+H]^+$ ($C_{27}H_{29}O_4N_2$) requires **m/z** 445.2122, found **m/z** 445.2118.

Methyl 2-cyclopropyl-3-(2-methoxyphenyl)-8-phenyl-2-azabicyclo[2.2.2]oct-7-ene-6-carboxylate (19j)

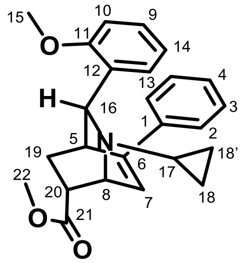

Prepared according to **General procedure E** from **15g** and methyl acrylate **22j**. Purification *via* FCC (9 : 1 pentane/EtOAc) gave **19j** as a slightly yellow oil (35.8 mg, 91.9 µmol, 92%).

IR 3006, 2960, 1733 (C=O), 1599, 1485, 1367.

^{1}H NMR ($CDCl_3$, 500 MHz) δ_H: 7.20 (dd, *J* = 7.5, 1.8 Hz, 1H, $C^{13}H$), 7.11–7.01 (m, 4H, $C^{3}H$, $C^{3'}H$, $C^{4}H$, $C^{9}H$), 6.82 (d, *J* = 8.1, 1.1 Hz, 1H, $C^{10}H$), 6.76–6.72 (m, 2H, $C^{2}H$, $C^{2'}H$), 6.70 (t, *J* = 7.4, 1.1 Hz, 1H, $C^{14}H$), 6.50 (dd, *J* = 6.6, 2.1 Hz, 1H, $C^{7}H$), 4.17 (d, *J* = 2.0 Hz, 1H, $C^{16}H$), 4.14 (dd, *J* = 6.6, 2.9 Hz, 1H, $C^{7}H$), 3.91 (s, 3H, $C^{15}H$), 3.70 (s, 3H, $C^{22}H$), 3.48 (ddd, *J* = 9.6, 4.9, 2.9 Hz, 1H, $C^{20}H$), 3.25–3.21 (m, 1H, $C^{5}H$), 2.16 (ddd, *J* = 12.6, 9.6, 2.8 Hz, 1H, $C^{19}H$), 2.13–2.06 (m, 1H, $C^{17}H$), 2.06–1.98 (m, 1H, $C^{19'}H$), 0.53–0.41 (m, 2H, $C^{18}H$), 0.32–0.18 (m, 2H, $C^{18'}H$).

^{13}C NMR ($CDCl_3$, 126 MHz) δ_C: 175.4 (C^{21}), 156.6 (C^{11}), 143.9 (C^{6}), 139.2 (C^{1}), 133.6 (C^{12}), 129.0 (C^{13}), 128.1 (C^{3},$C^{3'}$), 127.1 (C^{9}), 126.9 (C^{4}), 126.3 (C^{7}), 125.4 (C^{2},$C^{2'}$), 120.3 (C^{14}), 109.4 (C^{10}), 60.3 (C^{16}), 56.0 (C^{8}), 55.5 (C^{15}), 51.9 (C^{22}), 39.8 (C^{5f}), 37.4 (C^{20}), 34.9 (C^{17}), 28.3 (C^{19}), 6.8 (C^{18}), 5.8 ($C^{18'}$).

HRMS (ES^+) exact mass calculated for $[M+H]^+$ ($C_{25}H_{28}O_3N$) requires **m/z** 390.2064, found **m/z** 390.2058.

Dimethyl 2-cyclopropyl-3-(2-methoxyphenyl)-8-phenyl-2-azabicyclo[2.2.2]oct-7-ene-5,6-dicarboxylate (19k)

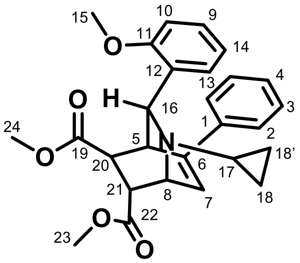

Prepared according to **General procedure E** from **15g** and dimethyl fumarate **22k**. Purification *via* FCC (9 : 1 pentane/EtOAc) gave **19k** as a yellow oil (42.5 mg, 95.0 mmol, 95%).

IR 3005, 2950, 1731 (C=O), 1599, 1486, 1435.

^{1}H NMR ($CDCl_3$, 500 MHz) δ_H: 7.17 (dd, *J* = 7.6, 1.8 Hz, 1H, $C^{13}H$), 7.08 (qd, *J* = 5.3, 4.3, 2.0 Hz, 4H, C^3H, $C^{3'}H$, C^4H, C^9H), 6.80 (dd, *J* = 8.1, 1.1 Hz, 1H, $C^{10}H$), 6.73–6.66 (m, 3H, C^2H, $C^{2'}H$, $C^{14}H$), 6.55 (dd, *J* = 6.6, 2.0 Hz, 1H, C^7H), 4.20 (q, *J* = 3.1 Hz, 2H, C^8H,), 4.12 (dd, *J* = 5.5, 2.7 Hz, 1H, $C^{16}H$), 3.88 (d, *J* = 6.6 Hz, 6H, $C^{23}H$, $C^{24}H$), 3.71 (s, 3H, $C^{15}H$), 3.61 (q, *J* = 2.0 Hz, 1H, $C^{20}H$), 3.21 (dd, *J* = 5.5, 2.8 Hz, 1H, C^5H), 2.33 (dq, *J* = 6.6, 3.4 Hz, 1H, $C^{17}H$), 0.57 – 0.35 (m, 2H, $C^{18}H$), 0.29–0.16 (m, 2H, $C^{18'}H$).

^{13}C NMR ($CDCl_3$, 126 MHz) δ_C: 174.3 (C^{22}), 174.0 (C^{19}), 156.5 (C^{11}), 143.3 (C^6), 138.5 (C^1), 132.6 (C^{12}), 129.0 (C^{13}), 128.2 (C^3, $C^{3'}$), 127.5 (C^9), 127.4 (C^4), 127.3 (C^7), 125.4 (C^2, $C^{2'}$), 120.4 (C^{14}), 109.5 (C^{10}), 56.2 (C^8), 55.7 (C^{23}), 55.5 (C^{21}), 52.3 (C^{24}), 52.2 (C^{15}), 45.3 (C^5), 42.8 (C^{20}), 40.3 (C^{16}), 33.9 (C^{17}), 6.9 (C^{18}), 5.8 ($C^{18'}$).

HRMS (ES^+) exact mass calculated for $[M+H]^+$ ($C_{27}H_{30}O_5N$) requires **m/z** 448.2118, found **m/z** 448.2121.

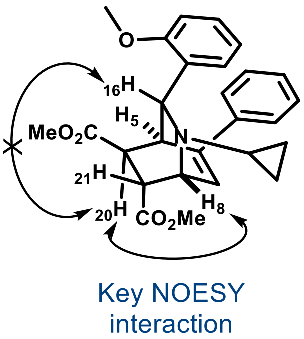

2-Cyclopropyl-3-(2-methoxyphenyl)-8-phenyl-2-azabicyclo[2.2.2]oct-7-ene-6-carbonitrile (19l)

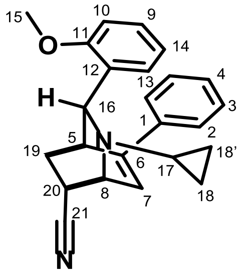

Prepared according to **General procedure E** from **15g** and acrylonitrile **22l**. Purification *via* FCC (8 : 2 pentane/EtOAc) gave **19l** as a white solid (major 21.4 mg, 60.2 μmol, 60%; minor 12.1 mg, 12.1 μmol, 33.8%, total 33.5 mg, 94.0 μmol, 94%).

Data for major isomer:

mp 175 °C

IR 3007, 2957, 2231 (CN), 1599, 1485, 1359.

^{1}H NMR (CDCl$_3$, 500 MHz) δ$_H$: 7.15–7.06 (m, 5H, C^{3}H, C$^{3'}$H, C^{4}H, C^{9}H, C^{13}H), 6.83 (dd, *J* = 8.2, 1.1 Hz, 1H, C^{10}H), 6.80–6.76 (m, 2H, C^{2}H, C$^{2'}$H), 6.73–6.67 (m, 2H, C^{7}H, C^{14}H), 4.16 (d, *J* = 2.0 Hz, 1H, C^{16}H), 4.11 (dd, *J* = 6.6, 2.8 Hz, 1H, C^{8}H), 3.91 (s, 3H, C^{15}H), 3.59–3.42 (m, 1H, C^{20}H), 3.29 (m, 1H, C^{5}H), 2.42 (ddd, *J* = 12.7, 9.8, 2.8 Hz, 1H, C^{19}H), 2.04–1.96 (m, 1H, C^{17}H), 1.88–1.69 (m, 1H, C$^{19'}$H), 0.52–0.42 (m, 2H, C^{18}H), 0.31–0.20 (m, 2H, C$^{18'}$H).

^{13}C NMR (CDCl$_3$, 126 MHz) δ$_C$: 156.4 (C^{11}), 144.8 (C^{12}), 138.3 (C^{1}), 132.4 (C^{6}), 128.8 (C^{13}), 128.3 (C^{3}, C$^{3'}$), 127.5 (C^{4}, C^{9}), 125.9 (C^{7}), 125.5 (C^{2}, C$^{2'}$), 122.9 (C^{21}), 120.4 (C^{14}), 109.5 (C^{10}), 59.6 (C^{16}), 55.7 (C^{15}), 55.5 (C^{8}), 38.7 (C^{5}), 34.8 (C^{17}), 30.5 (C^{19}), 22.5 (C^{20}), 7.0 (C^{18}), 5.7 (C$^{18'}$).

HRMS (ES^{+}) exact mass calculated for [M+H]$^{+}$ ($C_{24}H_{24}ON_2$) requires **m/z** 357.1961, found **m/z** 357.1963.

Data for minor isomer:

mp 169 °C

IR 3008, 2935, 2233 (CN), 1724, 1486.

^{1}H NMR (CDCl$_3$, 500 MHz) δ$_H$: 7.13–7.05 (m, 5H, C^{3}H, C$^{3'}$H, C^{4}H, C^{9}H, C^{13}H), 6.82 (d, *J* = 8.7 Hz, 1H, C^{10}H), 6.78–6.71 (m, 2H, C^{2}H, C$^{2'}$H), 6.68 (t, *J* = 7.5, 1.1 Hz, 1H, C^{14}H), 6.63 (dd, *J* = 6.8, 2.1 Hz, 1H, C^{7}H), 4.54 (d, *J* = 2.1 Hz, 1H, C^{16}H), 4.25 (dd, *J* = 6.8, 1.5 Hz, 1H, C^{8}H), 3.93 (s, 3H, C^{15}H), 3.53–

3.14 (m, 1H, C^5H), 2.83 (septet, *J* = 6.7, 3.5 Hz, 1H, $C^{17}H$), 2.64 (ddd, *J* = 11.6, 5.8, 1.5 Hz, 1H, $C^{20}H$), 2.35 (ddd, *J* = 13.1, 5.8, 2.4 Hz, 1H, $C^{19}H$), 2.12 (ddd, *J* = 13.2, 11.7, 3.6 Hz, 1H, $C^{19'}H$), 0.65–0.56 (m, 1H, $C^{18}H$), 0.54–0.45 (m, 1H, 18), 0.30–0.14 (m, 2H, $C^{18'}H$).

^{13}C NMR ($CDCl_3$, 126 MHz) δ_C: 156.6 (C^{11}), 142.5 (C^{12}), 138.3 (C^1), 132.3 (C^6), 128.8 (C^{13}), 128.3 (C^3, $C^{3'}$), 127.9 (C^7), 127.5 (d, *J* = 3.2 Hz, C^4, C^9), 125.2 (C^2, $C^{2'}$), 123.4 (C^{21}), 120.3 (C^{14}), 109.6 (C^{10}), 60.5 (C^{16}), 55.5 (C^{15}), 55.2 (C^8), 38.6 (C^5), 35.3 (C^{17}), 29.3 (C^{19}), 26.7 (C^{20}), 8.4 (C^{18}), 5.5 ($C^{18'}$).

HRMS (ES^+) exact mass calculated for $[M+H]^+$ ($C_{24}H_{24}ON_2$) requires **m/z** 357.1961, found **m/z** 357.1963.

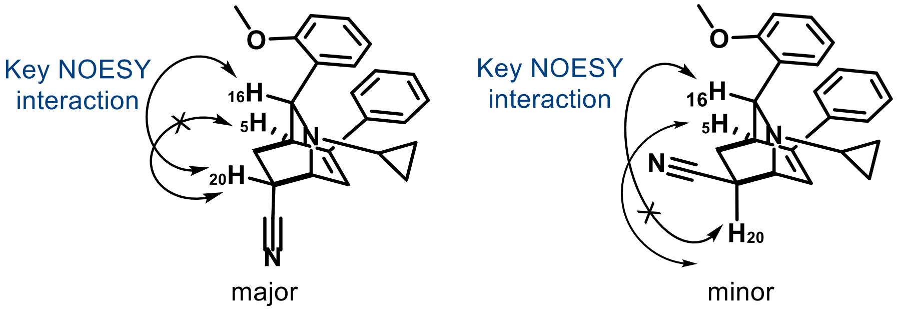

4-(Benzyl(methyl)amino)-2-phenyl-3a,4,7,7a-tetrahydro-1*H*-isoindole-1,3(2*H*)-dione (24a)

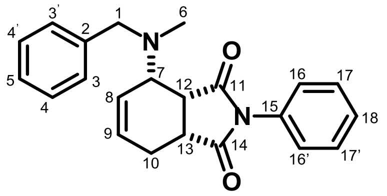

Prepared according to a modification of **General procedure E** (reaction time: 3 h) from **22a** and *N*-phenyl maleimide. Purification *via* recrystallization in ether at 0 °C gave **24a** as a slightly yellow solid (27.8 mg, 80.3 μmol, 80%).

mp 106 °C

IR 2891, 1709 (2 * C=O), 1598, 1497, 1381.

^{1}H NMR (C_6D_6, 400 MHz) δ_H: 7.50–7.40 (m, 4H, C^3H, $C^{3'}H$, $C^{16}H$, $C^{16'}H$), 7.24–7.11 (m, 5H, C^4H, $C^{4'}H$, C^5H, $C^{17}H$, $C^{17'}H$), 7.07–6.98 (m, 1H, $C^{18}H$), 5.74 (ddd, *J* = 9.8, 4.3, 2.2 Hz, 1H, C^8H), 5.68–5.58 (m, 1H, C^9H), 3.78 (d, *J* = 13.4 Hz, 1H, C^1H), 3.53 (d, *J* = 13.4 Hz, 1H, C^1H), 3.21–3.12 (m, 1H, C^7H), 2.83 (dd, *J* = 9.4, 7.0 Hz, 1H, $C^{12}H$), 2.62 (ddd, *J* = 16.5, 5.5, 2.7 Hz, 1H, $C^{10}H$), 2.45 (ddd, *J* = 9.4, 8.5, 2.7 Hz, 1H, $C^{13}H$), 2.18 (s, 3H, C^6H), 1.82–1.69 (m, 1H, $C^{10'}H$).

^{13}C NMR (C_6D_6, 101 MHz) δ_C: 178.1 (C^{11}), 175.4 (C^{14}), 140.1 (C^{2}), 133.3 (C^{15}), 129.2 (C^{8}), 129.0 (C^{3}, $C^{3'}$), 128.6 (C^{9}), 128.3 (C^{4}, $C^{4'}$), 128.2 (C^{5}), 127.9 (C^{18}), 127.3 (C^{17}, $C^{17'}$), 126.8 (C^{16}, $C^{16'}$), 60.5 (C^{1}), 59.8 (C^{7}), 42.8 (C^{12}), 39.5 (C^{6}), 39.2 (C^{13}), 23.0 (C^{10}).

HRMS (ES$^+$) exact mass calculated for $[M+Na]^+$ ($C_{22}H_{23}O_2N_2$) requires **m/z** 347.1754, found **m/z** 347.1753.

2-Phenyl-4-(piperidin-1-yl)-3a,4,7,7a-tetrahydro-1*H*-isoindole-1,3(2*H*)-dione (24b)

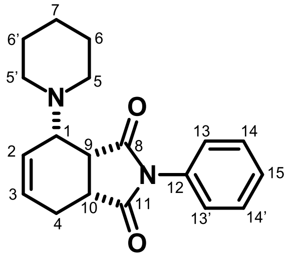

Prepared according to a modification of **General procedure E** (reaction time: 3 h) from **22b** and *N*-phenyl maleimide. Purification *via* recrystallization in ether at 0 °C gave **24b** as a white solid (27.1 mg, 87.3 μmol, 87%).

mp 182 °C

IR 2933, 1708 (2 * C=O), 1598, 1499, 1380.

^{1}H NMR (C_6D_6, 400 MHz) δ_H: 7.60–7.52 (m, 2H, $C^{13}H$, $C^{13'}H$), 7.19–7.13 (m, 2H, $C^{14}H$, $C^{14'}H$), 7.05–6.94 (m, 1H, $C^{15}H$), 5.75–5.66 (m, 1H, $C^{2}H$), 5.65–5.56 (m, 1H, $C^{3}H$), 2.98–2.90 (m, 1H, $C^{1}H$), 2.75 (dd, J = 9.4, 6.6 Hz, 1H, $C^{9}H$), 2.64–2.50 (m, 3H, $C^{4}H$, $C^{5}H$, $C^{5'}H$), 2.46–2.36 (m, 1H, $C^{10}H$), 2.41–2.31 (m, 2H, $C^{5}H$, $C^{5'}H$), 1.87–1.75 (m, 1H, $C^{4}H$), 1.60–1.41 (m, 4H, $C^{6}H$, $C^{6'}H$), 1.25 (p, J = 6.0 Hz, 2H, $C^{7}H$).

^{13}C NMR (C_6D_6, 101 MHz) δ_C: 178.2 (C^{8}), 175.1 (C^{11}), 133.4 (C^{12}), 128.8 (C^{2}), 128.2 (C^{3}), 127.9 (C^{15}), 127.9 (C^{14}, $C^{14'}$), 126.5 (C^{13}, $C^{13'}$), 60.9 (C^{1}), 53.4 (C^{5}, $C^{5'}$), 43.0 (C^{9}), 39.0 (C^{10}), 26.8 (C^{6}, $C^{6'}$), 24.9 (C^{7}), 22.7 (C^{4}).

HRMS (ES$^+$) exact mass calculated for $[M+Na]^+$ ($C_{19}H_{22}O_2N_2$) requires **m/z** 311.1754, found **m/z** 311.1755.

4-(Benzyl(2-(1-tosyl-1*H*-indol-3-yl)ethyl)amino)-2-phenyl-3a,4,7,7a-tetrahydro-1*H*-isoindole-1,3(2*H*)-dione (24c)

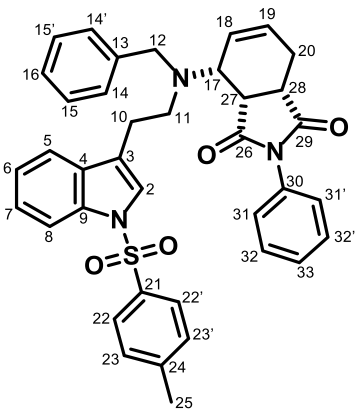

Prepared according to a modification of **General procedure E** (reaction time: 3 h) from **22c** and *N*-phenyl maleimide. Purification *via* recrystallization in ether at 0 °C gave **24c** as a slightly white solid (49.2 mg, 78.1 μmol, 78%).

mp 107 °C

IR 3029, 2797, 1705 (C=O), 1496, 1369.

^{1}H NMR ($CDCl_3$, 500 MHz) δ_H: 8.27 (d, *J* = 8.3 Hz, 1H, C^8H), 7.69–7.63 (m, 2H $C^{22}H$, $C^{22'}H$), 7.47 (s, 1H, C^2H), 7.44–7.33 (m, 4H, $C^{15}H$, $C^{15'}H$, $C^{31}H$, $C^{31'}H$), 7.27 (d, *J* = 7.9 Hz, 1H, C^5H), 7.20 (t, *J* = 7.5 Hz, 2H, $C^{14}H$, $C^{14'}H$), 7.15–7.08 (m, 4H, C^7H, $C^{16}H$, $C^{32}H$, $C^{32'}H$), 7.06–6.94 (m, 2H, C^6H, $C^{33}H$), 6.47 (d, *J* = 8.2 Hz, 2H, $C^{23}H$, $C^{23'}H$), 5.73 (dd, *J* = 9.8, 3.2 Hz, 1H, $C^{18}H$), 5.58 (ddt, *J* = 9.5, 6.2, 3.0 Hz, 1H, $C^{19}H$), 3.99 (d, *J* = 14.9 Hz, 1H, $C^{12}H$), 3.78 (d, *J* = 14.8 Hz, 1H, $C^{12'}H$), 3.48 (dq, *J* = 8.0, 2.7 Hz, 1H, $C^{17}H$), 3.03–2.89 (m, 2H, $C^{11}H$), 2.86 (dd, *J* = 9.3, 7.8 Hz, 1H, $C^{27}H$), 2.79 (ddd, *J* = 14.9, 9.4, 5.5 Hz, 1H, $C^{10}H$), 2.70–2.54 (m, 2H, $C^{10'}H$, $C^{20}H$), 2.33 (ddd, *J* = 9.5, 7.3, 2.3 Hz, 1H, $C^{28}H$), 1.61 (s, 3H, $C^{25}H$), 1.45 (ddq, *J* = 15.9, 7.6, 2.8 Hz, 1H, $C^{20'}H$).

^{13}C NMR ($CDCl_3$, 126 MHz) δ_C: 177.8 (C^{29}), 176.2 (C^{26}), 144.3 (C^{24}), 140.9 (C^{13}), 136.2 (C^{21}), 136.0 (C^9), 133.1 (C^{30}), 131.7 (C^4), 129.8 (C^{23}, $C^{23'}$), 129.6 (C^{18}), 129.0 (C^{32}, $C^{32'}$), 128.7 (C^{15}, $C^{15'}$), 128.7 (C^{14}, $C^{14'}$), 128.3 (C^{19}), 127.4 (C^7), 126.9 (C^{22}, $C^{22'}$), 126.5 (C^{31}, $C^{31'}$), 126.0 (C^{33}), 124.9 (C^{16}), 123.8 (C^2), 123.4 (C^6), 121.6 (C^3), 120.0 (C^5), 114.3 (C^8), 58.0 (C^{17}), 56.9 (C^{12}), 52.6 (C^{11}), 43.2 (C^{27}), 39.7 (C^{28}), 25.4 (C^{10}), 22.9 (C^{20}), 21.0 (C^{25}).

HRMS (ES^+) exact mass calculated for $[M+Na]^+$ ($C_{38}H_{36}O_4N_3{}^{32}S$) requires **m/z** 630.2421, found **m/z** 630.2415.

3-(2-(Benzyl(methyl)amino)cyclohex-3-ene-1-carbonyl)oxazolidin-2-one (24d)

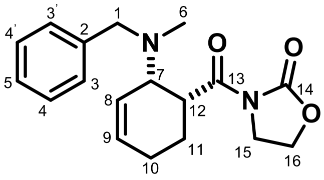

Prepared according to a modification of **General procedure E** (reaction time: 3 h, catalyst loading: 0.5 mol%, 2 equivalents oxazolidinone **21i**, 48 h reaction time) from **22a** and oxazolidinone **21i**. Purification *via* FCC (9 : 1 : 1 pentane:EtOAc:Et_3N) at 0 °C gave **24d** as a colourless oil (23.1 mg, 73.5 μmol, 74%).

IR 3022, 2923, 1774 (C=O) 1701 (C=O), 1385.

^{1}H NMR (C_6D_6, 500 MHz) δ_H: 7.29–7.24 (m, 2H, C^3H, $C^{3'}$H), 7.22–7.17 (m, 2H, C^4H, $C^{4'}$H), 7.14–7.06 (m, 1H, C^5H), 5.92–5.81 (m, 1H, C^9H), 5.75–5.67 (m, 1H, C^8H), 4.24–4.11 (m, 1H, C^7H), 4.01 (ddd, *J* = 14.0, 7.4, 3.3 Hz, 1H, C^{12}H), 3.64 (d, *J* = 13.2 Hz, 1H, C^1H), 3.43 (d, *J* = 13.1 Hz, 1H, $C^{1'}$H), 3.41–3.35 (m, 1H, C^{16}H), 3.31–3.21 (m, 2H, C^{15}H, $C^{16'}$H), 3.19–3.12 (m, 1H, $C^{15'}$H), 2.25–2.17 (m, 4H, C^6H, C^{11}H), 1.96–1.87 (m, 1H, C^{10}H), 1.81–1.69 (m, 2H, $C^{10'}$H, $C^{11'}$H).

^{13}C NMR (C_6D_6, 126 MHz) δ_C: 173.4 (C^{13}), 152.8 (C^{14}), 140.5 (C^2), 130.8(C^9), 128.7 (C^3, $C^{3'}$), 128.1 (C^4, $C^{4'}$), 126.8 (C^5), 123.2 (C^8), 60.9 (C^{15}), 60.7 (C^1), 56.0 (C^7), 44.9 (C^{12}), 42.3 (C^{16}), 39.6 (C^6), 24.2 (C^{10}), 21.3 (C^{11}).

HRMS (ES^+) exact mass calculated for $[M+H]^+$ ($C_{18}H_{23}O_3N_2$) requires **m/z** 315.1703, found **m/z** 315.1704.

Note: stereochemistry was assigned by analogy.

4-(benzyl(methyl)amino)-3a,4,7,7a-tetrahydro-1H-isoindole-1,3(2H)-dione (24e)

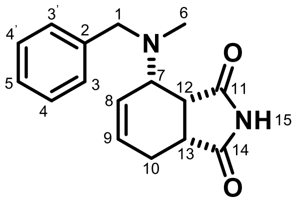

Prepared according to a modification of **General procedure E** (reaction time: 3 h) from **22a** and maleimide **22e**. Purification *via* recrystallization in 1 : 1 pentane and ether at 0 °C gave **24e** as a slightly yellow solid (23.7 mg, 87.7 μmol, 87%).

mp 136 °C

IR 3215, 3060, 2847, 1709 (2 * C=O), 1494, 1354.

^{1}H NMR ($CDCl_3$, 400 MHz) δ_H: 7.93 (br s, 1H, C^{16}H), 7.32–7.21 (m, 4H, C^{3}H, C$^{3'}$H, C^{4}H, C$^{4'}$H), 7.22–7.13 (m, 1H, C^{5}H), 6.01 (ddd, *J* = 9.7, 3.9, 2.3 Hz, 1H, C^{8}H), 5.98–5.89 (m, 1H, C^{9}H), 3.81 (d, *J* = 13.6 Hz, 1H, C^{1}H), 3.58 (d, J = 13.6 Hz, 1H, C$^{1'}$H), 3.39 (dd, *J* = 9.1, 6.9 Hz, 1H, C^{12}H), 3.35–3.26 (m, 1H, C^{7}H), 3.12 (ddd, *J* = 9.1, 8.3, 2.5 Hz, 1H, C^{13}H), 2.64 (ddd, *J* = 16.2, 5.7, 2.5 Hz, 1H, C^{10}H), 2.24 (s, 3H, C^{6}H), 2.21–2.08 (m, 1H, C$^{10'}$H).

^{13}C NMR ($CDCl_3$, 101 MHz) δ_C: 179.6 (C^{14}), 176.9 (C^{11}), 139.0 (C^{2}), 129.9 (C^{8}), 129.0 (C^{3}, C$^{3'}$), 128.4 (C^{4}, C$^{4'}$), 128.0 (C^{9}), 127.2 (C^{5}), 59.8 (C^{1}, C^{7}), 43.9 (C^{12}), 41.0 (C^{13}), 40.0 (C^{6}), 23.0 (C^{10}).

HRMS (ES^{+}) exact mass calculated for $[M+H]^{+}$ ($C_{16}H_{19}O_2N_2$) requires **m/z** 271.1441, found **m/z** 271.1442.

4-(benzyl(methyl)amino)-2-methyl-3a,4,7,7a-tetrahydro-1H-isoindole-1,3(2H)-dione (24f)

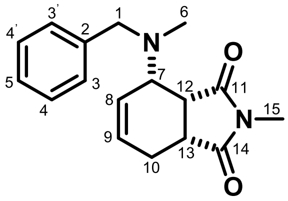

Prepared according to a modification of **General procedure E** (reaction time: 3 h) from **22a** and *N*-methyl maleimide. Purification *via* recrystallization in 1 : 1 pentane and ether at 0 °C gave **24f** as a slightly yellow solid (23.8 mg, 83.7 μmol, 84%).

mp 130 °C

IR 2653, 1697 (2 * C=O), 1434, 1383.

^{1}H NMR (C_6D_6, 500 MHz) δ_H: 7.61–7.54 (m, 2H, C^3H, $C^{3'}$H), 7.37 – 7.30 (m, 2H, C^4H, $C^{4'}$H), 7.25–7.17 (m, 1H, C^5H), 5.83 (ddd, *J* = 9.6, 4.0, 2.7 Hz, 1H, C^8H), 5.70–5.60 (m, 1H, C^9H), 3.86 (d, *J* = 13.6 Hz, 1H, C^1H), 3.61 (d, *J* = 13.6 Hz, 1H, $C^{1'}$H), 3.02 (dddd, *J* = 6.5, 4.0, 2.3, 1.5 Hz, 1H, C^7H), 2.82–2.78 (m, 1H, C^{12}H), 2.77 (s, 3H, C^{15}H), 2.64 (ddd, *J* = 15.9, 6.3, 2.4 Hz, 1H, C^{10}H), 2.42–2.34 (m, 1H, C^{13}H), 2.30 (s, 3H, C^6H), 1.80–1.63 (m, 1H, $C^{10'}$H).

^{13}C NMR (C_6D_6, 126 MHz) δ_C: 178.4 (C^{14}), 175.7 (C^{11}), 140.0 (C^2), 130.4 (C^8), 128.7 (C^3, $C^{3'}$), 128.2 (C^4, $C^{4'}$), 127.3 (C^9), 126.9 (C^5), 60.2 (C^7), 59.5 (C^1), 42.1 (C^{12}), 39.4 (C^6), 39.4 (C^{13}), 24.1 (C^{15}), 22.9 (C^{10}).

HRMS (ES^+) exact mass calculated for $[M+H]^+$ ($C_{17}H_{21}O_2N_2$) requires **m/z** 285.1598, found **m/z** 285.1598.

4-Morpholino-2-phenylpyrrolo[3,4-*a*]carbazole-1,3(2*H*,10*H*)-dione (27a)

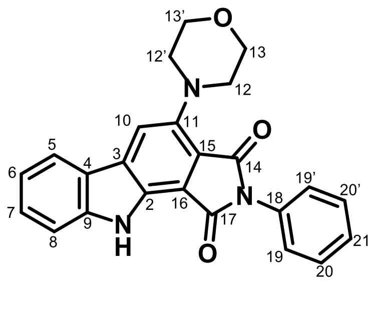

To a stirred solution of relevant amide **25a** (0.1 mmol) and Vaska's complex (0.1 mol%) under nitrogen atmosphere in dry toluene (0.1 M) was added TMDS (0.3 mmol, 3 eq.) at room temperature. This resulted in a bubbling solution, which was left to stir for 5 min, before adding dienophile (0.105 mmol). This mixture was then stirred overnight, followed by addition of DDQ (0.11 mmol, 1.1 eq.) in toluene (0.1 M). The resulted mixture was heated to 80 °C for 3 h, concentrated, purified Purification *via* FCC (8 : 2 pentane/EtOAc) to afford cycloadduct **27a** as yellow solid (30.6 mg, 77.0 μmol, 77 %).

mp >270 °C

IR IR 3374 (broad NH), 3063, 2923, 1702 (2 * C=O), 1495, 1387.

^{1}H NMR ($CDCl_3$, 400 MHz) δ_H: 9.28 (br s, 1H, NH), 8.08 (dd, *J* = 7.9, 1.1 Hz, 1H, C^5H), 7.89 (s, 1H, C^{10}H), 7.58–7.50 (m, 2H, C^{19}H, $C^{19'}$H), 7.54–7.47 (m, 2H, C^{20}H, $C^{20'}$H), 7.50–7.43 (m, 1H, C^{21}H), 7.47–7.39 (m, 1H, C^7H), 7.35–7.24 (m, 2H. C^6H, C^8H), 4.03–3.97 (m, 4H, C^{13}H, $C^{13'}$H), 3.40–3.33 (m, 4H, C^{12}H, C^{12}H).

^{13}C NMR ($CDCl_3$, 101 MHz) $\delta_{C:}$ 168.1 (C^{17}), 167.3 (C^{14}), 144.6 (C^{11}), 142.8 (C^9), 132.4 (C^{16}), 132.1 (C^{18}), 130.5 (C^{15}), 129.4 (C^{19}, $C^{19'}$), 129.0 (C^{21}), 128.2 (C^7), 127.2 (C^{20}, $C^{20'}$), 121.7 (C^5), 121.4 (C^4), 120.7 (C^8), 117.1 (C^2), 114.7 (C^{10}), 114.0 (C^3), 111.9 (C^6), 67.3 (C^{13}, $C^{13'}$), 52.7 (C^{12}, $C^{12'}$).

HRMS (ES^+) exact mass calculated for $[M+Na]^+$ ($C_{24}H_{20}O_3N_3$) requires **m/z** 398.1499, found **m/z** 398.1497.

4-Morpholino-2-phenyl-10-tosylpyrrolo[3,4-*a*]carbazole-1,3(2*H*,10*H*)-dione (27b)

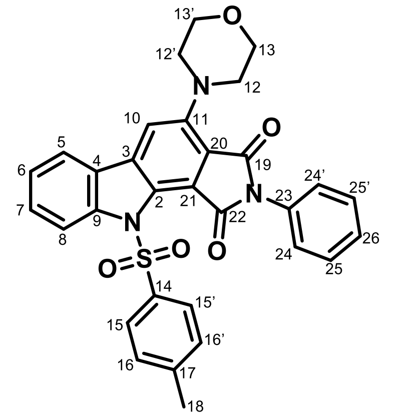

To a stirred solution of relevant amide **25b** (0.1 mmol) and Vaska's complex (0.1 mol%) under nitrogen atmosphere in dry toluene (0.1 M) was added TMDS (0.2 mmol, 2 eq.) at room temperature. This resulted in a bubbling solution, which was left to stir for 5 min, before adding dienophile (0.105 mmol). This mixture was then stirred overnight, followed by addition of DDQ (0.11 mmol, 1.1 eq.) in toluene (0.1 M). The resulted mixture was heated to 80 °C for 3 h, concentrated, purified Purification *via* FCC (8 : 2 pentane/EtOAc) to afford cycloadduct **27b** as brown solid (49.0 mg, 88.8 μmol, 88 %).

mp 258-259 °C

IR 2981, 2889, 1713 (C=O), 1598, 1380.

^{1}H NMR ($CDCl_3$, 400 MHz) δ_H: 7.91–7.81 (m, 2H, C^{5}H, C^{8}H), 7.71–7.66 (m, 2H, C^{15}H, C$^{15'}$H), 7.66 (s, 1H, C^{10}H), 7.53–7.44 (m, 5H, C^{7}H, C^{24}H, C$^{24'}$H, C^{25}H, C^{25}H), 7.44–7.32 (m, 2H, C^{6}H, C^{26}H), 7.19–7.12 (m, 2H, C^{16}H, C$^{16'}$H), 4.04–3.97 (m, 4H, C^{13}H, C$^{13'}$H), 3.43–3.36 (m, 4H, C^{12}H, C$^{12'}$H), 2.34 (s, 3H, C^{18}H).

^{13}C NMR ($CDCl_3$, 101 MHz) δ_C: 166.2 (C^{19}), 164.5 (C^{22}), 148.60 (C^{11}), 144.7 (C^{17}), 143.6 (C^{9}), 137.1 (C^{23}), 135.6 (C^{14}), 132.1 (C^{2}), 129.7 (C^{21}), 129.5 (C^{16}, C$^{16'}$), 129.1 (C^{25}, C$^{25'}$), 128.1 (C^{26}), 127.5 (C^{15}, C$^{15'}$), 127.2 (C^{24}, C$^{24'}$), 126.6 (C^{20}), 125.3 (C^{6}), 123.2 (C^{4}), 120.8 (C^{5}), 119.2 (C^{3}), 118.3 (C^{8}), 113.3 (C^{10}), 67.1 (C^{13}, C$^{13'}$), 52.5 (C^{12}, C$^{12'}$), 21.8 (C^{18}).

HRMS (ES^{+}) exact mass calculated for $[M+Na]^{+}$ ($C_{31}H_{26}O_5N_3{}^{32}S$) requires **m/z** 552.1588, found **m/z** 552.1585.

4,7-Bis(benzyl(methyl)amino)-2-phenyl-3a,4,7,7a-tetrahydro-1*H*-isoindole-1,3(2*H*)-dione (30)

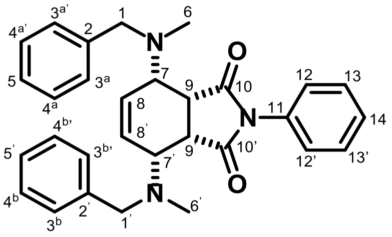

Prepared according to a modification of **General procedure E** (reaction time: 3 h) from **28** and *N*-phenyl maleimide. Purification *via* recrystallization in ether at 0 °C gave **30** as a brown solid (35.2 mg, 76%).

mp 138 °C

IR 3011, 1711 (2 * C=O), 1598, 1498, 1377.

^{1}H NMR (C_6D_6, 400 MHz) δ_H: 7.31–7.23 (m, 4H, C^{3a}H, C$^{3a'}$H, C^{3b}H, C$^{3b'}$H), 7.20–7.14 (m, 2H, C$^{12'}$H, C$^{12'}$H), 6.95 (dd, *J* = 8.2, 6.9 Hz, 4H, C^{4a}H, C$^{4a'}$H, C^{4b}H, C$^{4b'}$H), 6.88 (d, *J* = 6.1 Hz, 2H, C^{5}H, C$^{5'}$H), 6.85–6.82 (m, 2H, C^{13}H, C$^{13'}$H), 6.75–6.69 (m, 1H, C^{14}H), 5.57 (d, *J* = 1.3 Hz, 2H, C^{8}H, C$^{8'}$H), 3.64 (d, *J* = 13.3 Hz, 2H, C^{1}H, C$^{1'}$H), 3.22 (d, *J* = 13.4 Hz, 2H, C^{1}H, C$^{1'}$H), 2.61 (s, 4H, C^{7}H, C$^{7'}$H, C^{9}H, C$^{9'}$H), 2.01 (s, 6H, C^{6}H, C$^{6'}$H).

^{13}C NMR (C_6D_6, 101 MHz) δ_C: 173.9 (C^{10}, C$^{10'}$), 140.2 (C^2, C$^{2'}$), 133.3 (C^{11}), 131.0 (C^8, C$^{8'}$), 129.2 (C^{3a}, C$^{3a'}$, C^{3b}, C$^{3b'}$), 129.0 (C^5, C$^{5'}$), 128.7 (C^{4a}, C$^{4a'}$, C^{4b}, C$^{4b'}$), 128.2 (C^{14}), 127.4 (C^{13}, C$^{13'}$), 126.9 (C^{12}, C$^{12'}$), 61.7 (C^7, C$^{7'}$), 59.8 (C^1, C$^{1'}$), 42.8 (C^9, C$^{9'}$), 40.4 (C^6, C$^{6'}$).

9. Total Synthesis of catharanthine

1-(2-(1H-indol-3-yl)ethyl)-3,6-Dihydropyridin-2(1H)-one (S3) and 1-(2-(1H-indol-3-yl)ethyl)-5,6-dihydropyridin-2(1H)-one (S3')

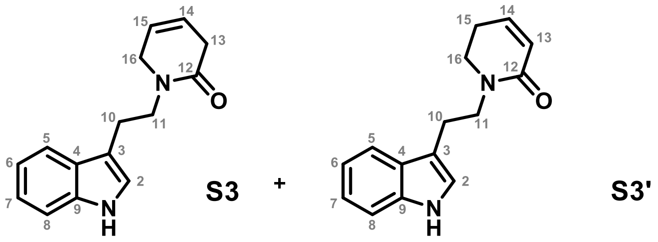

Prepared according to **General Procedure A** from tryptamine and dihydropyranone **34**. Purification *via* FCC (100% EtOAc) gave an inconsequential 2 : 1 mixture of constitutional isomers **S3** (**A**) and **S3'** (**B**), respectively, as an orange solid (1.23 g, 5.12 mmol, 51%). Further purification of the mixture was achieved by recrystallisation from EtOAc (25 mL/g of product, 78 °C to 0 °C).

^{1}H NMR ($CDCl_3$, 500 MHz) δ_H: 8.01 (br s, 1H, NH (**A**), NH (**B**)), 7.70–7.64 (m, 1H, C^5H (**A**), C^5H (**B**)), 7.37 (app d, 1H, *J* = 8.1 Hz, C^8H (**A**), C^8H (**B**)), 7.20 (app ddd, 1H, *J* = 8.1, 7.0, 1.2 Hz, C^7H (**A**), C^7H (**B**)), 7.13 (ddd, 1H, *J* = 8.1, 7.0, 1.1 Hz, C^6H (**A**), C^6H (**B**)), 7.07 (app d, 1H, *J* = 2.4 Hz, C^2H (**A**), C^2H (**B**)), 6.49 (dt, 0.33H, *J* = 9.7, 4.2 Hz, $C^{14}H$ (**B**)), 5.94 (dt, 0.33H, *J* = 9.8, 1.8 Hz, $C^{13}H$ (**B**)), 5.74 (dtd, 0.66H, *J* = 10.8, 3.4, 1.7 Hz, $C^{15}H$ (**A**)), 5.63 (dtd, 0.67H, *J* = 10.1, 3.1, 1.5 Hz, $C^{14}H$ (**A**)), 3.84 (tdd, 1.33H, *J* = 5.0, 3.1, 2.1 Hz, $C^{16}H_2$ (**A**)), 3.73 (td, 2H, *J* = 7.4, 1.5 Hz, $C^{11}H_2$ (**A**), $C^{11}H_2$ (**B**)), 3.27 (t, 0.67H, $C^{16}H_2$ (**B**)), 3.08 (app ddt, 2H, 7.8, 6.9, 2.2 Hz, $C^{10}H_2$ (**A**), $C^{10}H_2$ (**B**)), 2.99 (tdd, 1.33H, *J* = 5.1, 3.4, 1.9 Hz, $C^{13}H_2$ (**A**)), 2.24–2.12 (m, 0.66H, $C^{15}H_2$ (**B**)).

^{13}C NMR ($CDCl_3$, 101 MHz) δ_C: 167.3 (C^{12} (**A**)), 164.6 (C^{12}(**B**)), 139.5 (C^{14}(**B**)), 136.5 (C^4(**A**), C^4(**B**)), 127.51 (C^9(**B**)), 127.47 (C^9(**A**)), 125.6 (C^{13}(**B**)), 122.6 (C^{14} (**A**)), 122.3 (C^2(**B**)), 122.3 (C^2(**A**)), 122.0 (C^7(**A**)), 121.9 (C^7(**B**)), 121.0 (C^{15} (**A**)), 119.3 (C^7(**A**)), 119.2 (C^7(**B**)), 118.72 (C^5(**B**)), 118.69 (C^5(**A**)), 113.0 (C^3(**B**)), 112.8 (C^3(**A**)), 111.4 (C^8 (**A**), C^8 (**B**)), 49.6 (C^{16}(**A**)), 48.0 (C^{11}(**B**)), 47.9 (C^{11}(**A**)), 46.1 (C^{16}(**B**)), 32.4 (C^{13}(**A**)), 24.2 (C^{15}(**B**)), 23.8 (C^{10}(**B**)), 23.0 (C^{10}(**A**)).

1-(2-(1H-indol-3-yl)ethyl)-3-Ethyl-3,6-dihydropyridin-2(1H)-one (35)

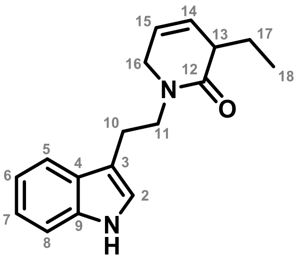

Prepared according to **General Procedure B** from a 2 : 1 mixture of **S3** and **S3'** and EtI. Purification *via* FCC (1 : 1 pentane/EtOAc) gave **35** as a white solid (1.09 g, 4.01 mmol, 84%).

mp 102–104 °C

IR 3266 (br), 2963, 2925, 1618 (C=O, lactam), 1496, 1457, 1437, 1344, 1233.

^{1}H NMR ($CDCl_3$, 500 MHz) δ_H: 8.11 (s, 1H, NH), 7.69 (app dq, 1H, *J* = 7.8, 0.9 Hz, C^5H), 7.36 (dt, 1H, *J* = 8.1, 1.0 Hz, C^8H), 7.19 (ddd, 1H, *J* = 8.1, 7.0, 1.3 Hz, C^7H), 7.12 (ddd, 1H, *J* = 8.0, 7.0, 1.1 Hz, C^6H), 7.02 (br d, 1H, *J* = 2.2 Hz, C^2H), 5.70 (2 * d, 2H, *J* = 11.2 Hz, $C^{14}H$, $C^{15}H$), 3.91–3.74 (m, $C^{11}Ha$, $C^{16}H_2$), 3.66 (dt, 1H, *J* = 13.2, 7.4 Hz, $C^{11}Hb$), 3.11–3.04 (m, 2H, $C^{10}H_2$), 3.01–2.94 (m, 1H, *J* = 6.7, 3.3, 1.5 Hz, $C^{13}H$), 1.91 (app tt, 1H, *J* = 13.9, 7.3 Hz, $C^{17}Ha$), 1.73 (dqd, 1H, *J* = 13.4, 7.5, 4.3 Hz, $C^{17}Hb$), 0.88 (t, 3H, *J* = 7.5 Hz, $C^{18}H_3$).

^{13}C NMR ($CDCl_3$, 101 MHz) δ_C: 170.2 (C^{12}), 136.4 (C^4), 127.5 (C^9), 127.1 (C^{14}), 122.2 (C^7), 122.0 (C^{15}), 121.1 (C^2), 119.4 (C^6), 118.8 (C^5), 113.0 (C^3), 111.3 (C^8), 49.5 (C^{16}), 48.0 (C^{11}), 42.4 (C^{13}), 26.3 (C^{17}), 23.1 (C^{10}), 10.1 (C^{18}).

HRMS (ES^+) exact mass calculated for $[M+H]^+$ ($C_{17}H_{20}ON_2^+$) requires **m/z** 291.1468, found **m/z** 291.1468.

Data in toluene for NMR tube experiment:

^{1}H NMR (C_7D_8, 400 MHz) δ_H: 7.74–7.71 (m, 1H, C^5H), 7.35 (br s, 1H, NH), 7.25–7.16 (m, 2H, C^6H, C^7H), 7.16–7.14 (m, 1H, C^8H), 6.66 (d, 1H, *J* = 2.3 Hz, C^2H), 5.37 (ddt, 1H, *J* = 10.1, 3.8, 2.0 Hz,

$C^{14}H$), 5.24 (dtd, 1H, *J* = 10.1, 3.1, 1.6 Hz, $C^{15}H$), 3.79 (ddd, 1H, *J* = 13.1, 8.4, 6.8 Hz, $C^{11}Ha$), 3.48 (ddd, 1H, *J* = 13.1, 8.1, 6.8 Hz, $C^{11}Hb$), 3.34 (ddt, 1H, *J* = 17.6, 4.3, 2.6 Hz, $C^{16}Ha$), 3.22 (dddd, 1H, *J* = 17.6, 4.5, 3.4, 1.9 Hz, $C^{16}Hb$), 3.00 (dddd, 1H, *J* = 7.6, 6.7, 2.4, 0.9 Hz, $C^{10}H_2$), 2.87 (tq, 1H, *J* = 6.0, 4.3, 3.6 Hz, $C^{13}H$), 2.04 (dqd, 1H, *J* = 13.7, 7.4, 6.3 Hz, $C^{17}Ha$), 1.66 (dqd, 1H, *J* = 13.3, 7.5, 4.4 Hz, $C^{17}Hb$), 0.88 (t, *J* = 7.4 Hz, 3H, $C^{18}H_3$).

Methyl 2-bromo-2-(diethoxyphosphoryl)acetate (36)[9]

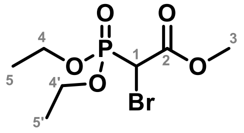

In a modification of a literature procedure,[9] to a solution of freshly distilled methyl diethylphosphonoacetate (5.25 g, 25 mmol) in anhydrous DME (70 mL) was added *n*-BuLi (2.5 M in hexanes, 10.2 mL, 1.05 eq.) at 0 °C. The resulting solution was stirred for 5 minutes, before Br_2 (1.35 mL, 1.05 eq.) was added. The resulting solution was allowed to warm to rt, and stirred for a further 2 hours. It was then carefully quenched with water (50 mL), and CH_2Cl_2 (60 mL) was added. Layers were separated, and extraction with CH_2Cl_2 (2 x 60 mL), followed by drying of the combined organics with Na_2SO_4 and concentration in vacuo gave the crude **36**. Purification *via* FCC (3 : 2 to 2 : 1 Pentane/EtOAc) gave **36** (5.02 g, 17.4 mmol, 70%) as a colorless free-flowing oil.

^{1}H NMR ($CDCl_3$, 400 MHz) δ_H: 4.36 (d, 1H, *J* = 14.2 Hz, C^1H), 4.30–4.19 (m, 4H, C^4H_2, $C^{4'}H_2$), 3.81 (s, C^3H_3), 1.35 (td, 6H, *J* = 7.1 Hz, 0.7 Hz, C^5H_3, $C^{5'}H_3$).

^{13}C NMR ($CDCl_3$, 101 MHz) δ_C: 165.7 (C^2), 64.8 (2 x d, J = 6.5 Hz, C^4, $C^{4'}$), 53.9 (C^3), 35.5 (d, *J* = 146.3 Hz, C^1), 16.4 (d, *J* = 5.9 Hz, C^5).

^{31}P NMR ($CDCl^3$, 162 MHz) δ_P: 12.4.

NMR spectra and physical properties matched those reported in literature.[9]

Methyl 2-(diethoxyphosphoryl)-2-(3-(2-(3-ethyl-2-oxo-3,6-dihydropyridin-1(2H)-yl)ethyl)-1H-indol-2-yl)acetate (37)

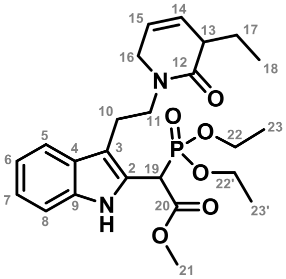

A vial was charged with phosphono-ester **36** (26.1 mg, 0.0900 mmol), lactam **35** (26.8 mg, 0.100 mmol), PhN(PMP)$_2$ (5.5 mg, 0.020 mmol), and Ir(ppy)$_3$ (0.9 mg, 0.001 mmol). The vial was flushed with Argon and DCE (0.5 mL) was introduced. The vial was then placed in a photobox (Hepatochem EvoluChem™ PhotoRedOx Duo, equipped with two EvoluChem™ LED 18W lights) and irradiated while stirred and cooled with a ventilator to *ca.* 30 °C for 14 h. It was then directly loaded on a FCC and eluted with EtOAc to give C^2-functionalised **37** as an inconsequential 54 (**A**) : 46 (**B**) mixture of diastereoisomers and as a yellow oil (25.6 mg, 54%).

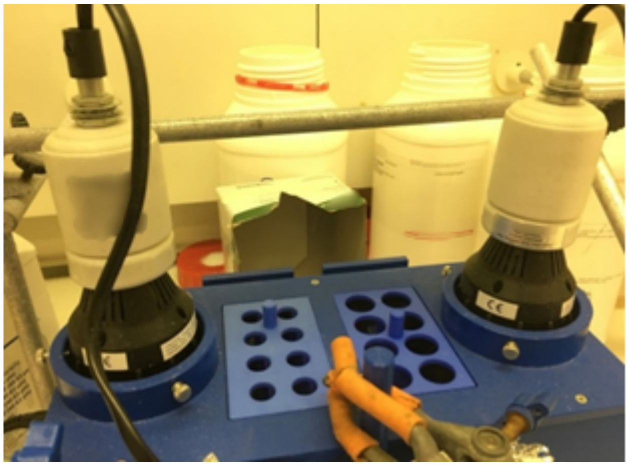
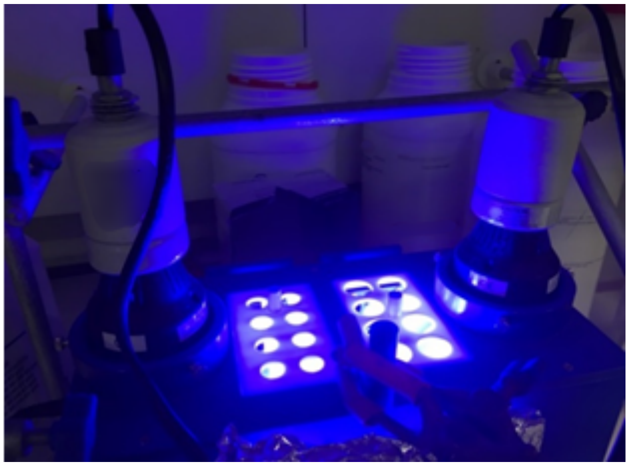

^{1}H NMR (CDCl$_3$, 500 MHz) δ_H: 9.20 (s, 0.46H, NH (**B**)), 9.19 (s, 0.54H, NH (**A**)), 7.72–7.66 (app t, 1H, J = 8.8 Hz, C^{5}H (**A**), C^{5}H (**B**)), 7.39–7.35 (app ddt, 1H, J = 8.2, 2.0, 0.9 Hz, C^{8}H (**A**), C^{8}H (**B**)),

7.21–7.16 (m, 1H, C^7H (**A**), C^7H (**B**)), 7.13–7.08 (m, 1H, C^6H (**A**), C^6H (**B**)), 5.76–5.65 (m, 2H, $C^{14}H$ (**A**), $C^{14}H$ (**B**), $C^{15}H$ (**A**), $C^{15}H$ (**B**)), 4.73 (d, 0.54H, *J* = 23.8 Hz, $C^{19}H$ (**A**)), 4.72 (d, 0.46H, *J* = 23.7 Hz, $C^{19}H$ (**B**)), 4.21–4.07 (m, 2H, $C^{22}H_2$ (**A**), $C^{22}H_2$ (**B**)), 4.07–3.81 (m, 3.46H, $C^{16}H_2$ (**A**), $C^{16}Ha$ (**B**), $C^{22'}H_2$ (**A**), $C^{22'}H_2$ (**B**)), 3.78 (s, 1.38H, $C^{21}H_3$ (**B**)), 3.77 (s, 1.62H, $C^{21}H_3$ (**A**)), 3.75–3.67 (m, 1H, $C^{16}Hb$ (**B**), $C^{11}Ha$ (**A**)), 3.62 (ddd, 0.54H, *J* = 13.2, 9.6, 6.3 Hz, $C^{11}Hb$ (**A**)), 3.56 (ddd, 0.46H, *J* = 13.2, 9.6, 6.3 Hz, $C^{11}Ha$ (**B**)), 3.46 (ddd, 0.46H, *J* = 13.2, 9.5, 6.8 Hz, $C^{11}Hb$ (**B**)), 3.12–2.95 (m, 3H, $C^{10}H_2$ (**A**), $C^{10}H_2$ (**B**), $C^{13}H$ (**A**), $C^{13}H$ (**B**)), 1.93 (pd, 0.54H, *J* = 7.3, 4.0 Hz, $C^{17}Ha$ (**A**)), 1.90 (pd, 0.46H, *J* = 7.3, 4.0 Hz, $C^{17}Ha$ (**B**)), 1.81–1.69 (m, 1H, $C^{17}Hb$ (**A**), $C^{17}Hb$ (**B**)), 1.32 (t, 1.62H, *J* = 7.1 Hz, $C^{23}H_3$ (**A**)), 1.31 (t, 1.38H, *J* = 7.1 Hz, $C^{23}H_3$ (**B**)), 1.15 (t, 1.38H, *J* = 7.1 Hz, $C^{23'}H_3$ (**B**)), 1.14 (t, 1.62H, *J* = 7.1 Hz, $C^{23'}H_3$ (**A**)), 0.90 (t, 1.38H, *J* = 7.5 Hz, $C^{18}H_3$ (**B**)), 0.89 (t, 1.62H, *J* = 7.5 Hz, $C^{18}H_3$ (**A**)).

^{13}C NMR ($CDCl_3$, 126 MHz) δ_C: 170.24 (C^{12} (**A**)), 170.20 (C^{12} (**B**)), 167.6–167.7 (m, C^{20} (**A**), C^{20} (**B**)), 135.92 (C^4 (**A**)), 135.88 (C^4 (**B**)), 127.6 (d, *J* = 2.4 Hz, (C^9 (**B**)), 127.5 (d, *J* = 2.4 Hz, (C^9 (**A**)), 127.3 (C^{14} (**B**)), 127.2 (C^{14} (**A**)), 123.7 (d, *J* = 11.4 Hz, C^2 (**A**)), 123.5 (d, *J* = 11.1 Hz, C^2 (**B**)), 122.6 (C^7 (**A**), C^7 (**B**)), 121.11 (C^{15} (**A**)), 121.07 (C^{15} (**B**)), 119.70 (C^6 (**B**)), 119.67 (C^6 (**A**)), 119.0 (C^5 (**B**)), 118.9 (C^5 (**A**)), 112.09 (d, *J* = 10.0 Hz, (C^3 (**B**)), 112.03 (d, *J* = 9.9 Hz, (C^3 (**A**)), 111.42 (C^8 (**A**)), 111.38 (C^8 (**B**)), 63.8–63.9 (m, C^{22} (**A**), C^{22} (**B**), $C^{22'}$ (**A**), $C^{22'}$ (**B**)), 53.30 (C^{21} (**B**)), 53.28 (C^{21} (**A**)), 50.0 (C^{16} (**A**)), 49.7 (C^{16} (**B**)), 48.4 (d, *J* = 2.8 Hz, C^{11} (**A**)), 48.3 (d, *J* = 2.8 Hz, C^{11} (**B**)), 43.5 (d, *J* = 133.6 Hz, C^{19} (**B**)), 43.4 (d, *J* = 132.9 Hz, C^{19} (**A**)), 42.43 (C^{13} (**B**)), 42.41 (C^{13} (**A**)), 26.3 (C^{17} (**A**)), 26.2 (C^{17} (**B**)), 22.0 (C^{10} (**A**), C^{10} (**B**)), 16.5 (d, *J* = 5.9 Hz, (C^{23} (**A**), C^{23} (**B**)), 16.41 (d, *J* = 5.8 Hz, $C^{23'}$ (**B**)), 16.38 (d, *J* = 5.8 Hz, $C^{23'}$ (**A**)), 10.2 (C^{18} (**B**)), 10.1 (C^{18} (**A**)).

^{31}P NMR ($CDCl_3$, 162 MHz) δ_P: 17.51 (**B**), 17.38 (**A**).

HRMS (ES^+) exact mass calculated for $[M+H]^+$ ($C_{24}H_{34}O_6N_2P^+$) requires **m/z** 477.21490, found **m/z** 477.21484.

Methyl 2-(diethoxyphosphoryl)-2-(3-(2-(3-ethyl-2-oxopyridin-1(2H)-yl)ethyl)-1H-indol-2-yl)acetate (37')

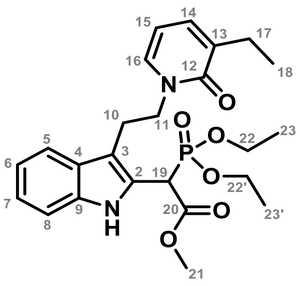

Original procedure to give analytical quantities of the product:
A 10 ml vial was charged with **35** (26.8 mg, 100 μmol), Ru(bpy$_3$).6H_2O (0.8 mg, 1 μmol), PhN(PMP)$_2$ (55.0 mg, 200 μmol), **36** (28.9 mg, 100 μmol), and DMF (1 ml). The vial was then irradiated for 14 h under the Blue LED "loop" system (see pictures below). The solution was then diluted with EtOAc (5 ml), and extracted with 5% (w/w) LiCl aqueous solution (5 × 3 mL). The organic layer was then concentrated *in vacuo* and purification *via* FCC gave a mixture of desired product **37** and byproduct pyridone **37'**. Further purification of the mixture *via* reverse-phase HPLC (1 : 2 MeCN/H_2O) afforded **37** as an inconsequential mixture of two diastereomers and as a yellow oil (1.8 mg, 4%), and **37'** as a yellow oil (1.0 mg, 2%).

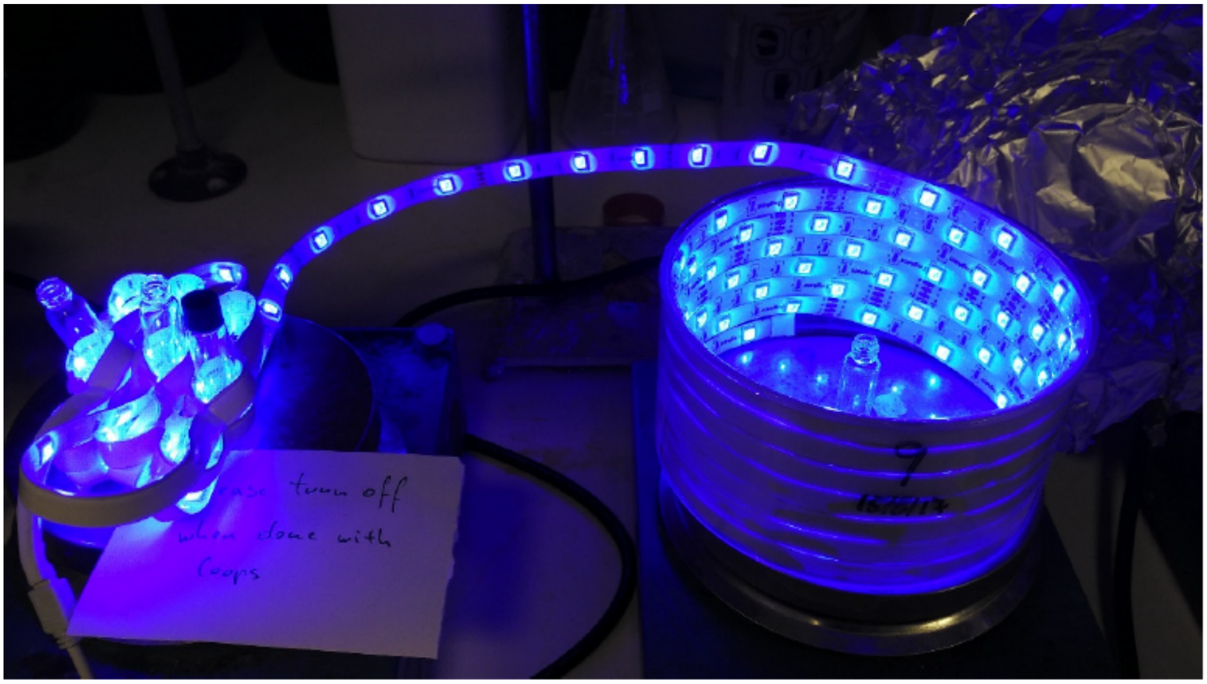

^{1}H NMR ($CDCl_3$, 400 MHz) δ_H: 9.20 (s, 1H, NH), 7.64 (dt, 1H, *J* = 7.8, 1.0 Hz, $C^{5}H$), 7.37 (dt, 1H, *J* = 8.0, 0.9 Hz, $C^{8}H$), 7.20 (ddt, 1H, *J* = 8.1, 7.0, 1.1 Hz, $C^{7}H$), 7.15–7.07 (m, 2H, $C^{6}H$, $C^{16}H$), 6.89 (dd, 1H, *J* = 6.8, 2.0 Hz, $C^{14}H$), 5.93 (t, 1H, *J* = 6.8 Hz, $C^{15}H$), 4.60 (d, 1H, *J* = 24.0 Hz, $C^{19}H$), 4.32 (ddd, 1H, *J* = 12.6, 8.5, 5.2 Hz, $C^{11}H$a), 4.18–3.96 (m, 4H, $C^{11}H$b, $C^{22}H_2$, $C^{22'}H$a), 3.94–3.79 (m, 1H, $C^{22'}H$b), 3.75 (d, 3H, *J* = 0.5 Hz, $C^{21}H_3$), 3.34–3.19 (m, 1H, $C^{10}H$a), 3.19–3.06 (m, 1H, $C^{10}H$b), 2.68–2.54 (m, 2H, $C^{17}H_2$), 1.30 (td, 3H, *J* = 7.1, 0.6 Hz, $C^{23}H_3$), 1.23 (t, 3H, *J* = 7.5 Hz, $C^{23'}H_3$), 1.16 (td, 3H, *J* = 7.0, 0.6 Hz, $C^{18}H_3$).

^{13}C NMR ($CDCl_3$, 126 MHz) δ_C: 167.6 (C^{12}), 162.9 (C^{20}), 135.9 (C^{4}), 135.4 (C^{14}), 135.2 (C^{9}), 134.9 (C^{16}), 124.0 (d, *J* = 11.5 Hz, C^{2}), 122.7 (C^{7}), 119.9 (C^{6}), 118.8 (C^{5}), 111.4 (C^{8}), 105.3 (C^{15}), 64.0- 63.8 (m, C^{22}, $C^{22'}$), 52.7 (C^{21}), 51.0 (d, *J* = 2.8 Hz, C^{11}), 43.6 (d, *J* = 132.4 Hz, C^{19}), 23.7 (app s, C^{10}, C^{17}), 16.7 (m, C^{23}, $C^{23'}$) 12.9 (C^{18}).

Note : C^{3} was not observed.

^{31}P NMR ($CDCl^{3}$, 162 MHz) δ_P: 17.1.

HRMS (ES+) exact mass calculated for $[M+H]^+$ ($C_{24}H_{31}O_6N_2NaP$) requires **m/z** 497.1811, found **m/z** 497.1809

Methyl 2-(3-(2-(3-ethyl-2-oxo-3,6-dihydropyridin-1(2H)-yl)ethyl)-1H-indol-2-yl)acrylate (38)

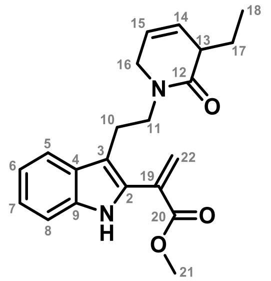

A round-bottom flask was charged with phosphonate **37** (26.3 mg, 0.055 mmol), paraformaldehyde (16.8 mg, 0.55 mmol), LiBr (5.8 mg, 0.066 mmol), and THF (1.1 mL). NEt_3 (8.0 µL, 0.055 mmol) was added dropwise, and the resulting suspension was stirred at rt for 30 min. The reaction mixture was quickly concentrated at 0 °C, and loaded on a cold column in the minimum quantity of CH_2Cl_2. Elution with cold 5 : 1 Toluene/EtOAc, followed by concentration at 0 °C (product must not be kept neat more than 5 minutes or decomposition will happen) gave **38** as a yellow oil (15.9 mg, 45.1 µmol, 81%). It was characterized immediately in deuterated toluene and used in the next 3 h.

IR 3257 (br), 2963, 1723 (C=O, ester), 1623 (C=O, lactam), 1496, 1457, 1437, 1343, 1286.

^{1}H NMR (C_7D_8, 500 MHz) δ_H: 9.19 (s, 1H, NH), 7.73–7.67 (m, 1H, C^5H), 7.19–7.13 (m, 1H, C^7H, C^6H), 7.03–7.00 (m, 1H, C^8H), 6.56 (s, 1H, $C^{22}Ha$), 6.52 (s, 1H, $C^{22}Hb$), 5.33 (ddt, 1H, J = 10.2, 3.7, 2.0 Hz, $C^{14}H$), 5.24 (dtd, 1H, J = 10.1, 3.0, 1.6 Hz, $C^{15}H$), 3.58–3.41 (m, 2H, $C^{11}H_2$), 3.38–3.34 (m, 4H, $C^{16}Ha$, $C^{21}H_3$), 3.28 (dddd, 1H, J = 17.5, 4.9, 3.3, 1.9 Hz, $C^{16}Hb$), 3.18–3.01 (m, 2H, $C^{10}H_2$), 2.83–2.76 (m, 1H, $C^{13}H$), 2.05–1.92 (m, 1H, $C^{17}Ha$), 1.66–1.55 (m, 1H, $C^{17}Hb$), 0.84 (t, 3H, *J* = 7.4 Hz, $C^{18}H_3$).

^{13}C NMR (C_7D_8, 126 MHz) δ_C: 168.9 (C^{12}), 167.5 (C^{20}), 135.9 (C^9), 131.0 (C^{19}), 130.2 (C^2), 128.4 (C^{22}), 127.6 (C^{14}), 123.2 (C^7), 121.4 (C^{15}), 119.9 (C^6), 119.2 (C^5), 112.6 (C^3), 111.7 (C^8), 51.8 (C^{21}), 49.3 (C^{16}), 47.9 (C^{11}), 42.5 (C^{13}), 26.4 (C^{17}), 23.2 (C^{10}), 10.2 (C^{18}).

Note: C^4 is overlapping with some of the deuterated toluene peaks.

HRMS (ES^+) exact mass calculated for $[M+H]^+$ ($C_{21}H_{25}O_3N_2$) requires **m/z** 353.1860, found **m/z** 353.1859.

Catharanthine (3) [10]

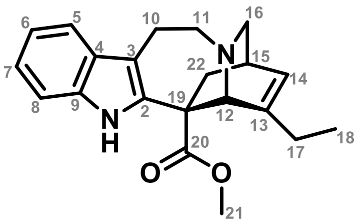

An NMR tube was charged with **38** (5.6 mg, 0.016 mmol), deuterated toluene (0.5 mL), and Vaska's complex (0.1 mg, 0.00016 mmol, 1 mol%). A solution of TMDS (8.5 μL, 0.048 mmol) in deuterated toluene (0.25 mL) was added dropwise over 20 min with the help of a syringe pump. Mild bubbling was observed. The reaction was monitored by NMR. After 2 h at rt, the resulting solution was directly loaded on a preparative TLC, and elution with 100% EtOAc gave catharanthine **3** (0.6 mg, 1.8 μmol, 11%).

^{1}H NMR ($CDCl_3$, 500 MHz) δ_H: 7.61 (s, 1H, NH), 7.48 (d, 1H, J = 7.7 Hz, C^5H), 7.23 (app d, 1H, J = 8.1 Hz, C^8H), 7.14 (ddd, 1H, J = 8.1, 7.1, 1.3 Hz, C^7H), 7.10 (app td, 1H, J = 7.4, 7.0, 1.2 Hz, C^6H), 5.94–5.91 (m, 1H, $C^{14}H$), 4.17 (d, 1H, J = 1.5 Hz, $C^{12}H$), 3.73 (s, 3H, $C^{21}H_3$), 3.56 (ddd, 1H, J = 14.1, 10.5, 3.9 Hz, $C^{11}Ha$), 3.38 (dt, 1H, J = 13.8, 4.6 Hz, $C^{11}Hb$), 3.29 (ddd, 1H, J = 15.4, 10.5, 5.4 Hz, $C^{10}Ha$), 2.91 (dt, 1H, J = 16.5, 4.4 Hz, $C^{10}Hb$), 2.86 (br d, 1H, J = 8.6 Hz, $C^{16}Ha$), 2.83 (dt, 1H, J = 8.7, 2.7 Hz, $C^{16}Hb$), 2.75–2.69 (m, 2H, $C^{15}H$, $C^{22}Ha$), 2.31 (ddd, 1H, J = 17.1, 7.3, 2.2 Hz, $C^{17}Ha$), 2.11 (ddd, 1H, J = 17.2, 7.4, 1.8 Hz, $C^{17}Hb$), 1.78 (dd, 1H, J = 13.9, 3.3 Hz, $C^{22}Hb$), 1.06 (t, 3H, J = 7.3 Hz, $C^{18}H_3$).

^{13}C NMR ($CDCl_3$, 126 MHz) δ_C: 174.3 (C^{20}), 149.6 (C^{13}), 136.6 (C^2), 135.1 (C^9), 129.2 (C^4), 123.7 (C^{14}), 122.0 (C^7), 119.6 (C^6), 118.4 (C^5), 110.9 (C^3), 110.6 (C^8), 62.1 (C^{12}), 55.6 (C^{19}), 53.2 (C^{11}), 52.5 (C^{21}), 49.4 (C^{16}), 38.9 (C^{22}), 30.9 (C^{15}), 26.3 (C^{17}), 21.5 (C^{10}), 10.8 (C^{18}).

Data in toluene for NMR tube experiment: ^{1}H NMR (C_7D_8, 400 MHz) δ_H [selected peaks]: 5.76–5.72 (m, 1H, $C^{14}H$), 4.22 (d, 1H, *J* = 1.5 Hz, $C^{12}H$), 3.39 (ddd, 1H, *J* = 12.7, 9.4, 3.9 Hz, $C^{11}Ha$), 3.25 (s, 3H, $C^{21}H_3$), 3.16–3.00 (m, 2H), 2.83 (dt, 1H, *J* = 12.7, 3.3 Hz), 2.76 (dt, 1H, *J* = 8.3, 2.8 Hz), 2.73–2.66 (m, 1H), 2.60 (dd, 1H, *J* = 8.1 Hz, 1.5 Hz), 2.38–2.33 (m, 1H).

NMR spectra and physical properties matched those reported in literature.[10]

10. NMR Data

15b

^{1}H NMR (500 MHz, $CDCl_3$)

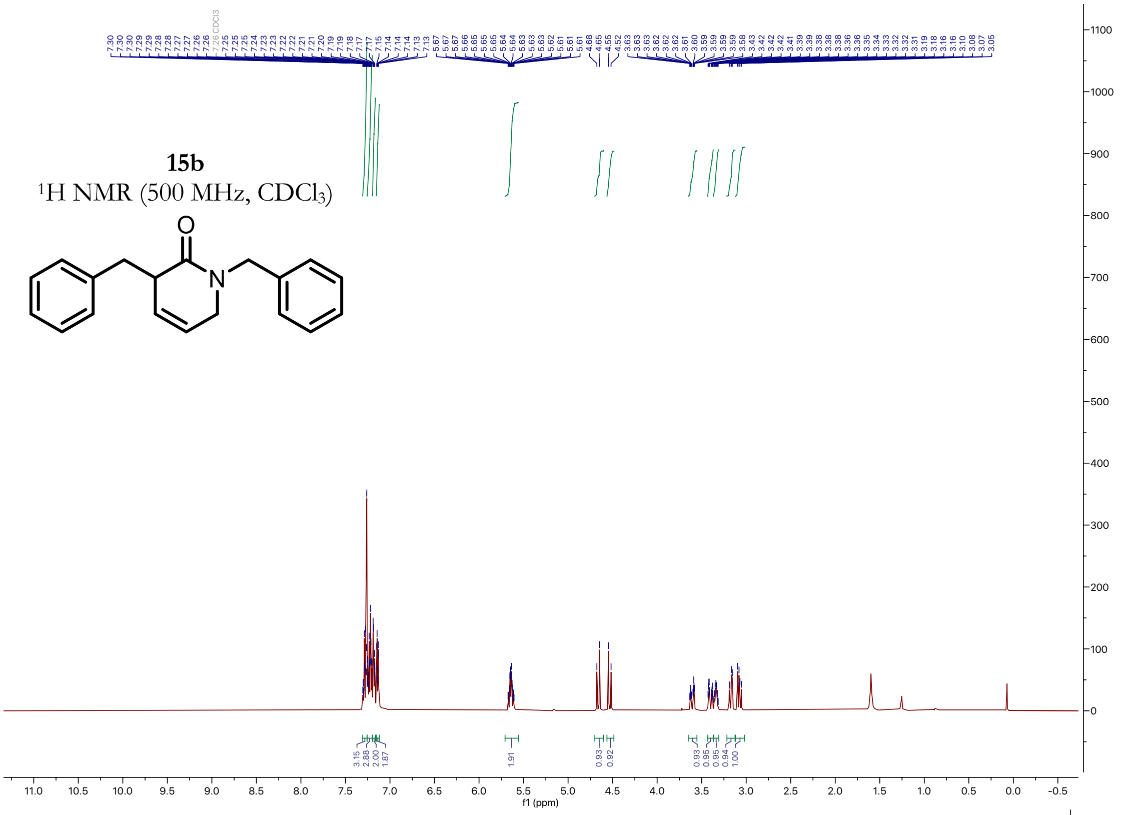

15b

^{13}C NMR (126 MHz, $CDCl_3$)

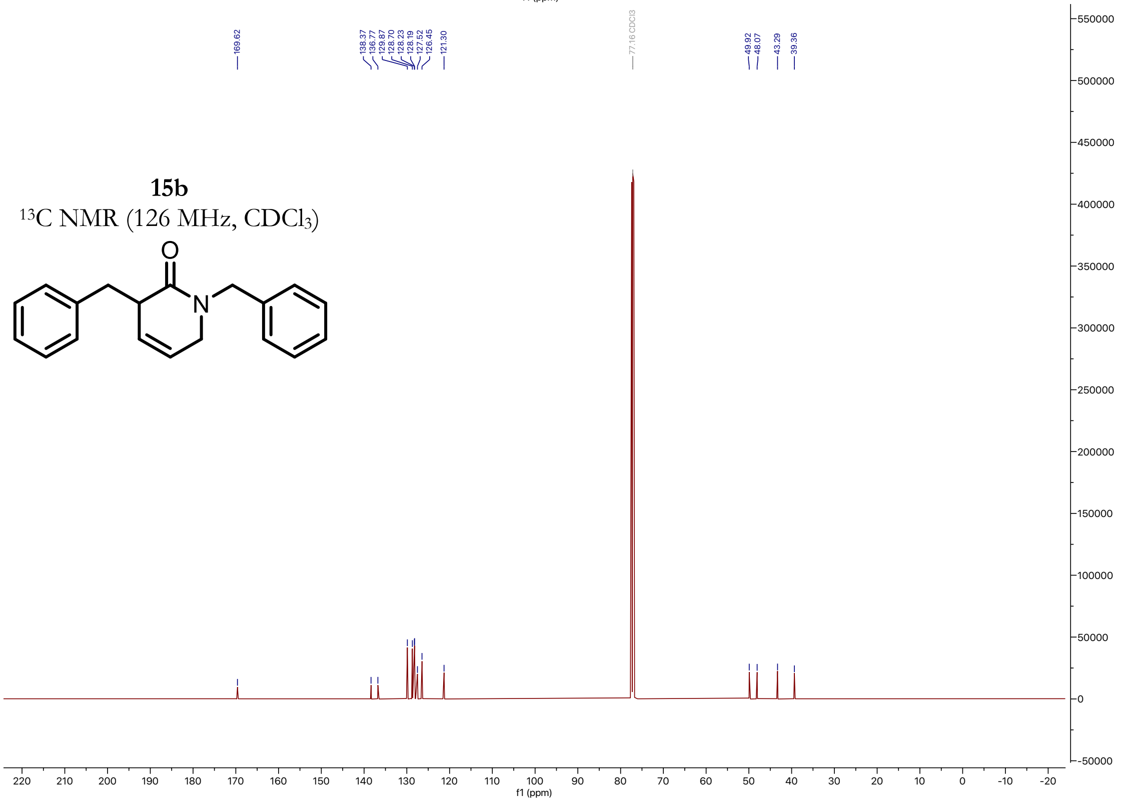

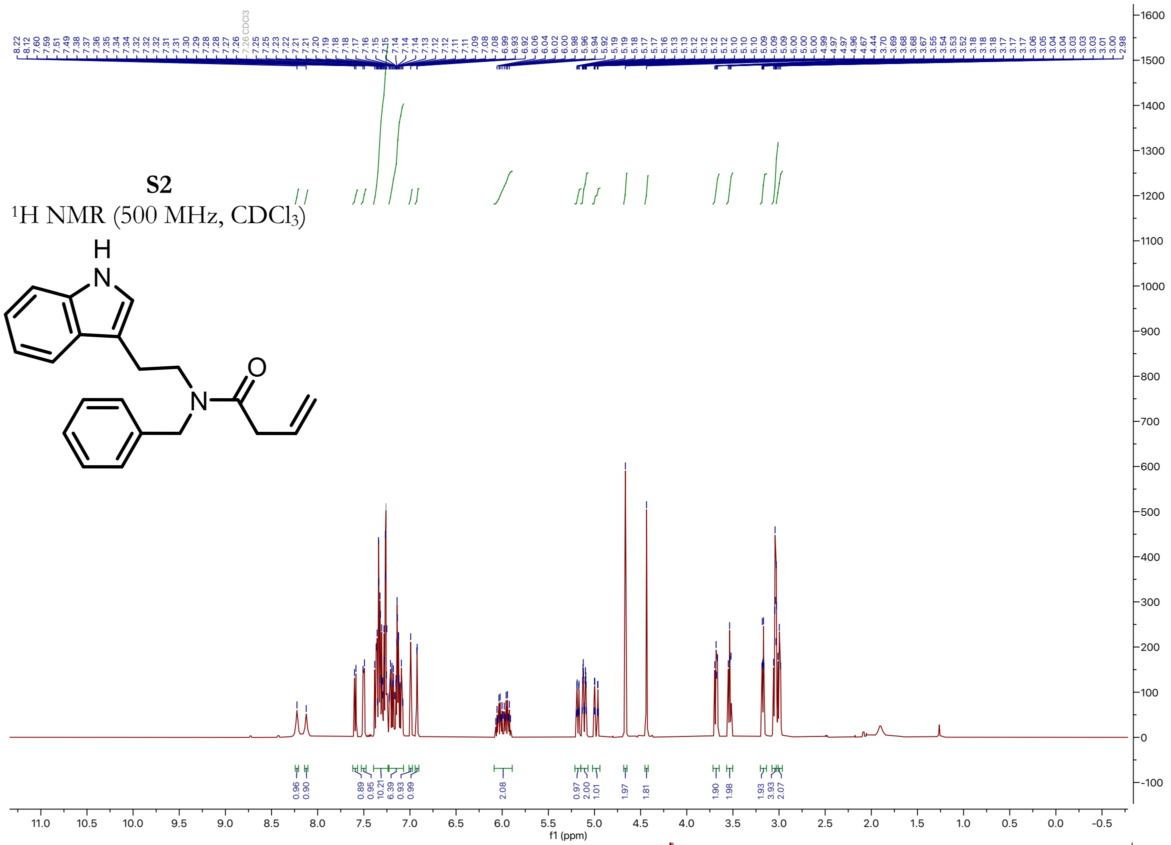
S2
¹H NMR (500 MHz, CDCl₃)
f1 (ppm)

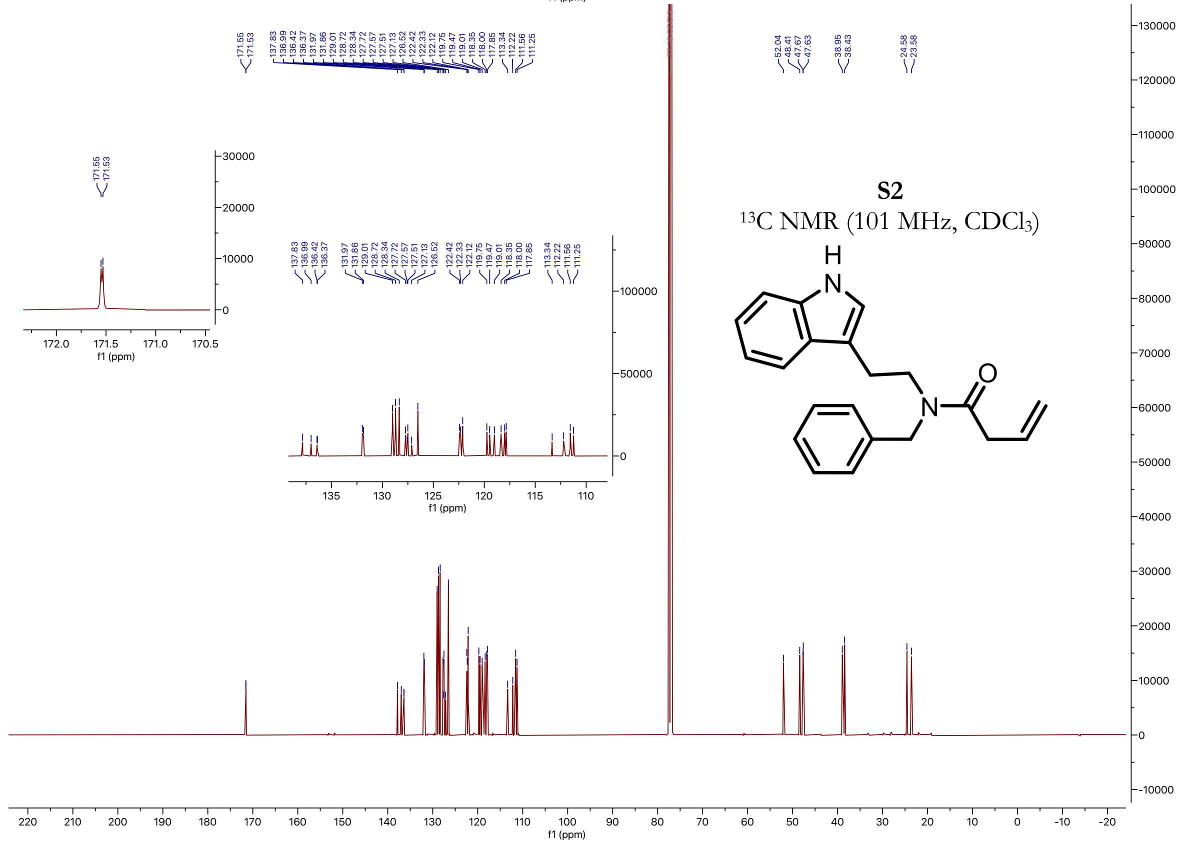
S2
¹³C NMR (101 MHz, CDCl₃)
f1 (ppm)

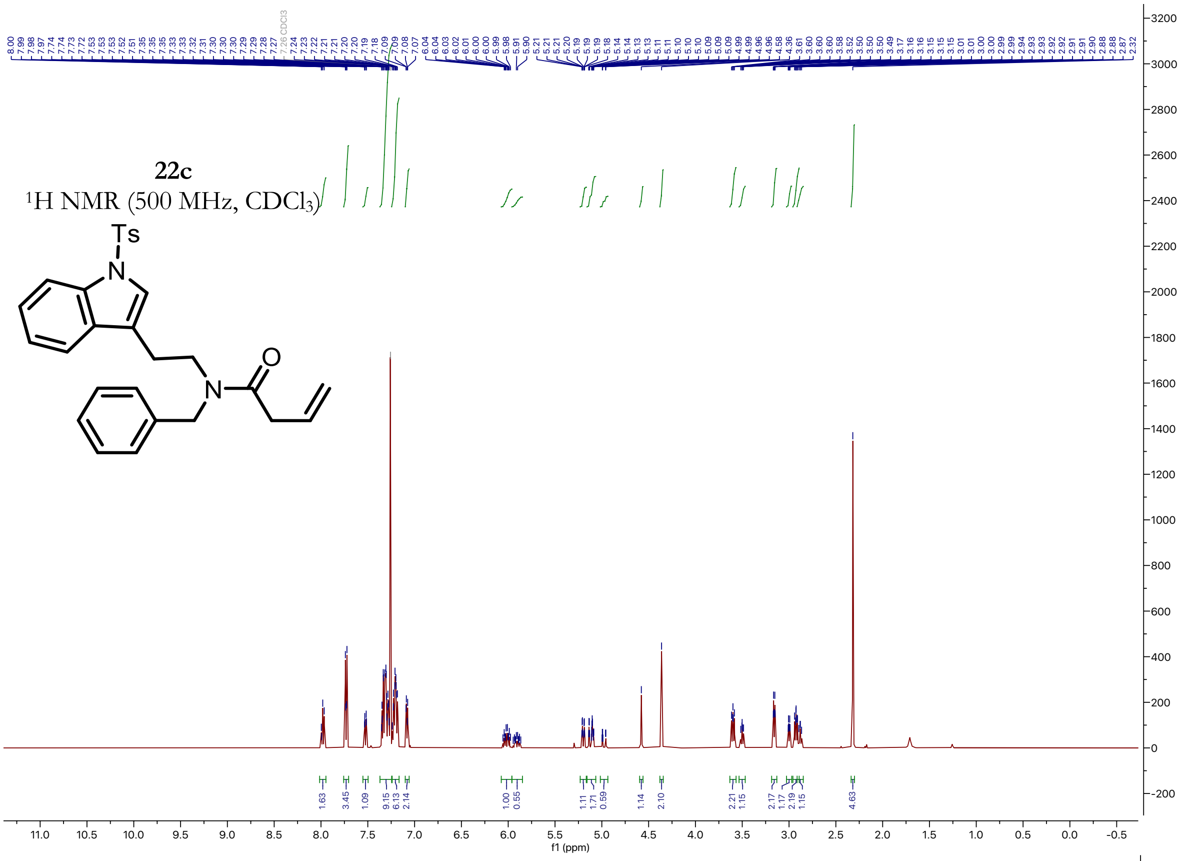
22c
1H NMR (500 MHz, CDCl3)
Ts
N
O
f1 (ppm)

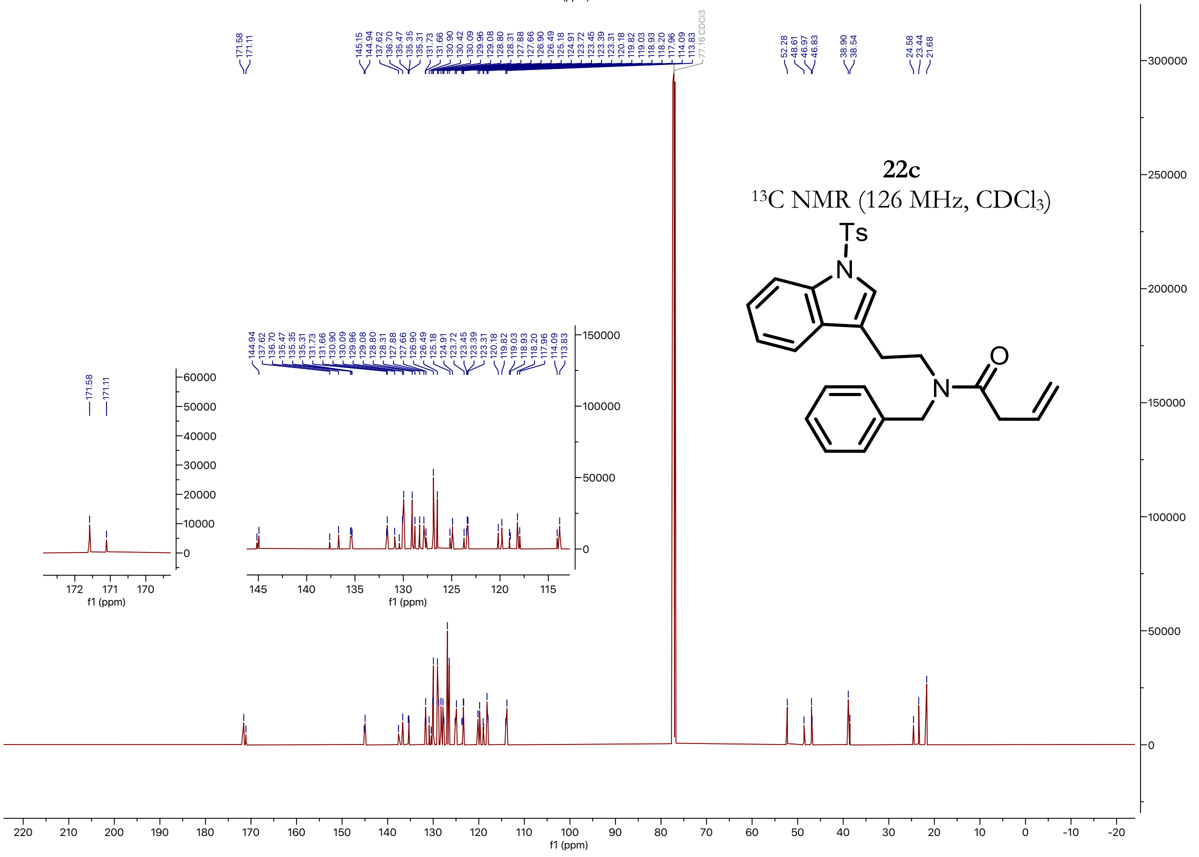
22c
13C NMR (126 MHz, CDCl3)
Ts
N
O
f1 (ppm)

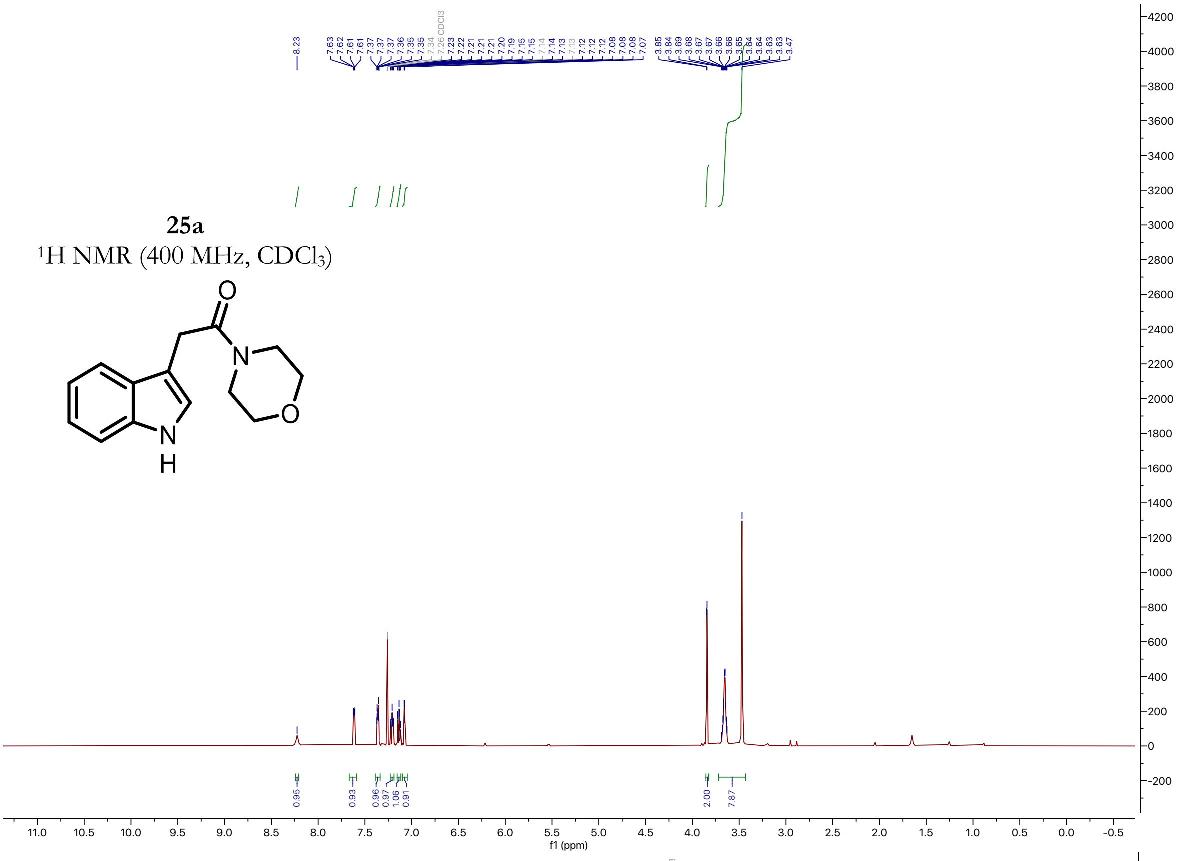

25a

^{1}H NMR (400 MHz, $CDCl_3$)

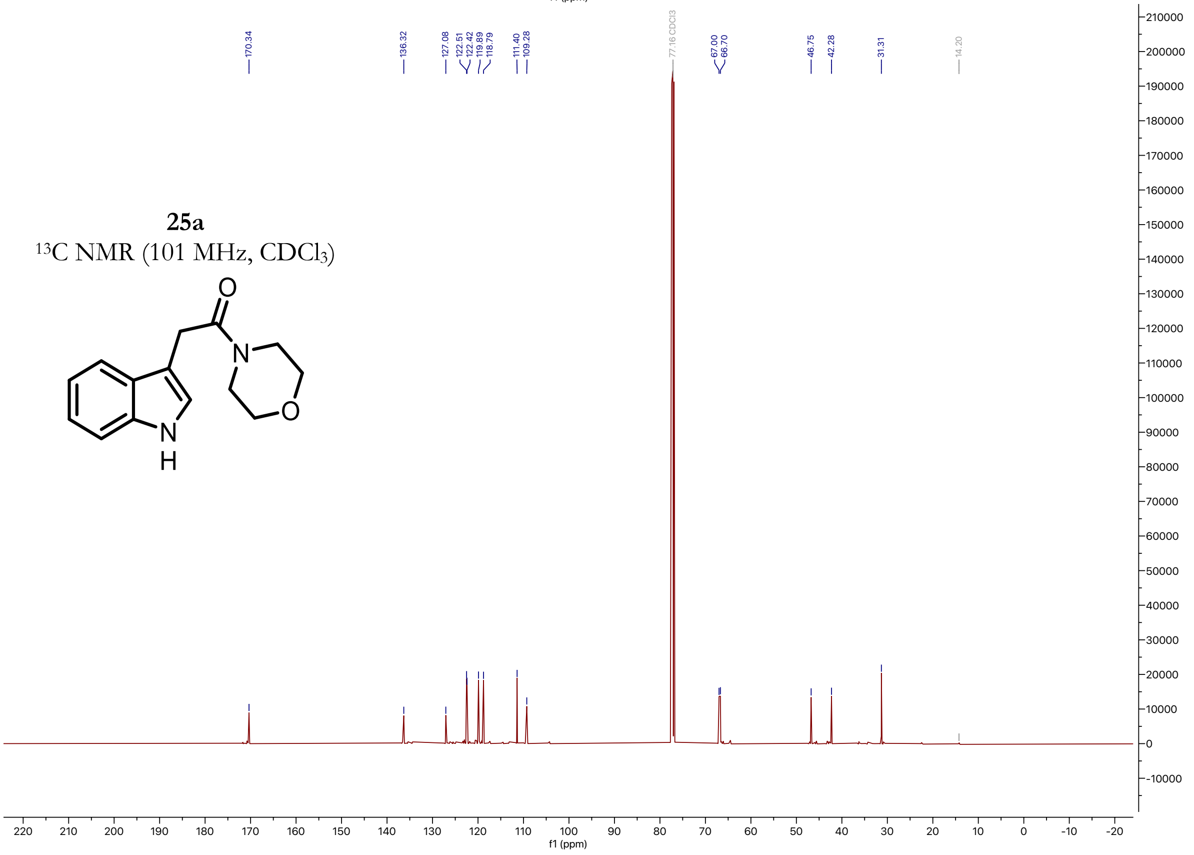

25a

^{13}C NMR (101 MHz, $CDCl_3$)

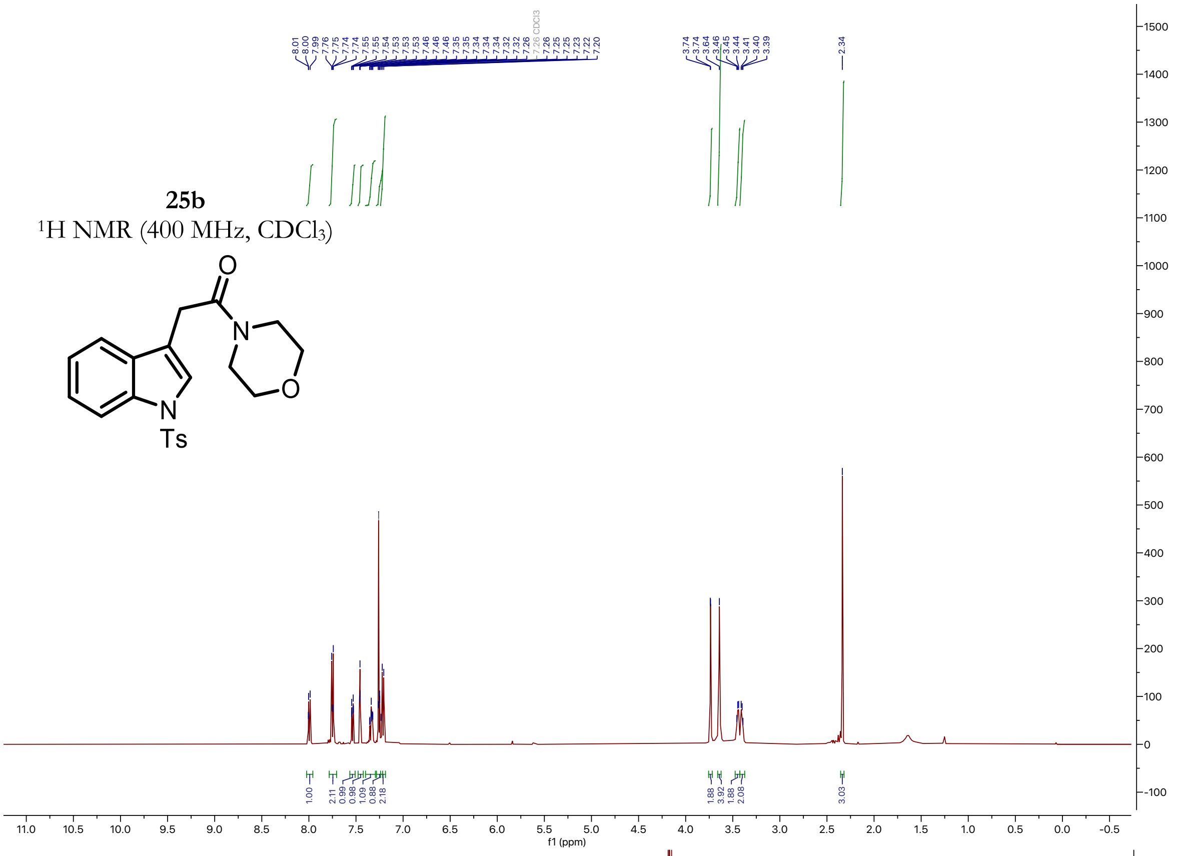
25b
¹H NMR (400 MHz, CDCl3)
Ts
8.01 8.00 7.99 7.76 7.75 7.74 7.74 7.55 7.55 7.54 7.53 7.53 7.53 7.46 7.46 7.46 7.35 7.35 7.34 7.34 7.34 7.32 7.32 7.26 CDCl3 7.26 7.25 7.25 7.23 7.22 7.20
3.74 3.74 3.64 3.46 3.45 3.44 3.41 3.40 3.39
2.34
1.00 2.11 0.99 0.98 1.09 0.88 2.18
1.88 3.92 1.88 2.08
3.03
f1 (ppm)

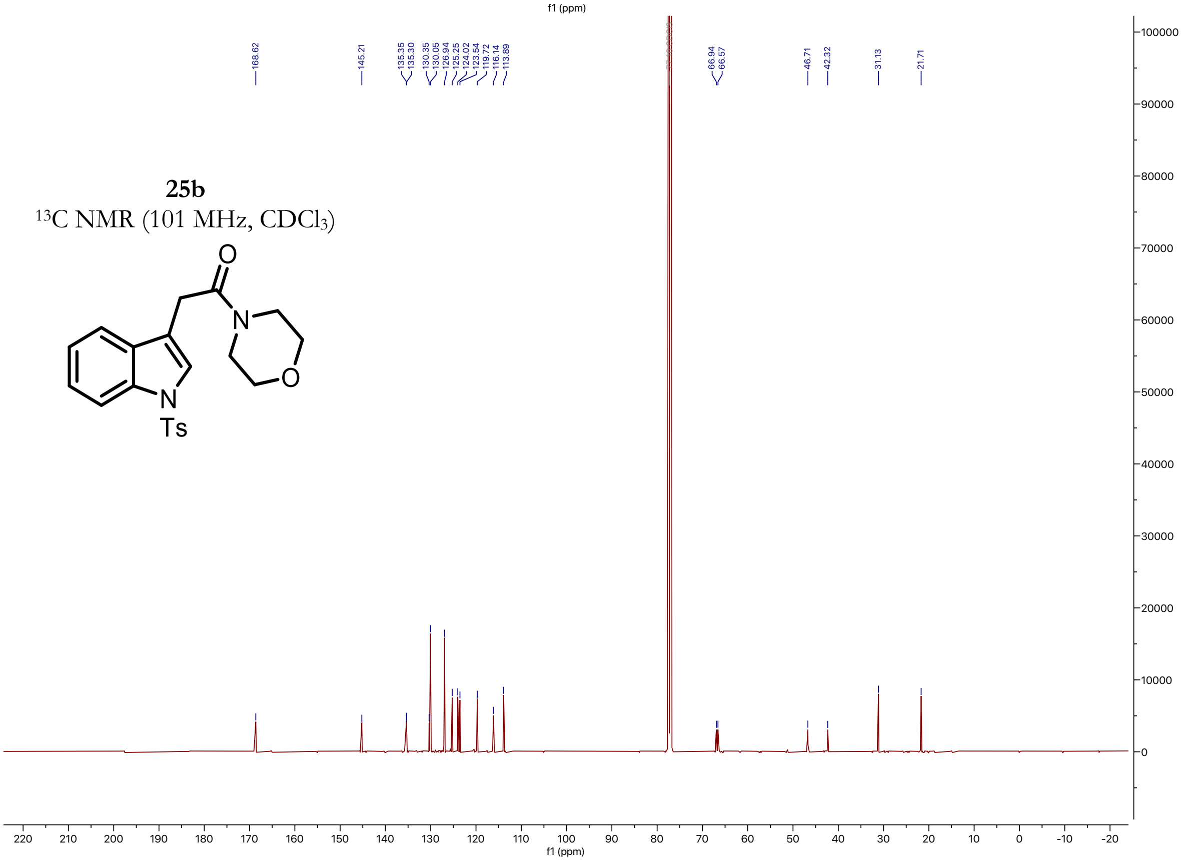
25b
¹³C NMR (101 MHz, CDCl3)
Ts
168.62
145.21
135.35 135.30 130.35 130.05 126.94 125.25 124.02 123.54 119.72 116.14 113.89
66.94 66.57
46.71
42.32
31.13
21.71
f1 (ppm)

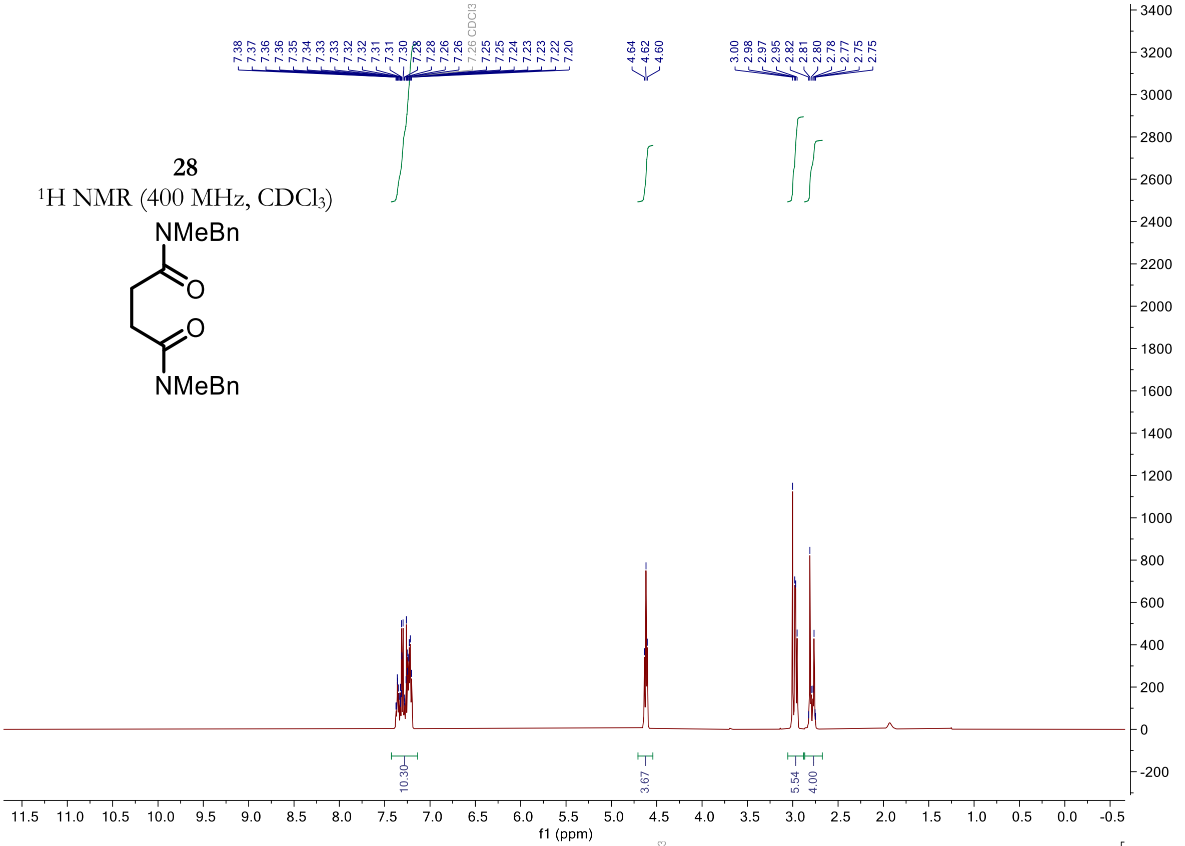
7.38 7.37 7.36 7.36 7.35 7.34 7.33 7.33 7.32 7.32 7.31 7.31 7.30 7.28 7.28 7.26 7.26 7.26 CDCl3 7.25 7.25 7.24 7.23 7.23 7.22 7.20
4.64 4.62 4.60
3.00 2.98 2.97 2.95 2.82 2.81 2.80 2.78 2.77 2.75 2.75
28
^{1}H NMR (400 MHz, CDCl$_3$)
NMeBn
O
O
NMeBn
10.30
3.67
5.54
4.00
f1 (ppm)

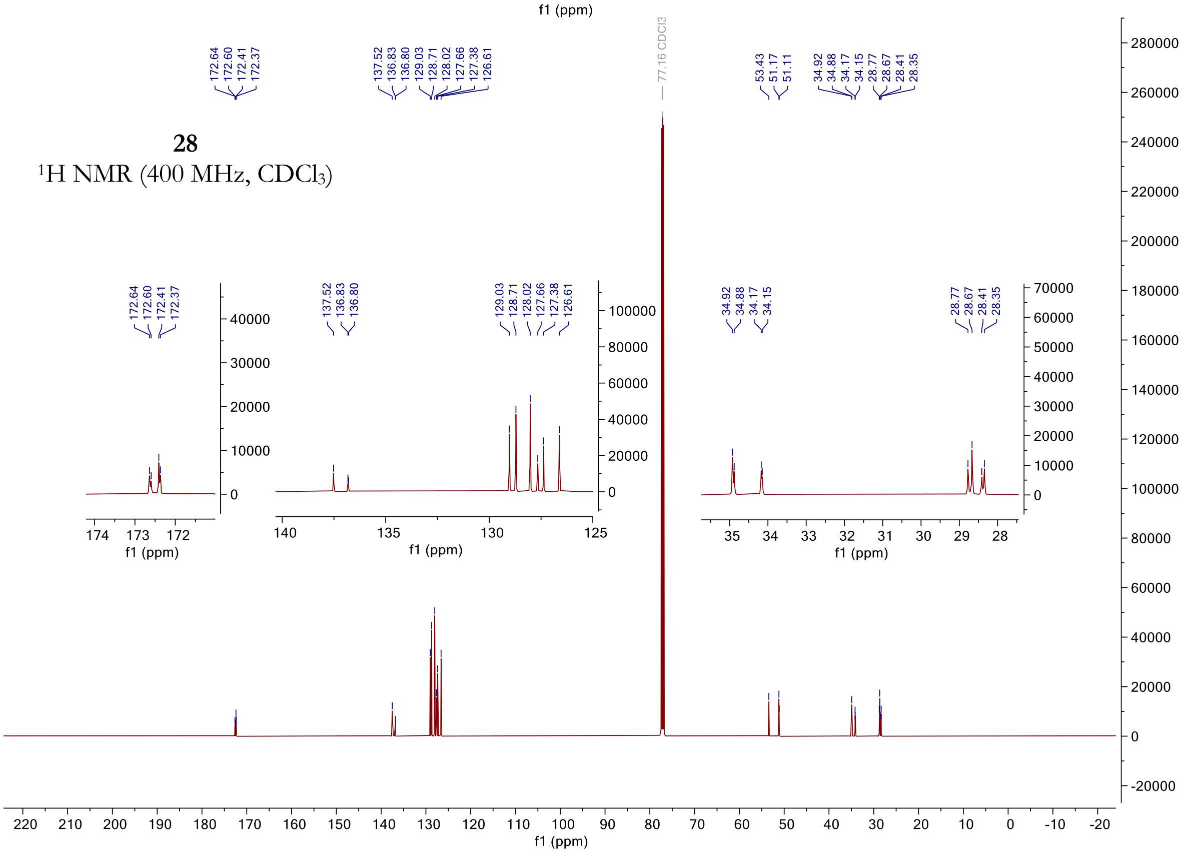
172.64 172.60 172.41 172.37
137.52 136.83 136.80 129.03 128.71 128.02 127.66 127.38 126.61
77.16 CDCl3
53.43 51.17 51.11
34.92 34.88 34.17 34.15 28.77 28.67 28.41 28.35
28
^{1}H NMR (400 MHz, CDCl$_3$)
f1 (ppm)

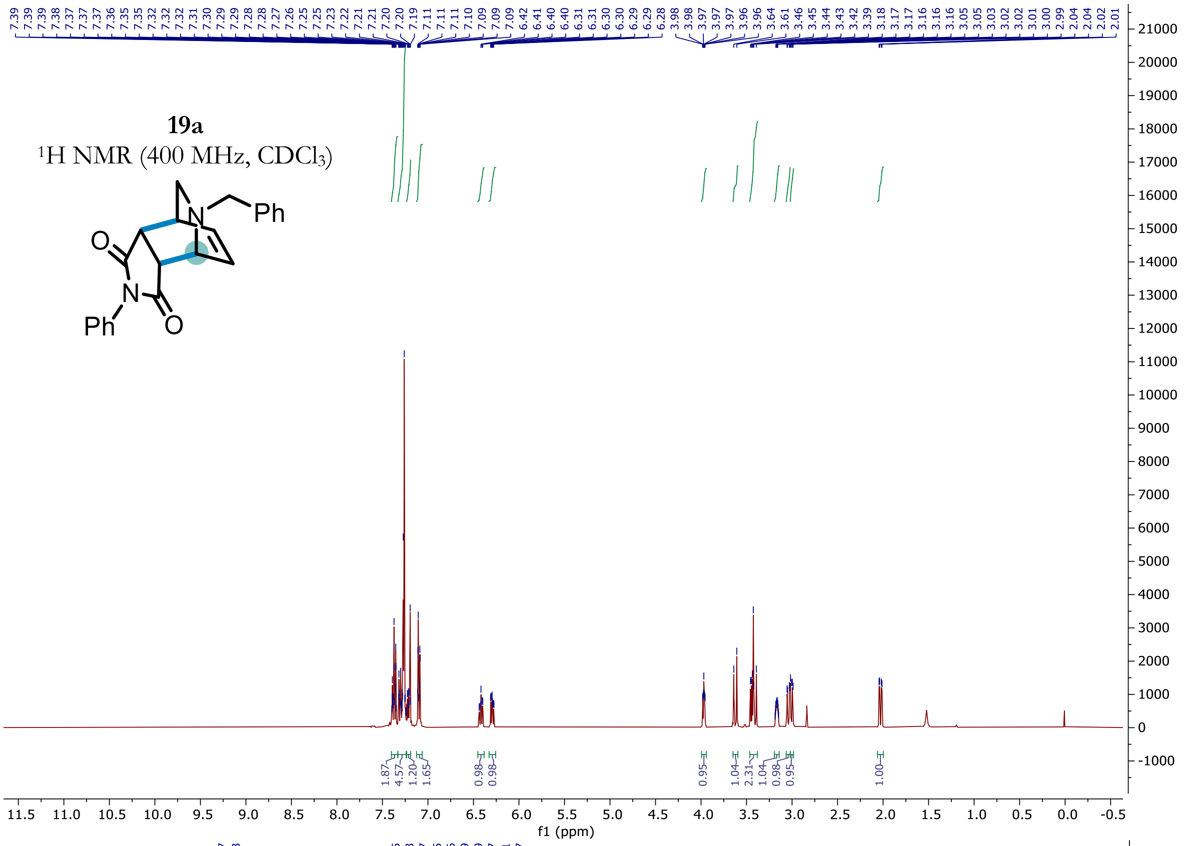
7.39 7.39 7.39 7.38 7.37 7.37 7.37 7.36 7.35 7.35 7.32 7.32 7.32 7.31 7.30 7.29 7.29 7.28 7.28 7.27 7.26 7.25 7.25 7.23 7.22 7.21 7.21 7.20 7.20 7.19 7.11 7.11 7.11 7.10 7.09 7.09 7.09 6.42 6.41 6.40 6.31 6.31 6.30 6.29 6.28 3.98 3.98 3.97 3.97 3.96 3.96 3.64 3.61 3.46 3.45 3.44 3.43 3.42 3.39 3.18 3.17 3.17 3.16 3.16 3.05 3.05 3.03 3.02 3.02 3.01 3.00 2.99 2.04 2.04 2.02 2.01
19a
1H NMR (400 MHz, CDCl3)
Ph
N
O
Ph
O
1.87 4.57 1.20 1.65 0.98 0.98 0.95 1.04 2.31 1.04 0.98 0.95 1.00
f1 (ppm)

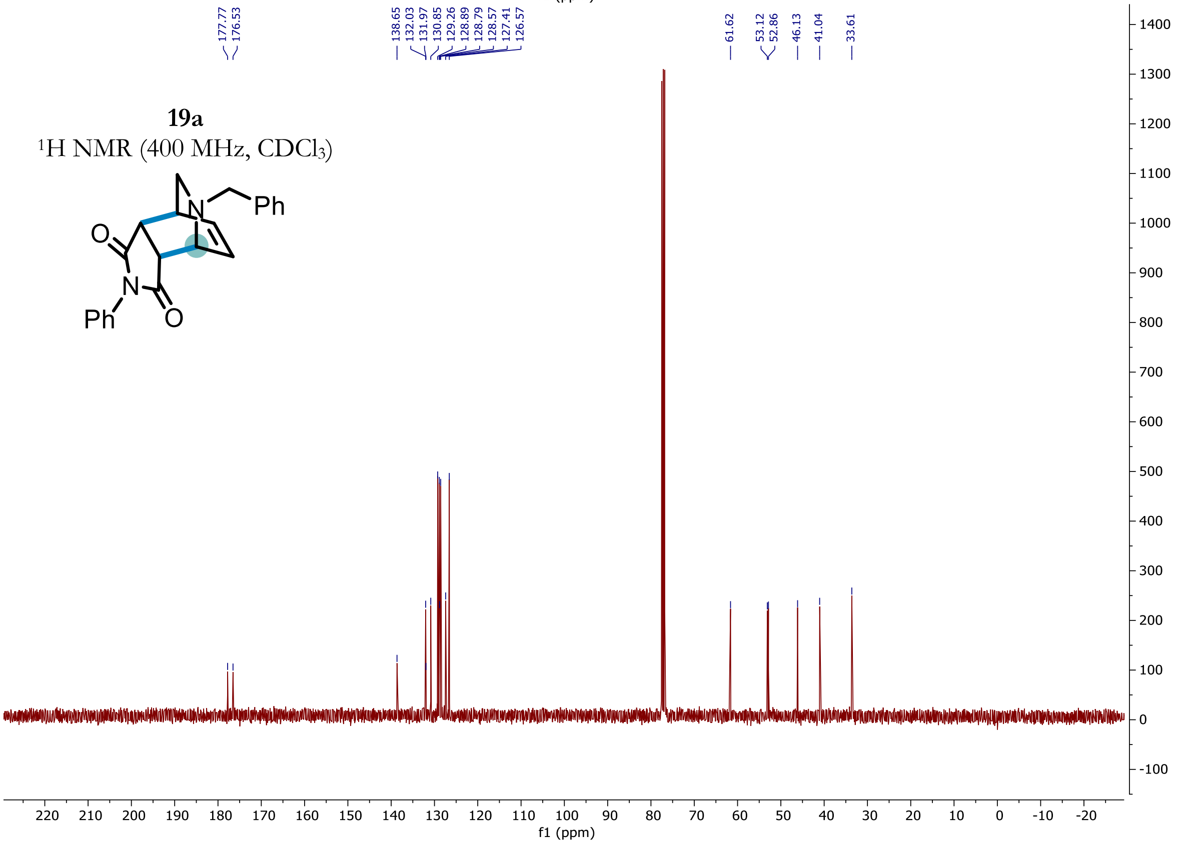
177.77 176.53 138.65 132.03 131.97 130.85 129.26 128.89 128.79 128.57 127.41 126.57 61.62 53.12 52.86 46.13 41.04 33.61
19a
1H NMR (400 MHz, CDCl3)
Ph
N
O
Ph
O
f1 (ppm)

19b

^{1}H NMR (400 MHz, CDCl$_3$)

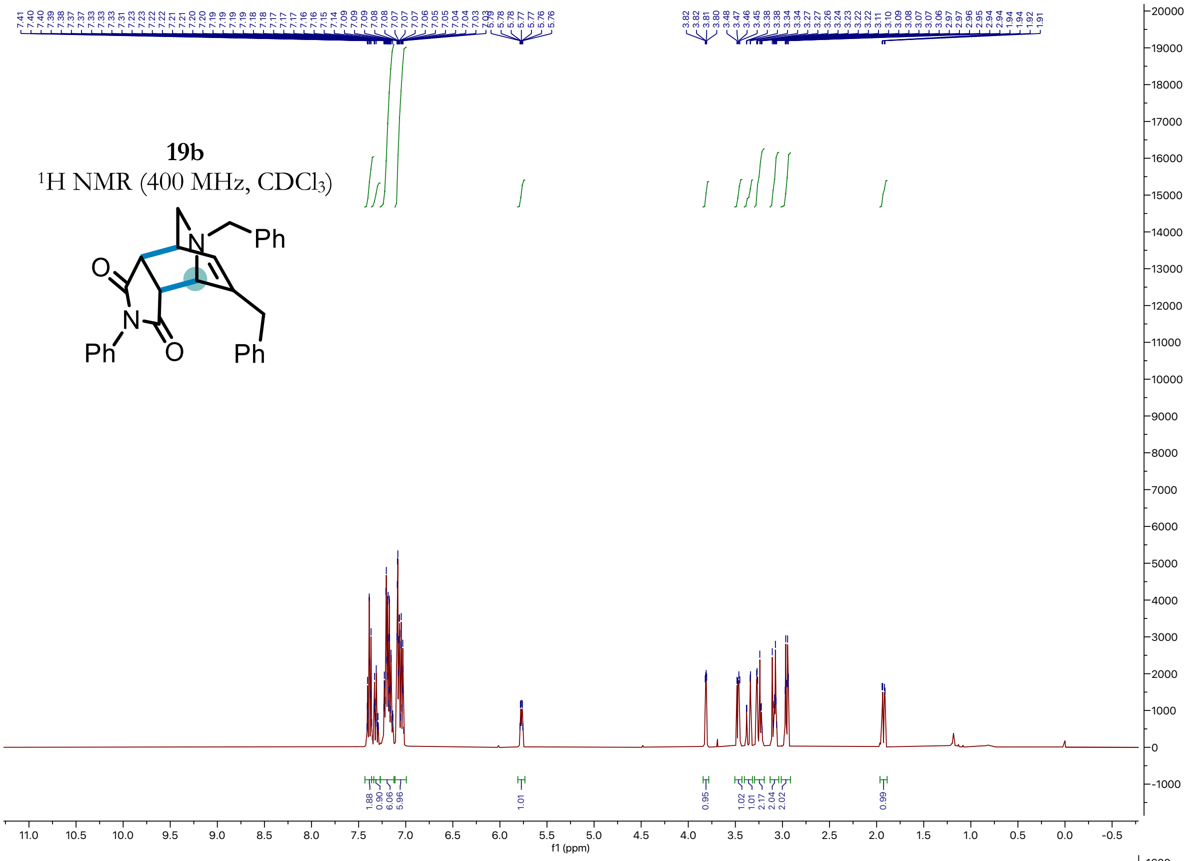

19b

^{13}C NMR (101 MHz, CDCl$_3$)

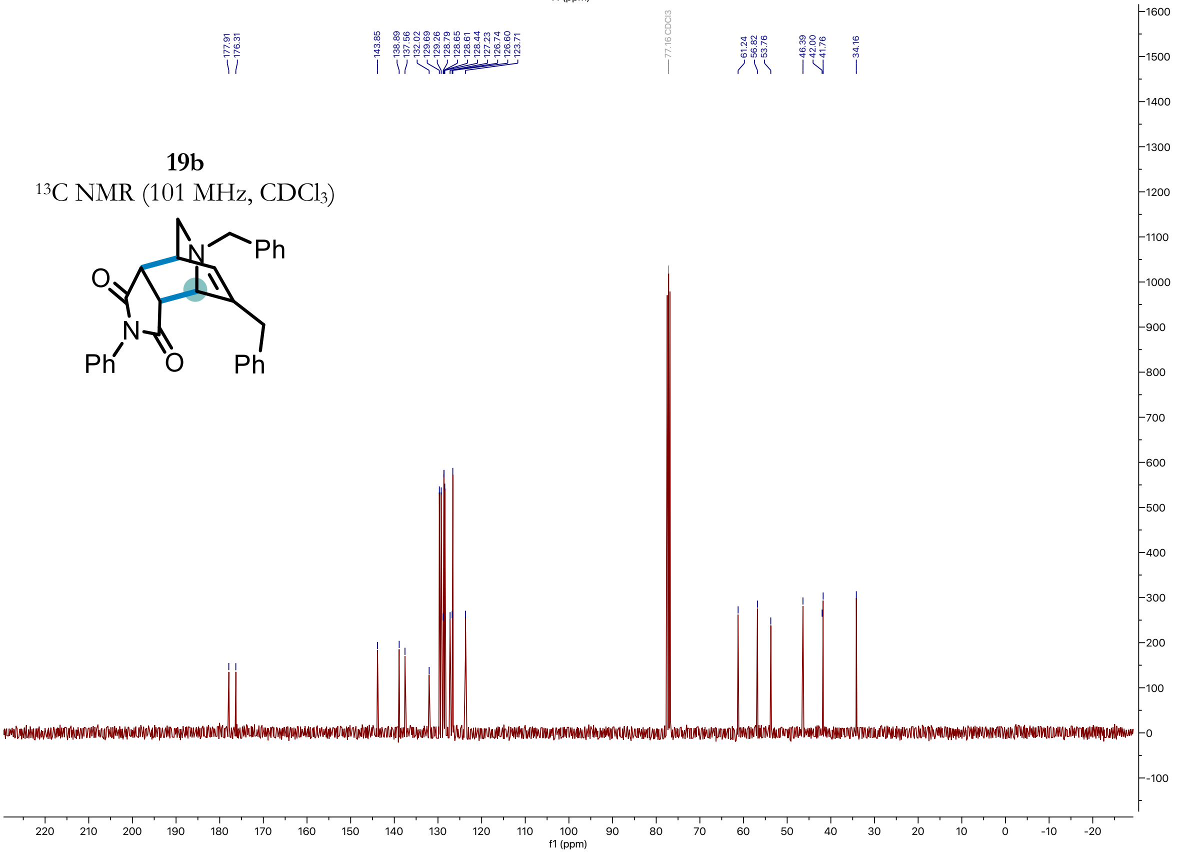

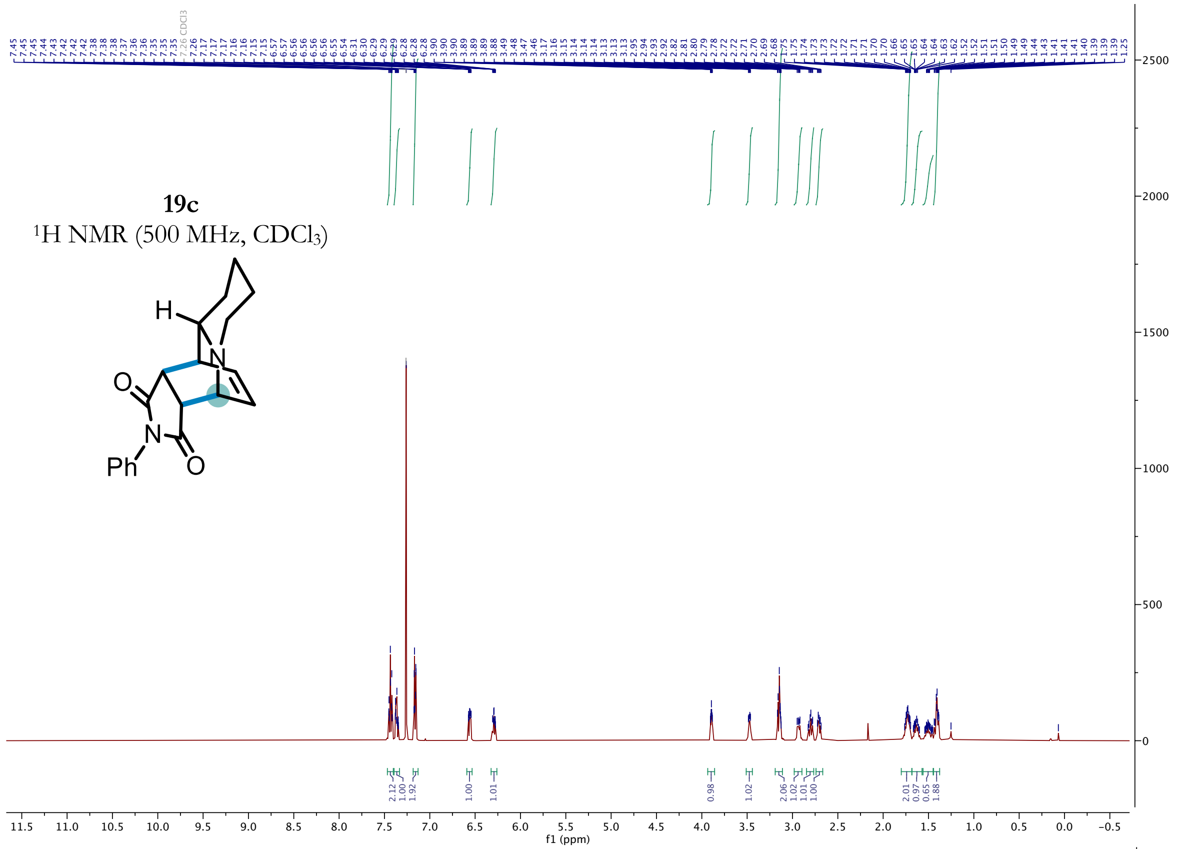
19c
¹H NMR (500 MHz, CDCl₃)
f1 (ppm)

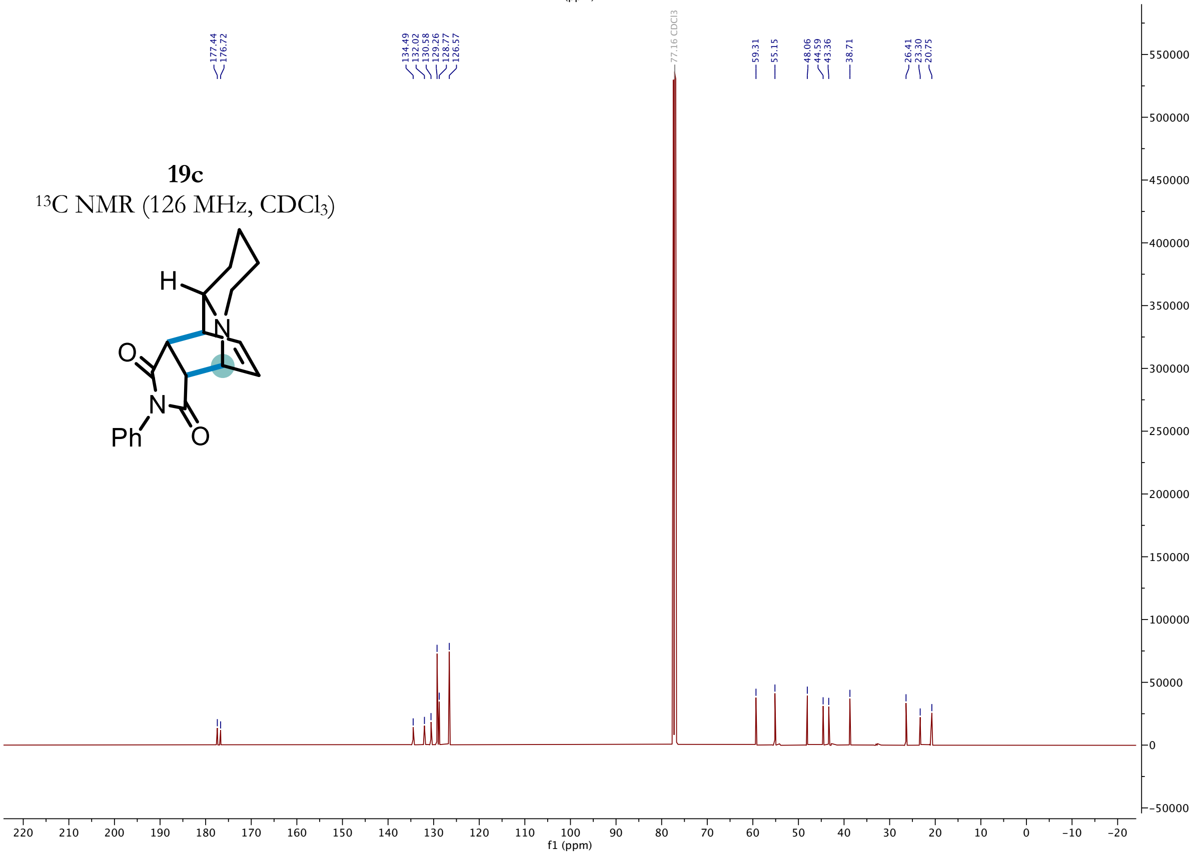
19c
¹³C NMR (126 MHz, CDCl₃)
177.44
176.72
134.49
132.02
130.58
129.26
128.77
126.57
77.16 CDCl3
59.31
55.15
48.06
44.59
43.36
38.71
26.41
23.30
20.75
f1 (ppm)

19d

^{1}H NMR (500 MHz, C_6D_6)

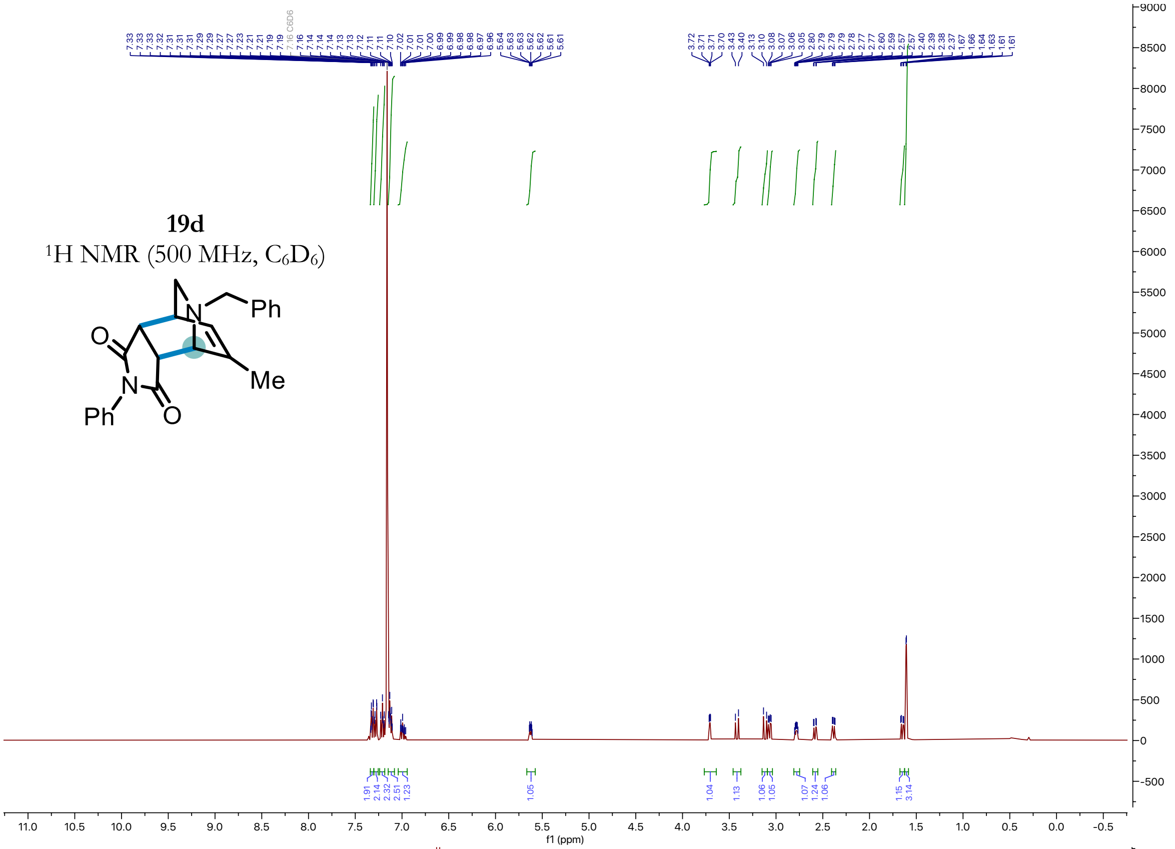

19d

^{13}C NMR (126 MHz, C_6D_6)

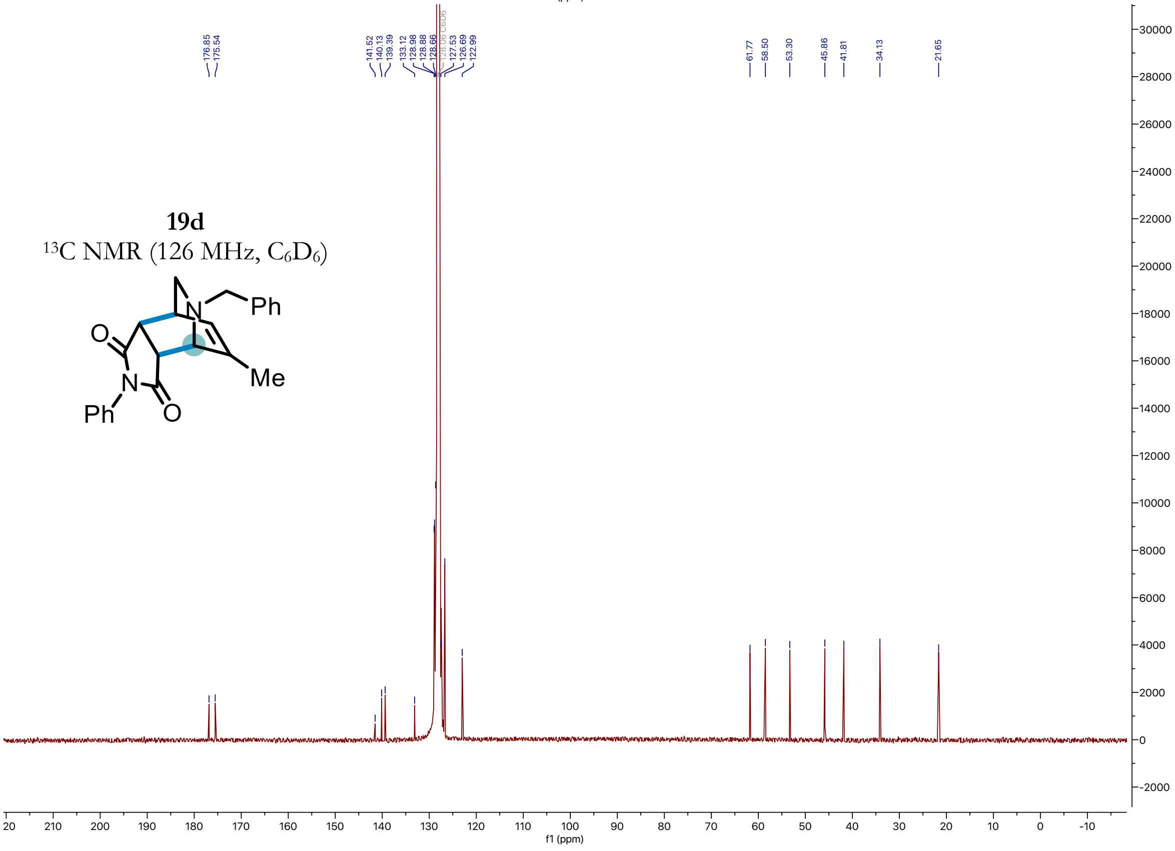

19e

^{1}H NMR (500 MHz, CDCl$_3$)

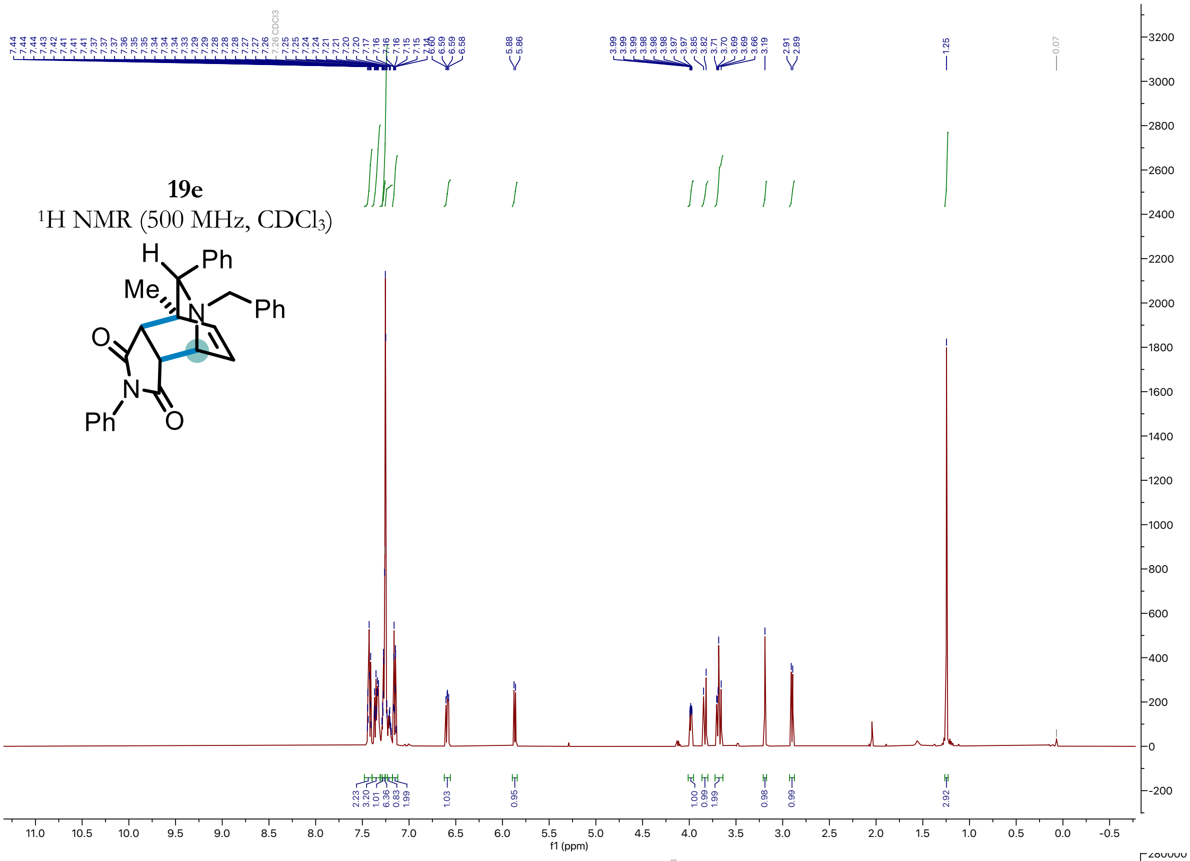

19e

^{13}C NMR (126 MHz, CDCl$_3$)

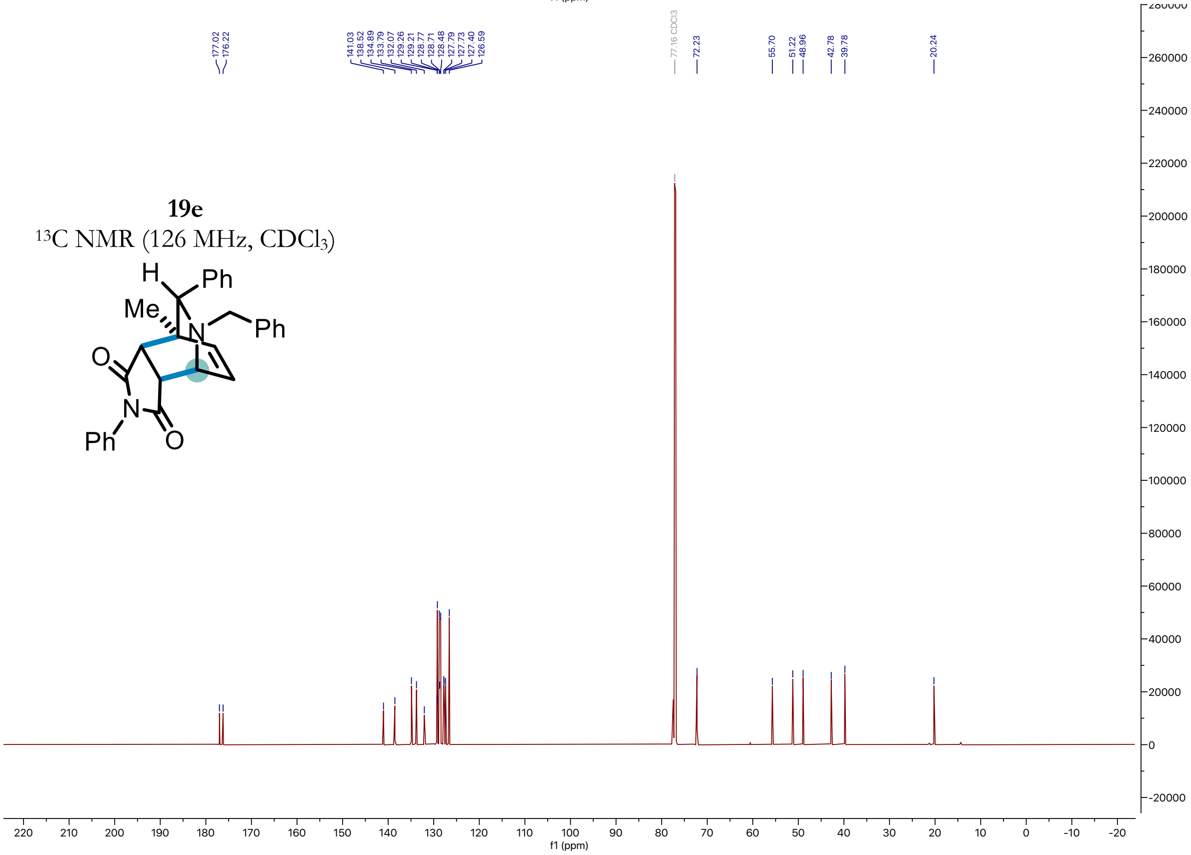

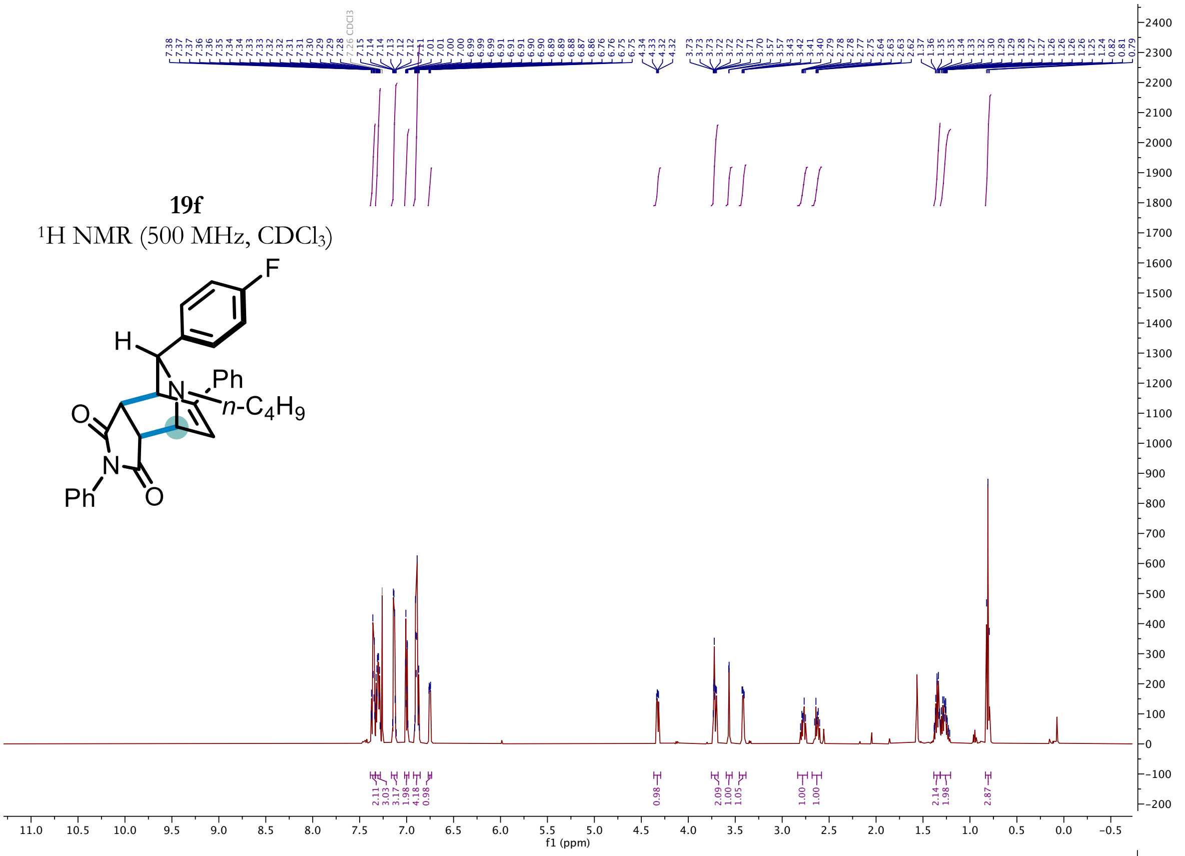
19f
1H NMR (500 MHz, CDCl3)
f1 (ppm)

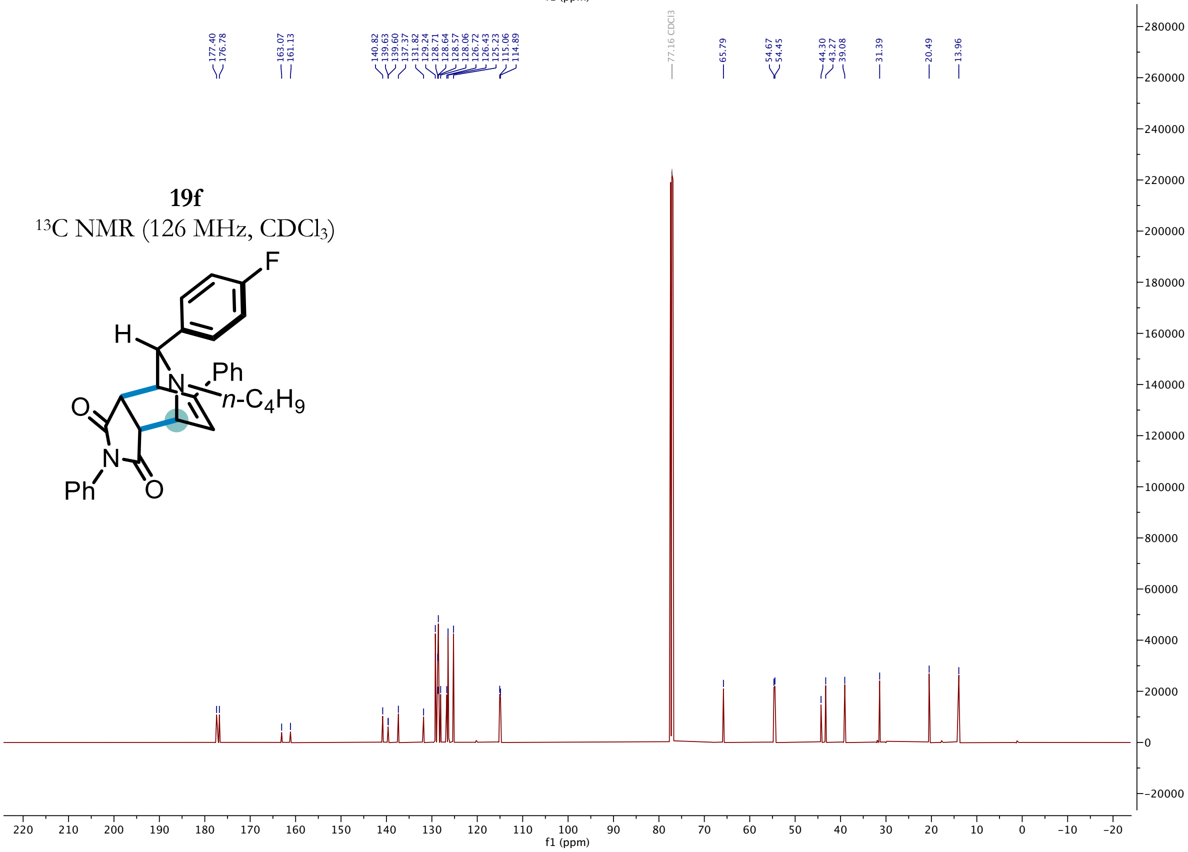
19f
13C NMR (126 MHz, CDCl3)
f1 (ppm)

19f

^{19}F NMR (376 MHz, $CDCl_3$)

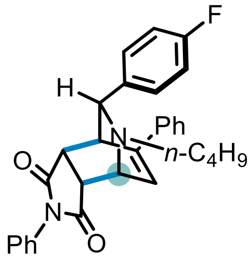

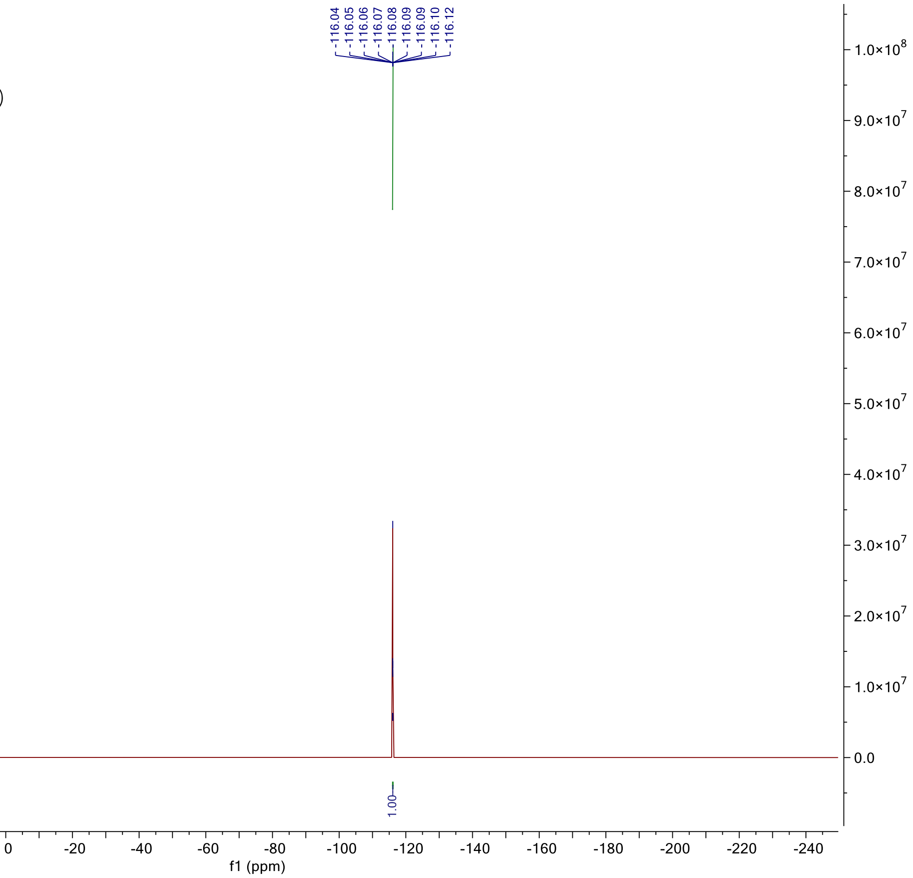

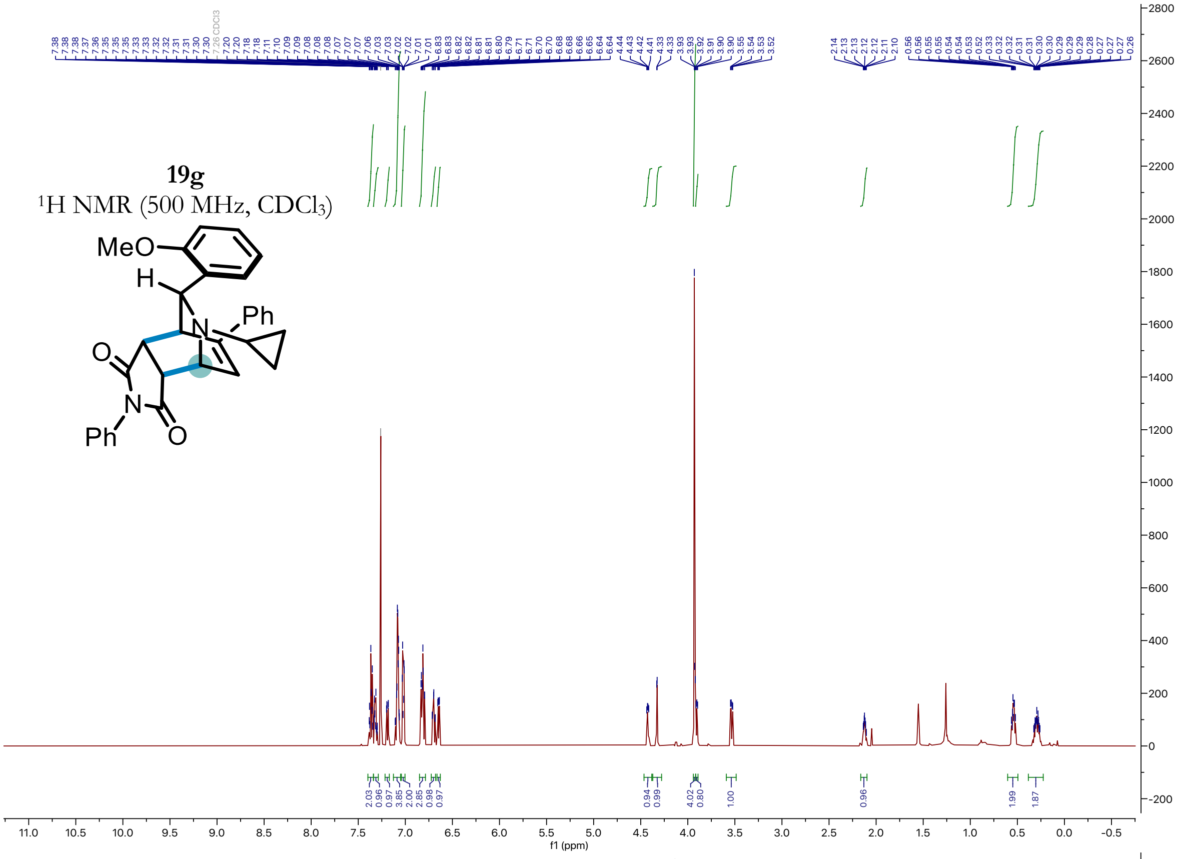
19g
^{1}H NMR (500 MHz, CDCl$_3$)
MeO
H
N
Ph
O
N
Ph
O
f1 (ppm)

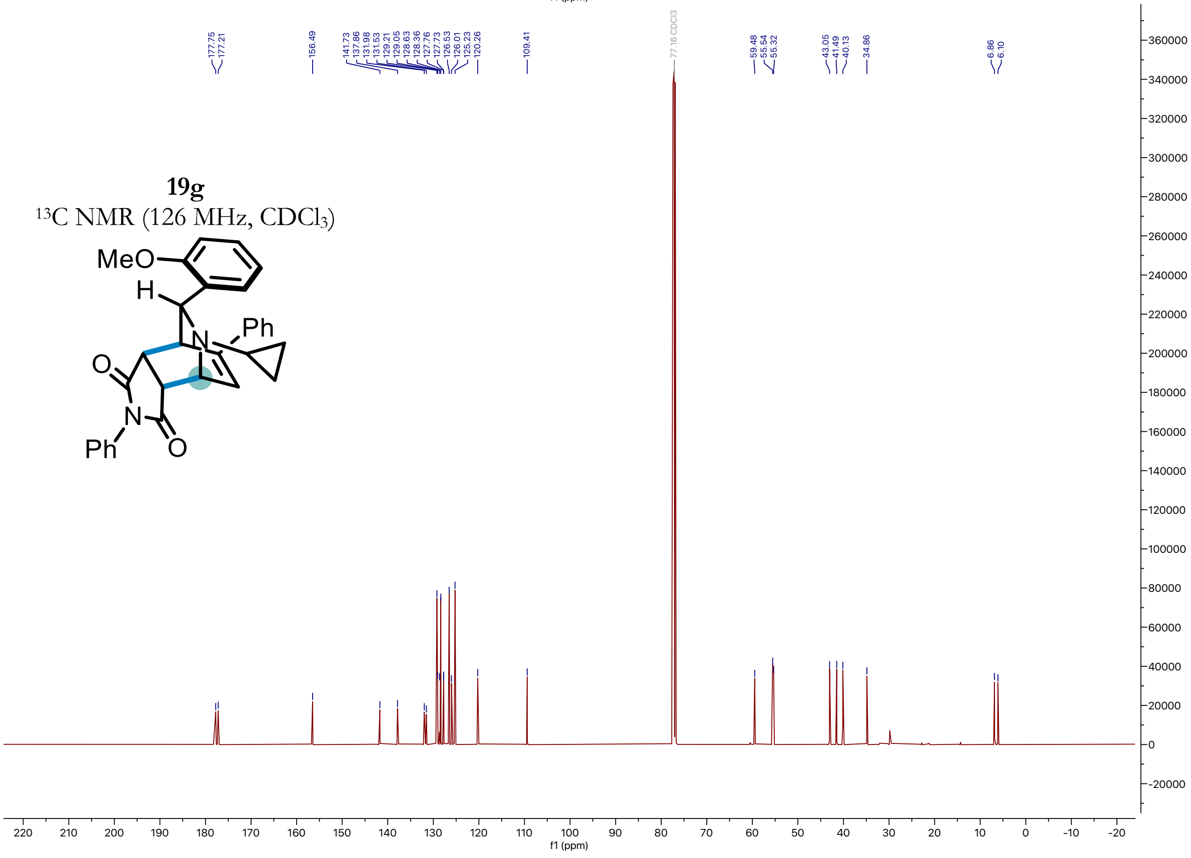
19g
^{13}C NMR (126 MHz, CDCl$_3$)
MeO
H
N
Ph
O
N
Ph
O
177.75
177.21
156.49
109.41
77.16 CDCl3
59.48
55.54
55.32
43.05
41.49
40.13
34.86
6.86
6.10
f1 (ppm)

19h

^{13}C NMR (500 MHz, $CDCl_3$)

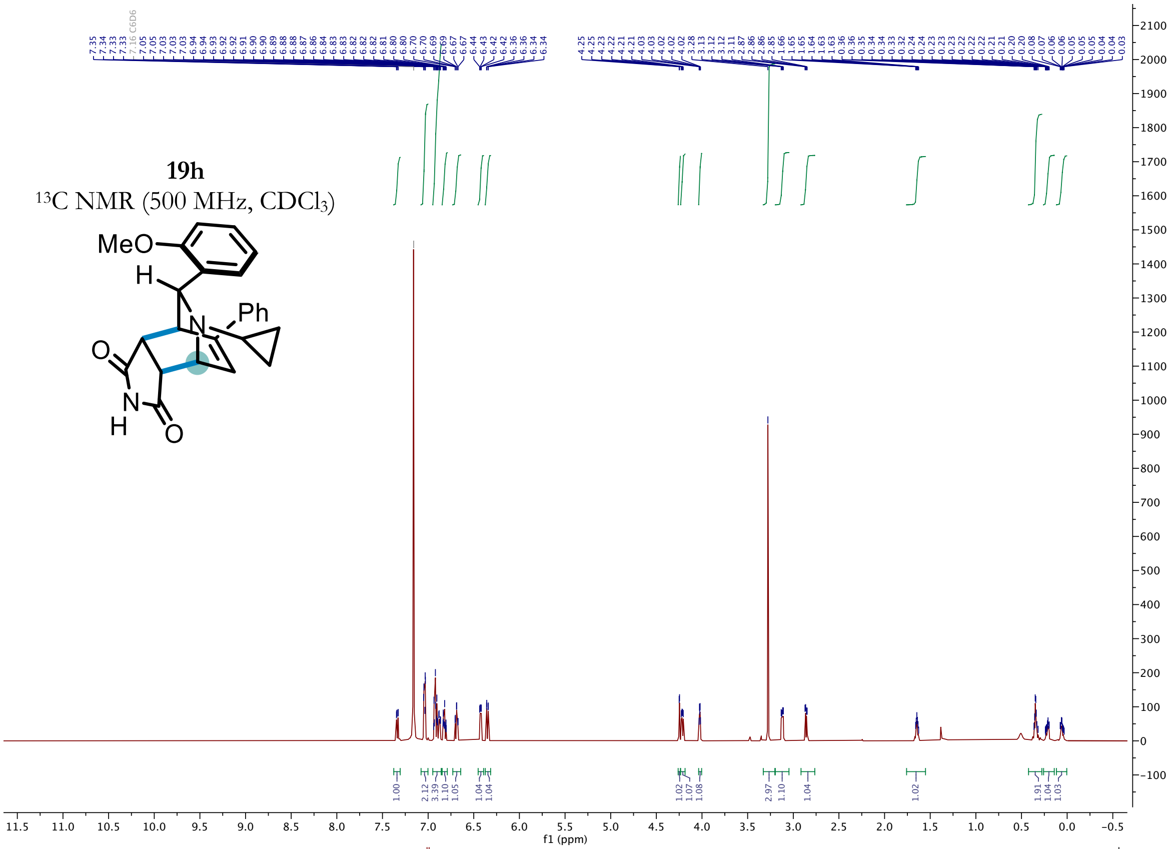

19h

^{13}C NMR (126 MHz, $CDCl_3$)

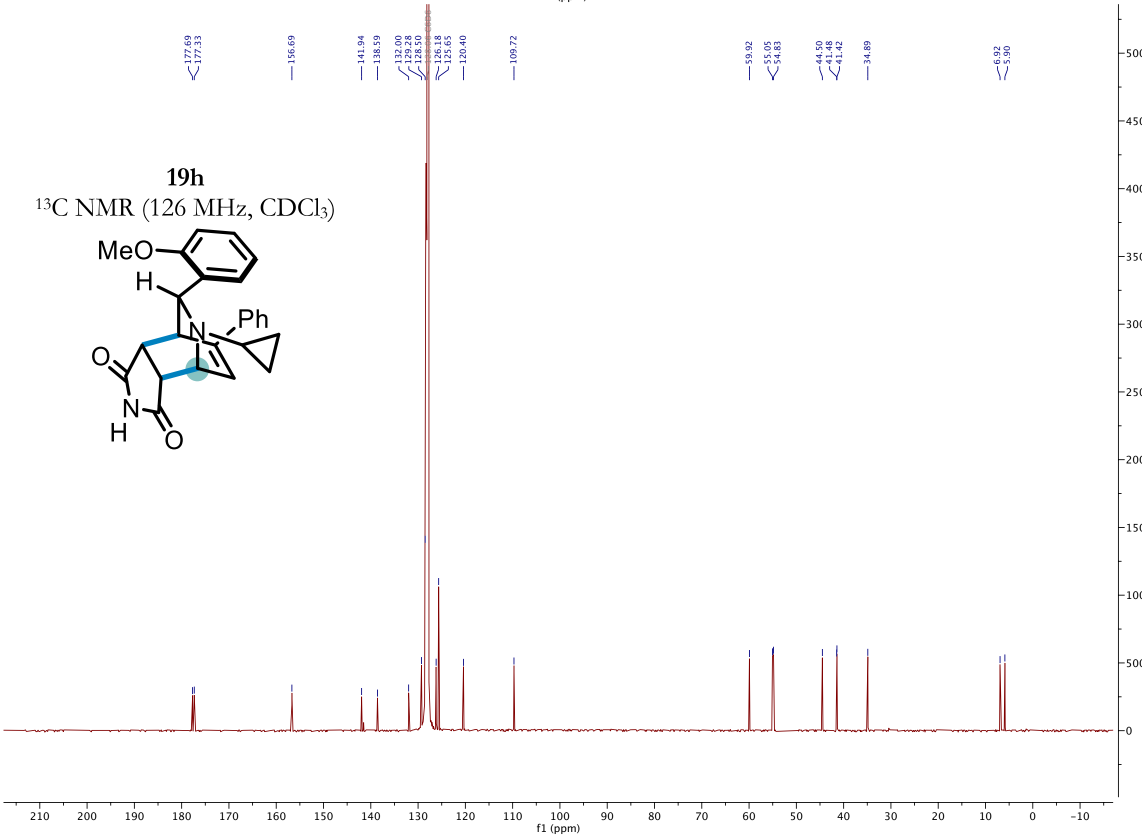

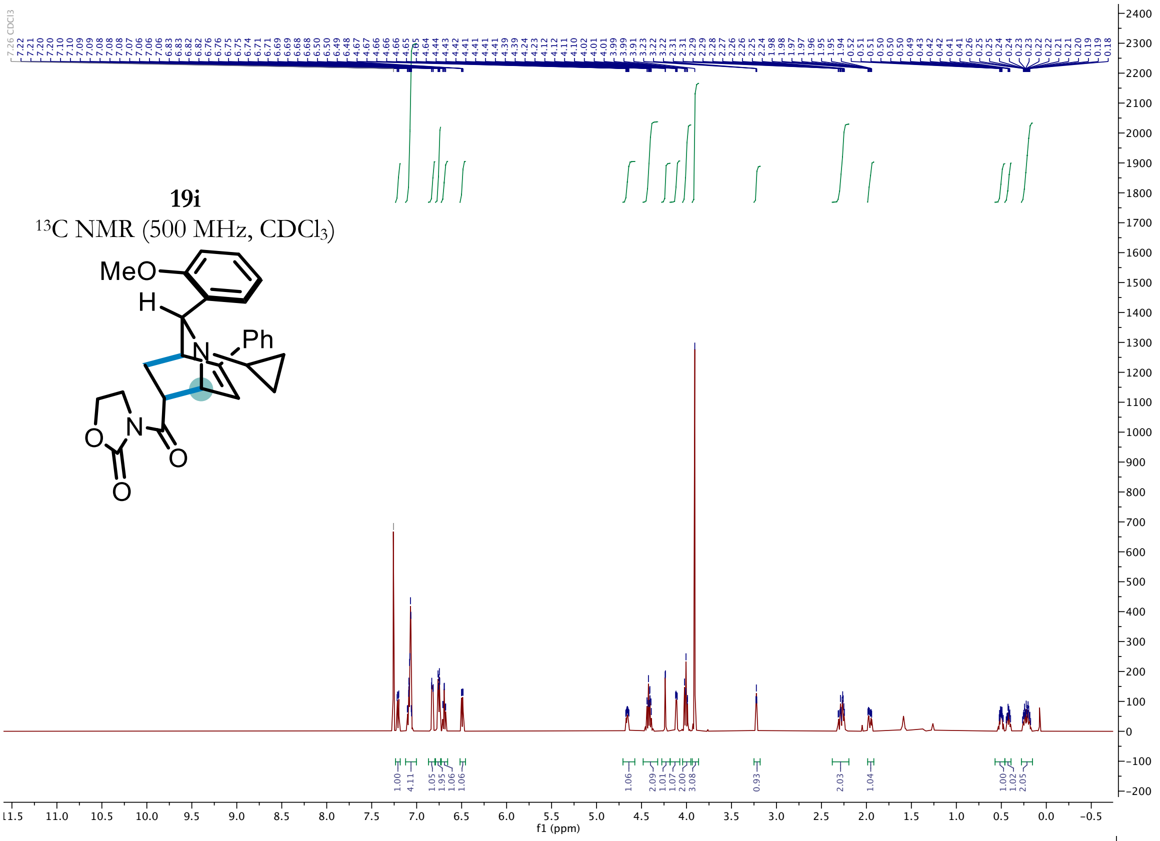
19i
13C NMR (500 MHz, CDCl3)
MeO
H
N
Ph
O
f1 (ppm)

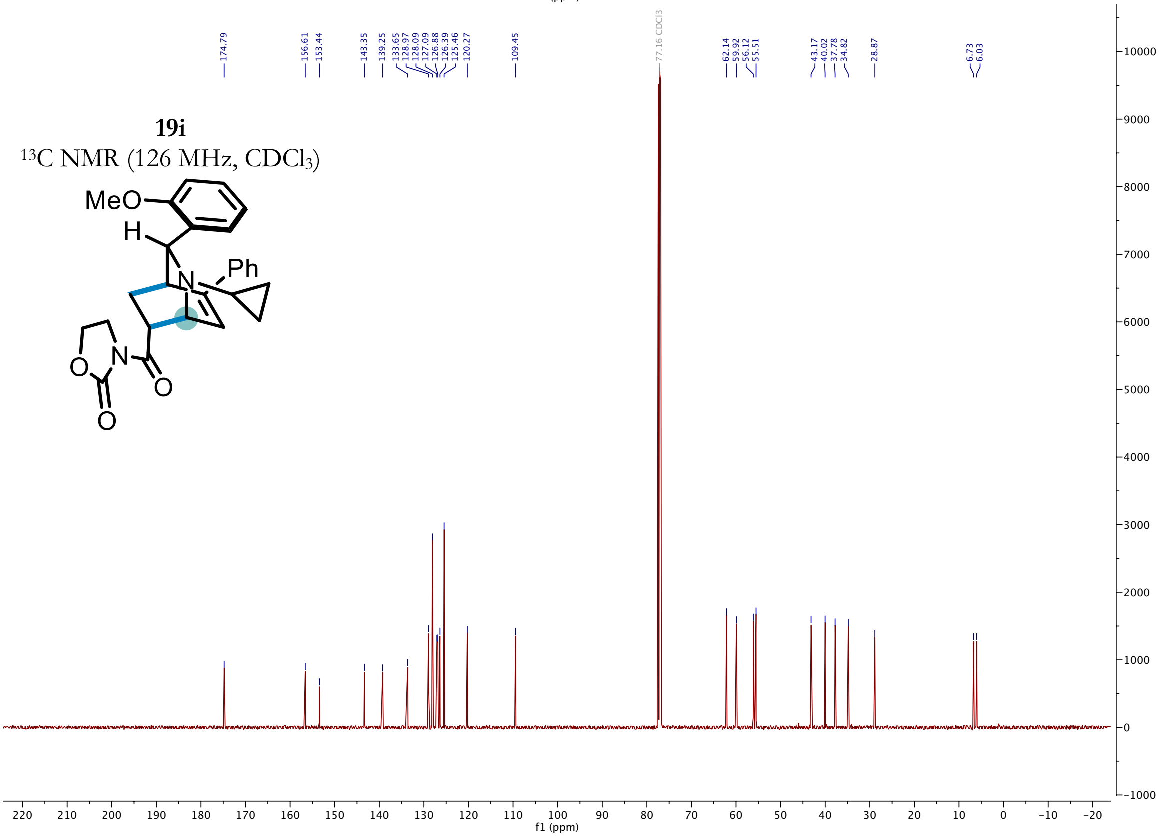
19i
13C NMR (126 MHz, CDCl3)
MeO
H
N
Ph
O
174.79
156.61
153.44
143.35
139.25
133.65
128.97
128.09
127.09
126.88
126.39
125.46
120.27
109.45
77.16 CDCl3
62.14
59.92
56.12
55.51
43.17
40.02
37.78
34.82
28.87
6.73
6.03
f1 (ppm)

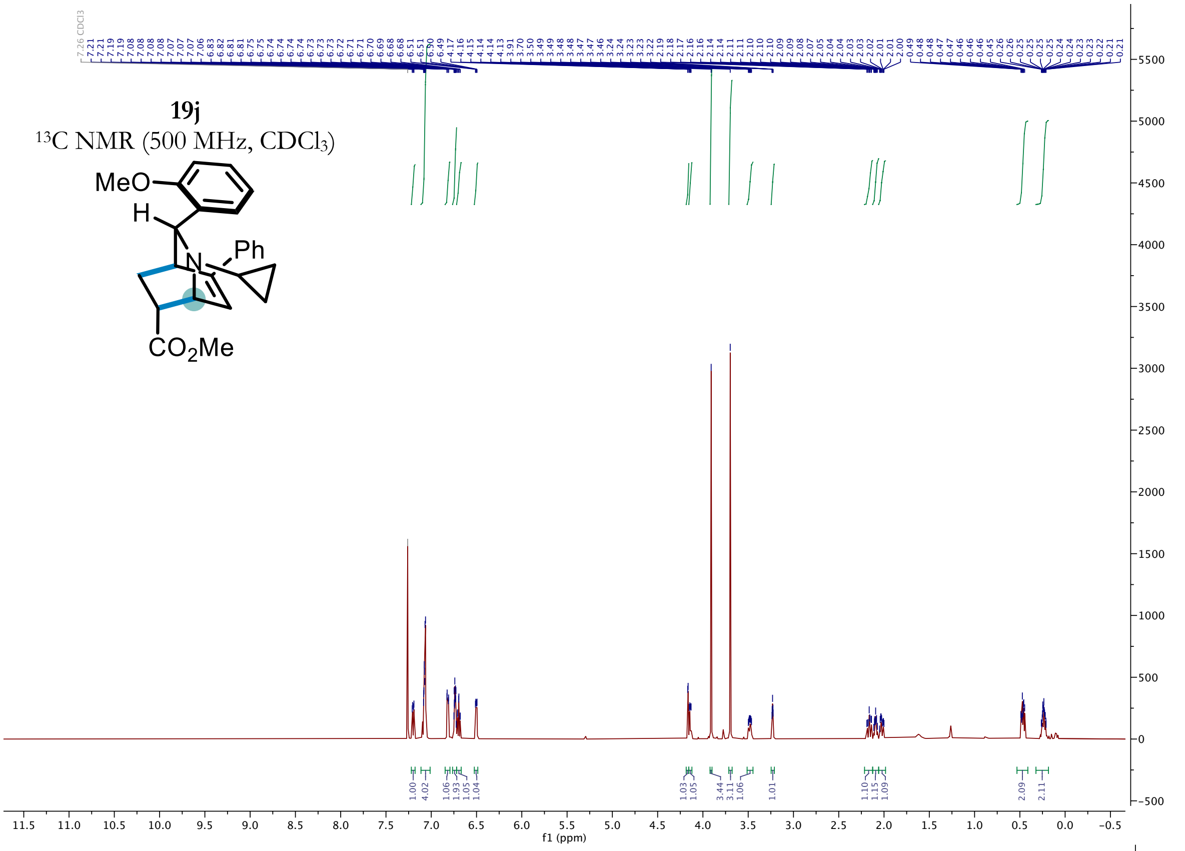
19j
13C NMR (500 MHz, CDCl3)
MeO
H
N
Ph
CO2Me
f1 (ppm)

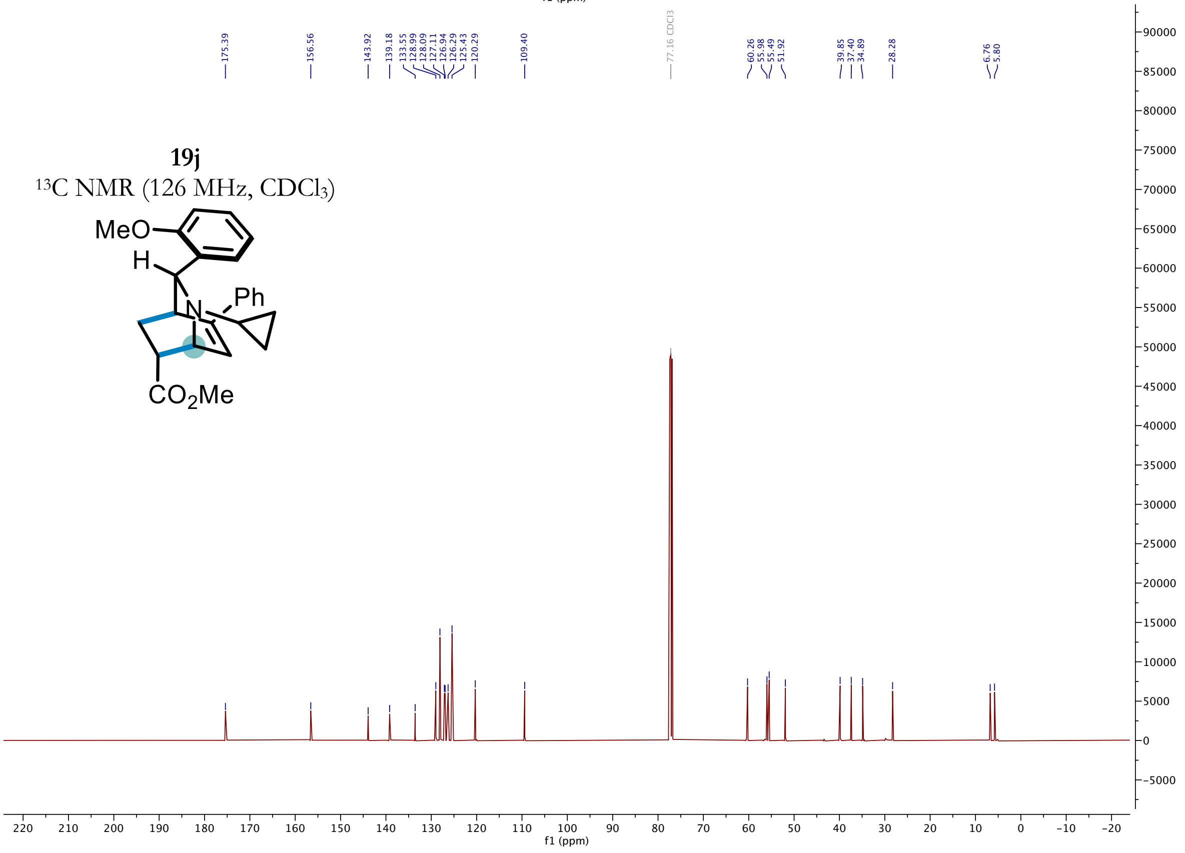
19j
13C NMR (126 MHz, CDCl3)
MeO
H
N
Ph
CO2Me
f1 (ppm)

19k

^{13}C NMR (500 MHz, $CDCl_3$)

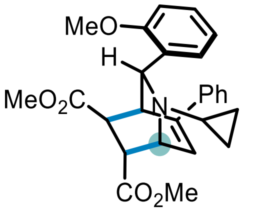

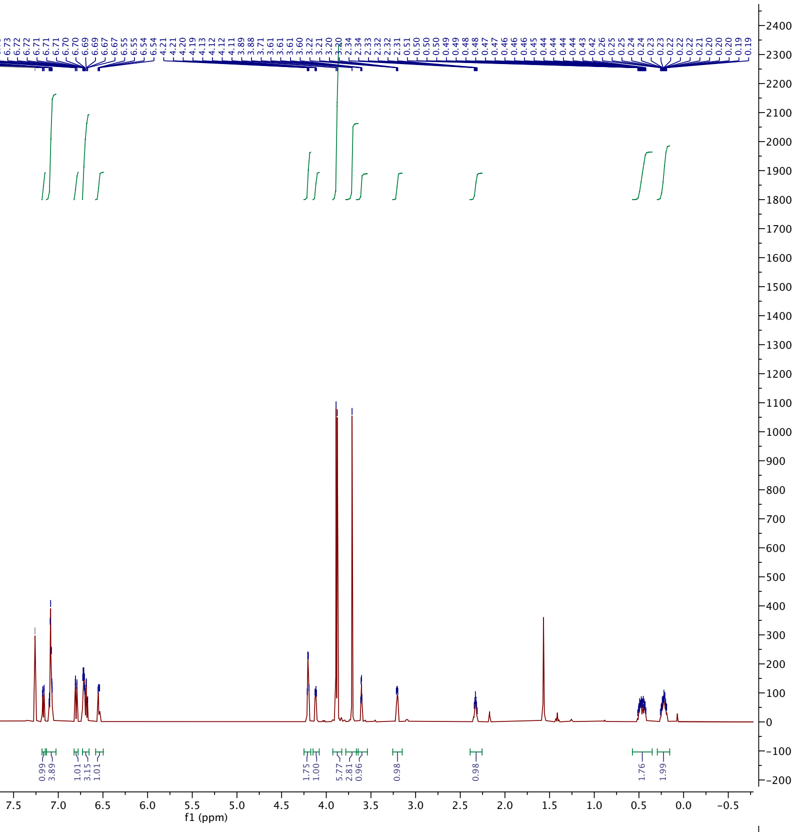

19k

^{13}C NMR (126 MHz, $CDCl_3$)

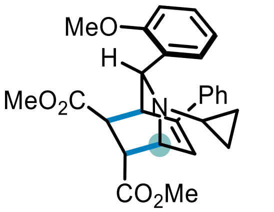

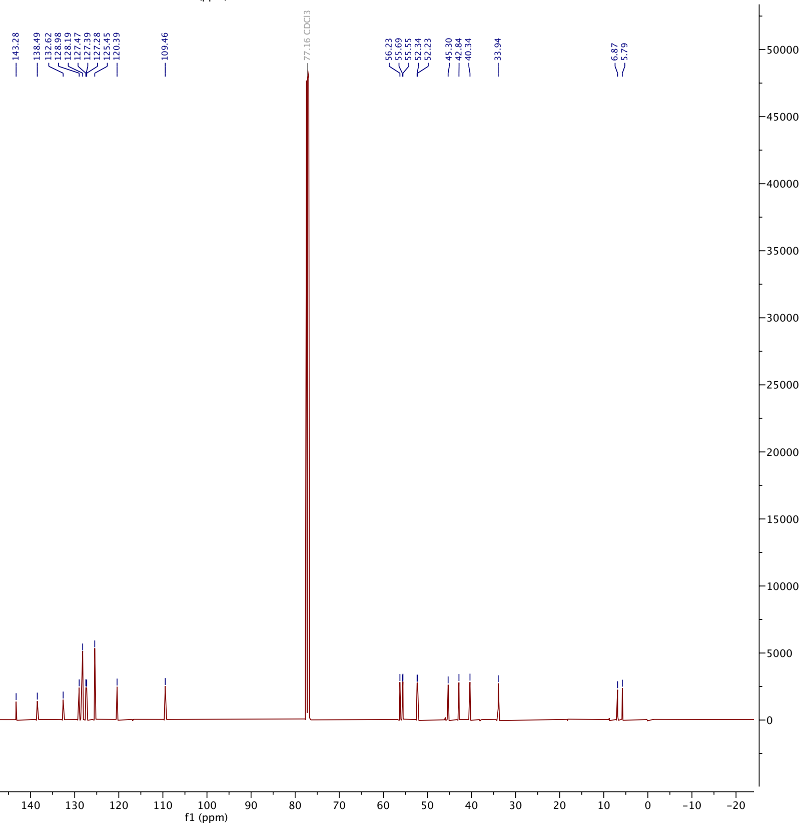

19l (Major)
^{13}C NMR (500 MHz, $CDCl_3$)

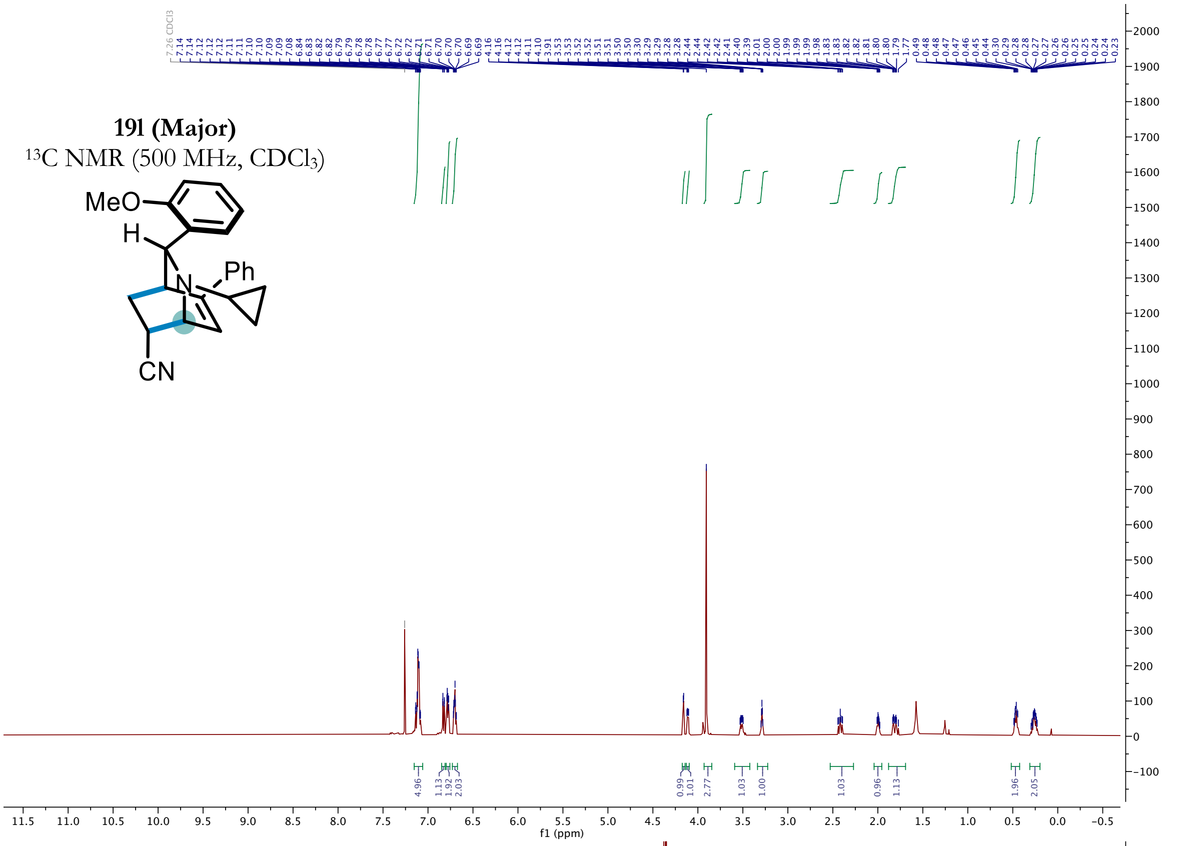

19l (Major)
^{13}C NMR (126 MHz, $CDCl_3$)

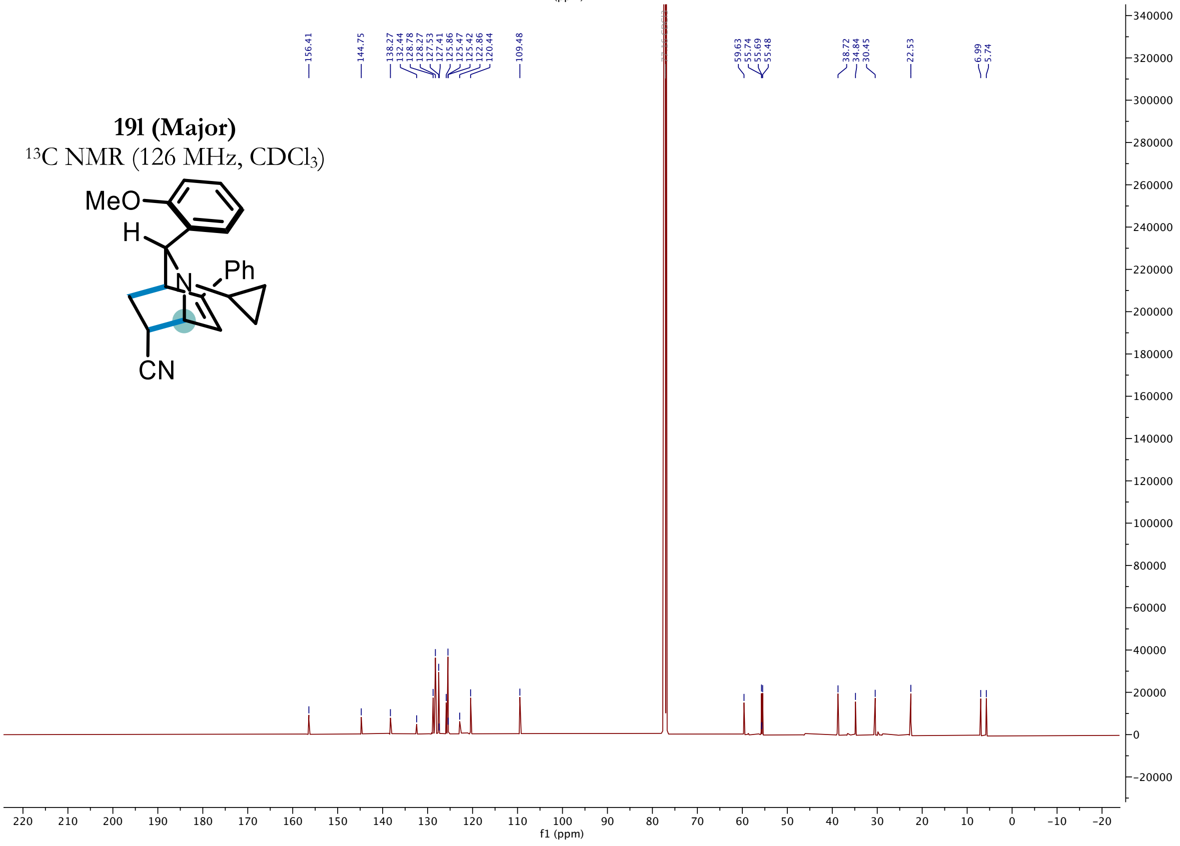

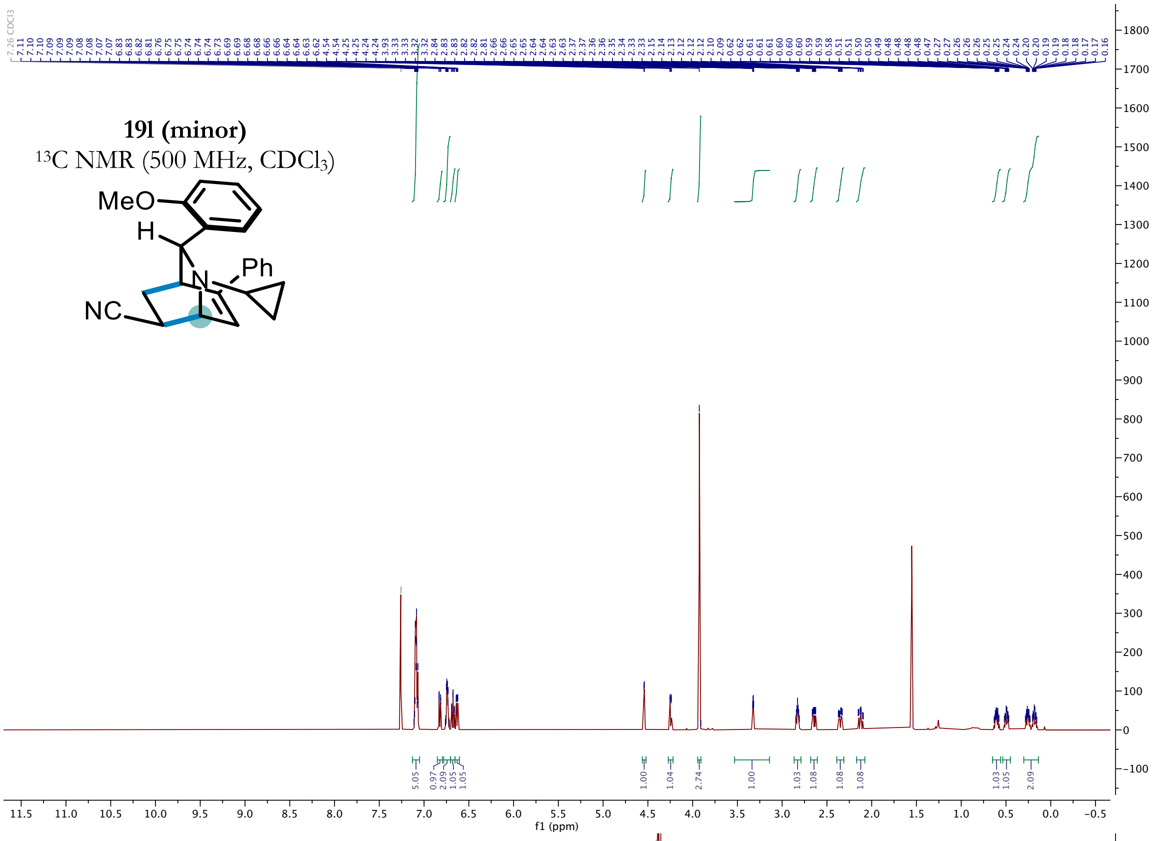
191 (minor)
13C NMR (500 MHz, CDCl3)
MeO
H
N
Ph
NC
5.05
0.97
2.09
1.05
1.05
1.00
1.04
2.74
1.00
1.03
1.08
1.08
1.08
1.03
1.05
2.09
f1 (ppm)

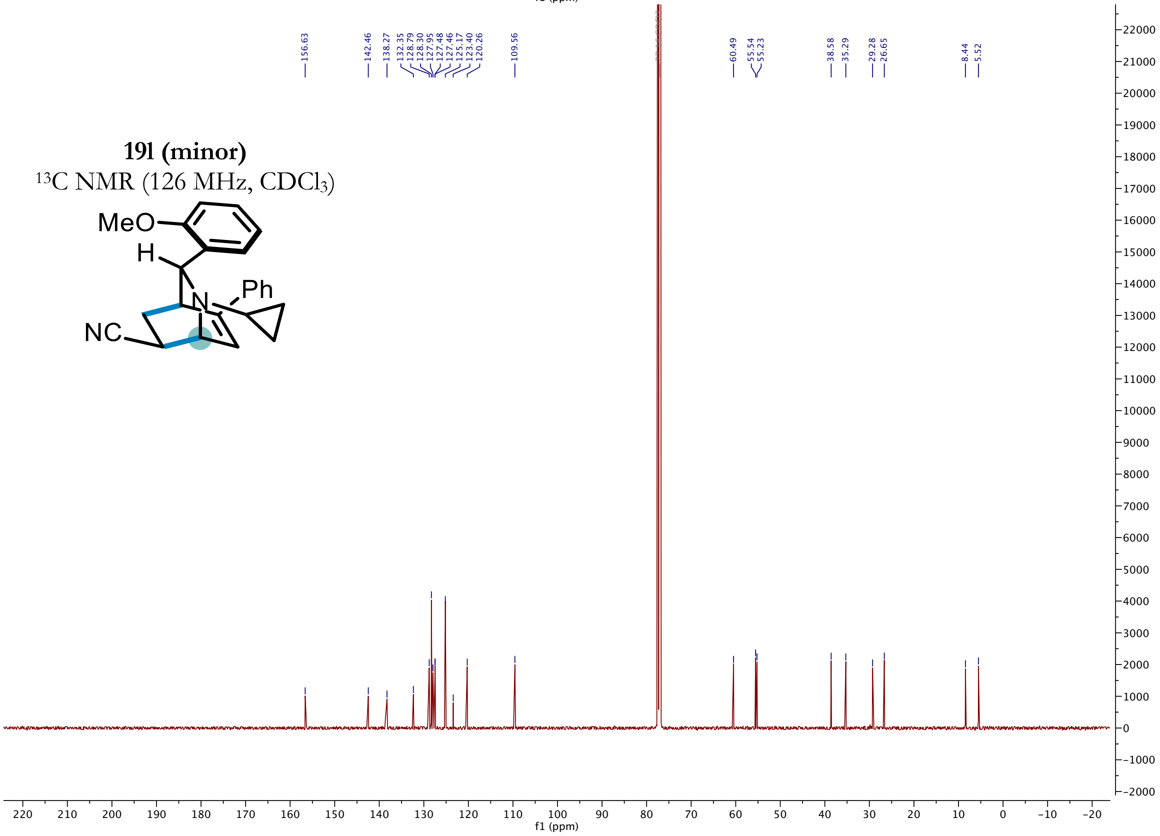
156.63
142.46
138.27
132.35
128.79
128.30
127.95
127.48
127.46
125.17
123.40
120.26
109.56
60.49
55.54
55.23
38.58
35.29
29.28
26.65
8.44
5.52
191 (minor)
13C NMR (126 MHz, CDCl3)
MeO
H
N
Ph
NC
f1 (ppm)

24a

^{1}H NMR (400 MHz, C_6D_6)

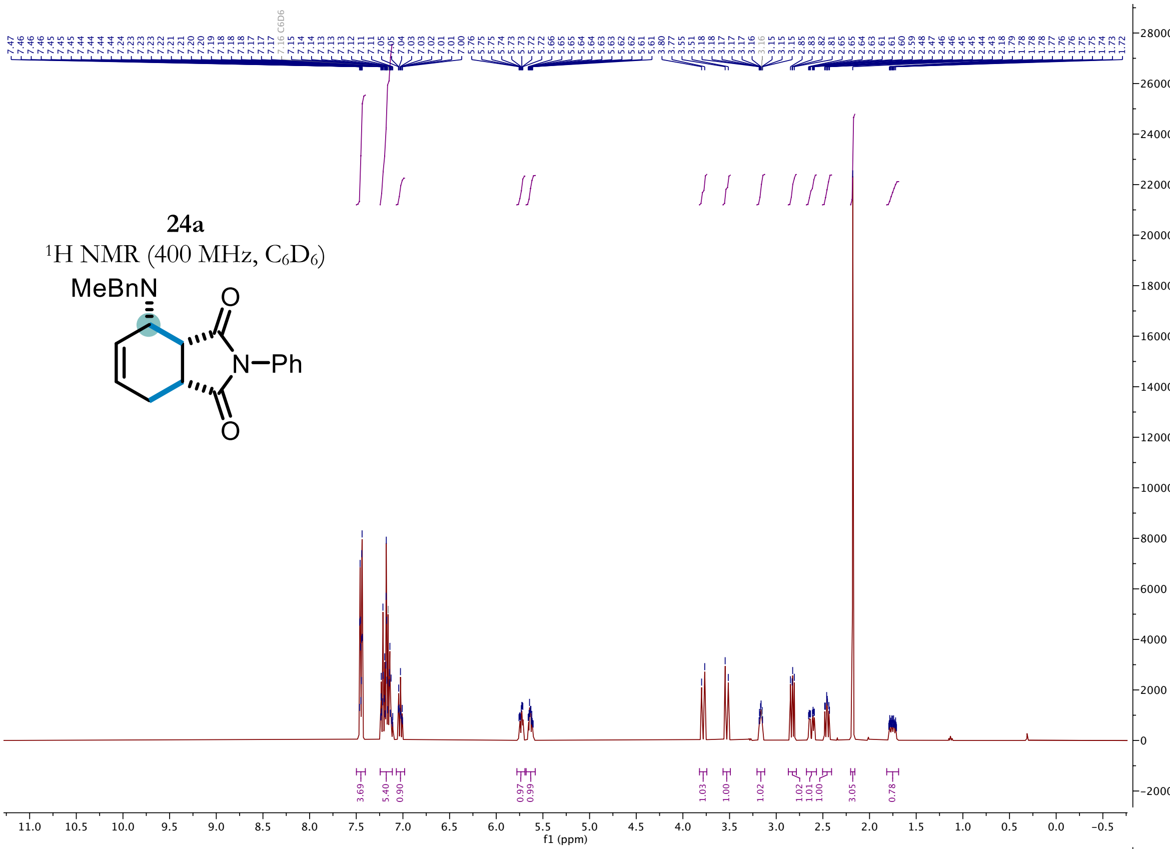

24a

^{13}C NMR (101 MHz, C_6D_6)

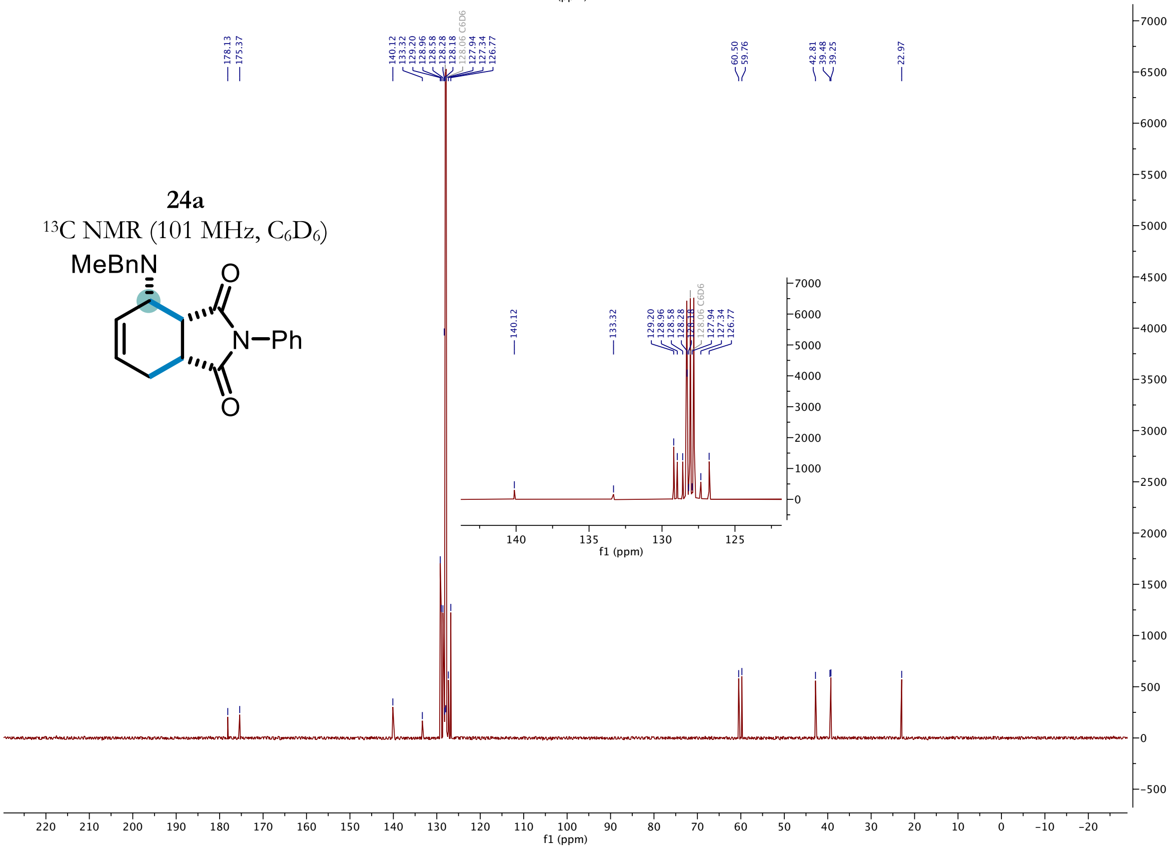

24b

^1H NMR (400 MHz, C_6D_6)

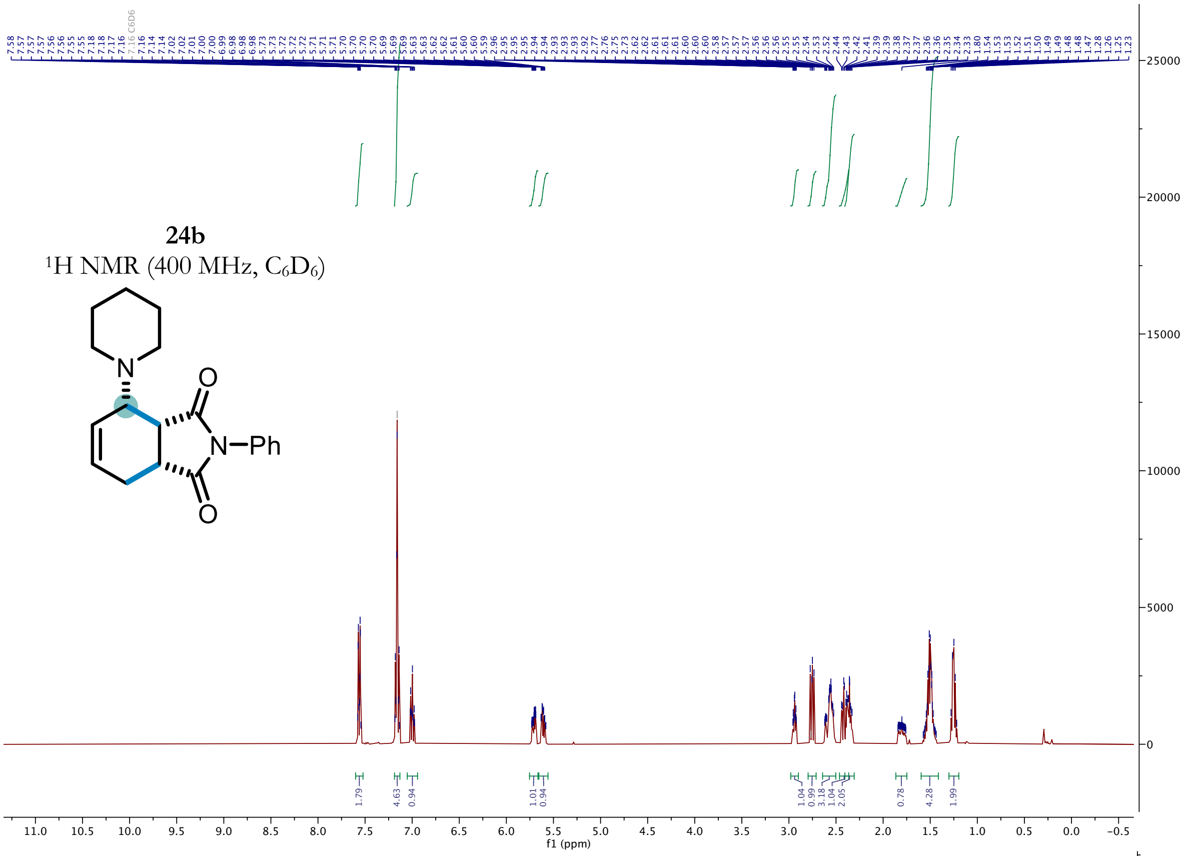

24b

^{13}C NMR (101 MHz, C_6D_6)

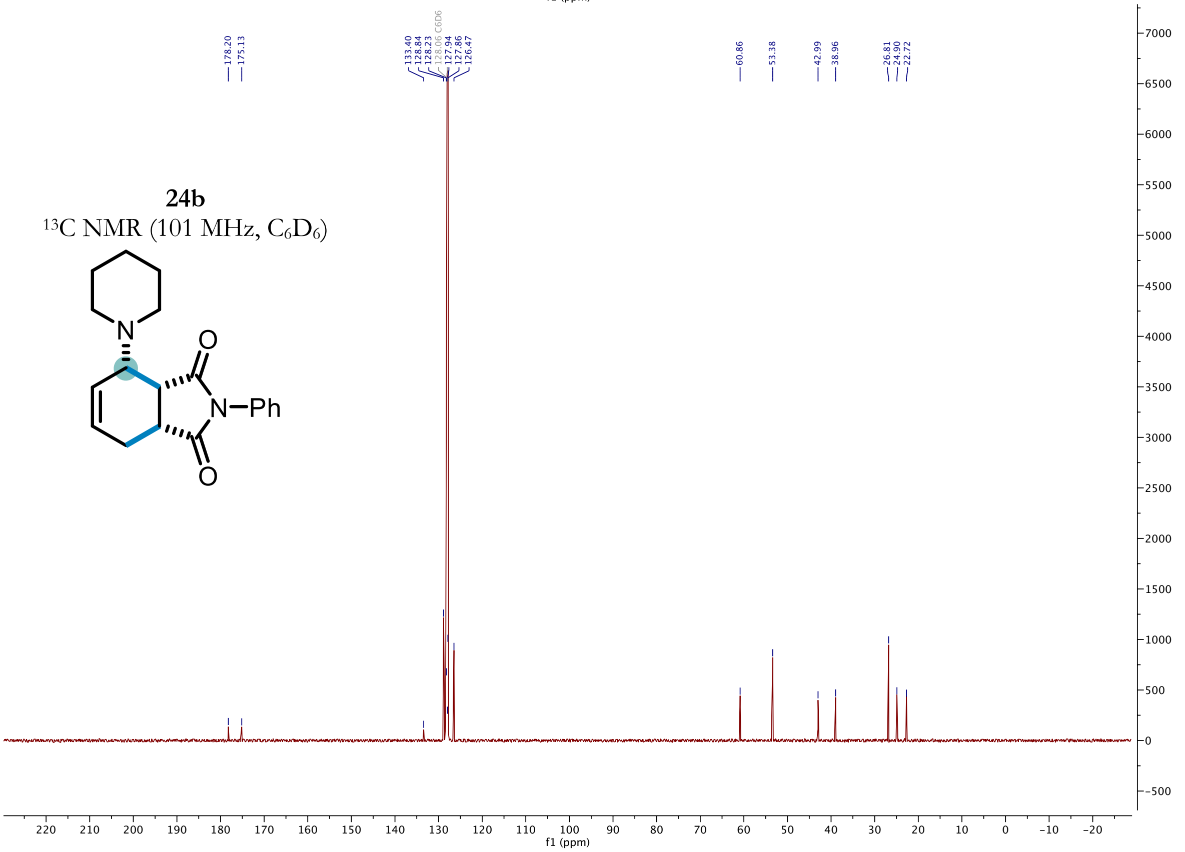

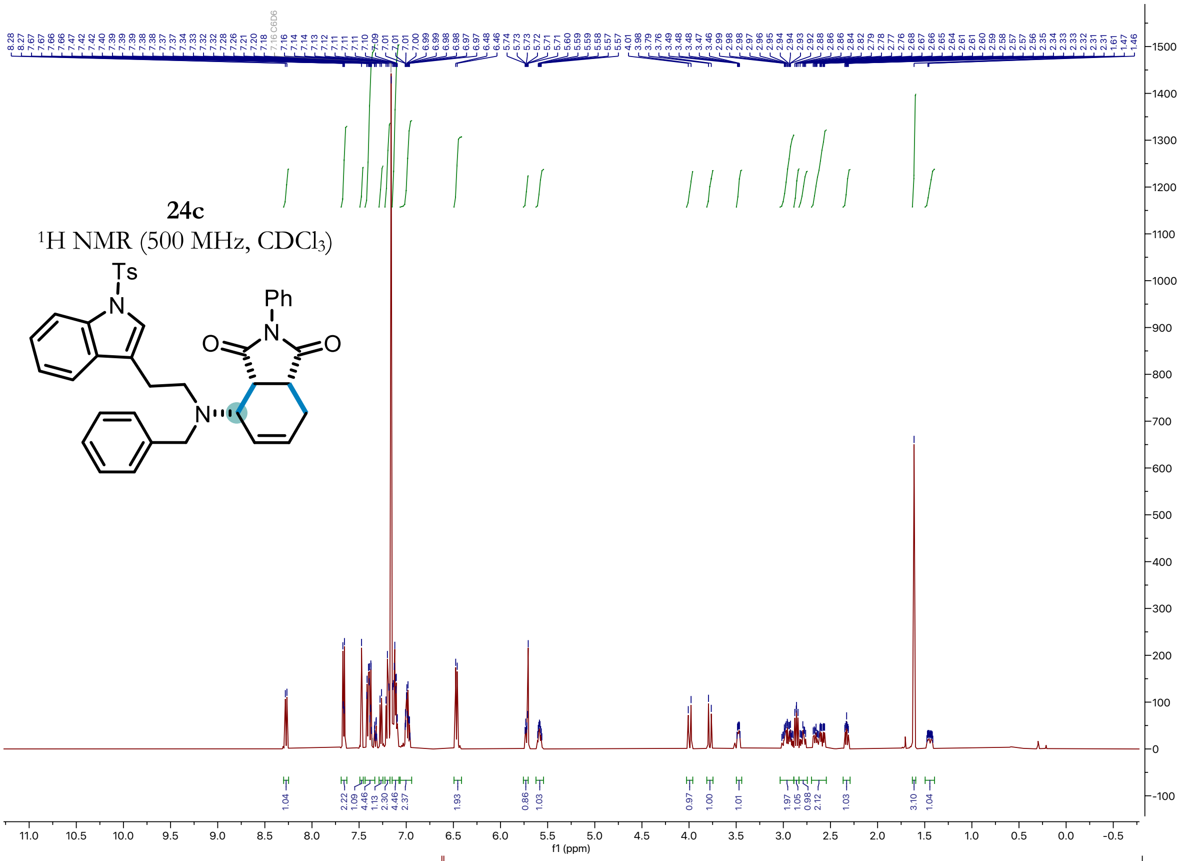
8.28 8.27 7.67 7.67 7.66 7.66 7.47 7.42 7.42 7.40 7.39 7.39 7.39 7.38 7.38 7.37 7.37 7.34 7.33 7.32 7.28 7.26 7.21 7.20 7.18 7.16 C6D6 7.16 7.14 7.14 7.13 7.12 7.11 7.11 7.11 7.10 7.09 7.01 7.01 7.01 7.00 6.99 6.99 6.98 6.98 6.97 6.97 6.48 6.46 5.74 5.73 5.73 5.72 5.71 5.71 5.60 5.59 5.59 5.58 5.57 5.57 4.01 3.98 3.79 3.76 3.49 3.48 3.47 3.46 2.99 2.98 2.98 2.97 2.96 2.95 2.94 2.94 2.93 2.92 2.88 2.86 2.86 2.84 2.82 2.79 2.78 2.77 2.76 2.68 2.67 2.66 2.65 2.64 2.61 2.60 2.59 2.58 2.57 2.57 2.56 2.35 2.34 2.33 2.33 2.32 2.31 1.61 1.47 1.46
24c
1H NMR (500 MHz, CDCl3)
Ts
N
Ph
N
O
O
N
1.04 2.22 1.09 4.46 1.13 2.30 4.46 2.37 1.93 0.86 1.03 0.97 1.00 1.01 1.97 1.05 0.98 2.12 1.03 3.10 1.04
f1 (ppm)

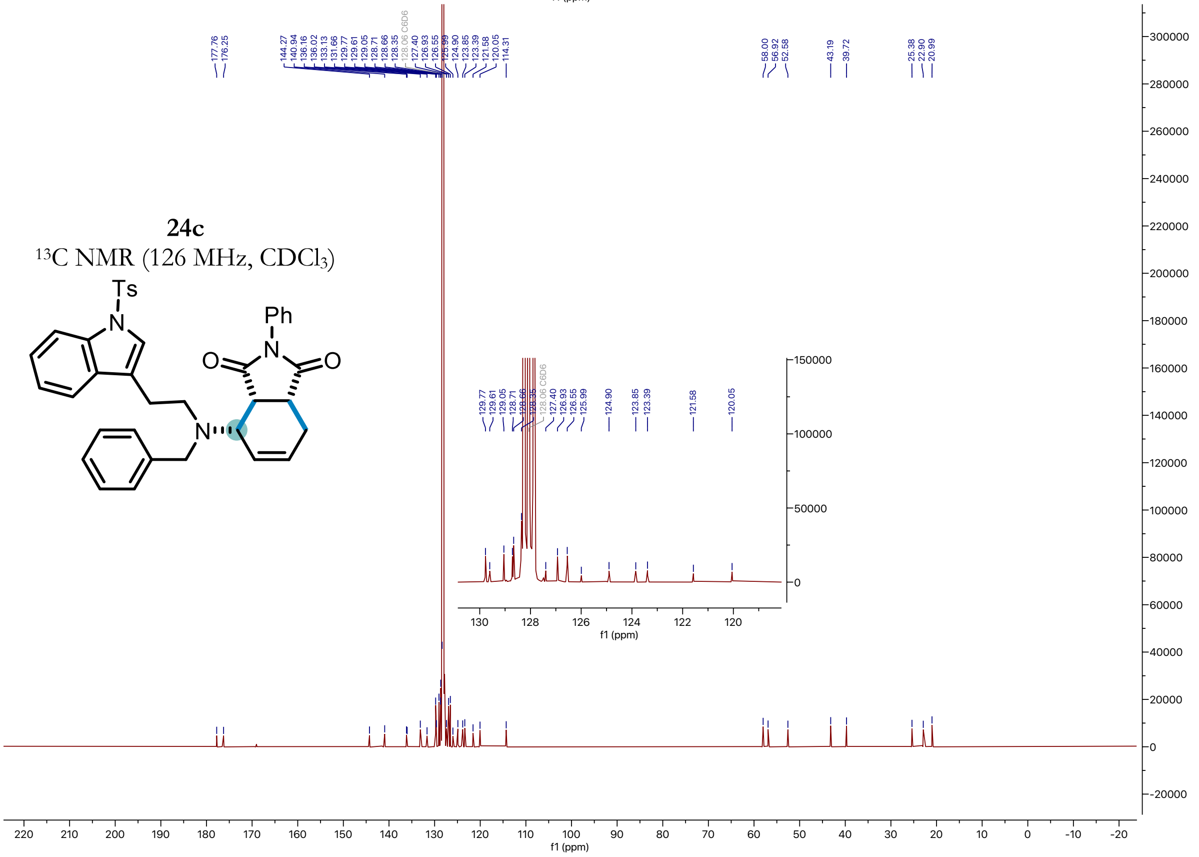
177.76 176.25 144.27 140.94 136.16 136.02 133.13 131.66 129.77 129.61 129.05 128.71 128.66 128.35 128.06 C6D6 127.40 126.93 126.55 125.99 124.90 123.85 123.39 121.58 120.05 114.31 58.00 56.92 52.58 43.19 39.72 25.38 22.90 20.99
24c
13C NMR (126 MHz, CDCl3)
Ts
N
Ph
N
O
O
N
f1 (ppm)

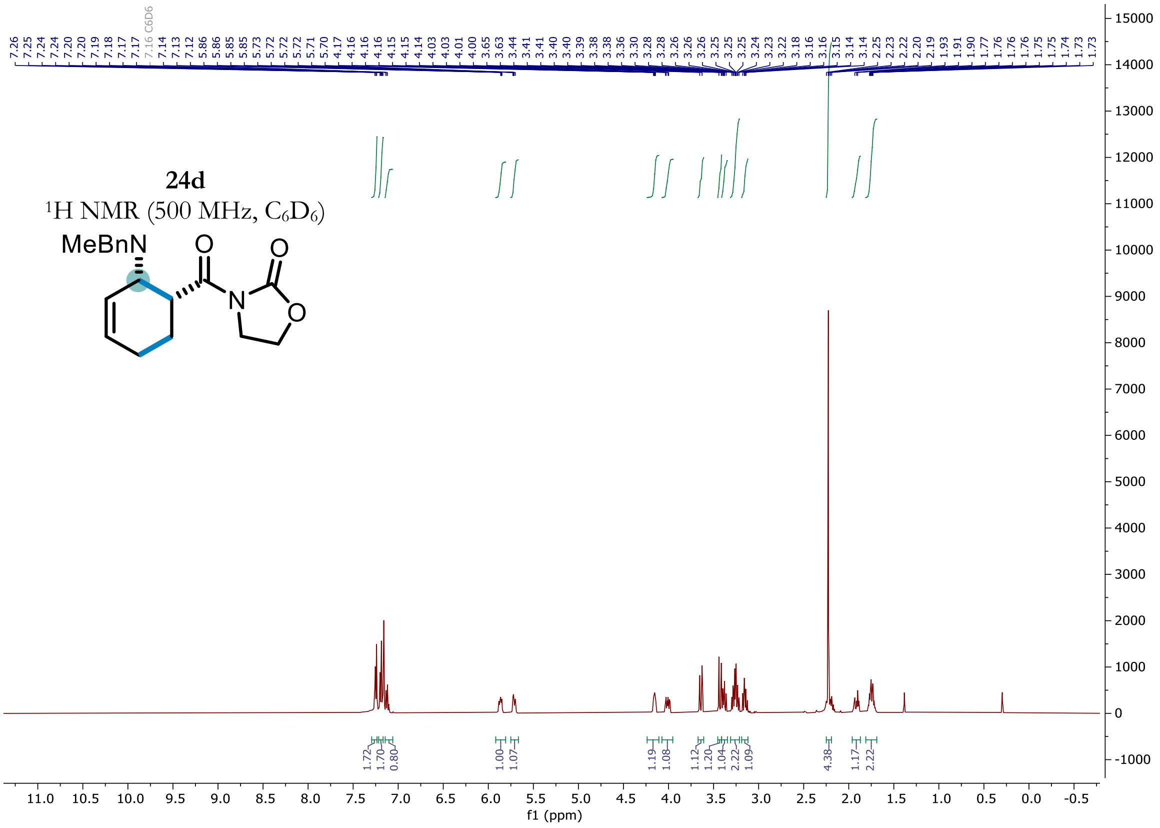
24d
1H NMR (500 MHz, C6D6)
MeBnN
O
O
N
O
f1 (ppm)

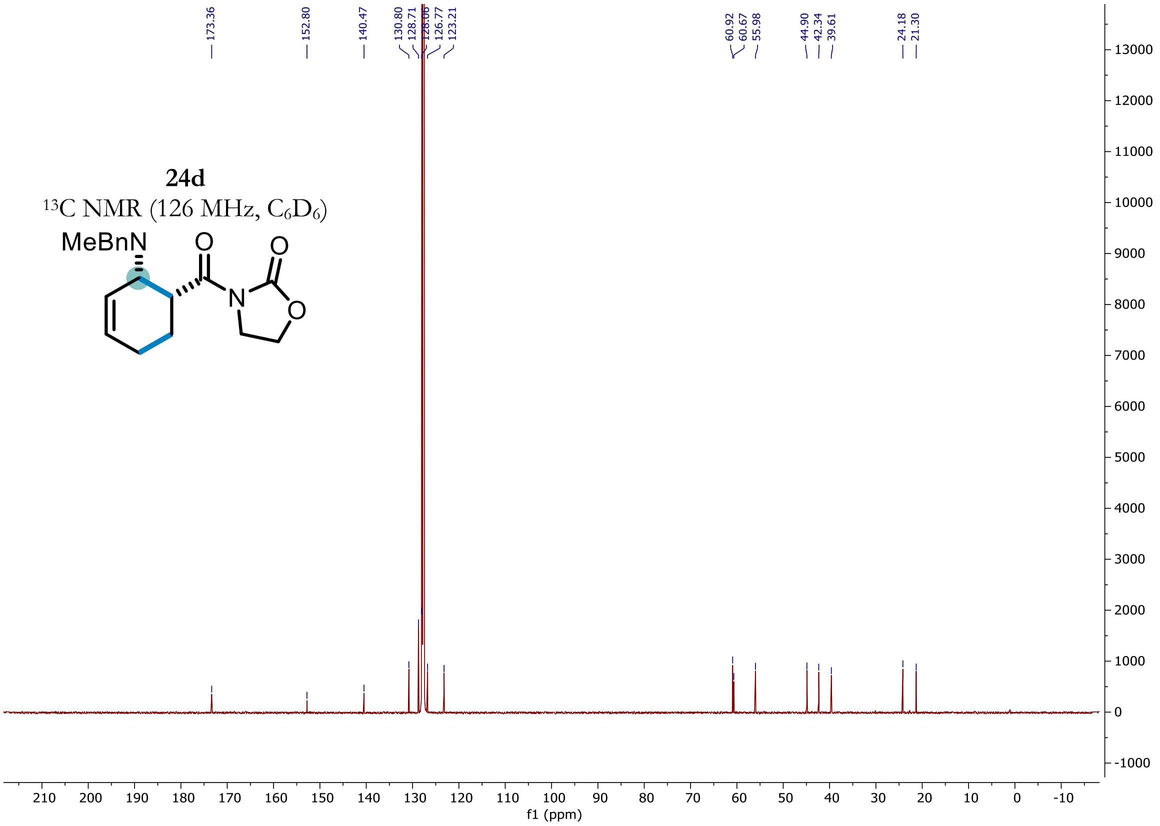
173.36
152.80
140.47
130.80
128.71
128.06
126.77
123.21
60.92
60.67
55.98
44.90
42.34
39.61
24.18
21.30
24d
13C NMR (126 MHz, C6D6)
MeBnN
O
O
N
O
f1 (ppm)

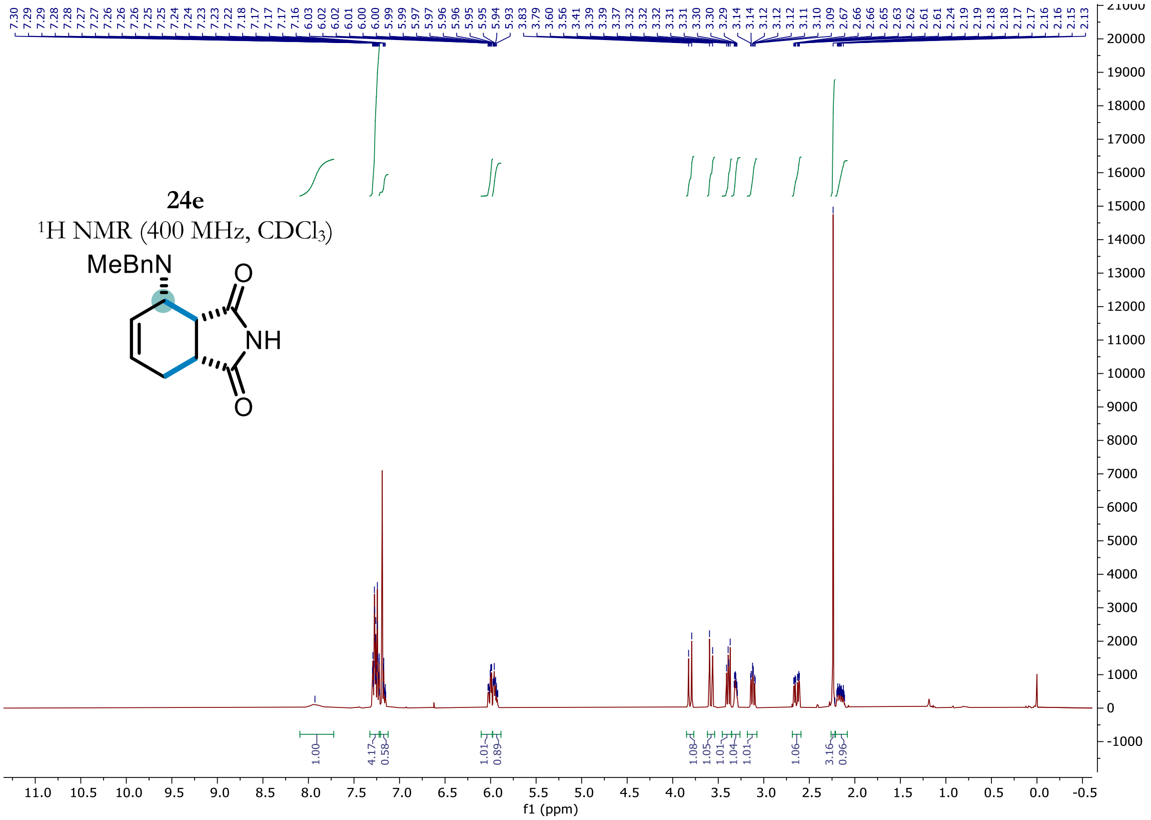
7.30 7.29 7.29 7.28 7.28 7.27 7.27 7.26 7.26 7.25 7.25 7.24 7.24 7.23 7.23 7.22 7.18 7.17 7.17 7.17 7.16 6.03 6.02 6.02 6.01 6.00 6.00 5.99 5.99 5.97 5.97 5.96 5.96 5.95 5.95 5.94 5.93 3.83 3.79 3.60 3.56 3.41 3.39 3.39 3.37 3.32 3.32 3.32 3.31 3.31 3.30 3.30 3.29 3.14 3.14 3.12 3.12 3.12 3.11 3.10 3.09 2.67 2.66 2.66 2.65 2.63 2.62 2.61 2.61 2.24 2.19 2.19 2.18 2.18 2.17 2.17 2.16 2.15 2.13
24e
1H NMR (400 MHz, CDCl3)
MeBnN
O
NH
O
1.00 4.17 0.58 1.01 0.89 1.08 1.05 1.01 1.04 1.01 1.06 3.16 0.96
f1 (ppm)

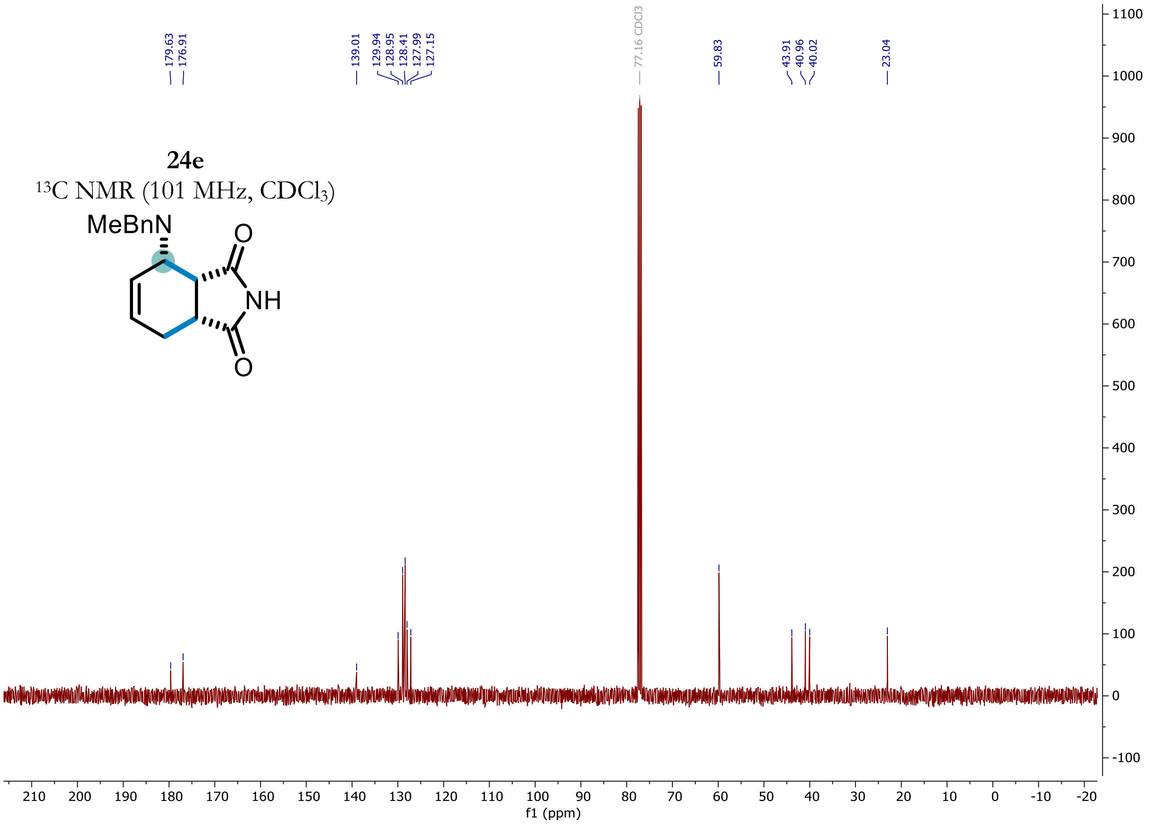
179.63 176.91 139.01 129.94 128.95 128.41 127.99 127.15 77.16 CDCl3 59.83 43.91 40.96 40.02 23.04
24e
13C NMR (101 MHz, CDCl3)
MeBnN
O
NH
O
f1 (ppm)

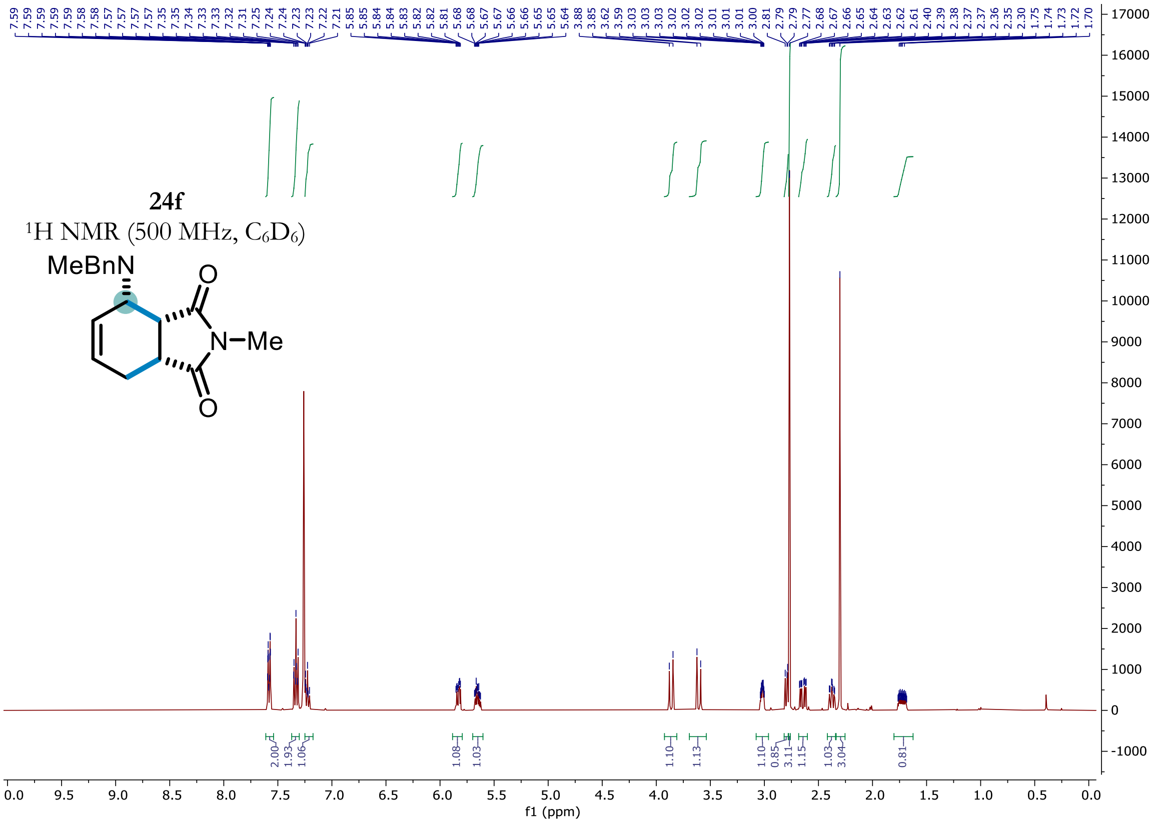
24f
1H NMR (500 MHz, C6D6)
MeBnN
O
N−Me
O
f1 (ppm)

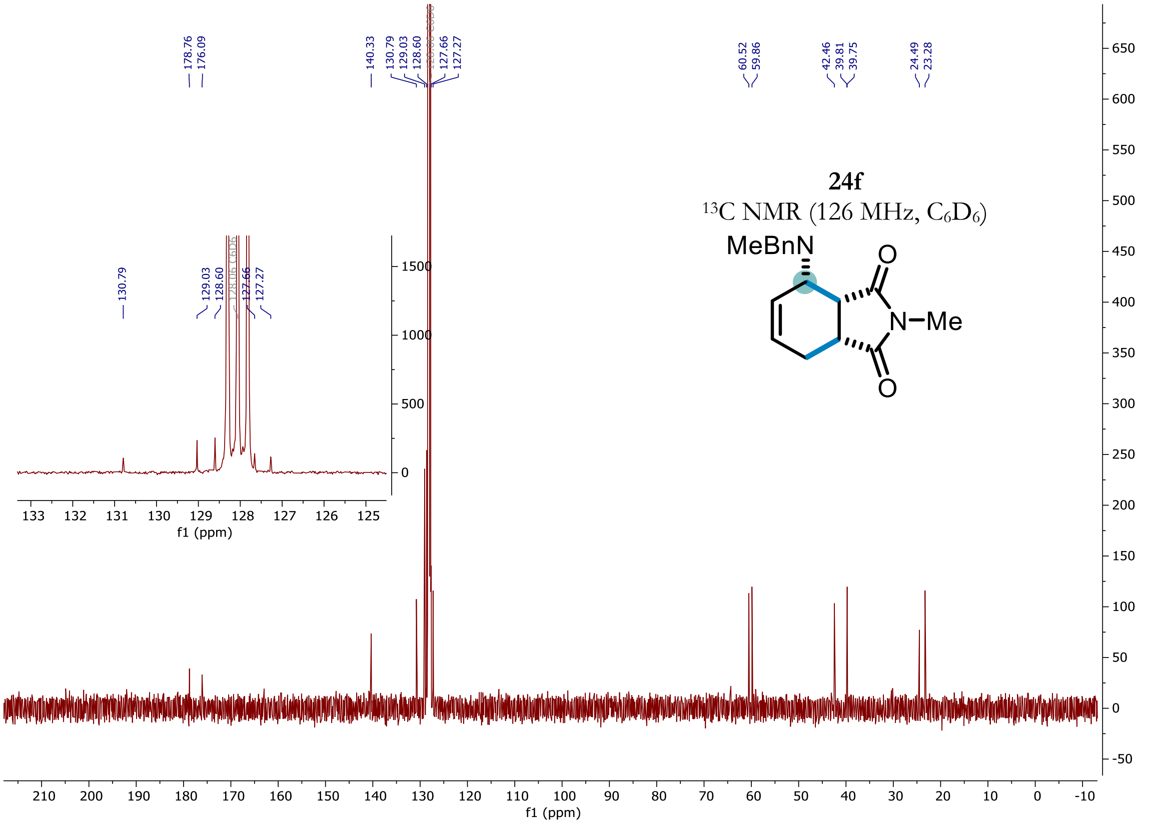
24f
13C NMR (126 MHz, C6D6)
MeBnN
O
N−Me
O
f1 (ppm)

27a

^{1}H NMR (400 MHz, $CDCl_3$)

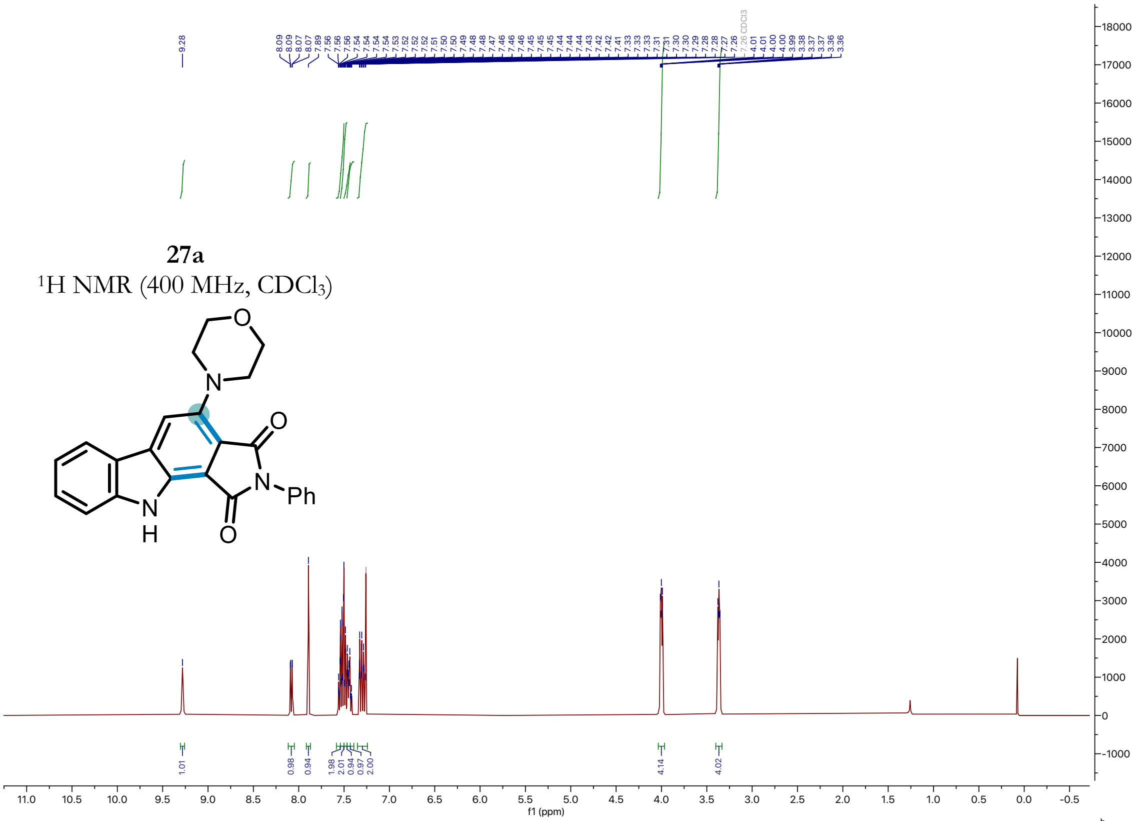

27a

^{1}H NMR (400 MHz, $CDCl_3$)

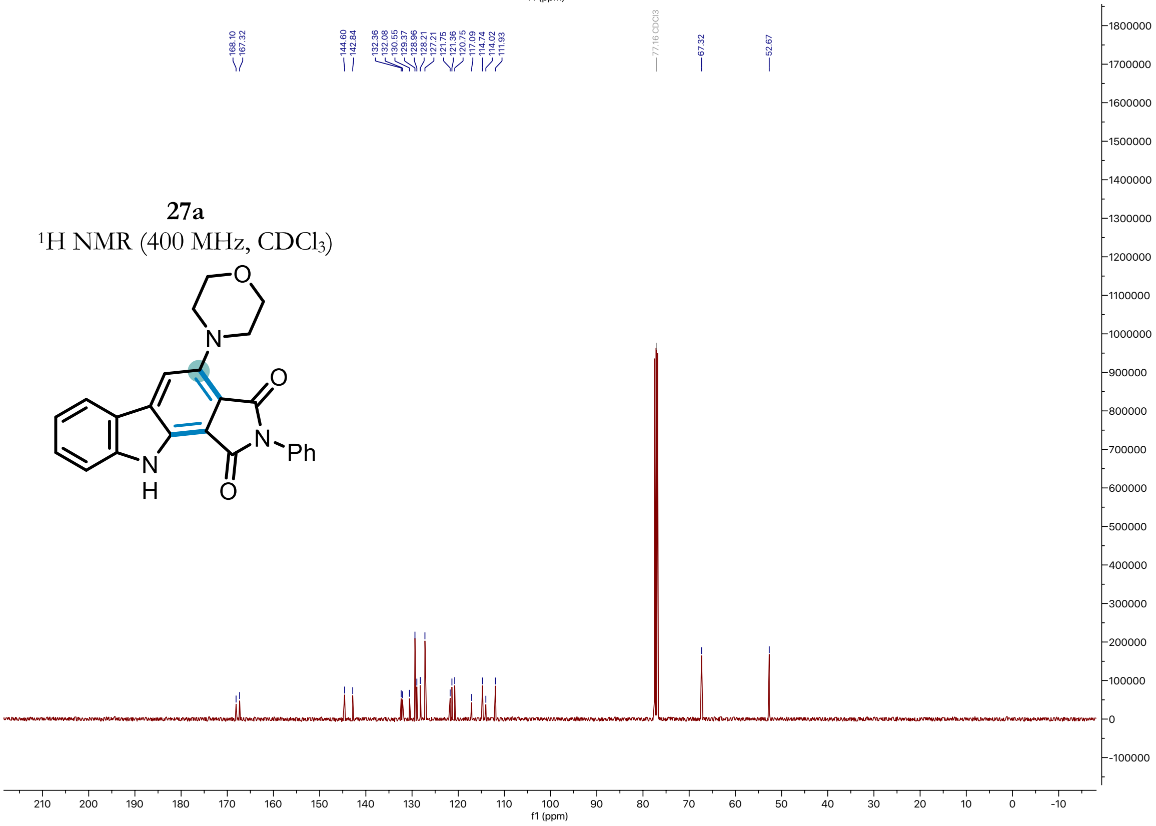

27b

^{1}H NMR (400 MHz, CDCl$_3$)

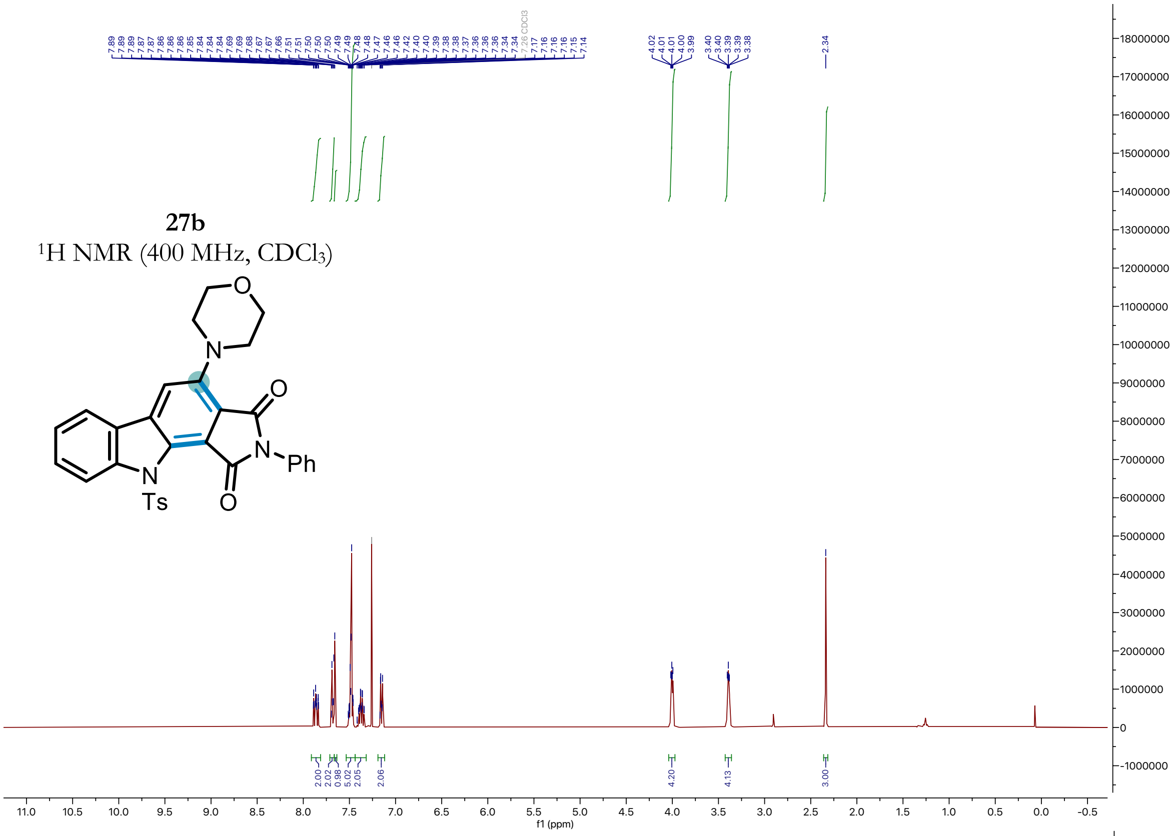

27b

^{13}C NMR (101 MHz, CDCl$_3$)

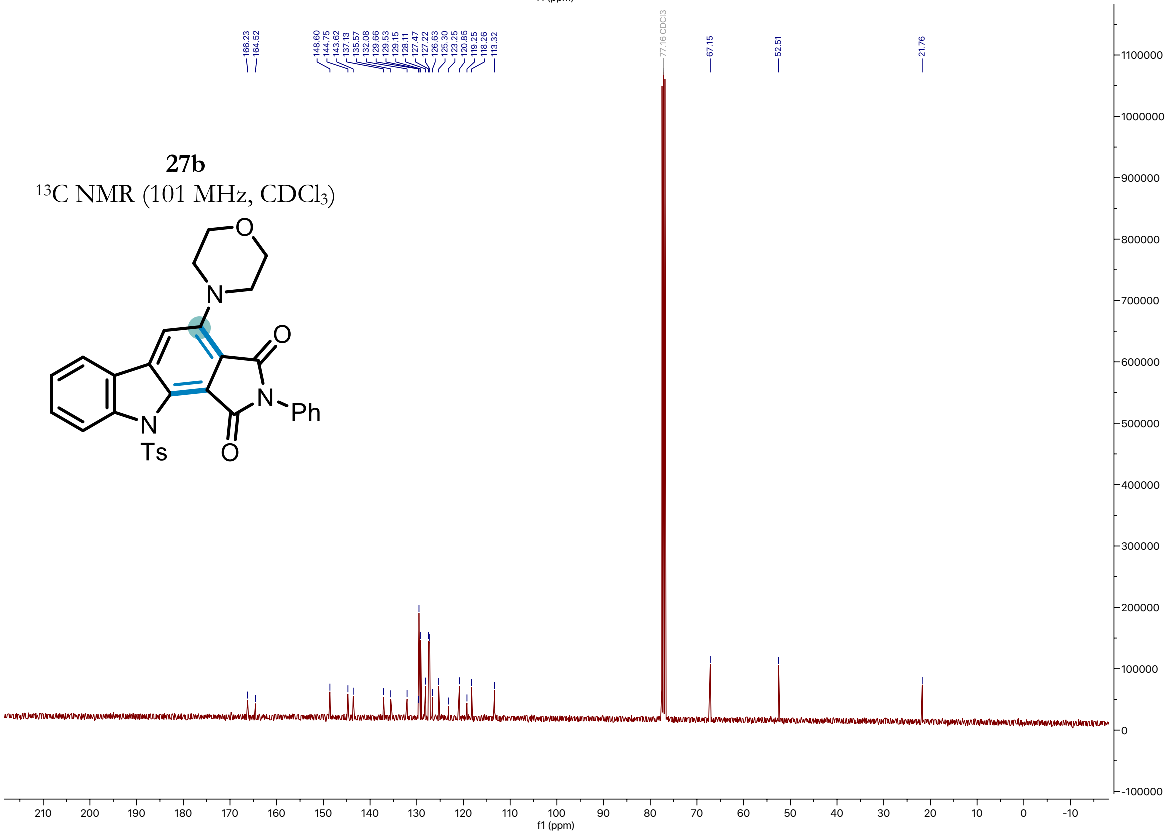

30

^{1}H NMR (400 MHz, C_6D_6)

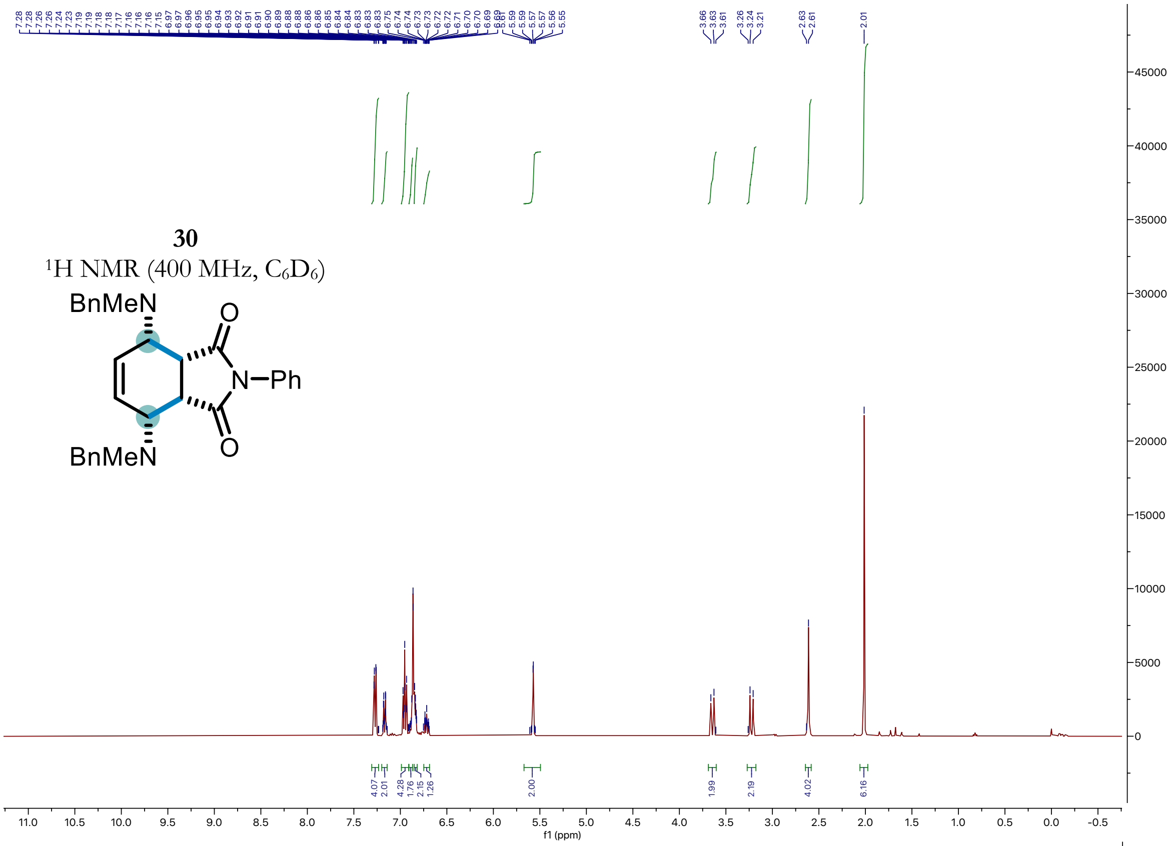

30

^{13}C NMR (101 MHz, C_6D_6)

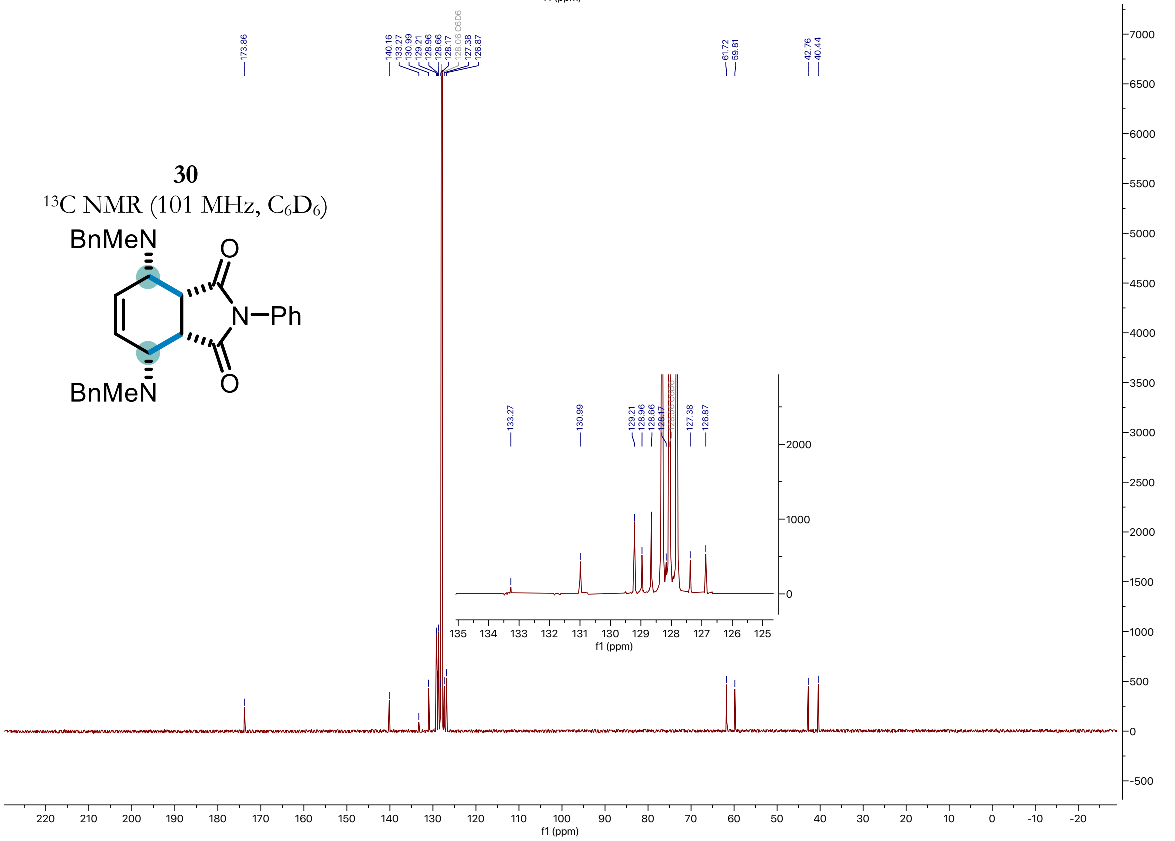

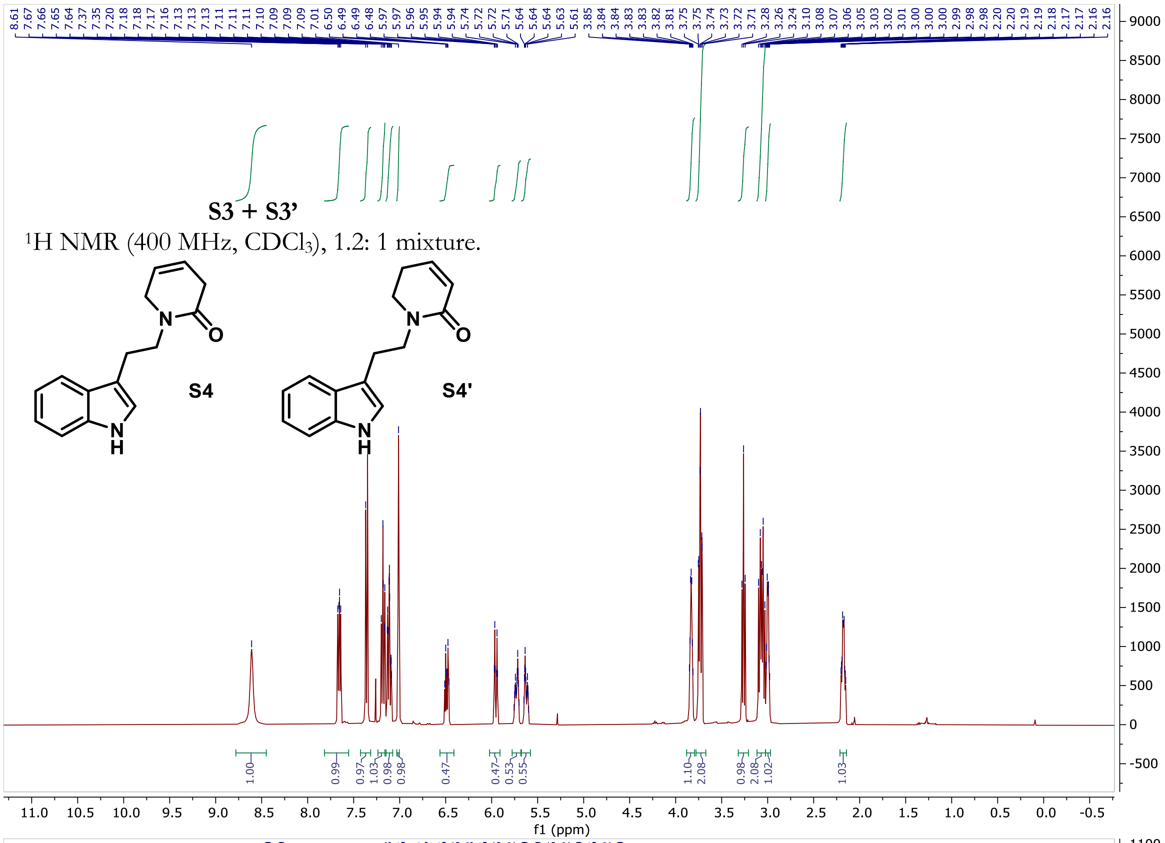
S3 + S3’
^{1}H NMR (400 MHz, CDCl$_3$), 1.2: 1 mixture.
S4
S4'
f1 (ppm)

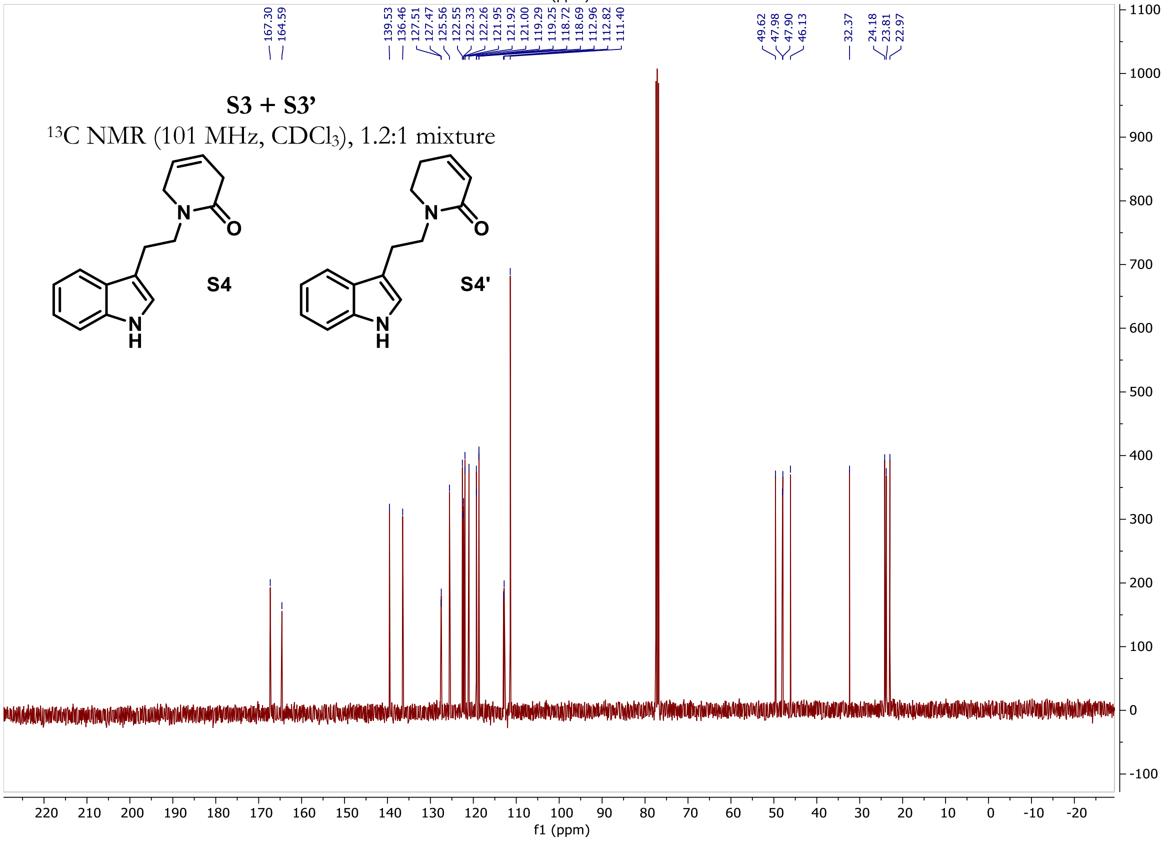
S3 + S3’
^{13}C NMR (101 MHz, CDCl$_3$), 1.2:1 mixture
S4
S4'
f1 (ppm)

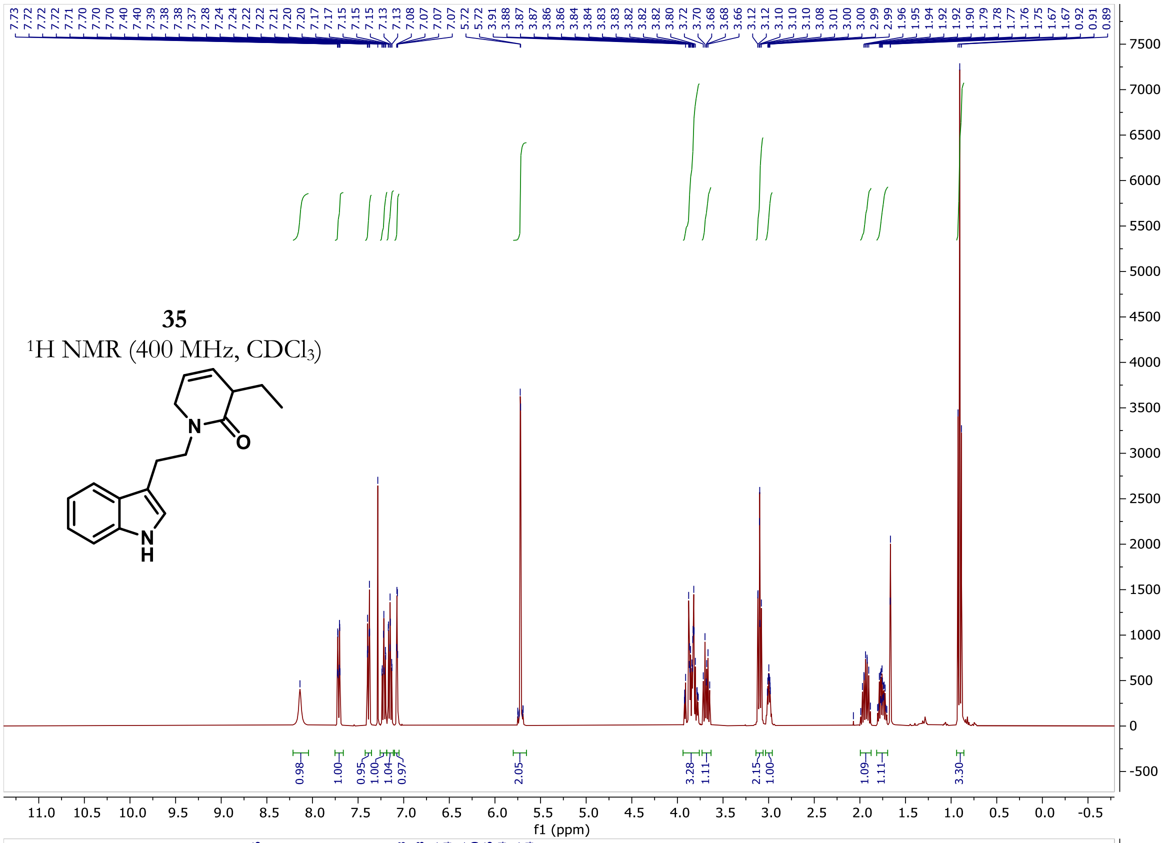
35
^{1}H NMR (400 MHz, CDCl$_3$)
f1 (ppm)

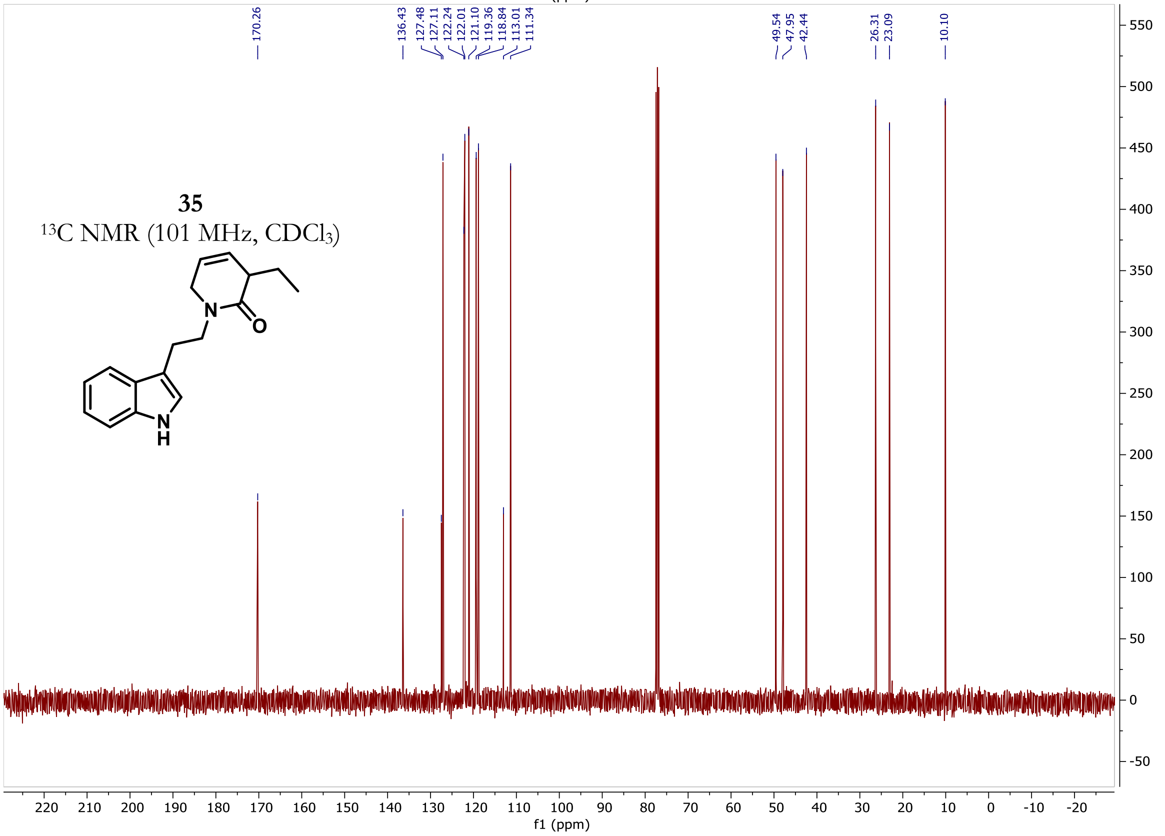
35
^{13}C NMR (101 MHz, CDCl$_3$)
f1 (ppm)

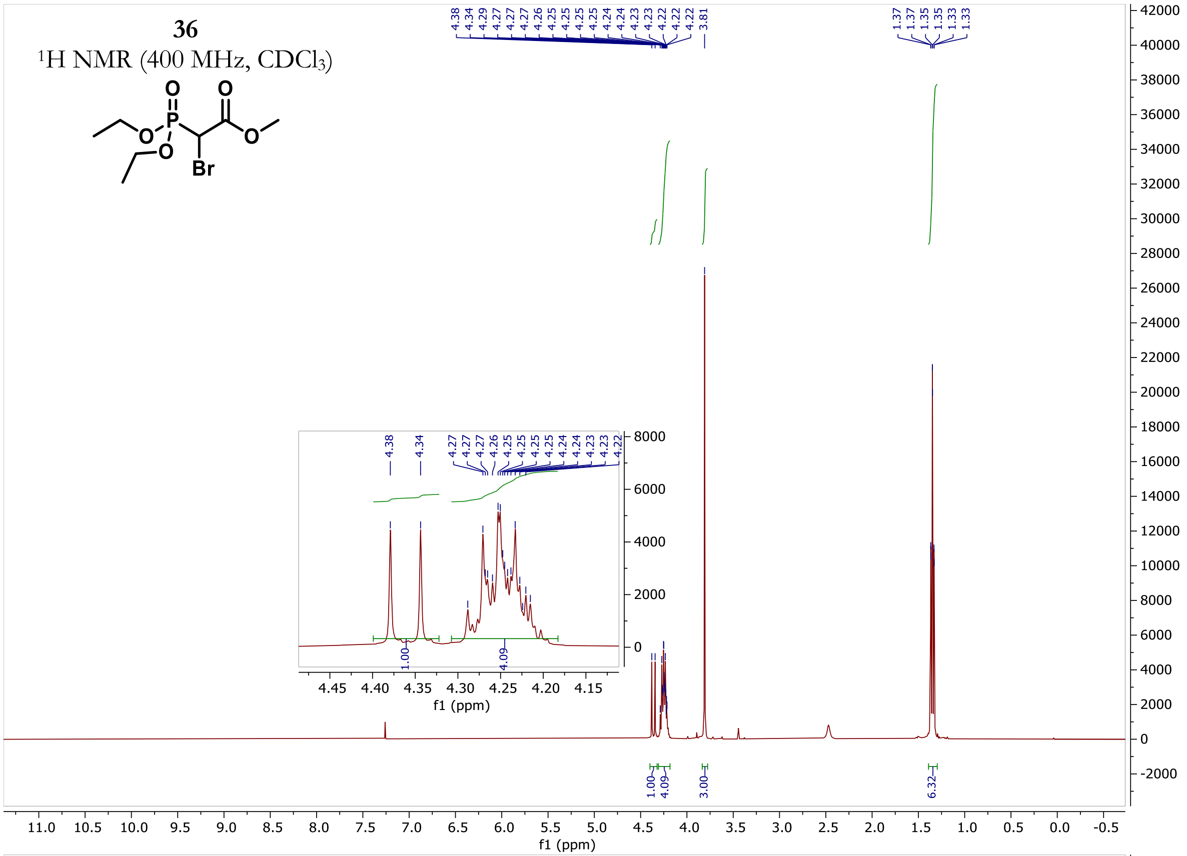
36
^{1}H NMR (400 MHz, CDCl$_3$)
O O P O O O Br
4.38 4.34 4.29 4.27 4.27 4.27 4.26 4.25 4.25 4.25 4.25 4.24 4.24 4.23 4.23 4.22 4.22 4.22 3.81
1.37 1.37 1.35 1.35 1.33 1.33
1.00 4.09 3.00 6.32
f1 (ppm)

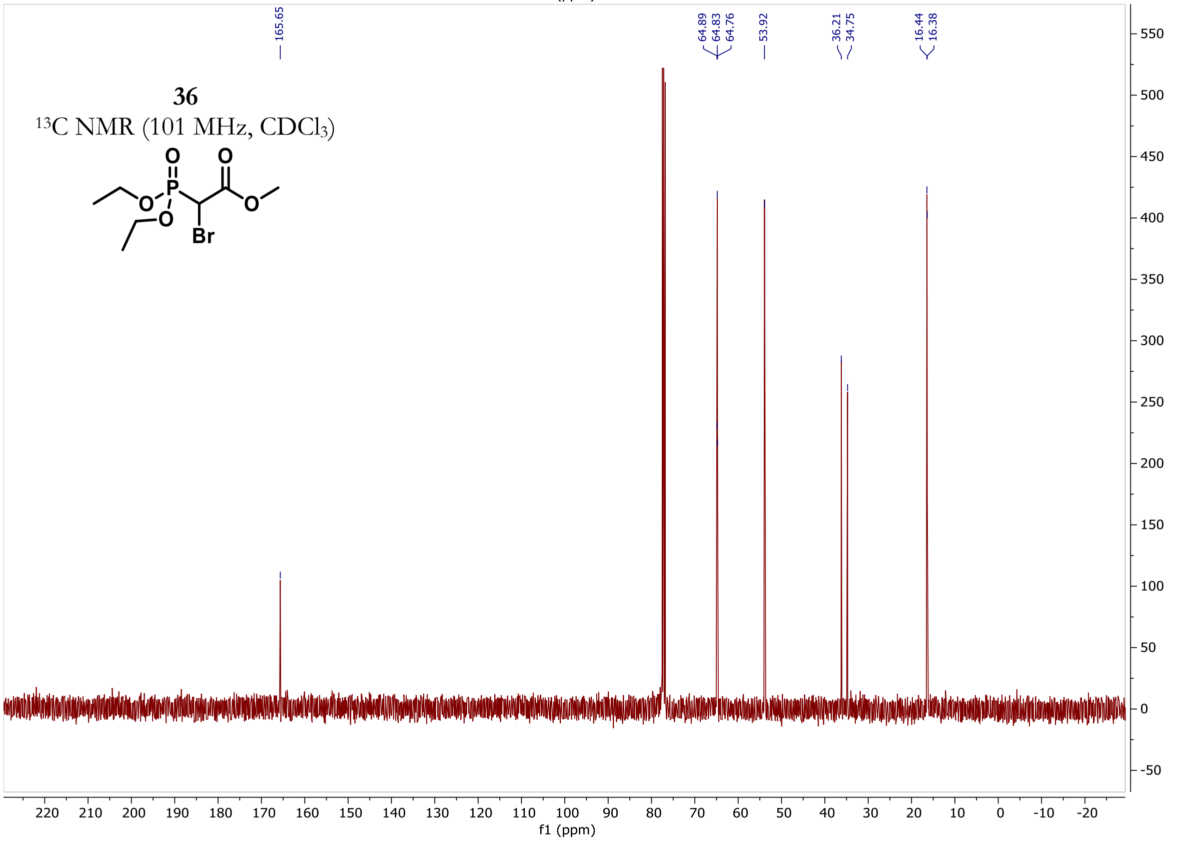
36
^{13}C NMR (101 MHz, CDCl$_3$)
O O P O O O Br
165.65
64.89 64.83 64.76
53.92
36.21 34.75
16.44 16.38
f1 (ppm)

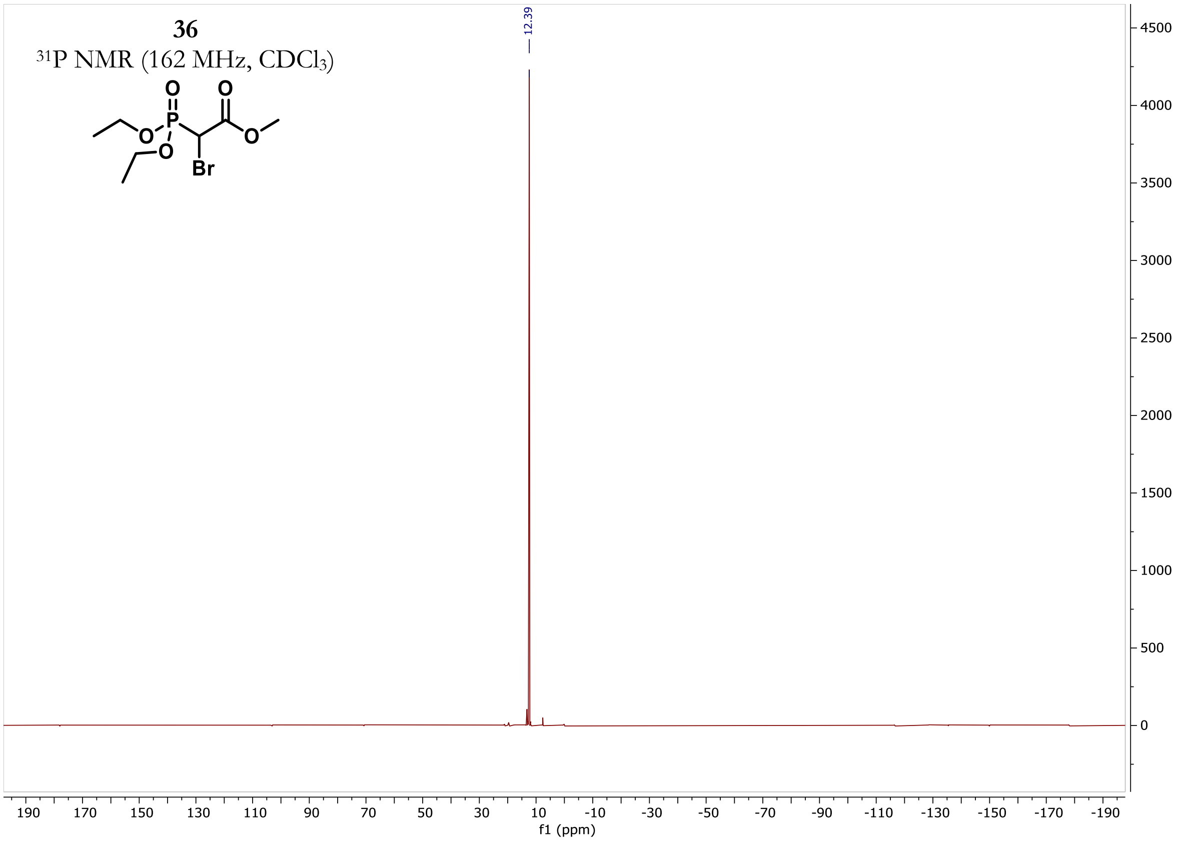
36
31P NMR (162 MHz, CDCl3)
Br
12.39
f1 (ppm)

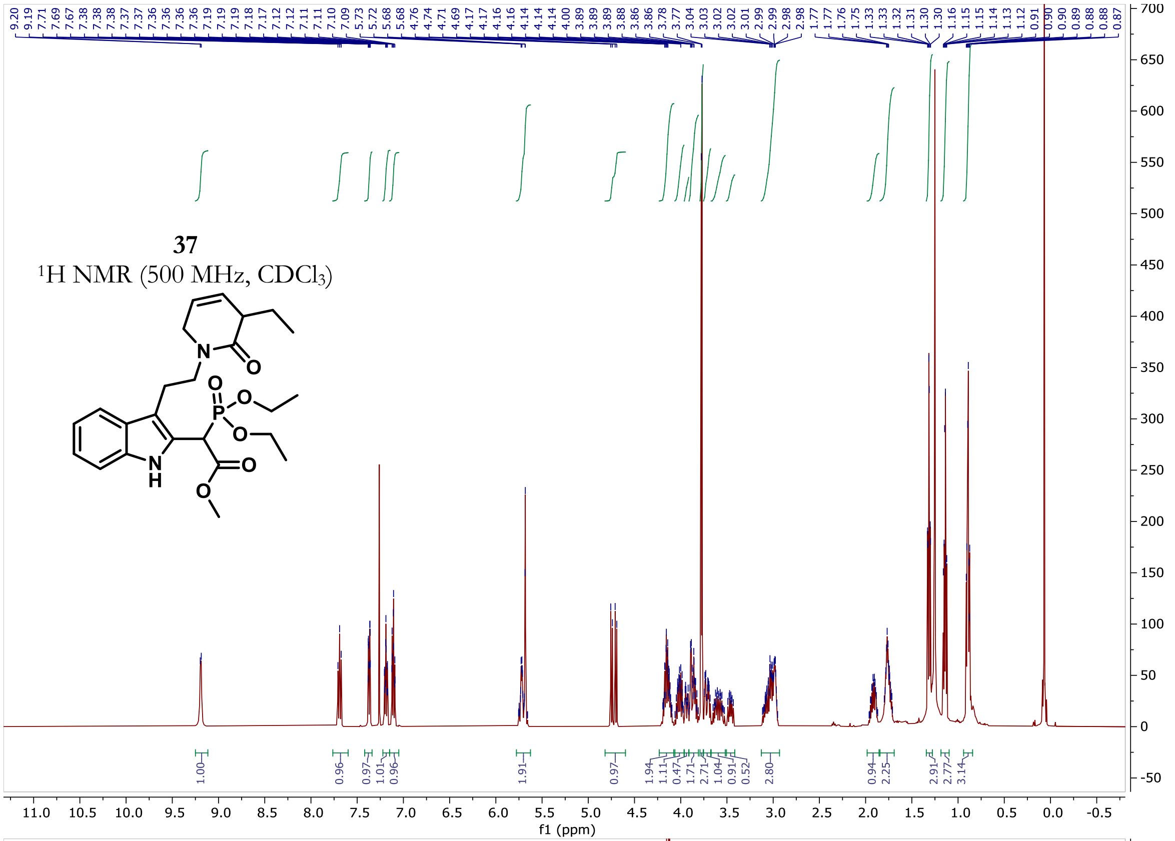
9.20 9.19 7.71 7.69 7.67 7.38 7.38 7.38 7.37 7.37 7.36 7.36 7.36 7.36 7.19 7.19 7.19 7.18 7.17 7.12 7.12 7.11 7.11 7.10 7.09 5.73 5.72 5.68 5.68 4.76 4.74 4.71 4.69 4.17 4.17 4.16 4.14 4.14 4.14 4.00 3.89 3.89 3.88 3.86 3.78 3.77 3.04 3.03 3.02 3.02 3.01 2.99 2.99 2.98 2.98 1.77 1.77 1.76 1.75 1.33 1.33 1.32 1.31 1.30 1.30 1.16 1.15 1.15 1.14 1.13 1.12 0.91 0.90 0.90 0.89 0.88 0.87
37
1H NMR (500 MHz, CDCl3)
1.00 0.96 0.97 1.01 0.96 1.91 0.97 1.94 1.11 0.47 1.71 2.71 1.04 0.91 0.52 2.80 0.94 2.25 2.91 2.77 3.14
f1 (ppm)

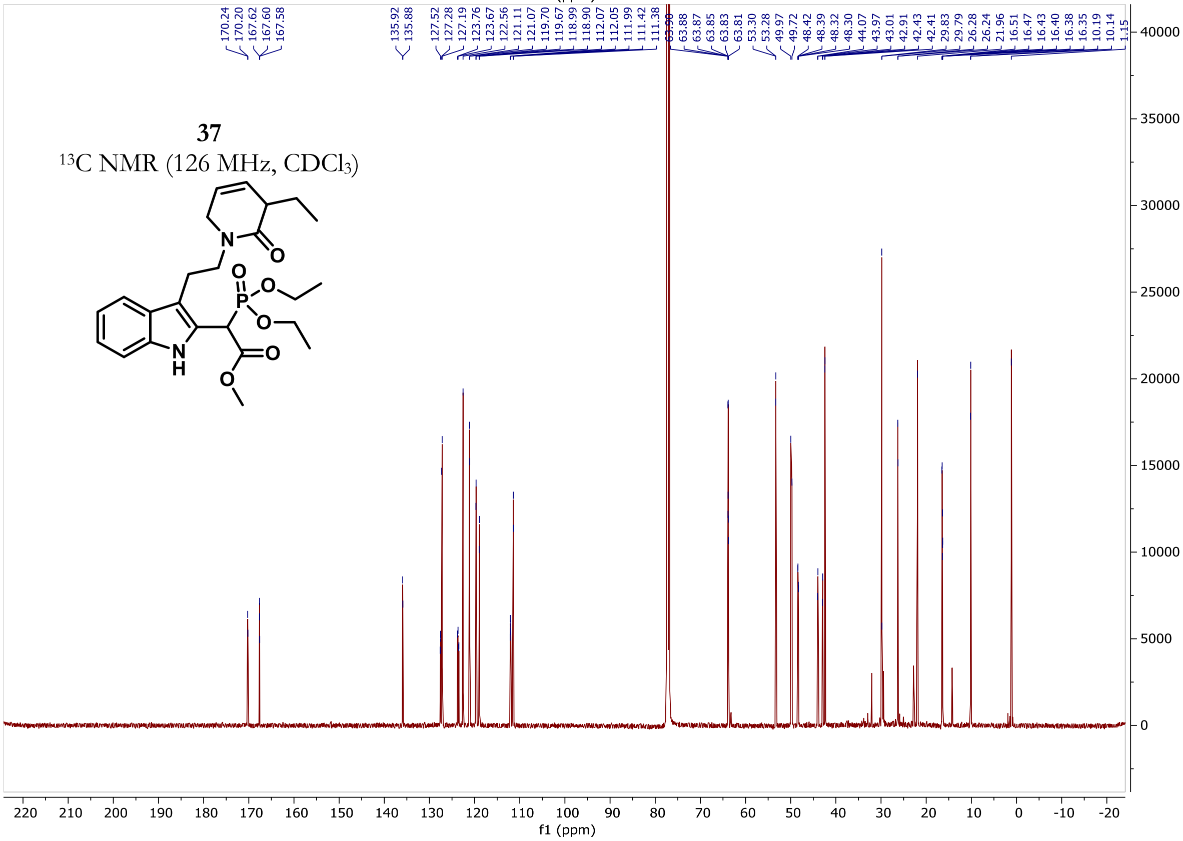
170.24 170.20 167.62 167.60 167.58 135.92 135.88 127.52 127.28 127.19 123.76 123.67 122.56 121.11 121.07 119.70 119.67 118.99 118.90 112.07 112.05 111.99 111.42 111.38 63.90 63.88 63.87 63.85 63.83 63.81 53.30 53.28 49.97 49.72 48.42 48.39 48.32 48.30 44.07 43.97 43.01 42.91 42.43 42.41 29.83 29.79 26.28 26.24 21.96 16.51 16.47 16.43 16.40 16.38 16.35 10.19 10.14 1.15
37
13C NMR (126 MHz, CDCl3)
f1 (ppm)

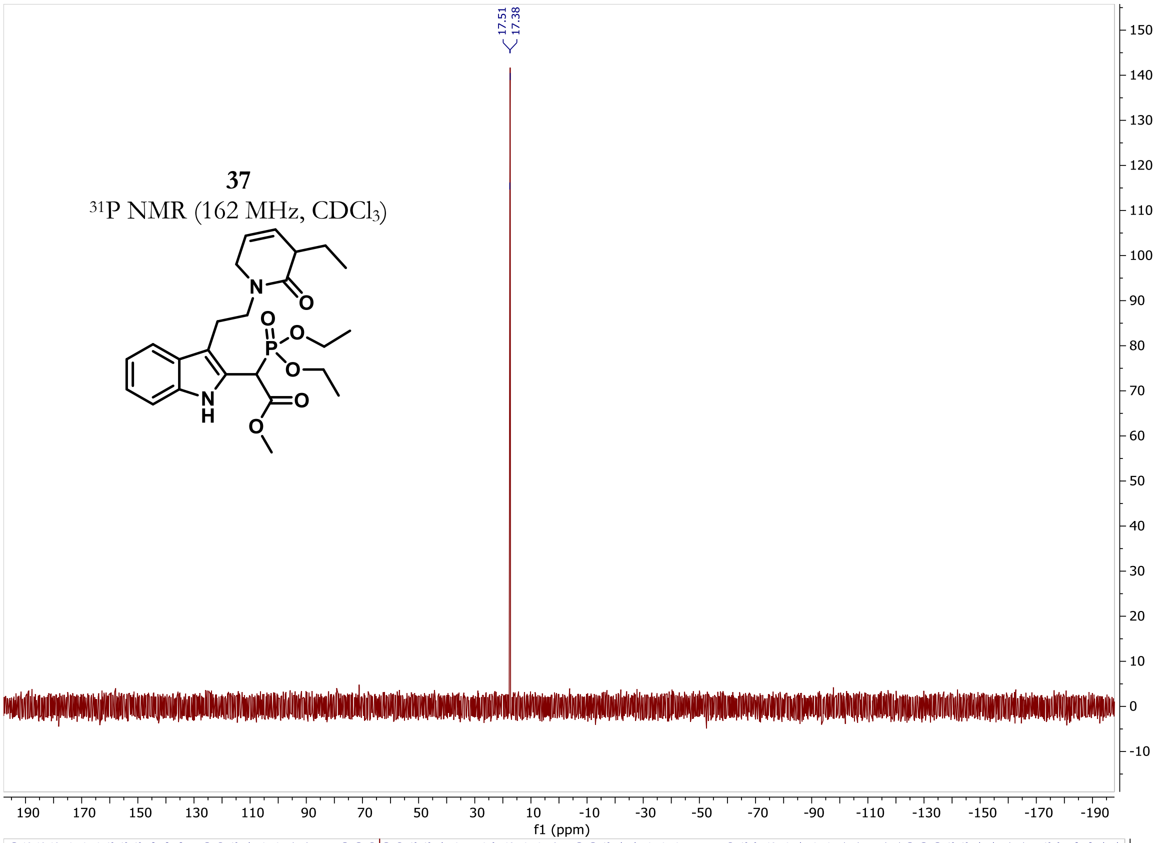
37
31P NMR (162 MHz, CDCl3)
17.51
17.38
f1 (ppm)

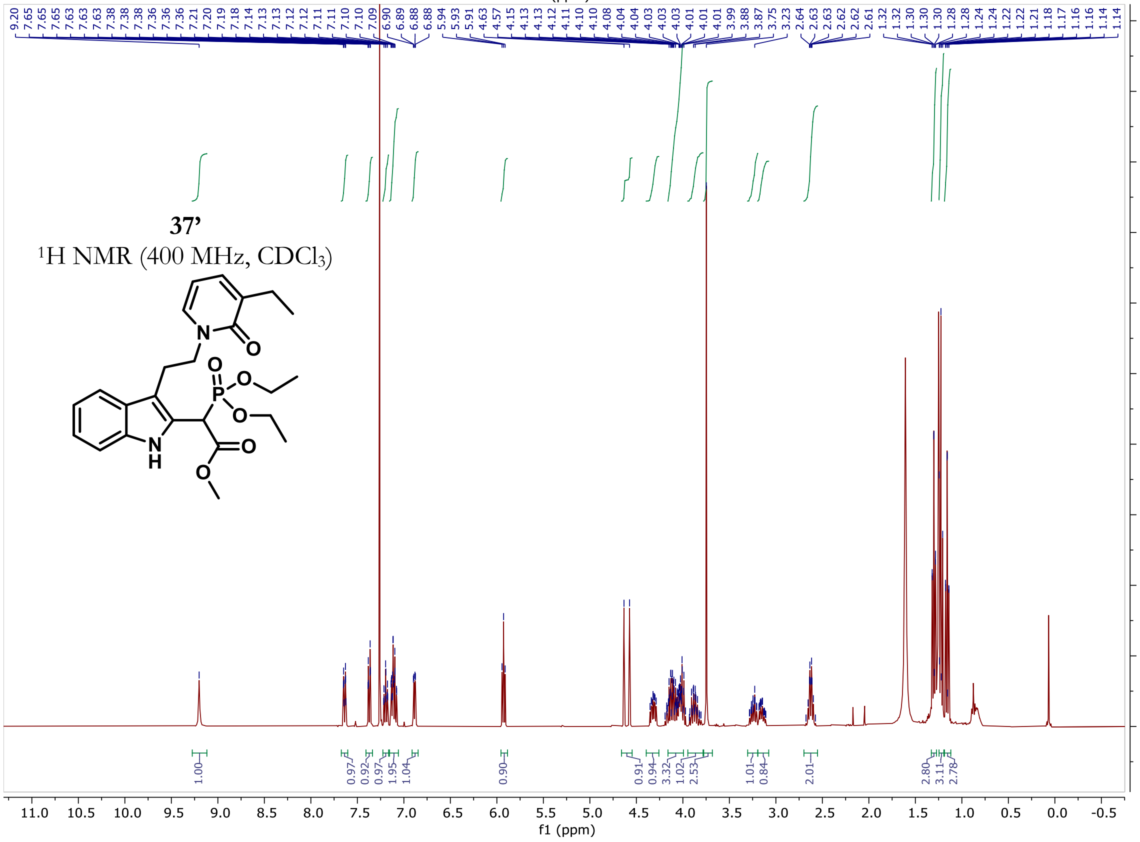
37’
1H NMR (400 MHz, CDCl3)
f1 (ppm)

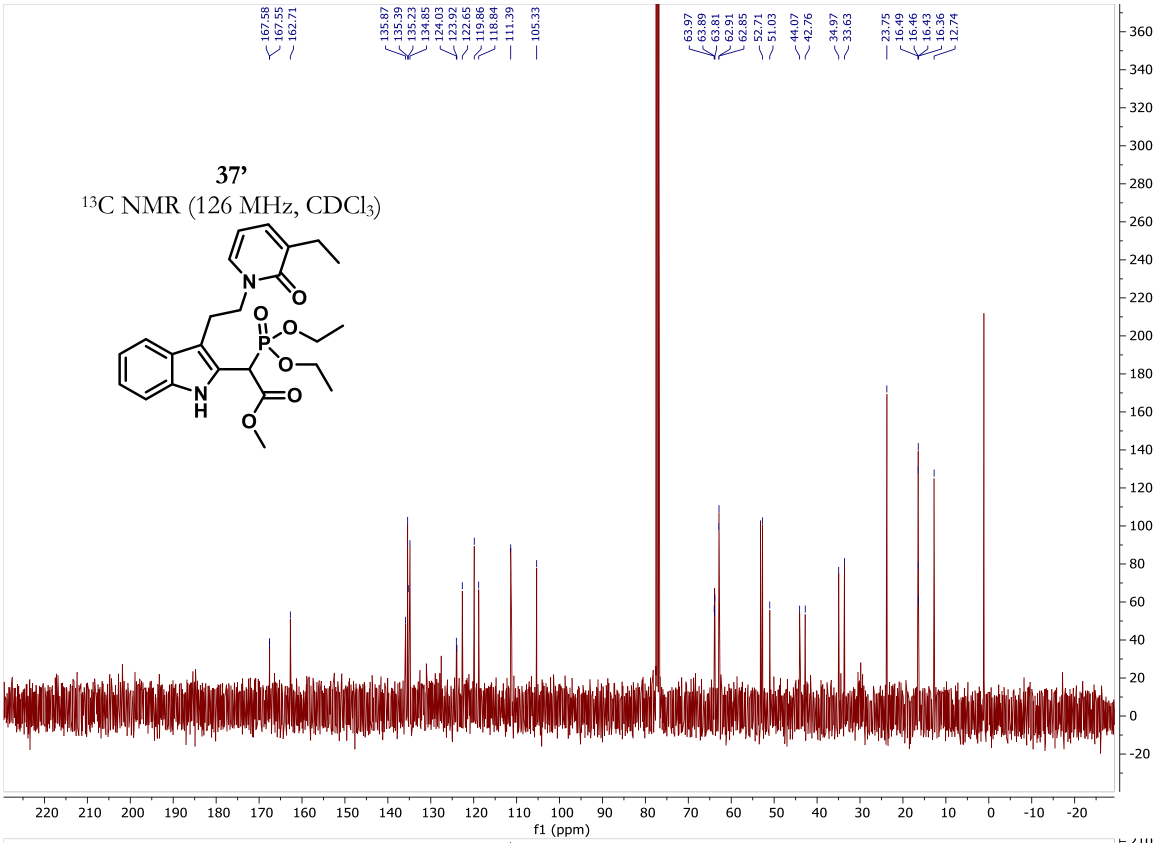

37’
^{13}C NMR (126 MHz, CDCl$_3$)
167.58
167.55
162.71
135.87
135.39
135.23
134.85
124.03
123.92
122.65
119.86
118.84
111.39
105.33
63.97
63.89
63.81
62.91
62.85
52.71
51.03
44.07
42.76
34.97
33.63
23.75
16.49
16.46
16.43
16.36
12.74
f1 (ppm)

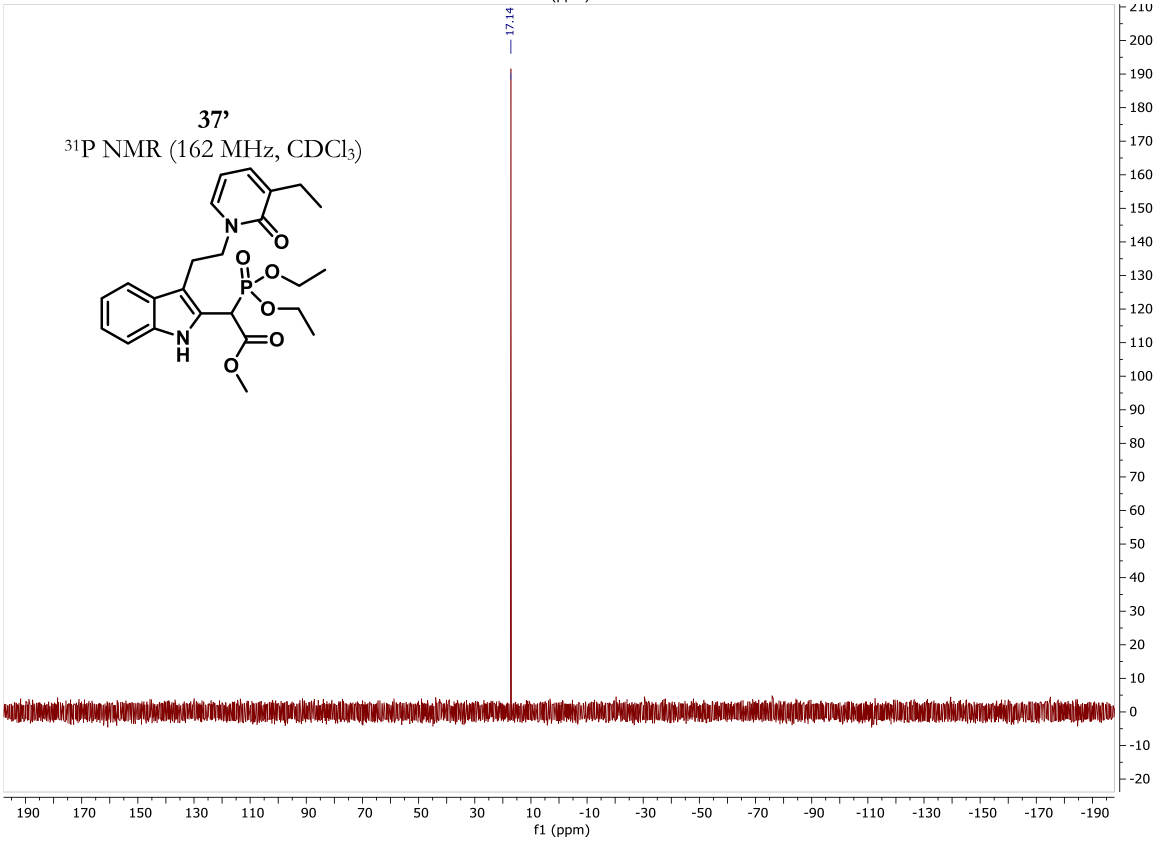

37’
^{31}P NMR (162 MHz, CDCl$_3$)
17.14
f1 (ppm)

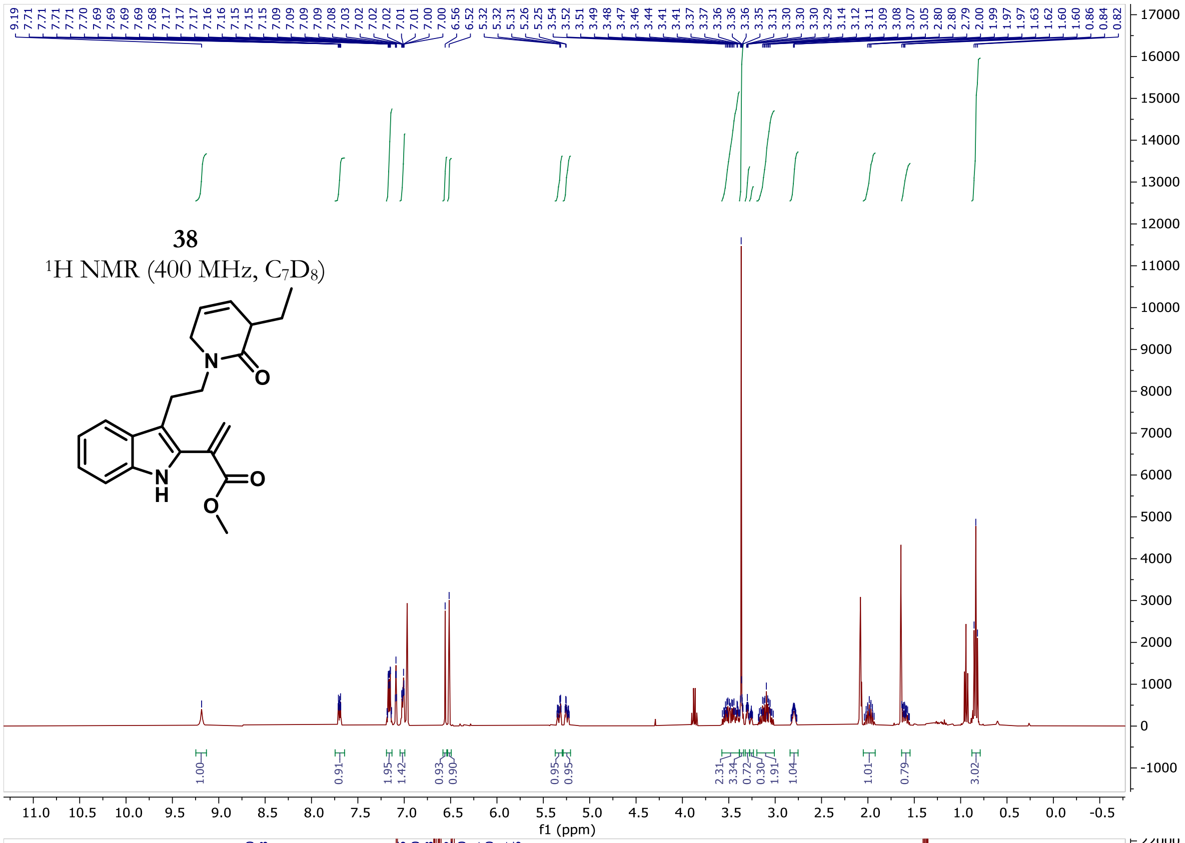
38
^{1}H NMR (400 MHz, C_7D_8)
f1 (ppm)

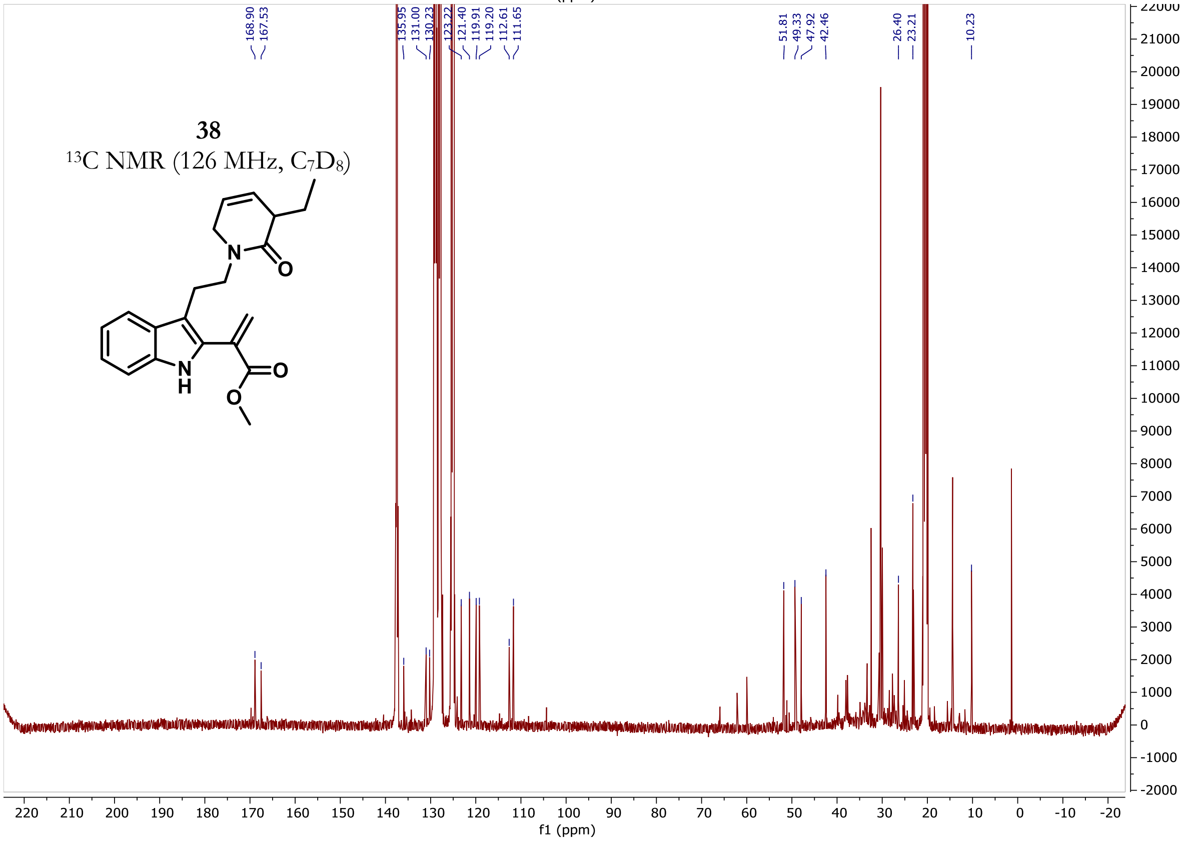
168.90
167.53
135.95
131.00
130.23
123.22
121.40
119.91
119.20
112.61
111.65
51.81
49.33
47.92
42.46
26.40
23.21
10.23
38
^{13}C NMR (126 MHz, C_7D_8)
f1 (ppm)

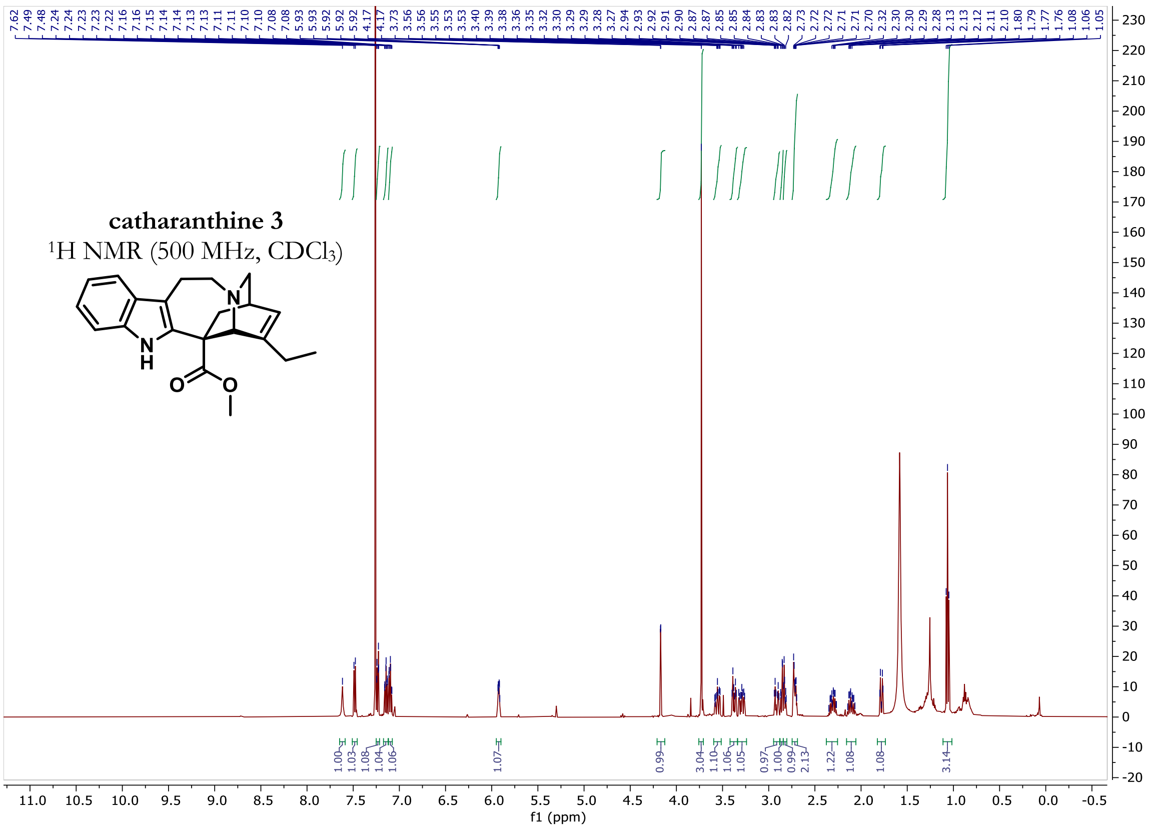
catharanthine 3
1H NMR (500 MHz, CDCl3)
7.62 7.49 7.48 7.24 7.24 7.23 7.23 7.22 7.16 7.16 7.15 7.14 7.14 7.13 7.13 7.11 7.11 7.10 7.10 7.08 7.08 5.93 5.93 5.92 5.92 5.92 4.17 4.17 3.73 3.56 3.56 3.55 3.53 3.53 3.40 3.39 3.38 3.36 3.35 3.32 3.30 3.29 3.29 3.28 3.27 2.94 2.93 2.92 2.91 2.90 2.87 2.87 2.85 2.85 2.84 2.83 2.83 2.82 2.73 2.73 2.72 2.72 2.71 2.71 2.70 2.32 2.30 2.30 2.29 2.28 2.13 2.13 2.12 2.11 2.10 1.80 1.79 1.77 1.76 1.08 1.06 1.05
1.00 1.03 1.08 1.04 1.06 1.07 0.99 3.04 1.10 1.06 1.05 0.97 1.00 0.99 2.13 1.22 1.08 1.08 3.14
f1 (ppm)

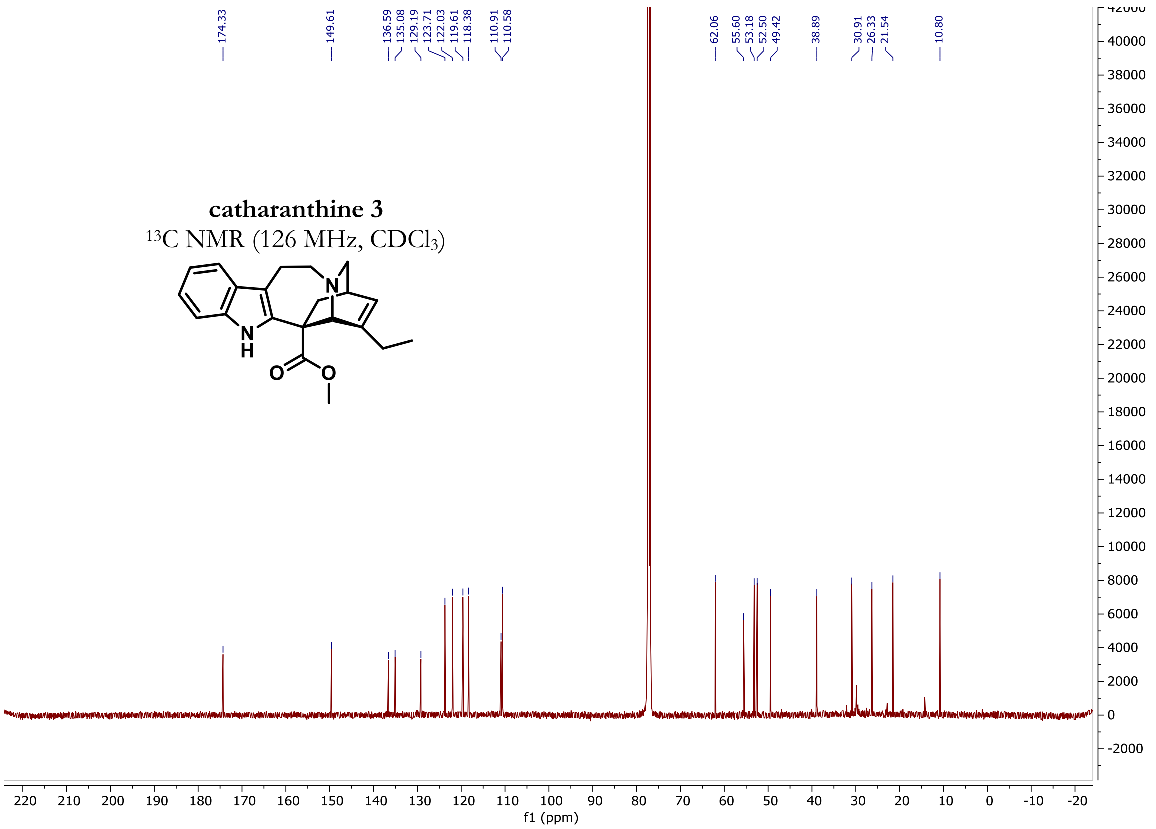
catharanthine 3
13C NMR (126 MHz, CDCl3)
174.33 149.61 136.59 135.08 129.19 123.71 122.03 119.61 118.38 110.91 110.58 62.06 55.60 53.18 52.50 49.42 38.89 30.91 26.33 21.54 10.80
f1 (ppm)

11. References:

1. Zhao, Z.; Duesler, E.; Wang, C.; Guo, H.; Mariano, P. S. Photocyclization reactions of cyclohexa-and cyclopenta-fused pyridinium salts. Factors governing regioselectivity. *J. Org. Chem.* **2005**, *70* (21), 8508-8512.
2. Huang, L.; Gu, Y.; Fürstner, A. Iron-Catalyzed Reactions of 2-Pyridone Derivatives: 1,6-Addition and Formal Ring Opening/Cross Coupling. *Chem. Asian J.* **2019**, *14* (22), 4017-4023.
3. Firsov, A.; Chupakhin, E.; Dar'in, D.; Bakulina, O.; Krasavin, M. Three-Component Castagnoli–Cushman Reaction of 3-Arylglutaconic Acids with Aromatic Aldehydes and Amines Delivers Rare 4,6-Diaryl-1,6-dihydropyridin-2(3 H)-ones. *Org. Lett.* **2019**, *21* (6), 1637-1640.
4. Andrade, C. K. Z.; Rocha, R. O.; Vercillo, O. E.; Silva, W. A.; Matos, R. A. F. DCC/DMAP-Mediated Coupling of Carboxylic Acids with Oxazolidinones and Thiazolidinethiones. *Synlett* **2003**, *15*, 2351-2352.
5. Derosa, J.; Kleinmans, R.; Tran, V. T.; Karunananda, M. K.; Wisniewski, S. R.; Eastgate, M. D.; Engle, K. M. Nickel-Catalyzed 1,2-Diarylation of Simple Alkenyl Amides. *J. Am. Chem. Soc.* **2018**, *140* (51), 17878-17883.
6. Nakagawa, M.; Tonozuka, M.; Obi, M.; Kiuchi, T.; Hino, T. Synthesis of 5,6-Dihydro-2-pyrone and 2-Pyrone. *Synthesis* **1974**, 510.
7. Yasui, Y.; Kakinokihara, I.; Takeda, H.; Takemoto, Y. Preparation of α, β-Unsaturated Lactams through Intramolecular Electrophilic Carbamoylation of Alkenes. *Synthesis* **2009**, *23*, 3989-3993.
8. Nguyen, V. T.; Nguyen, V. D.; Haug, G. C.; Dang, H. T.; Jin, S.; Li, Z.; Flores-Hansen, C.; Benavides, B. S.; Arman, H. D.; Larionov, O. V. Alkene Synthesis by Photocatalytic Chemoenzymatically Compatible Dehydrodecarboxylation of Carboxylic Acids and Biomass. *ACS Catalysis* **2019**, *9* (10), 9485-9498.
9. Lin, L.; Romano, C.; Mazet, C. Palladium-Catalyzed Long-Range Deconjugative Isomerization of Highly Substituted α,β-Unsaturated Carbonyl Compounds. *J. Am. Chem. Soc.* **2016**, *138* (32), 10344-10350.
10. Reding, M. T.; Fukuyama, T. Stereocontrolled Total Synthesis of (±)-Catharanthine via Radical-Mediated Indole Formation. *Org. Lett.* **1999**, *1* (7), 973-976.
